# The diversity of ACBD proteins – From lipid binding to protein modulators and organelle tethers

**DOI:** 10.1016/j.bbamcr.2020.118675

**Published:** 2020-05

**Authors:** Markus Islinger, Joseph L. Costello, Suzan Kors, Eric Soupene, Timothy P. Levine, Frans A. Kuypers, Michael Schrader

**Affiliations:** a Institute of Neuroanatomy, Medical Faculty Manheim, University of Heidelberg, 68167 Mannheim, Germany; b College of Life and Environmental Sciences, Biosciences, University of Exeter, Exeter EX4 4QD, Devon, UK; c Children's Hospital Oakland Research Institute, Oakland, CA 94609, USA; d UCL Institute of Ophthalmology, London EC1V 9EL, UK

**Keywords:** ACBD, acyl-CoA binding domain containing protein, ACB, acyl-CoA binding domain, ACBP, acyl-CoA binding protein, ER, endoplasmic reticulum, FFAT, two phenylalanines (FF) in an acidic tract, GABA, gamma-aminobutyric acid, GOLD, Golgi dynamic, LCFA, long-chain fatty acid, MCS, membrane contact site, MTS, mitochondrial targeting sequence, POMC, pro-opiomelanocortin, PTS, peroxisomal targeting signal, RO, replication organelle, TMD, transmembrane domain, VAP, vesicle-associated membrane protein (VAMP)–associated protein, VLCFA, very long-chain fatty acid, Acyl-CoA binding domain containing protein, Peroxisomes, Lipid metabolism, Membrane contact sites, FFAT motif, Pathogen host interaction

## Abstract

Members of the large multigene family of acyl-CoA binding domain containing proteins (ACBDs) share a conserved motif required for binding of Coenzyme A esterified fatty acids of various chain length. These proteins are present in the three kingdoms of life, and despite their predicted roles in cellular lipid metabolism, knowledge about the precise functions of many ACBD proteins remains scarce. Interestingly, several ACBD proteins are now suggested to function at organelle contact sites, and are recognized as host interaction proteins for different pathogens including viruses and bacteria. Here, we present a thorough phylogenetic analysis of the ACBD family and discuss their structure and evolution. We summarize recent findings on the various functions of animal and fungal ACBDs with particular focus on peroxisomes, the role of ACBD proteins at organelle membranes, and their increasing recognition as targets for pathogens.

## Highlights

• Acyl-CoA binding domain-containing proteins (ACBDs) can be found among eukaryotes and prokaryotes.
• Additional protein interaction domains are a characteristic of most extended ACBDs.
• A set of a small ACBD and an ACBD with a C-terminal ankyrin-repeat motif is found in all eukaryote branches.
• Fungi and metazoans contain genes for ACBD5/ATG37 but the proteins do not appear to possess conserved functions
• Several extended metazoan ACBDs are hijacked by viruses in order to facilitate effective viral replication.

## Graphical abstract

**Figure:**
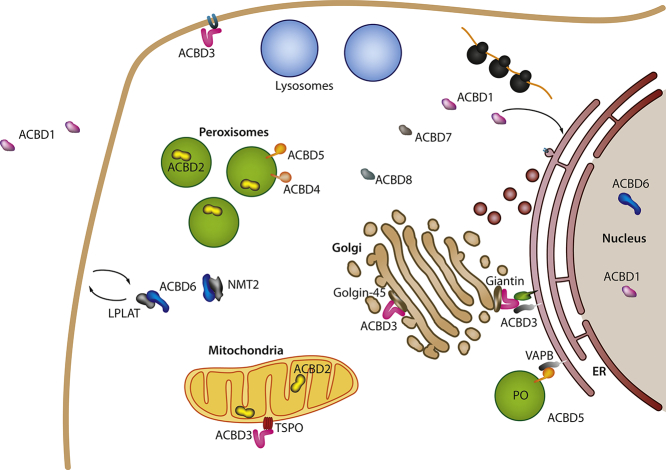

## 1 Introduction

Acyl-CoA binding domain containing proteins (ACBDs) comprise a large multigene family of diverse proteins that are defined by the presence of a conserved 80 residues-long acyl-CoA binding motif (ACB) [1,2]. Activated fatty acids play important roles as lipid metabolites, but also in the regulation of lipid metabolism and in cellular signaling [1,3], and ACBD proteins fulfill important functions in controlling their concentration. In mammals, seven ACBD proteins (ACBD1–7) have been specified so far (see Section 2 for ACBD8), often with different transcript variants [1]. Those include small, soluble proteins (ACBD1, ACBD7), but also large, multifunctional enzymes (ACBD2), membrane proteins (ACBD4, ACBD5), and proteins with a GOLD (Golgi dynamics)-domain (ACBD3) or ankyrin repeats (ACBD6). Homologous ACBD proteins are found in animals, plants and fungi, but also in bacteria and archaea (see Section 2). Despite their predicted roles in cellular lipid metabolism, the functions of many of the ACBD proteins still remain unclear. Interestingly, several of the ACBD proteins have recently been linked to peroxisome function [[4], [5], [6], [7], [8], [9], [10]]. Peroxisomes are ubiquitous membrane-bound organelles with key roles in lipid metabolism, including the breakdown and detoxification of fatty acids (via fatty acid α- and β-oxidation), and the synthesis of ether-phospholipids (e.g. plasmalogens enriched in myelin sheaths), bile acids and polyunsaturated fatty acids (PUFAs) such as docosahexaenoic acid in humans. Defects in peroxisome biogenesis and metabolic function cause severe disorders with developmental and neurological defects [[11], [12], [13]]. Peroxisomal lipid metabolism requires cooperation and interaction with other organelles such as the endoplasmic reticulum (ER), mitochondria or lipid droplets [14]. Those peroxisome-organelle interactions are mediated by membrane contact sites (MCSs), which involve membrane-bound tether proteins to bring organelles in close apposition [[15], [16], [17]]. ACBD2, ACBD4, ACBD5 are now suggested to function as peroxisome-organelle tethers thus contributing to the formation of lipid hubs which facilitate lipid metabolism at the organelle interface [17]. ACBD3 was reported to function at Golgi-ER contact sites [18]. Importantly, organelle-associated ACBDs are now increasingly recognized as targets for different pathogens including viruses, *Salmonella* and *Chlamydia* [[19], [20], [21], [22]]. These recent discoveries underline that ACBD proteins may have evolved to fulfill functions other than acyl-CoA buffering. Based on a comprehensive phylogenetic analysis, we will discuss the evolution of the structurally diverse ACBD proteins and will summarize their functions. We will also focus on the functions of ACBD proteins in peroxisome metabolism and at membrane contact sites.

## 2 Structure and evolution of the ACBD family

### 2.1 Structure and function of the ACB core domain

The ACB domain is a phylogenetically very ancient protein structure, which is found in all eukaryote branches. ACBDs which only carry the ACB domain are small proteins (≈10–15 kDa) and are distributed among metazoans, fungi and plants, but are also found in eubacteria and archaea (see below). Thus, in general they appear to be the origin of all further extended forms of the protein family. In animals, these ACBD forms are represented by the acyl-CoA binding protein ACBD1 (ACBP, DBI), which preferentially binds long-chain acyl-CoAs [23,24]. Structurally, the ACB domain consists of a bundle of four α-helices. They form a bowl-like structure which has a largely non-polar cavity, lined by polar amino acids [25,26]. In its monomeric form, the acyl-chain is located in a bent conformation inside the cavity and is covered by the CoA group shielding it from the surrounding solvent [25]. When two ACBD1 are assembled into a dimer, the 3′-phosphate-AMP moiety of acyl-CoA is bound in the binding pocket of one ACBD1 molecule and the acyl chain is bound in the pocket of the other ACBD1 molecule [27]. According to the domain structures available at the Protein Data Bank [28], the ACB domain structures from small and extended ACBD proteins are conserved to a large extent. However, the individual ACBDs show particular variations in helix conformational assembly suggesting distinctly modified binding pockets which might result in differences in substrate specificities [29].

Functionally, the small soluble forms may act as acyl-CoA transporters and reservoirs, guaranteeing efficient provision and targeting of acyl-CoA to lipid metabolizing subcellular compartments. For example, earlier theoretical calculations on the cytosolic free and protein-bound concentrations of long-chain fatty acid (LCFA)-CoA imply that the predominant proportion of LCFA-CoA is bound to proteins with acyl-CoA binding capacity and potentially mainly to ACBD1 [30]. In this regard, ACBD1 may: i) stabilize LCFA-CoA preventing auto- or enzymatic hydrolysis [31,32], ii) prevent LCFA-CoAs from partitioning into cellular lipid bilayers, iii) extract membrane-inserted LCFA-CoAs for delivery to cellular sites of lipid metabolism, iv) hand over LCFA-CoAs to respective metabolizing enzymes or v) control stimulatory/inhibitory effects of LCFA-CoAs on lipid metabolizing proteins or lipid-binding receptors [30,33,34]. While these all seem logical roles, direct evidence in complex biological systems is lacking. After their formation by acyl-CoA synthetases [35], acyl-CoAs are either used for the generation of triglycerides and membrane lipids (e.g. phospholipids), for fatty acid elongation (e.g. to generate PUFAs), or degraded via mitochondrial or peroxisomal β-oxidation and are thus distributed between a set of pools in the cell, including membranes, lipid droplets, and other lipid binding proteins. As evidenced by previous and our own phylogenetic reconstructions [36], during the evolution of eukaryotes, these small ACBDs evolved by genomic and structural variation to include different C-terminal or N-terminal extensions. These extended ACBD proteins acquired diverse functions and subcellular locations (Table 1, Table 2). It can be speculated that such extensions might have been driven by a need to link the ACB domains to specific cellular locations (membranes, proteins, organelles) and locally control acyl-CoA concentration as required by metabolic pathways or regulate the function of proteins present at those locations.

**Table 1:** Cellular localization and proposed functions of mammalian ACBDs.^a^

ACBD protein	Cellular localization	Proposed functions	Disease
ACBD1 (synonyms: ACBP, DBI, endozepine)	Primarily cytosolic, ER, Golgi, mitochondria, nucleus [1,36,67]	Regulation of acyl-CoA transport; Modulation of acyl-CoA-regulated enzymes; Fatty acid synthesis and degradation; Steroid, bile acid and complex lipid synthesis; Synaptic neuropeptide (endozepine); GABA receptor modulation; Control of feeding behaviour and energy expenditure via the melanocortin system [1,36,88,89,96,69,[73], [74], [75],[84], [85], [86], [87]]	May influence social behaviour, learning, anxiety, feeding behaviour (rodents) [[90], [91], [92], [93], [94], [95]] Obesity? [96,97] Anorexia nervosa? [97] (OMIM 606788)
ACBD2 (synonyms: ECI2, PECI, D3,D2-enoyl-CoA isomerase, DBI-related protein 1)	Soluble, peroxisomes (matrix), mitochondria [104]	Δ3,Δ2-enoyl-CoA isomerase involved in PO β-oxidation; PO-mitochondria tether (?) [10,107]	Up-regulated in prostate cancer [109]
ACBD3 (synonyms: GCP60, GOCAP1, GOLPH1, PAP7)	ER - Golgi, mitochondria, plasma membrane, cytosol [29]	Facilitates multiple protein interactions; Golgi-ER tether; Golgi scaffold protein; Vesicle trafficking; Sphingolipid transport; Mitochondrial cholesterol transport/steroid synthesis; Regulation of cellular iron uptake [[115], [116], [117], [118], [119], [120], [121], [122], [123], [124], [125], [126],^128^,[131], [132], [133], [134], [135]]	Host interaction protein for the replication of multiple members of the picornavirus family [19,22,[136], [137], [138], [139], [140]]
ACBD4	Peroxisomes (membrane) [7]	PO-ER tether; Capturing of acyl-CoA at the PO-ER interface [9,17]	Cardiac conduction (rodents) [185]
ACBD5 (synonyms: KIAA1996, ATG37 in yeast)	Peroxisomes (membrane) [7]	PO-ER tether; Modulation of PO motility and distribution; ER-PO lipid transfer; Capturing of CoA-VLCFA for PO β-oxidation [4,5,17]	ACBD5 deficiency [6,8] (OMIM 616618) Host interaction protein for Zika virus [21]
ACBD6	Cytosol, nucleus [155]	Regulation of acylation of lipids and proteins; Regulation of N-myristoyltransferase [19,119,158,159]	ACBD6 function exploited by *Chlamydia trachomatis* [19,160]
ACBD7	Soluble	Control of feeding behaviour and energy expenditure via the melanocortin system [99]	Obesity?
ACBD8 (synonyms: testis-specific endozepine-like peptide, ELP)	Soluble (pseudogene in humans) [37,38]	Testis-specific endozepine-like peptide [37,38]	

For details see 3, 4, 5, 6, 7 and Fig. 4. Note that isoforms are not included. ER, endoplasmic reticulum; PO, peroxisome.

a For the localization and function of plant ACBPs see [2].

**Table 2:** Association of ACBDs^a^ in protein complexes (findings from high-throughput approaches not included).

Protein complex	Complex function	Complex localization	Reference
**ACBD1**			
ACBD1-HNF-1α	Regulating transcription of HNF-1α target genes	Nucleus	[68]
ACBD1-CPTI	Acyl-CoA ester delivery to mitochondria	Mitochondria	[[73], [74], [75]]
ACBD1-CerS2/3(-ELOVL1)	Coordination of VLCFA ceramides (and esters) synthesis	ER	[82,83]
ACBD1-GABA receptor^c^	Modulating GABA signaling	Plasma membrane - extracellular	[84,87]

**ACBD2**			
Pex5-ACBD2-Tomm20	Tethering between peroxisomes and mitochondria	Peroxisomes, mitochondria	[10]

**ACBD3**			
ACBD3-giantin	Golgi structure maintenance	Golgi cisternae	[115]
GRASP55-Golgin45-ACBD3-TBC1D22	Golgi structure maintenance and membrane trafficking	Medial Golgi cisternae	[116]
ACBD3-FAPP2^b^	Glycosphingolipid metabolism	Trans-Golgi network	[120]
Giantin-ACBD3-PPM1L-VAPA-CERT	Glycosphingolipid metabolism	ER-Golgi contact sites	[18]
ACBD3-golgin160^b^	Regulation of apoptosis	Golgi	[122]
PI4KB-ACBD3-giantin	Phosphatidylinositol phosphorylation to control Golgi structure/function	Golgi	[125]
ACBD3-SREBP1^b^	Regulation of de novo fatty acid synthesis	Golgi	[52]
ACBD3-Numb	Cell fate determination	Cytosol	[134,135]
StAR-PKARIα-ACBD3-TSPO/PBR-VDAC1	Cholesterol uptake for steroidogenesis	Mitochondria	[128,186]
DMT1-ACBD3-DEXRAS	Cellular iron uptake	Plasma membrane of neurons and brush border cells	[131,132]
Picornavirus 3A protein-ACBD3-PI4KB	Viral genome replication	Viral replication organelle	[137,138]
Picornavirus 2B, 2BC, 2C, 3A, 3AB proteins-ACBD3-OSBP-SAC1-VAPA/B	Cholesterol transport from Golgi to viral replication organelle	Golgi-Aichi virus replication at organelle contact sites	[139,140]

**ACBD4**			
ACBD4-VAPB	Tethering between peroxisomes and ER	Peroxisomes, ER	[9]

**ACBD5**			
ACBD5-VAPA/B	Tethering between peroxisomes and ER	Peroxisomes, ER	[4,5]
ACBD5-VAPB-ACSL1	Coordination of fatty acid metabolism between ER and peroxisomes?	Peroxisomes, ER	[152]

**ACBD6**			
ACBD6-NMT2	Stimulation of protein N-myristoylation by providing substrates to the enzyme	Cytosolic side of cellular membranes	[159]
ACBD6-LPLATs^c^	Preventing LPLAT inhibition by access acyl-CoAs	Cytosolic side of cellular membranes	[119]

a Currently no specific protein interactions for ACBD7 and ACBD8 have been described.

b Likely, the protein complex includes a further Golgi protein to anchor ACBD3 at the Golgi membrane.

c Interaction at functional level; regulation via a direct protein interaction remains to be determined.

### 2.2 Small ACBD containing proteins

**Fig. 1:**
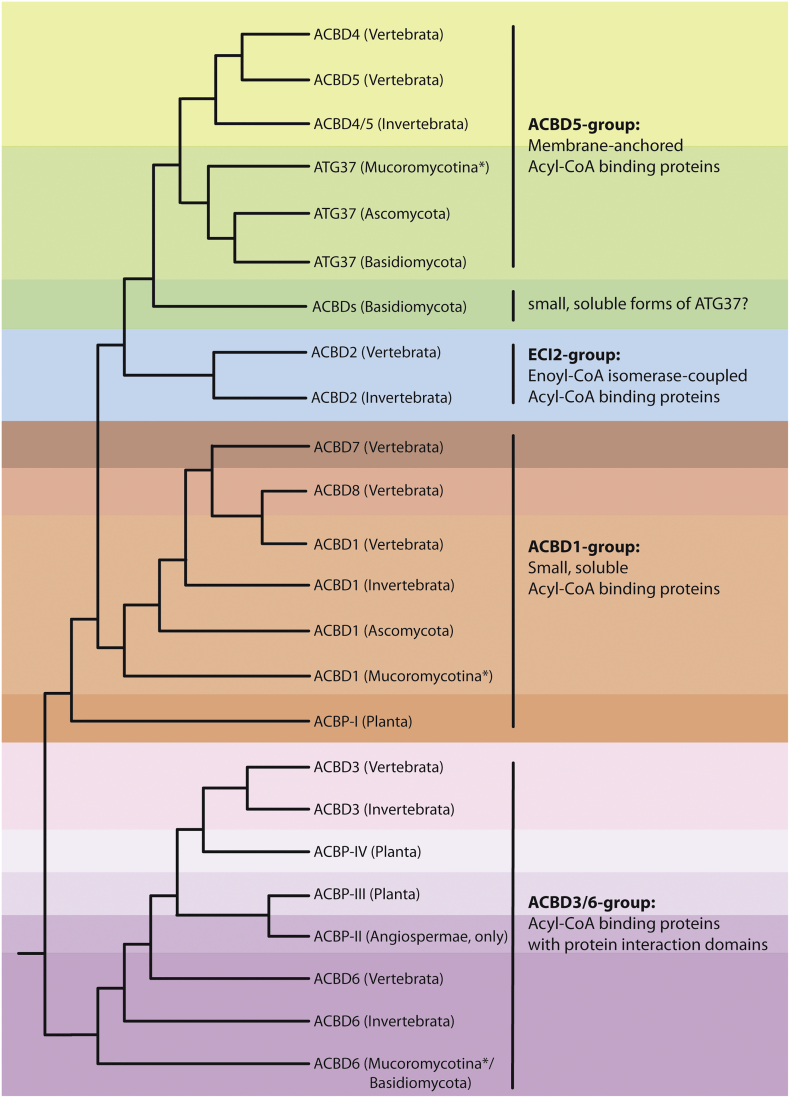
Evolution of Acyl-CoA binding domain containing proteins (ACBDs) among metazoans. The scheme summarizes data from a phylogenetic reconstruction including 449 sequences from animals, fungi and plants (for details see Fig. S1). After alignment by the ClustalW 2.0 algorithm included in the Seaview software package and manual correction of misaligned sequences, phylogenetic reconstructions were performed with PhyML3.0 using the aLRT algorithm for branch support, BioNJ for tree topology optimization, 4 rate categories for RHAS (Rate Heterogeneity Among Sites) modelling and including NNI and SPR tree searching operations. Furthermore, we used Marcoil1.0 online prediction tool [181] to screen the sequences for coiled-coil motifs and TMHMM2.0 and TMPred for TMD prediction [182,183]. Colours highlight four major categories of ACBDs – the C-terminally membrane-anchored ACBD4/5 (green), the enoyl-CoA isomerase containing ACBD2 (blue), the small soluble ACBD1 (red) and the extended forms with protein interaction domains ACBD3/6 and extended plant forms (purple; ACBP class II–IV). Taxonomic categories shown name the minimum organism group in which a distinct protein subfamily was detected. E.g. while ACBD2 was only found in vertebrate and invertebrate animals, ACBD4/5 occur in both animals and fungi, more specifically in basidiomycota and several early branching fungal divisions of incertae sedis (*symbolized with Mucoromycota in the figure). By contrast, the ankyrin repeat-containing ACBD6/ACBP II is distributed among all metazoans indicating its early evolution from the ACBD1 form. Evolution of Acyl-CoA binding domain containing proteins (ACBDs) among metazoans. The scheme summarizes data from a phylogenetic reconstruction including 449 sequences from animals, fungi and plants (for details see Fig. S1). After alignment by the ClustalW 2.0 algorithm included in the Seaview software package and manual correction of misaligned sequences, phylogenetic reconstructions were performed with PhyML3.0 using the aLRT algorithm for branch support, BioNJ for tree topology optimization, 4 rate categories for RHAS (Rate Heterogeneity Among Sites) modelling and including NNI and SPR tree searching operations. Furthermore, we used Marcoil1.0 online prediction tool [181] to screen the sequences for coiled-coil motifs and TMHMM2.0 and TMPred for TMD prediction [182,183]. Colours highlight four major categories of ACBDs – the C-terminally membrane-anchored ACBD4/5 (green), the enoyl-CoA isomerase containing ACBD2 (blue), the small soluble ACBD1 (red) and the extended forms with protein interaction domains ACBD3/6 and extended plant forms (purple; ACBP class II–IV). Taxonomic categories shown name the minimum organism group in which a distinct protein subfamily was detected. E.g. while ACBD2 was only found in vertebrate and invertebrate animals, ACBD4/5 occur in both animals and fungi, more specifically in basidiomycota and several early branching fungal divisions of incertae sedis (*symbolized with Mucoromycota in the figure). By contrast, the ankyrin repeat-containing ACBD6/ACBP II is distributed among all metazoans indicating its early evolution from the ACBD1 form.

In order to recapitulate the structural and functional evolution from the small ACBDs to the extended ACBD proteins we performed a phylogenetic analysis of 449 complete, ACBD-containing sequences from 116 animal, plant and fungal species extracted from GenBank and the JGI Genome Portal (Figs. 1, S1). According to our analysis, most species possess at least one small (non-membrane anchored/soluble) ACBD with a mass of approximately 10 kDa, and a tendency to accumulate several small forms in the genome can be observed throughout all organismal classes (Fig. 1). The origin of the small forms in plants, where the small ACBDs are termed ACBP class I, and in fungi could not be clearly resolved. They may have arisen polyphyletically by species-specific gene duplications or domain loss from extended forms. However, vertebrates developed two small, more closely related forms, ACBD1 and ACBD7, from a single invertebrate precursor. A third group of mammalian, small ACBD sequences form an individual branch on the cladogram (Figs. 1, S1). This third small ACBD form (in the following termed ACBD8) was originally identified as testis-specific endozepine-like peptide (ELP) in mice [37]. In humans the protein was not found to be expressed but still exists as pseudogene in the genome [38]. As the limited number of vertebrate species used in our initial phylogenetic analysis did not allow us to discriminate whether these three small ACBD variants descended from a single ancestor, we refined our analysis for the small forms. To this end, we restricted cladogram reconstruction to 246 sequences from 77 metazoan species using the nearest neighbor ACBD2 cluster from our initial analysis as an outgroup (Fig. S2). According to the phylogenetic tree, there is evidence that the ancestor ACBD was duplicated into ACBD1 and ACBD7 during early vertebrate evolution, followed by a second gene duplication into ACBD8. Interestingly, proteins related to ACBD8 exist in the more primitive reptilian groups of crocodiles and turtles, suggesting that this event occurred already at an earlier stage of amniotic evolution. Like in humans, predicted potential full-length ACBD8 protein sequences could not be retrieved from insectivoras, monotremata (mammalia), squamata (reptilia) and birds arguing for multiple evolutionary losses of a protein with a specialized or tissue-confined function.

**Fig. 2:**
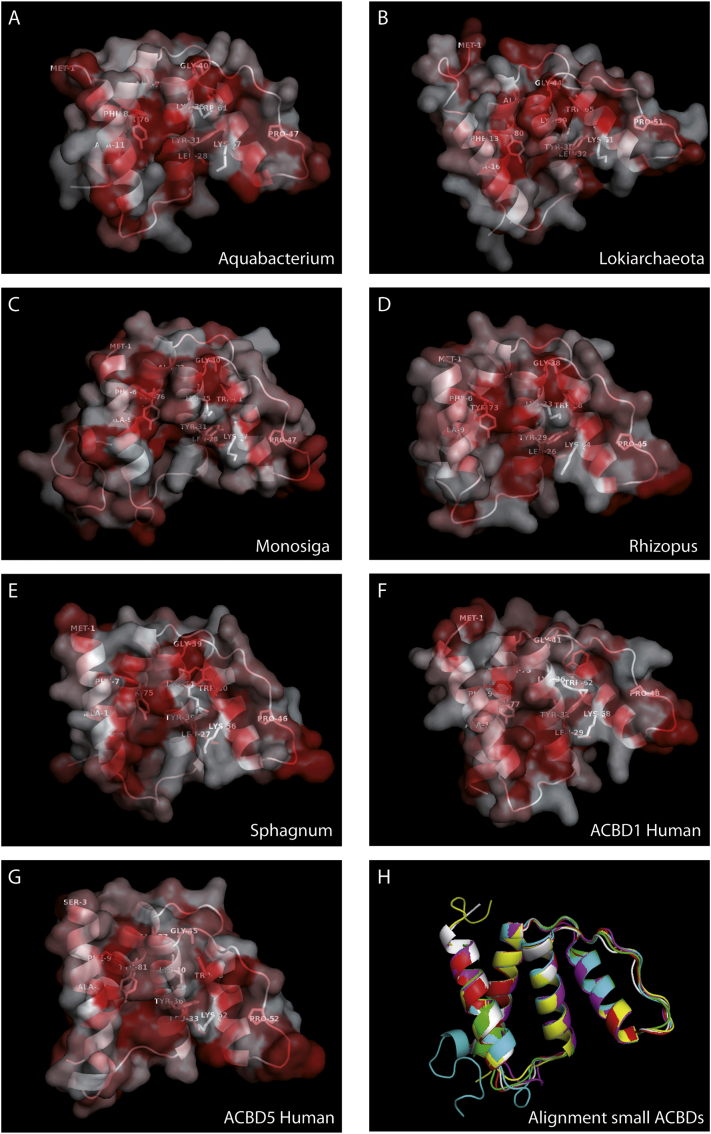
3D predictions of selected acyl-CoA binding proteins/domains. The predictions were accomplished with Phyre^2^ using entire cDNA sequences from ACBD1 of *Homo sapiens* (F) and homologous small ACBDs from the β-proteobacterium *Aquabacterium parvum beta* (A), an hitherto undefined ACBD sequence (TFG05618) of Lokiarchaeota origin (B), the choanoflagellate *Monosiga brevicollis* (C), the Mucoromycota fungus *Rhizopus oryzae* (D), the moss *Sphagnum fallax* (E); for comparison the ACB domain of human ACBD5 was added subjected to 3D prediction (G). Hydrophobic regions in the protein surface structure are highlighted in red and residues conserved among all species are shown. (H) Tertiary structure reconstructions from (A)–(F) are additionally shown as sequence overlay in ribbon cartoon format to illustrate the significant structural homology in the distantly related species (depicted in different colours). Conversion of the PDB files into graphical illustrations was performed with PyMOL 2.3.2.

Unexpectedly, a significant number of small ACBD-like sequences were detected among prokaryotes during the searches (distributed in 108 bacterial genera). Thus, to unravel if the small ACBDs might have appeared already before the origin of eukaryotes, Blast-searches among all major prokaryote branches were performed. Indeed, numerous ACBD-like sequences with significant similarities could be retrieved (see Fig. S3 for sequence comparison) and were used for a phylogenetic reconstruction. As no extended forms were found among prokaryotes, the small forms from eukaryotes were routed with the prokaryotic sequences using the animal ACBD2 cluster as outgroup. Remarkably, the ACBDs from the major prokaryotic organism groups of archaea, β-, γ-, δ-proteobacteria and bacteroidetes clustered into individual distinct branches in parallel to the eukaryotic clades, while the rare sequences found in α-proteobacteria did not align in a single branch but distributed as orphan sequences among the branches of the tree (Fig. S4). To further evaluate if the prokaryotic sequences could theoretically assemble into an ACBD structure, Phyre^2^ was used to predict potential tertiary protein conformations of selected prokaryotic and eukaryotic ACBD sequences [39]. As depicted in Fig. 2, all sequence models can theoretically form the 4 helix bundle structure typical for ACB domains. Remarkably, hydrophilic and hydrophobic patches on the protein surface like the hydrophobic acyl chain binding cleft between A2 and A3 or Phe5 and Tyr73 for adenine binding [36] are also structurally conserved in all 3D predictions. As the possibility of numerous horizontal gene transfers in prokaryotes does not allow a simple cladistics reconstruction of bacterial protein evolution, it is not possible to speculate on the phylogeny of ACBDs in bacteria [40]. The occurrence of structurally homoform ACBD sequences in many prokaryote groups suggests that a common ancestor of the small ACBDs may have already arisen before appearance of the first eukaryotes. However, it cannot be excluded that multiple individual lateral gene transfers may have distributed the small ACBD sequences among the different prokaryote groups.

With respect to their intracellular localization, the small ACBDs appear to reside in the cytosol but might associate temporarily with specific subcellular structures or may even be secreted [1,2]. Thus, their intracellular location may reflect the function of the small ACBDs as intracellular lipid carriers or modulators but direct proof under in vivo conditions is currently lacking.

### 2.3 Extended ACBD containing proteins

**Fig. 3:**
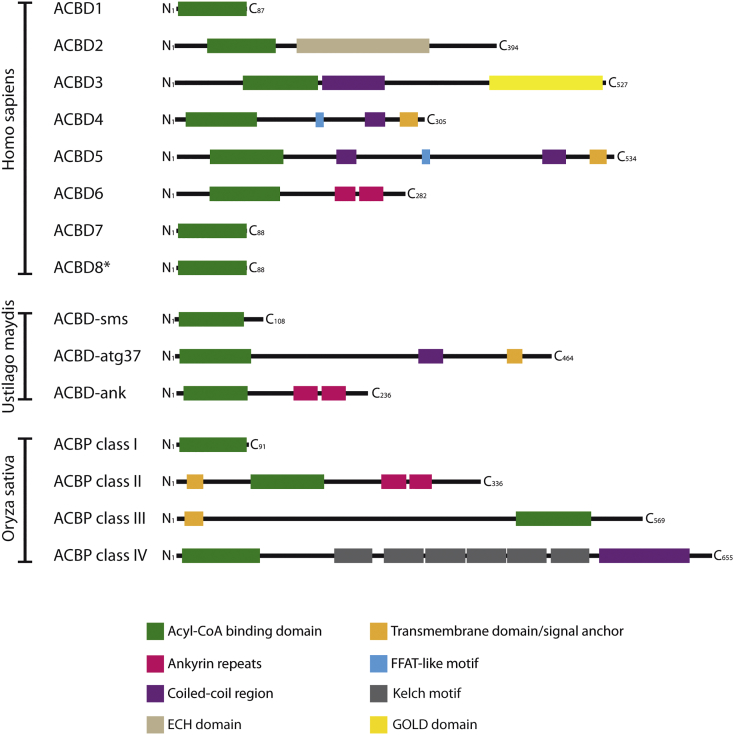
Domain structures of animal, fungal and plant acyl-CoA binding proteins. The schemes represent ACBDs from *Homo sapiens* (Mammalia, Animalia), *Ustilago maydis* (Basidiomycota, Fungi) and *Oryza sativa* (Angiospermae, Planta); sequence and domain lengths as well as position are proportional to the original protein structure. For some ACBDs, several isoforms have been reported or predicted. In such cases, only the currently best characterized isoform is presented. Note the structural similarity between the mammalian, fungal and plant ankyrin repeat-containing ACBDs, which suggests an early evolution already found in the LECA. ECH, enoyl-CoA hydratase domain; FFAT, two phenylalanines in an acidic tract motif.

Based on the structure and motifs of the different accessory sequences in the extended ACBDs and the phylogenetic reconstruction, animal ACBD proteins can be classified into three additional principal protein groups: the tail-anchored ACBD proteins with a C-terminal α-helical membrane anchor, the enzyme-coupled ACBDs and the extended, soluble forms with C-terminal interaction domains (Fig. 1, Fig. 3). In the heterogeneous group of extended, soluble ACBD forms, ankyrin-repeat motifs, which are positioned downstream of the ACB domain, are frequently found and also appear in the choanoflagellate *Monosiga*, the amoebozoan *Dictyostelium* and in planta but also among fungi in *Basidiomycota* and some of the early branching groups like e.g. *Mucoromycotina*. Unfortunately, the low sequence similarity and restricted availability of genomes in the more primitive plant, fungal, and animal classes prevented a full resolution of the evolutionary radiation of these extended ACBD subfamilies (Fig. S1). Nevertheless, based on the structural conservation among all different phyla it can be assumed that the ankyrin-repeat containing ACBDs, which in animals are represented by ACBD6, may have already developed in the last eukaryotic common ancestor (LECA).

Plant ACBPs have been grouped into the ACBP classes I–IV, which consist of the ACBP class I (10 kDa form) and the larger, extended forms from class II–IV [41]. For easier discrimination, we will hence term metazoan and fungal ACB-domain containing proteins according to the ACBD nomenclature and the plant forms as ACBP class I–IV. In plants, ankyrin-repeat containing ACBD proteins are classified as the ACBP class II [2], which clusters in proximity to the ACBD6 group in the phylogenetic reconstruction (Fig. 1). Compared to the animal and fungal ankyrin-repeat containing ACBD6 proteins, the plant ACBP IIs are larger proteins with an additional N-terminal extension bearing a transmembrane helix (TMD) which may also act as a signal sequence for subcellular targeting [41] (Fig. 3). The plant ACBP class III is closely related to class II, also exhibiting an N-terminal TMD and an ACB domain, however, it lacks the C-terminal ankyrin-repeat motifs (Fig. 3). Thus, if the fusion of the ankyrin and ACB domains did not occur by convergent evolution in metazoans, plants and fungi, plant ACBP class III may have evolved from class II by secondary loss of this motif. The important structural difference of the additional TMD in plants is further demonstrated by the different subcellular localization of ankyrin-repeat containing ACBDs in animals and plants. While under normal conditions ACBD6 is a soluble protein locating to the cytosol and nucleus [19], plant ACBP class II and class III proteins are membrane-anchored and were found at the ER, Golgi and plasma membrane [2]. Functionally, ankyrin-repeats are typical protein structures facilitating protein interactions, however, they were also recently found to mediate lipid binding at subcellular membranes [42]. Thus, ankyrin-repeats of ACBP II/ACBD6 proteins might mediate temporary contacts with intracellular membranes, either directly or via interaction with a transmembrane protein.

In addition to the ACBPs with C-terminal ankyrin-repeats, C-terminal Kelch motifs and GOLD (Golgi dynamics) domains can be found in plants and metazoans including choanoflagellates, respectively (ACBP IV, ACBD3) (Fig. 3). Both groups may have independently evolved; according to sequence similarities and routing in the ACBD3 branch of the cladogram, however, they may share a common extended ACBD ancestor protein (Figs. 1, S1). ACBD3 is structurally related to ACBD6, but instead of ankyrin-repeats the protein possesses a β-strand-rich GOLD domain in its C-terminal region, which can facilitate interactions with other proteins and may be used to stabilize peripheral membrane proteins at intracellular membranes [43] (Fig. 3). In addition, ACBD3 exhibits a highly conserved coiled-coil region located in the middle of the sequence, which might act as another protein interaction domain. Unlike ACBD6, ACBD3 can only be found in animals and choanoflagellates and thus appears to have evolved comparatively late. ACBD3 localizes to the ER/Golgi intermediate compartment facilitating important functions in vesicle trafficking, as well as at mitochondria [46]. Since GOLD domains can be found in several lipid binding or lipid transport proteins such as the tocopherol associated proteins, the FYVE finger domain containing proteins or the oxysterol-binding proteins [43,44], GOLD domain containing proteins such as ACBD3 might regularly assist in lipid/vesicle transfer at intracellular membranes. Kelch repeats of the plant ACBP class IV, which form a β-propeller protein domain fold, are also typical structures mediating protein interactions [45]. Like ACBD3, the plant class IV ACBPs possess additional conserved coiled-coil motifs. However, as those are located at the very most C-terminal part of the proteins after the Kelch repeats, this seems to be a convergent evolution event combining two protein interaction domains (Fig. 3). Most Kelch repeat containing ACBPs seem to localize to the cytosol, where they might temporarily associate with various intracellular membrane structures; but ACBP6 of the Asian rice *Oryza sativa* was recently found to reside at peroxisomes where it may cooperate in peroxisomal fatty acid β-oxidation [46].

In addition to the ACBD3/6 branch, our phylogenetic reconstruction shows two further clusters of extended ACBDs, which are restricted to fungi and metazoans (ACBD4/5) or only metazoans (ACBD2). ACBD5, which in fungi was termed Atg37 [47], consists of an N-terminal ACB domain, a C-terminal TMD and three structures suited for mediating protein interactions: a FFAT (two phenylalanines (**FF**) in an **a**cidic **t**ract) and two coiled coil motifs in the intermediate part of the protein (Fig. 3). ACBD4 is a structurally and phylogenetically closely related protein with the same domain arrangement (Fig. 3). However, ACBD4s are significantly more compact proteins which appear to have evolved from ACBD5 by a later gene duplication, which only occurred in vertebrates (Fig. 1). Using a FFAT motif prediction tool (with a cut-off FFAT score ≤2.5) [48], we checked for the presence of predicted FFAT motifs in ACBD4/5/Atg37 proteins used in our phylogenetic analysis and identified a FFAT motif in almost all (89%) of the animal ACBD4/5 proteins. By contrast, the motif was not found in the majority of fungal species analysed but interestingly did appear in several cases (12.5%) (Table S1). To investigate this further, we extended our analysis to include 161 fungal species where we could identify a clear ACBD5 orthologue (Atg37) and also performed randomised sequence comparisons, applying a simple statistical analysis (as used in [49]) to control for false positives. Here, we observed an enrichment of FFAT motifs in the Ascomycota, which was most notable among the Dothideomycetes class (Table S1). Although this is not a completely comprehensive analysis and predictions of FFAT motifs can be problematic due to the variability of the motif [49], our analysis does suggest that not all ACBD5 class proteins contain a strong FFAT motif. The reason for the presence of a FFAT motif in certain fungal species and not others is not clear.

Atg37, ACBD4 as well as ACBD5 localize at peroxisomes, where they might perform complementary functions (see Section 6). However, ACBD4 isoforms lacking the TMD and protein interaction domains also exist [9] (Fig. 3). The FFAT motif is responsible for the function of ACBD4 and ACBD5 as ER tethering factors for peroxisomes by allowing interaction with ER VAP proteins (see Section 6). Since most fungal Atg37 proteins lack a FFAT motif, it is possible that Atg37 does not perform this function in the majority of fungi or it is replaced by a different interaction or tethering system [50].

ACBD2/ECI2 is probably the most uniquely extended ACBD protein, as its ACBD domain is fused to a lipid metabolizing enzymatic Δ3,Δ2-enoyl-CoA isomerase domain (Fig. 3). ACBD2, which also localizes to peroxisomes, is a relatively late evolutionary invention and is only found in metazoans. Enzymatic activity measurements with recombinant wild type and mutant forms lacking the ACB domain showed that the ACB domain is not absolutely essential for the isomerase reaction but that the ACB domain may have a role in effectively channeling acyl-CoAs to the isomerase domain [51]. At this time it cannot be excluded that the ACB domain may have an independent function not related to the enzymatic part of the protein (see Section 4).

**Fig. 4:**
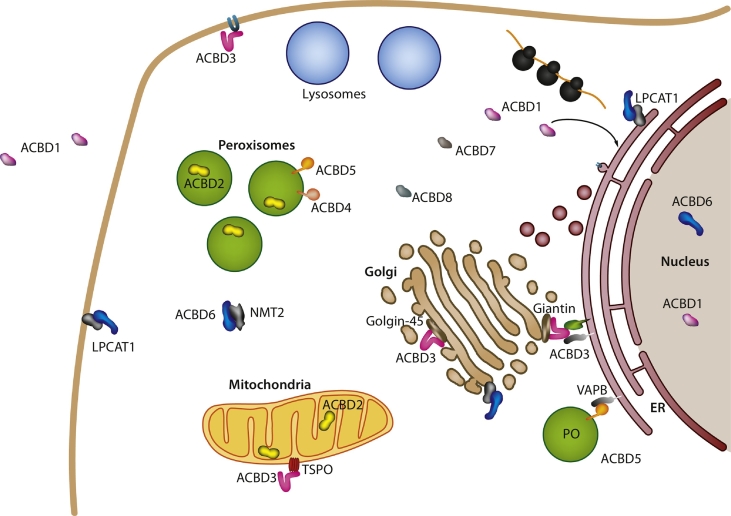
Schematic overview of the localization of ACBD proteins. ACBD, acyl-CoA binding domain containing protein (ACBD1–8); ER, endoplasmic reticulum; LPLAT, acyl-CoA:lysophospholipid acyltransferase; NMT2, N-myristoyltransferase 2; PO, peroxisome; TSPO, translocator protein for cholesterol; VAPB, vesicle-associated membrane protein (VAMP)–associated protein B. For details see 3, 4, 5, 6, 7 as well as Table 1, Table 2.

Overall, the combination of protein interaction domains with ACB domains might reflect a property of extended ACBD proteins in channeling lipid substrate to proteins involved in lipid metabolism or membrane transport (Table 1). As mentioned earlier, the role of ACBDs in lipid metabolism and transport is poorly defined in biological systems, but controlling/modifying the activity of other proteins or tethering membranes together may be an additional important function. We speculate that the protein interaction motifs and TMDs have been evolved to link the ACB domain-containing proteins to specific subcellular membranes, where they are exploited for organelle-specific functions, e.g. in acquiring lipids required for metabolic pathways or membrane lipid transport (Table 1, Table 2; Fig. 4). The increasing complexity of subcellular compartments during evolution appears to have led to a comparable evolution in ACBD proteins. While animals and fungi appear to share a principle ACBD inventory (ACBD1-, ACBD6-, ACBD5-like forms), plants likely accomplished a rather independent evolutionary radiation into structurally distinct forms, which may fulfill comparable but also distinct cellular functions.

This increasing functional complexity is reflected by the gain of diverse protein interaction domains in the extended ACBDs and the acquisition of functions, which could potentially be regulated by the acyl-CoA bound to the ACB [47,52] (or *vice versa*) or act independently (Fig. 3). Protein extension can also potentially change the cellular location of the ACBD protein (e.g. ACBD4/5; see Section 6), regulate the function of binding partners via protein-protein interaction (e.g. ACBD3; see Section 5), or add a new activity (e.g. ACBD2; see Section 4). In the following sections of this review, we will discuss the current knowledge of the various functions of animal and fungal ACBDs. More information on the structure, localization and function of the plant ACBPs can be found in several recent reviews and will thus not be discussed in detail [2,[53], [54], [55], [56]].

## 3 ACBD1 and ACBD7 proteins

ACBD1, originally known as acyl-CoA binding protein (ACBP) or diazepam-binding inhibitor (DBI), is the smallest member of the ACBD family, as it consists mainly of a soluble ACB domain (Fig. 3). This single-domain protein has been reported to bind saturated and unsaturated C14-C22 acyl-CoA esters with very high affinity and specificity [23,57,58], and has been associated with several lipid-related processes including the regulation of acyl-CoA transport to different acyl-CoA-utilizing enzymes [1] (Table 1, Table 2). ACBD1 is ubiquitously expressed [1,59], showing the highest expression in tissues with a significant fatty acid turnover such as liver, testis and adipose tissue [60,61]. Additionally, ACBD1 is highly expressed in the brain, including the hypothalamus, where it was initially identified as neuropeptide and named DBI [62,63].

Expression of ACBD1 can be activated by lipogenic as well as lipo-oxidative transcription factors, including sterol regulatory element-binding proteins (SREBPs), peroxisome proliferator-activated receptor α (PPARα) and PPARγ [64,65]. ACBD1 was suggested to function in a transcriptional feedback loop, since SREBPs and PPARγ were downregulated in ACBD1 overexpressing transgenic rats [66]. Additionally, ACBD1 could negatively affect PPAR activation, possibly by regulating the availability of PPAR-ligands, such as fatty acids, by its partly nuclear localization [67]. On the other hand, ACBD1 can enhance hepatocyte nuclear factor 4α (HNF-4α) transactivation suggesting an influence on the expression of HNF-4α target genes, including genes involved in lipid transport and metabolism [68]. It has been shown that ACBD1 and HNF-4α co-localize within the nucleus and in the perinuclear region in rat hepatoma and COS-7 cells, and interact directly in vitro. This all emphasises the important function of ACBD1 in the regulation of lipid metabolism. Studies indicate the involvement of ACBD1 in acyl-CoA transport and pool formation, modulating acyl-CoA-regulated enzymes, fatty acid synthesis and degradation, steroid and bile acid synthesis, and complex lipid synthesis (reviewed in [1,36,69]). Although ACBD1 function and peroxisome metabolism have not been linked directly, peroxisomes also contribute to several of these lipid-related processes [11,70].

ACBD1's regulation by and participation in both lipogenic and lipo-oxidative pathways may reflect its function as an acyl-CoA carrier, protecting acyl-CoA esters from hydrolysis [71], and transporting acyl-CoA esters to compartments for lipid synthesis as well as degradation. Although ACBD1 is primarily a cytosolic protein, the protein also localizes to specific compartments, including the ER, Golgi apparatus, mitochondria and nucleus [1,36] (Table 1, Table 2; Fig. 4). In this respect, ligand-binding may be required for the interaction with distinct subcellular compartments as shown for the localization to the ER/Golgi [72]. ACBD1 is involved in the degradation of fatty acids by transporting and donating acyl-CoA esters to mitochondria, likely by binding to the outer mitochondrial membrane protein carnitine palmitoyltransferase I (CPTI), the rate limiting enzyme in mitochondrial β-oxidation of long-chain fatty acids (LCFAs, C12-C20) [[73], [74], [75]]. Interestingly, ACBD1 has been reported to bind with high affinity to acyl-CoA esters with a chain length of 22 carbons [23], representing very long-chain fatty acids (VLCFAs, ≥C22). VLCFAs are not a substrate for CPTI and can only be oxidized in peroxisomes, not in mitochondria [11]. This leads to the possibility that ACBD1 may transport C22 acyl-CoA esters to peroxisomes for β-oxidation. A peroxisomal localization of ACBD1 has not been reported, but interestingly in a genome-wide analysis in mouse ACBD1 mRNA was found to be localized to peroxisomes [76], which might suggest an alternative mechanism to control targeting of the protein, as has been suggested for several peroxisomal proteins in *Saccharomyces cerevisiae* [77]. Interestingly, an interaction between peroxisomal acyl-coenzyme A oxidase 1 (ACOX1) and ACBD1 has been found in a high-throughput yeast-two-hybrid screening of *Drosophila melanogaster* [78]. ACBD1 has also been associated with ACOX1 in a genome-wide transcript profiling study in which ACOX1 gene expression was downregulated following ACBD1 knockdown in a human liver cancer cell line [79]. Thus, ACBD1 delivers LCFAs to mitochondria for degradation; whether ACBD1 also localizes to peroxisomes to deliver VLCFAs for degradation remains to be elucidated.

Furthermore, ACBD1 may regulate the synthesis of VLCFAs. Depletion of the ACBD1 orthologue in *S. cerevisiae* resulted in increased levels of C18:0 acyl-CoA esters and simultaneously in a strong reduction in C26:0 levels [80]. The study indicates that the transport of LCFAs to the chain elongation complex at the ER-membrane is impaired, so that these shorter-chain fatty acids cannot be elongated to VLCFAs. There is some evidence to suggest that ACBD1 may also play a role in the synthesis of VLCFAs in mammals. Increased C18:0-CoA levels have been reported in the brain of ACBD1 deficient mice [63]. Since the oxidation of C18:0 was not altered and the expression of genes coding for de novo fatty acid synthesis enzymes, including ACC1 and FAS, were downregulated in ACBD1 depleted astrocytes, the increased levels of C18:0 may be the result of decreased LCFA elongation [63]. Another study found a decrease in the VLCFA content in the stratum corneum of ACBD1 deficient mice, although the elongation activity in the whole epidermis was not altered [81]. A role of ACBD1 in the elongation of LCFAs has also been suggested in the synthesis of VLCFA ceramides. Both ceramide synthase 2 (CerS2) and CerS3 are activated by ligand-bound ACBD1 [82]. As both ceramide synthases utilize VLCFAs, it is likely that ACBD1 channels C22-C26-CoAs to CerS2 and CerS3. Gel shift assays show that ACBD1 has the ability to bind C24- and C26-CoA, however, exact affinities have yet to be determined. As CerS2 interacts with both ACBD1 [82] and VLCFA elongase ELOVL1 [83], the proteins may form a complex coordinating synthesis of VLCFA esters and ceramides. If ACBD1 does play a role in the synthesis of VLCFAs, it may indirectly affect the peroxisomal VLCFA β-oxidation. In addition, C24:0/C26:0-CoA has to be channeled from the ER to the peroxisomal membrane, but the proteins responsible for this remain unknown (see also Section 6). Therefore, ACBD1 might be a candidate. Overall, ACBD1 plays important roles in the transport and donation of fatty acids for both degradation and synthesis.

Besides its function as acyl-CoA carrier, ACBD1 was identified as a secreted brain neuropeptide, modulating the gamma-aminobutyric acid (GABA) type-A receptor, the main inhibitory neurotransmitter receptor located in brain synapses, and named diazepam-binding inhibitor (DBI), as it can inhibit the binding of the anxiolytic drug diazepam to the GABA receptor [84]. This could not be reproduced by others and a direct interaction between ACBD1 and the GABA receptor has never been shown [23,85], but multiple studies support modulation of GABA transmission by ACBD1; ACBD1 and its proteolytic peptide fragments, including octadecaneuropeptide (ODN) produced by astroglial cells, regulate neuronal activities by stimulating neurosteroid biosynthesis [86] and neurogenesis [87] via GABA receptor modulation. ACBD1 can also act as a neuroprotective factor by activation of a metabotropic receptor [88]. Moreover, ACBD1 functions not only as synaptic neuropeptide (endozepine), but also influences brain physiology via its endogenous expression by regulating LCFA metabolism in astroglial cells [63]. Breitling [89] hypothesized that ACBD1 may contribute to the pathogenesis of peroxisomal disorders by dysregulating the GABAergic system, causing severe neurological defects. The author suggests that peroxisomal dysfunction leads to the accumulation of lipid metabolites e.g. acyl-CoA, causing downregulation of ACBD1 and hence in its function as a diazepam-binding inhibitor an over-activation in GABA signaling in neurons. However, the paper does not show a direct link between ACBD1 and peroxisomal disorders. The possible role of ACBD1 in the pathogenesis of peroxisomal disorders relies largely on the identification of ACBD1 in a microarray database search in which proteins were screened for three relative general criteria: its involvement in lipid metabolism, brain or neuronal function, and signaling or transcription [89], though the study proposes a different perspective on a possible ACBD1-peroxisome connection.

Studies on rodent behaviour associate ACBD1 with social interest [90] and learning [91,92], and there are also links between ACBD1 and anxiety, since intracerebroventricular administration of ODN induces anxiogenic effects in rodents [93]. Additionally, ACBD1 polymorphisms have been associated with anxiety disorders with panic attacks in humans [94]. However, anxiety-like behaviour was not affected in ACBD1 knock-out mice [95] as well as transgenic mice overexpressing ACBD1 [91]. It was recently reported that astroglial ACBD1 deficiency promoted diet-induced obesity in mice [96]. This could be prevented by viral rescue of ACBD1 in astrocytes in the nucleus arcuatus (ARC), a hypothalamic nucleus that regulates energy homeostasis. In line with this, it was very recently reported that short-term starvation of cultured cells or mice caused the autophagy-dependent secretion of ACBD1 [97]. In mice, injection of ACBD1 protein inhibited autophagy, induced lipogenesis, reduced glycemia, and stimulated appetite as well as weight gain. In contrast, ACBD1 neutralization enhanced autophagy, stimulated fatty acid oxidation, inhibited appetite, reduced weight gain in the context of a high-fat diet or leptin deficiency, and accelerated weight loss [97]. The ACBD1 orthologues in *S. cerevisiae* and *Caenorhabditis elegans* have also been associated with food intake, indicating a conserved role for ACBD1 in appetite [98]. In humans, ACBD1 levels were observed to be abnormally low in persons with anorexia nervosa but excessively high in obese patients. Neutralization of ACBD1 might therefore constitute a strategy for treating obesity, whereas its administration may be beneficial in patients with anorexia [97]. Interestingly, ACBD1 paralog ACBD7 was also recently reported to influence feeding behaviour in mice [99]. ACBD7 and its processing product nonadecaneuropeptide (NDN) are produced by GABAergic and pro-opiomelanocortin (POMC) neuronal cells in the ARC. Intracerebroventricular administration of NDN inhibited food intake and enhanced energy expenditure in fasted mice. Both ACBD1 and ACBD7 seem to control feeding behaviour and energy expenditure via the melanocortin system [96,99] (Table 1). Peroxisomes have also been linked to this regulatory circuit; peroxisome proliferation-associated regulation of ROS levels in POMC neurons affected feeding behaviour in mice [100].

## 4 ACBD2/ECI2 protein

ACBD2 (ECI2, PECI) is a peroxisomal Δ3,Δ2-enoyl-CoA isomerase, which represents a fusion of a larger C-terminal, enzymatic enoyl-CoA isomerase part to an N-terminal ACB domain, which is structurally similar to ACBD1 [51] (Fig. 3). The enzyme was first identified as an enoyl-CoA isomerase according to its sequence similarity to its yeast orthologue ECI1 and found to be localized to peroxisomes, in which it is imported into the organelle matrix, in human and mice [101,102] (Table 1, Table 2; Fig. 4). ACBD2's ACB domain was identified according to its sequence similarity to ACBD1 [103]. A subsequent study on the rat enzyme further showed the bimodal distribution of ACBD2/ECI2 to peroxisomes as well as mitochondria [104], while its yeast homolog ECI2/DCI1 is solely peroxisomal [105,106]. Indeed, there are two cDNA isoforms in mammals; both exhibit a C-terminal peroxisomal targeting sequence 1 (PTS1), however, the longer form possesses an additional mitochondrial targeting sequence (MTS) at its N-terminus (aa 1–35). According to its enoyl-CoA isomerase domain, the protein is primarily required as an auxiliary enzyme for the β-oxidation of unsaturated long- and VLCFAs, specifically to convert Δ3 into Δ2 enoyl-CoAs which can be further degraded by the straight-chain peroxisomal β-oxidation pathway [107] (Table 1). However, while the enzymatic function of the protein is well established, the role of its ACB domain remains unresolved. Supposedly, the ACB domain works as an acceptor for acyl-CoAs in the peroxisome matrix in order to efficiently exchange substrates or products with the protein's enzymatic domain. In this regard, a recent study showed that a truncated version of ECI2 without the ACB domain was still functional but exhibited less enzymatic activity than the complete protein [51]. Interestingly, as described above, the ACB domain can only be found in animal species, whereas the homologous plant and fungal ECI2 proteins lack this N-terminal sequence extension. Moreover, in murine species a closely related peroxisomal protein, ECI3, has been described which also lacks the ACB domain found in ACBD2/ECI2 [108]. Thus, the ACB domain is not obligatory for ACBD2/ECI2's enzymatic activity but may increase the effectivity of the enzymatic domain by channeling acyl-CoAs for their subsequent metabolism. Alternatively, the ACB domain of ACBD2 might fulfill an independent, hitherto undetected function for example as an intraorganellar acyl-CoA reservoir/transporter (Table 1). Recently, ACBD2/ECI2 was found to be significantly up-regulated in prostate cancer [109]. Further peroxisomal enzymes which are consistently induced in malignant prostate tumors are AMACR and ACOX3 [110,111], which both contribute to the catabolism of branched-chain fatty acids [112,113]. However, there seems to be no general up-regulation of peroxisomal lipid metabolism [111]. While ACBD2/ECI2's role in the metabolism of unsaturated fatty acids does not explain such a concerted up-regulation with enzymes from the branched-chain fatty acid catabolism, these findings indicate that the protein's ACB domain might contribute to other peroxisome pathways.

A recent study reported that ACBD2/ECI2 might act as a tether between peroxisomes and mitochondria in mammalian cells [10] (Table 1, Table 2). As a mechanistic explanation the authors suggested that ACBD2/ECI2 interacts in parallel with the mitochondrial outer membrane import protein TOMM20 via its N-terminal MTS and with the cytosolic peroxisomal import receptor PEX5 via the C-terminal PTS1 sequence. Thereby, the protein would enter and block both import machineries and thus tether mitochondria and peroxisomes to each other [10]. Thus, in addition to ACBD4 and ACBD5 (see Section 6), ACBD2/ECI2 would be the third peroxisomal ACBD protein with an organelle tethering function, however, facilitating this function by a completely different and unique mechanism (see Section 6 for comparison with ACBD4/5). Moreover, protein import into peroxisomes (folded protein) and mitochondria (unfolded sequence) differs substantially, adding further complexity to the formation of a potential tethering complex. Remarkably, ACBD2/ECI2 is not the only protein with a combined N-terminal MTS and a C-terminal PTS1: several peroxisomal matrix proteins like e.g. peroxiredoxin 5, glutathione-S-transferase kappa or the Δ(3,5)-Δ(2,4)-dienoyl-CoA isomerase ECH1 are expressed as sequences or isoform sequences which have such opposing double-targeting signals (see [114]). Therefore, future studies are required to investigate if such proteins generally provide interactions between peroxisomes and mitochondria. Furthermore, it is indispensable to evaluate the existence of TOM20-ACBD2/ECI2-PEX5-PEX14 multimeric complexes at the biochemical level and to provide triple staining, showing in parallel mitochondrial and peroxisomal markers and signals for ACBD2/ECI2 in order to verify that a significant proportion of the latter protein is localized directly at the contact sites between both organelles.

## 5 ACBD3 protein

ACBD3 was formerly named GCP60 (Golgi complex-associated protein of 60 kDa), Golgi complex-associated protein 1 (GOCAP1), PAP7 (cAMP-dependent protein kinase and peripheral-type benzodiazepine receptor associated protein 7) and GOLPH1 (Golgi phosphoprotein 1) [29]. The multiple names reflect the diverse functions of ACBD3 and its probably most prominent biophysical quality – the interaction with numerous, different proteins (Table 1, Table 2). ACBD3 consists of an N-terminal ACB domain followed by a charged amino acid region, a glutamine-rich coiled coil region (Q-domain) and a C-terminal GOLD domain (Fig. 3). Protein interactions are facilitated by binding to the Q-region but more frequently by the GOLD domain. Proteins with C-terminal GOLD domains regularly contain N-terminal lipid binding domains as found in ACBD3 and may function as double-headed adaptors connecting a protein and a lipid entity [43]. Thereby, these proteins may act as cargo loading proteins attaching soluble proteins to a specific membrane or by transmitting lipid species from the cytosol to a membrane-anchored interaction partner.

According to its interaction with several members of the golgin-family, ACBD3's GOLD domain appears to facilitate its localization to the Golgi compartment (Table 1, Table 2; Fig. 4). In this respect, ACBD3 is potentially part of a protein scaffold interacting with the golgins giantin [115] and golgin-45 [116] at either the Golgi rim or in between Golgi cisternae, respectively. In line with the differential localization of the golgin-45 and giantin interaction, ACBD3 appears to participate in two distinct Golgi multiprotein complexes [117] Table 2:. This scaffold-forming quality of ACBD3 is supported by the finding that ACBD3 can self-interact via its GOLD domain [118], assembling oligomers of high molecular weight [119]. This structure-giving function of ACBD3 may be essential for maintaining Golgi integrity as ACBD3 knockdown resulted in Golgi fragmentation [120]. Additionally, the protein's GOLD domain is able to recruit further proteins to the Golgi membrane. ACBD3 was reported to localize the sphingolipid transfer protein FAPP2 to the Golgi, where it is required for transmitting glycosylceramides to the trans-Golgi compartment in order to be metabolized into complex glycosphingolipids [120]. Also associated with sphingolipid transport is ACBD3's interaction with the ER-resident phosphatase PPM1L [18]. Two ACBD3 molecules were proposed to tether ER and Golgi membranes via parallel interaction with PPM1L and giantin. In cooperation with the ER-tethering protein VAPA, PPM1L can dephosphorylate and modulate the activity of the ceramide transport protein CERT at the contact site thereby regulating ceramide transport between the ER and Golgi [18].

ACBD3 can further interact with Golgi proteins via its coiled-coil Q-region. Golgin-160 is a caspase-sensitive protein at the Golgi membrane, which after cleavage exposes a cryptic nuclear targeting signal inhibiting apoptosis upon nuclear translocation [121]. ACBD3 retains golgin-160 cleavage fragments at the Golgi surface, thereby preventing their nuclear translocation [122,123].

Phosphatidylinositol 4-phosphate (PI4P) is an essential Golgi-enriched membrane lipid involved in cell signaling and membrane lipid transport [124]. Thus, for local PI4P synthesis, PI4 kinases (PI4K) have to localize at the Golgi. While type II PI4Ks are palmitoyl-anchored, type III PI4Ks like PI4KB are cytosolic and have to be recruited by interaction with membrane proteins. PI4KB is localized to the Golgi membrane via interaction with ACBD3's Q-domain in parallel enhancing its catalytic activity [125]. The putative Rab33 GTPase-activating proteins (GAP) TBC1D22A and B were also reported to interact with ACBD3 via its Q-domain [118]. In this respect, facilitating multiple protein interactions via the protein's different protein-binding domains (Q-domain for TBC1D22, GOLD-domain for golgin45) allows the formation of large protein oligomers like the ACBD3-GRASP55-Golgin45-TBC1D22 cisternal adhesion complex [116].

In addition to the Golgi compartment, ACBD3 is localized at mitochondria where it interacts with the cholesterol translocator protein TSPO/peripheral benzodiazepine receptor (PBR) and the regulatory subunit RIα of protein kinase A (PKA) [126] (Table 1, Table 2; Fig. 4). TSPO is part of a multiprotein complex containing VDAC, StAR and ANT transmitting cholesterol to the inner mitochondrial membrane for steroid synthesis [127]. By interacting in concert with TSPO and RIα, ACBD3 functions as an A kinase anchoring protein (AKAP) connecting the PKA holoenzyme with its target TSPO to regulate steroidogenesis [126,128]. Interestingly, ACBD1 was also reported to interact with TSPO to stimulate steroidogenesis [129,130], which may suggest that both ACBDs contribute to a shared protein network regulating mitochondrial cholesterol transport.

ACBD3 is also observed at the plasma membrane in intestinal and neuronal cells [131,132] (Table 1). In neurons, ACBD3 interconnects the iron transporter DMT1 (divalent metal transporter 1) with the NO-activated GTPase DEXRAS1, thereby enabling regulation of cellular iron uptake [131]. In the intestine, ACBD3 interaction with DMT1 was found to depend on the cellular metabolic state. Under iron starvation conditions, DMT1 and ACBD3 both localized to the apical brush border membrane [132]. In response to iron feeding, the proteins translocated to different subcellular sites (for ACBD3 the basolateral membrane and Golgi). Thus, ACBD3-binding to DMT1 might contribute to the regulation of DMT1 internalization thereby influencing iron influx [132].

In addition to its membrane associated functions, ACBD3 is able to act in the cytosol. During division of neuronal progenitors, the cytosolic cell fate determining protein Numb is asymmetrically distributed among daughter cells inducing specification into a neuron and another progenitor cell for self-renewal [133]. During mitosis-induced Golgi fragmentation, ACBD3 is released into the cytosol where it interacts with Numb's N-terminal region [134,135]. Numb's N-terminal amino acid sequence exhibits two phosphorylation sites targeted by the atypical Protein Kinase C (aPKC) in the process of asymmetric Numb segregation. Thus, ACBD3 might modulate accessibility of these phosphorylation sites thereby regulating asymmetric Numb distribution into daughter cells.

Besides its cellular functions, ACBD3 has been co-opted for the replication of multiple members of the picornavirus family, which include important human pathogens such as poliovirus, enterovirus 71, coxsackieviruses, hepatitis A virus and rhinoviruses. PI4KB is an essential host factor for the formation of so-called replication organelles (RO) – rearranged membrane structures required for viral genome replication [22]. At the RO membrane, PI4KB synthesizes PI4P thereby attracting PI4P-binding proteins to the RO to be exploited for the viral RNA replication machinery [136]. In order to recruit PI4KB to the RO, ACBD3 interconnects the viral 2B, 2BC, 2C, 3A and 3B protein complex with PI4KB [137,138]. Since the viral 3A protein competes with golgins in binding to ACBD3's GOLD domain, the ACBD3-3A interaction likely induces a release of ACBD3 from the Golgi, thus ‘piggybacking’ PI4KB to the RO [118,139]. As reported above, ACBD3 not only binds PI4KB but also amplifies its enzymatic activity, thus increasing PI4P synthesis at RO membrane [125]. Remarkably, ACBD3 has been recently reported to connect the ER-Golgi cholesterol transport machinery with the RO of the Aichi virus [140]. The viral 2B, 2BC, 2C, 3A, and 3AB protein complex was found to interact with the cholesterol transferring oxysterol-binding protein (OSBP) thereby assembling a VAP/OSBP/SAC1 complex at RO-ER membrane contact sites. In this context, ACBD3 is recruited to the RO via interaction with each of the viral proteins. A parallel additional interaction with the VAPA/B, SAC1 and OSBP complex stabilizes the complex between the ER proteins VAP/OSBP/SAC1 and the viral RO proteins in order to interconnect the host ER membrane with the viral RO for cholesterol transfer from the ER to the RO.

Despite the increasing number of identified protein interactions implying that ACBD3 is a central player in cellular signaling and membrane domain organization [29,117], current knowledge on the relevance of ACBD3's name giving ACB domain is surprisingly scarce. Its predicted 3D-structure exhibits an altered structural conformation compared to the experimentally deduced 3D-structures of the ACB domains from ACBD1, ACBD2 and ACBD6, thus implying that ACBD3 binds distinct acyl-CoA species [29]. Moreover, incubation of purified ACBD3 with either palmitoyl- or oleoyl-CoA resulted in the formation of protein oligomers suggesting that the protein changes its conformation upon acyl-CoA binding [119]. This may indicate that ACBD3 function is regulated by acyl-CoA binding. SREBP1 is a nuclear transcription factor activating de novo synthesis of fatty acids [141]. ACBD3 expression was recently shown to attenuate nuclear translocation of SREBP1 by interacting and stabilizing SREBP1's membrane-associated full-length form at the Golgi thereby reducing cellular palmitate synthesis [52]. Expression of ACBD3 lacking the ACB domain did not induce this effect. Thus, ACBD3 might sense intracellular acyl-CoA availability with its ACB domain, which induces changes in ACBD3 conformation stabilizing interaction and Golgi association of full-length SREBP1. In this regard, ACBD3 may help to decrease gene expression of fatty acid synthesizing proteins when intracellular acyl-CoA pools are high. However, the actual binding affinities of ACBD3 for distinct acyl-CoAs have still to be determined in order to understand its particular role in the cellular network regulating lipid homeostasis.

As illustrated in this section, ACBD3 appears to be a multivalent scaffolding protein, regulating interactions between several core protein complexes and some more dynamically or temporarily associated functional proteins. Most of the interactions appear to be confined to the Golgi, where ACBD3 appears to localize by interaction with different member of the golgin-family in order to recruit further proteins to the Golgi-membrane. Nevertheless, the function of the bipartite protein interaction domains of ACBD3 can also be exploited at other subcellular membranes like the mitochondrial outer or plasma membrane. Several of the functions fulfilled by ACBD3 might be independent of the protein's ACB domain, while others might link the cell's lipid metabolic status with the general cellular homeostasis. In this respect, it remains unclear if the ACB domain was fused to a precursor protein with an already complex interactome, or if novel acyl-CoA binding independent functions were acquired after fusion to its C-terminal extension by a stepwise diversification protein interaction network. In order to understand ACBD3s role in cellular physiology, it will be important to unravel how ACBD3 can coordinate its large, variable and dynamic protein interaction network, and to identify potential targets for posttranslational modifications, which could facilitate the underlying regulatory mechanisms.

## 6 ACBD4/5 and Atg37 proteins

ACBD4 and ACBD5 proteins are only found in animals and fungi (Figs. 1, S1). The transcript variants encoding a C-terminal TMD have recently been found to localize to peroxisomes [7] (Table 1, Table 2; Fig. 4). The autophagy related protein Atg37 is the homolog of ACBD4/5 in fungi and likewise an ACB domain protein at the peroxisomal membrane. In the yeast *Pichia pastoris*, Atg37 is required for the formation of phagophores (membrane precursors formed upon induction of autophagy) during pexophagy, the specific degradation of peroxisomes [47,142]. The role of Atg37 in pexophagy is in the recruitment of the pexophagic receptor complexes which link peroxisomes to the autophagic machinery. Atg37 interacts with the pexophagy-specific autophagy receptor Atg30, allowing recruitment of peroxisomes to the pexophagic complex, and facilitating efficient degradation of peroxisomes [142]. Interestingly the interaction between Atg37 and Atg30 requires the ACB domain of Atg37, and in vitro Atg30 and palmitoyl-CoA compete for Atg37 binding, suggesting a potential mechanism for how Atg37-mediated pexophagy could be regulated [47]. Mammalian ACBD5s and isoform 2 of ACBD4 are C-tail-anchored peroxisomal membrane proteins with an N-terminal ACB domain [7,47,143,144] (Fig. 3). It was recently revealed that ACBD5 and ACBD4 function as molecular tethers in the formation of peroxisome-ER membrane contact sites (MCSs) [4,5,9] (Table 1, Table 2; Fig. 4). An estimated 80–90% of peroxisomes are closely associated with the ER, suggesting an important physiological role of those contacts in mammalian cells [145]. Both ACBD5 and ACBD4 interact through a FFAT-like motif with VAP proteins (VAPA, VAPB) at the ER [4,5,9]. VAP proteins (vesicle-associated membrane protein (VAMP)–associated proteins) are C-tail-anchored adaptor proteins in the ER membrane with roles in inter-organellar lipid exchange, MCS formation, and membrane trafficking [146]. They possess a major sperm protein (MSP) domain that interacts with the FFAT(-like) motif of protein partners located on the opposing membrane [147]. Supporting a function in peroxisome-ER tethering, co-expression of ACBD5 and VAPB increased contact sites, whereas depletion reduced them. Whereas mutations in the ACBD4/5 FFAT-like motif abolished interaction with VAPB and reduced peroxisome-ER interaction, a functional N-terminal ACB domain was not required for the tethering function [4,5,9]. In our analysis of the FFAT motif of ACBD4/5 proteins across species (see Section 2) we could not detect a specific FFAT motif in *Pichia pastoris* Atg37 suggesting that, unlike ACBD4/5, it may not have ER tethering function. Conversely, ACBD4/5 may not have a function in pexophagy [6,8].

Disruption of the ACBD5-VAP contacts increased peroxisome motility, revealing a new role of peroxisome-ER tethering in the regulation of peroxisome mobility and positioning [4,5] (Table 1). Loss of peroxisome-ER contacts also prevented peroxisomal membrane expansion, which is required for the formation of peroxisomes by membrane growth and division [4,148]. Conversely, overexpression of ACBD5 in cells deficient in peroxisome fission induced peroxisome elongation [5]. These observations support a role of the ACBD5-VAP contact in peroxisome biogenesis and membrane lipid transfer. Many of the metabolic functions of peroxisomes in lipid metabolism are carried out in partnership with the ER [14]. This includes the synthesis of ether-phospholipids (e.g. myelin sheath lipids), which in mammalian cells is initiated in peroxisomes but completed in the ER. The first patients diagnosed with a functional ACBD5 deficiency presented with retinal dystrophy and white matter disease [149]. Further characterization revealed a peroxisome-based disorder with progressive leukodystrophy, ataxia, progressive microcephaly with facial dysmorphisms, in addition to retinal dystrophy [6,8]. Patients showed a loss of ACBD5 protein, a reduction in ether-phospholipids [150], and accumulation of VLCFAs due to a dysfunction in peroxisomal β-oxidation [6,8]. It is suggested that ACBD5 facilitates transport of VLCFAs into peroxisomes for subsequent β-oxidation [6,8,150]. We hypothesize that the ACBD5-VAP tether contributes to the formation of a peroxisome-ER metabolic hub that allows control of fatty acid chain length: Fatty acids elongated at the ER can be prevented from synthetizing excess amounts of over-long VLCFA via transmission to peroxisomes for immediate degradation via β-oxidation [17]. In this context it should be noted that a disruption of peroxisome metabolism not only prevents VLCFA degradation but induces fatty acid elongation at the ER [151] suggesting that fatty acid synthesis and break down are regulated in a tightly controlled feed-back mechanism, potentially via coordinating catabolic and anabolic pathways at peroxisome-ER contact sites. Remarkably, the long-chain acyl-CoA synthetase ACSL1 was identified as a direct interaction partner of ACBD5 and VAPB [152]. As outlined above, there is evidence that inter-organelle communication at peroxisome-ER contacts mediated by the ACBD5-VAP tether regulates several physiological processes including fatty acid/lipid metabolism, membrane lipid exchange, peroxisome biogenesis, mobility and positioning [17] (Table 1, Table 2).

Interestingly, a recent interactomics study uncovered an important role for peroxisomes in Zika virus infection and revealed the peroxisome-ER tether proteins ACBD5 and VAPA/B as host interaction proteins [21]. The study used BioID and IP-MS to generate a global Zika virus-host protein interactome for the ten polypeptides encoded in the viral genome. Extensive organellar targeting of Zika virus to host cell organelles involved in lipid biosynthesis and metabolism, including the ER, peroxisome and lipid droplets was uncovered [21]. The non-structural viral protein NS2A specifically targeted peroxisomal membranes, and Zika virus replication was significantly impaired in cells lacking functional peroxisomes. The authors propose that NS2A targets peroxisomes to remodel host-cell lipid populations and/or to effect viral escape from the host innate immune response, which was previously shown for Dengue and West Nile virus [153]. Those viruses can cause mosquito-borne neurological disease and death. Lipidomic analysis revealed increased levels of plasmalogens in serum of Zika virus-infected subjects further suggesting a link between viral life cycle and peroxisomes, which are required for plasmalogen biosynthesis [154].

There is firm evidence now that ACBD4/5 mediate tethering of peroxisomes to the ER and contribute to fatty acid/lipid metabolism at the peroxisome-ER interface. It remains to be established how fatty acids/lipids are exchanged between peroxisomes and ER at those contacts, and how these mechanisms impact on human health and disease.

## 7 ACBD6 proteins

ACBD proteins carrying ankyrin-repeat motifs represent the second most abundant ACBD forms predicted from eukaryotic genomes [1]. The representative member of the mammalian family is annotated as ACBD6 (Fig. 3). It was first identified in hematopoietic cells [155], but is produced in other tissues and cells [156,157]. The human ACBD6 is a soluble protein detected in the cytosol and nuclei of cells [155] and is involved in the regulation of acylation of lipids and proteins [19,119,158,159] (Table 1). ACBD6 supports the re-acylation of lysophospholipids, which is an essential reaction necessary for the repair/renewal of damaged lipids in membranes. ACBD6 controls the rate of acylation of lipids at the level of acyl-CoA:lysophospholipid acyltransferases (LPLATs), preventing the inhibition of the LPLAT by excess acyl-CoAs. Acyl-CoAs have detergent-like properties that can damage proteins and the acyl-CoA sequestration by ACBD6 appears to protect the enzymes by limiting availability of free acyl-CoAs [119]. Interestingly, the acylation supporting function of ACBD6 can be exploited by pathogens. In cells infected with the bacterium *Chlamydia trachomatis*, ACBD6 was found to be recruited into the parasitophorous vacuole and depleted from the host compartment [19,160]. Moreover, ACBD6 modulates the bacterial acylating enzymes and may support remodeling of host lipids with bacterial-derived branched-chain fatty acids in pathogen infected cells [19,119,161]. Interestingly, peroxisomes are also translocated to the parasitophorous vacuole and may be exploited to produce bacteria-specific plasmalogens [162].

The N-myristoylation of proteins can affect their activity and association with membranes, and mediates oligomeric assembly and the interaction with other proteins [163,164]. After the removal of the initiator methionine, N-myristoyltransferases (NMTs) catalyze the amide linkage of the rare 14-carbon long saturated chain of myristoyl-CoA to an N-terminal glycine of proteins [163,[165], [166], [167]]. They are essential for the maintenance of cellular functions but as well for the intracellular development of pathogens such as parasitic protozoa, fungi, and viruses [[168], [169], [170], [171], [172]]. In this pathway, human ACBD6 stimulates the activity of the N-myristoyltransferases NMT1/NMT2 and provides specificity for their substrate [158,159] (Table 1, Table 2; Fig. 4). N-myristoyltransferases lack binding specificity for the 14-carbon acyl chain and other acyl-CoAs, such as the more abundant C16-CoA, can bind and block the enzymes [163,173,174]. ACBD6 interacts with NMT2 and forms a complex via its C-terminal domain, in which the ankyrin-repeat motifs are located [159]. Likewise, the *Drosophila* ACBD6 homolog *Anox* interacts with the NMT enzyme and may indicate that a similar mechanism is conserved in the fruit fly [175,176]. In the complex, ACBD6 stimulates activity and favors binding of C14-CoA to NMT2 even in the presence of competing acyl-CoAs. Complex formation does not require the ACB domain but the stimulatory effect of ACBD6 is dependent on both the presence of the ankyrin-repeat region and of an intact ACB domain. Acyl-CoA-bound ACBD6 forms a more stimulatory complex than apoACBD6, and ACB mutants deficient in ligand binding are impaired in their ability to stimulate NMT activity [158,159]. Phosphorylation of ACBD6 enhances the N-myristoyltransferase activity [158]. The regulation of N-myristoyltransferase is even conserved in the apicomplexan parasite *Plasmodium falciparum* ACBD6-like protein. This enzyme is more sensitive to competition by C12-CoA rather than C16-CoA and both *P. falciparum* and human ACBD6 protein can provide protection against C12-CoA [158].

Additionally, ACBD6 may be involved in the control of cellular metabolism at different regulatory levels as suggested by findings from invertebrate homologs. The loss of the *Drosophila* ACBD6 *Anox* in a mutant fly resulted in a significant decrease in feeding activity and food intake [175]. *Anox* is expressed in chemosensory organs and neurons of the central nervous system and appears essential for the regulation of insulin signaling by modulating sugar-induced nerve responses. In this respect, it should be mentioned that lipid and glucose-sensitive neurons of the CNS appear to form complex regulatory networks required for maintaining energy and glucose homeostasis [177], which might partially explain the association of an acyl-CoA binding protein with insulin signaling pathways in the fruit fly. The absence of the *Caenorhabditis elegans* ACBD6 homolog *Ce*ACBP-5 led to a 50% increase in the production of lipid droplets [178]. In the nematode, acyl-CoA binding proteins appear to regulate triacylglycerol metabolism, which was also drastically affected by loss of *Ce*ACBP-1.

Remarkably, acyl-CoA binding in ACBD6 appears to be regulated by phosphorylation. While several ACBD members are phosphorylated in vivo, ACBD6 appears to be the only member that is phosphorylated on residues within the ACB domain [158,179,180]. The two ACBD6 residues known to be phosphorylated are serine 106 (Ser106) and Ser108 which are −1 and +1 residue of the fourth α–helix of the ACB domain of human ACBD6 (according to the structural information available at entry PDB:2COP of ProteinDataBase). These serine residues are not essential for acyl-CoA binding and for the localization of ACBD6 [158]. Ser106 is conserved in the ACB domain of some other ACBD proteins but it is not known to be phosphorylated in these forms and the first residue of α-helix 4 (Ser108 in ACBD6) is often replaced by a glutamate residue. Purified phospho-ACBD6 protein has higher acyl-CoA binding capacity than ACBD6 and displacement of bound acyl-CoA to phospho-ACBD6 required a higher concentration of competitors compared to the non-phosphorylated form [158]. Glutamate can often mimic phosphorylation of a serine residue due to its negative charge, and the ‘permanent’ phosphorylation of the +1 residue of α–helix 4 compared to an alternative phospho-state of Ser108 supports the notion that acyl-CoA binding activity of ACBD6 can be regulated in vivo. Cell cycle global profiling of human phospho-proteins detected a specific upregulation of phosphorylation of ACBD6 Ser106 and Ser108 during the M-phase [179,180]. This finding strongly suggests that function of the phosphorylated form of ACBD6 is increased during mitosis and also raise the question whether de-phosphorylation is regulated.

Ankyrin-repeat containing ACBDs are not restricted to metazoans but are found among all three organismal phyla and were initially identified in *Arabidopsis thaliana* [154,155]. Of note, a general difference between the ACBP class II found in plants and the ACBD6 forms of metazoans, fungi and amoebozoa is the presence of a potential N-terminal TMD or hydrophobic signaling sequence (Fig. 3). In line with this motif, the two plant homologs *At*ACBP1 and *At*ACBP2 are located in the ER and plasma membrane but it remains to be determined if the N-terminal sequence functions as a true membrane anchor [57,156,157]. *At*ACBP1 and *At*ACBP2 are essential for embryonic development and absence of ACBP1 alters adaptation to cold and drought, heavy-metal stresses, sterol and very-long acyl-CoA metabolism and abscisic acid signaling [58,[158], [159], [160], [161], [162], [163]]. *At*ACBP2 interacts with the ethylene-responsive element-binding protein *At*EBP via its ankyrin-repeat motifs [157]. *At*ACBP1 protein has been shown to interact with a broad variety of compounds, such as lead [161], the phospholipids phosphatidylcholine and phosphatidic acid [159,160], and the phospholipase PLDα [159]. Moreover, ACBP class II proteins play a role in pathogen infection and growth. The absence of ACBP1 increases susceptibility of leaves to infection by the fungal pathogen *Botrytis cinerea* as a result of a decreased wax production and weakened cuticle layer [163].

Orthologous ankyrin-containing proteins from apicomplexan parasites (alveolata) are also membrane bound, but their N-terminal structure is heterogeneous. *Cryptosporidium parvum Cp*ACBP1 is associated to the parasitophorous vacuolar membrane but does not contain a potential TMD sequence [164,165]. *Plasmodium falciparum*, *Plasmodium vivax* and *Toxoplasma gondii* possess ACBD6-like proteins which exhibit predicted transmembrane motifs at their N-terminal end [164,166] and are thus potentially membrane-anchored. *Toxoplasma gondii Tg*ACBP2 is localized in the mitochondrial membrane but the phosphorylated form is sorted to the plasma membrane [167]. The acyl-CoA binding activity of the protozoan *Cryptosporidium parvum*, *Cp*ACBP1 and of *Plasmodium falciparum Pf*ACBD6 have been determined and show highest affinities for a chain length between 14–16 C-atoms [164,165]. *Toxoplasma gondii Tg*ACBP2 is important for cardiolipin metabolism and intracellular growth of the parasite [167], while the function of the homologs from *Cryptosporidium* and *Plasmodium* is still unknown. As exemplified in the last paragraph, evolutionary radiation can significantly modulate the protein architecture of ACBD orthologues in different organism groups. In this respect, the proteins may combine functions which are still conserved among species with novel ones which were e.g. acquired by gaining access to alternative subcellular localizations with different surrounding protein environments.

The functional relevance of mutations in the ACBD6 gene, which have not been associated with a disease so far, is now under investigation. Homozygous loss-of-function mutations in ACBD6 have been identified in individuals with a neurodevelopmental disorder (ES&FK, personal communication). Analysis of the processes regulated by ACBD6 (protein and lipid acylation) affecting the activities of neuronal proteins by lipid-modification represents a new avenue of research in neuron biology.

## 8 Concluding remarks

There is compelling evidence now that ACBD proteins are ancient proteins which have evolved to fulfill other functions in addition to their original roles in acyl-CoA transport and stabilization. The small soluble ACBDs appear to act as acyl-CoA transporters and reservoirs, enabling efficient provision and targeting of acyl-CoA to lipid metabolizing subcellular compartments. However, extended ACBDs have evolved which combine ACB domains with a membrane anchor, enzymatic activity or protein interaction domains. An increase in the complexity of ACBDs may have been driven by the evolution of more complex subcellular compartments. This has led to the acquisition of additional functions, which are no longer directly linked to the original acyl-CoA binding function. It also allowed ACB domain-containing proteins to be linked to specific subcellular membranes, where they developed organelle-specific functions in acquiring lipids for metabolic pathways, membrane lipid trafficking or signaling. Several ACBDs have been linked to peroxisome function and act as tethers or scaffold proteins at membrane contact sites. The medical importance of ACBDs is also increasingly recognized, as several are targeted by different pathogens to exploit cellular lipid metabolism. ACBD5 deficiency has recently been described as a new peroxisomal disorder characterized by the accumulation of VLCFA and neurological abnormalities (see Section 6). It will now be important to identify and characterize further interaction partners of ACBDs at organelle membranes, to understand how protein scaffolds at membranes are formed, and how their formation and ACBD interaction is regulated. Posttranslational modifications such as phosphorylation likely play a role. A challenge will be to investigate if and how acyl-CoA binding regulates protein interactions, and how this may be influenced by interaction of the ACB domain with different substrates. Further understanding of the molecular mechanisms and function of ACBDs may lead to therapeutic approaches to combat pathogens and impact on our understanding of human disease.

The following are the supplementary data related to this article.

Supplementary Fig. S1 Cladogram for ACBD containing sequences found in animals, fungi and plants (animal branches in red, plant branches in green, fungal branches in blue). The sequences were derived from GenBank and JGI and cover the major metazoan branches; the cladogram was constructed with PHYML 3.0 contained in the Seaview software package. Circular cladograms were drawn with Mesquite v3.2. Numbers at the branch nodes represent branching probabilities. *Several sequences from other early branching fungal groups are included in the Mucoromycota section. Supplementary Fig. S1

Supplementary Fig. S2 Evolution of the 3 small, soluble ACBDs found in mammals. The sequences were derived from GenBank and cover all major vertebrate and invertebrate classes; ACBD2 sequences branching next to ACBD1 in Fig. S1 were used as outgroup. The cladogram was constructed with PHYML 3.0 contained in the Seaview software package. Circular cladograms were drawn with Mesquite v3.2. As shown, all vertebrates possess two small soluble forms – ACBD1 and ACBD7. In addition, a second gene duplication resulted in another form – ACBD8 – found in mammals including marsupials. Interestingly, similar sequences were found in the Archosauria and Testudines indicating that ACBD8 may have been originated already during reptilian evolution. However, more reptilian sequences are required to more reliably reconstruct the evolutionary relation of these sequences. Numbers at the branch nodes represent branching probabilities. i.s., incertae sedis; vert., vertebrata; invert., invertebrata. Supplementary Fig. S2

Supplementary Fig. S3 Alignment of selected small ACBD proteins from animals, plants, fungi, choanoflagellates, eubacteria and archaea. Conserved residues are indicated by asterisks, neutral amino acid substitutions by colons/points. Note that the major amino acid residues involved in acyl-CoA binding (Lys33, Lys55, Tyr29, Tyr32, Tyr74, Phe6, Leu26, [36]) are conserved in all species. Alignment was performed with ClustalW 2.1 [184]. Supplementary Fig. S3

Supplementary Fig. S4 Phylogenetic analysis of small ACBD forms found in prokaryotes. The sequences were derived from GenBank and contain sequences from the major prokaryote and eukaryote organism groups. Archaeal sequences are derived from metagenome sequencing data. Circular cladograms were drawn with Mesquite v3.2. Vertebrate ACBD2 sequences as depicted in Fig. S1 were used as the outgroup. The cladogram was constructed with PHYML 3.0 contained in the Seaview software package. Note that the small ACBP-like sequences identified in β-, γ-, δ-proteobacteria, bacteroidetes and archaea cluster in distinct branches next to the eukaryotic ACBDs implying an evolution from a shared early ancestor protein. In contrast, the few sequences found in α-proteobacteria (α) and actinobacteria (Ac) align with sequences from different branches, suggesting that they might have arisen by lateral gene transfer or result from contaminated DNA. Numbers at the branch nodes represent branching probabilities. *Several sequences from other early branching fungal groups are included in the Mucoromycota section. i.s., incertae sedis. Supplementary Fig. S4

Supplementary Table S1 Analysis of FFAT motifs. In the initial analysis 56 fungal and 37 animal (20 vertebrate and 17 invertebrate) ACBD5 protein sequences were analysed for FFAT motifs using a previously described position weighted matrix analysis [48]. This gives a FFAT “score” based on the number of suboptimal elements within the motif with a cut-off of ≤2.5. This analysis was then expanded to include further fungal species to look for enrichment in a particular Order/Class. To confirm significance for the Animal, Fungal and Ascomycota sequences a randomisation analysis was performed in which amino acid sequences were randomised 10 times and the FFAT analysis repeated to give a false positive discovery or background rate. For each strength of FFAT score (≤1.0, ≤1.5, ≤2.0, ≤2.5), the observed number of motifs was compared with the background discovery rates across all amino acid residues, using the N-1 Chi-squared test [49]. Supplementary Table S1

Supplementary material 1 Sequences used for Fig. S1. Supplementary material 1

Supplementary material 2 Sequences used for Fig. S2. Supplementary material 2

Supplementary material 3 Sequences used for Fig. S4. Supplementary material 3

## Details of the contributions of individual authors

MS and MI planned the manuscript. MI, TL and JC performed analyses. MI, MS and JC prepared the figures. All authors contributed to the writing of the manuscript.

## Declaration of competing interest

The authors declare that they have no known competing financial interests or personal relationships that could have appeared to influence the work reported in this paper.
