# Supplementary figures and images for: The diversity of ACBD proteins – From lipid binding to protein modulators and organelle tethers
Source: Biochim Biophys Acta Mol Cell Res. 2020 May;1867(5):118675. doi: 10.1016/j.bbamcr.2020.118675 (PMC7057175; doi:10.1016/j.bbamcr.2020.118675)

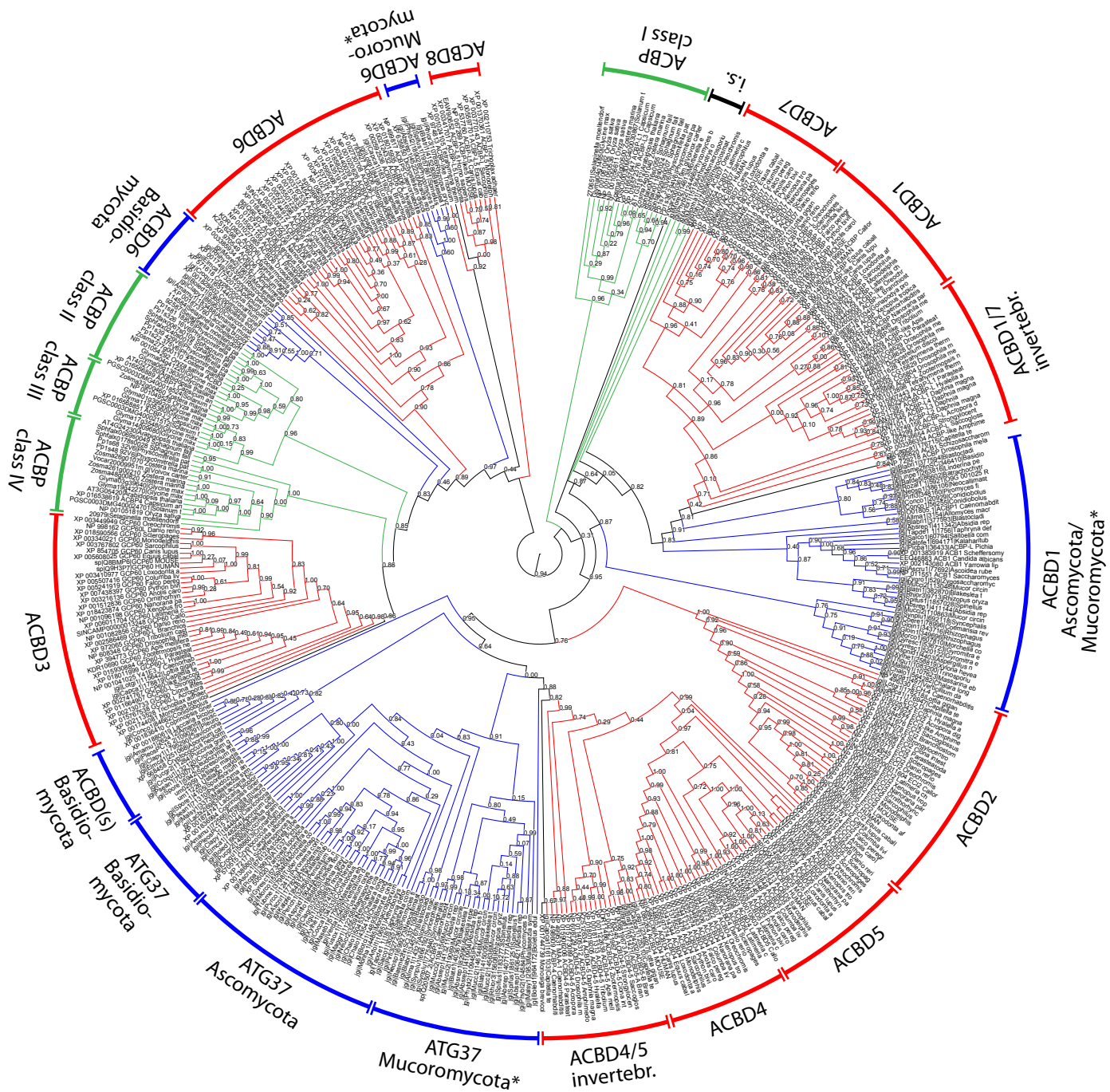

Supplement: Supplementary Fig. S1 — Cladogram for ACBD containing sequences found in animals, fungi and plants (animal branches in red, plant branches in green, fungal branches in blue). The sequences were derived from GenBank and JGI and cover the major metazoan branches; the cladogram was constructed with PHYML 3.0 contained in the Seaview software package. Circular cladograms were drawn with Mesquite v3.2. Numbers at the branch nodes represent branching probabilities. *Several sequences from other early branching fungal groups are included in the Mucoromycota section. [file mmc1.pdf]

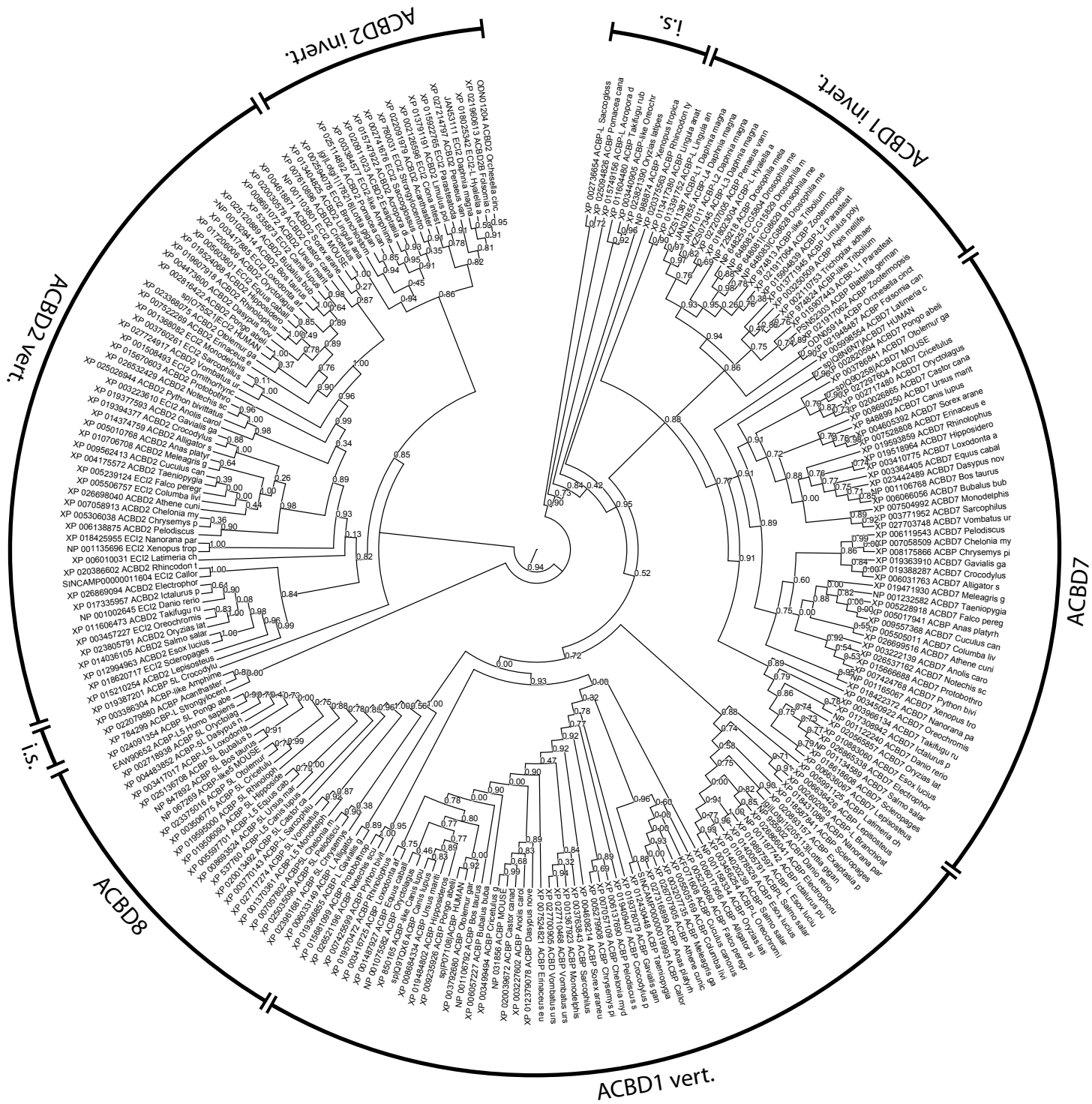

Supplement: Supplementary Fig. S2 — Evolution of the 3 small, soluble ACBDs found in mammals. The sequences were derived from GenBank and cover all major vertebrate and invertebrate classes; ACBD2 sequences branching next to ACBD1 in Fig. S1 were used as outgroup. The cladogram was constructed with PHYML 3.0 contained in the Seaview software package. Circular cladograms were drawn with Mesquite v3.2. As shown, all vertebrates possess two small soluble forms – ACBD1 and ACBD7. In addition, a second gene duplication resulted in another form – ACBD8 – found in mammals including marsupials. Interestingly, similar sequences were found in the Archosauria and Testudines indicating that ACBD8 may have been originated already during reptilian evolution. However, more reptilian sequences are required to more reliably reconstruct the evolutionary relation of these sequences. Numbers at the branch nodes represent branching probabilities. i.s., incertae sedis; vert., vertebrata; invert., invertebrata. [file mmc2.pdf]

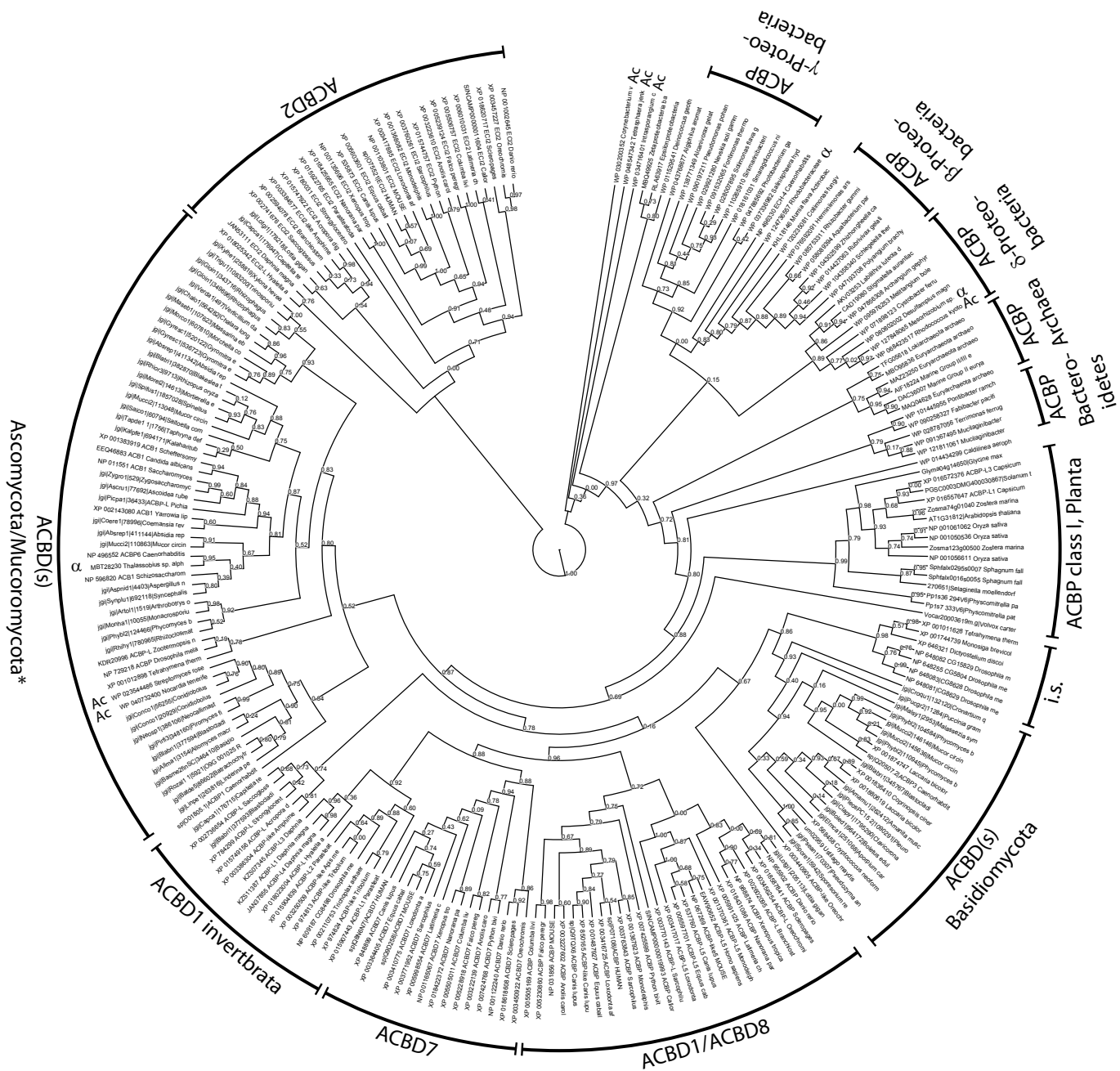

Supplement: Supplementary Fig. S4 — Phylogenetic analysis of small ACBD forms found in prokaryotes. The sequences were derived from GenBank and contain sequences from the major prokaryote and eukaryote organism groups. Archaeal sequences are derived from metagenome sequencing data. Circular cladograms were drawn with Mesquite v3.2. Vertebrate ACBD2 sequences as depicted in Fig. S1 were used as the outgroup. The cladogram was constructed with PHYML 3.0 contained in the Seaview software package. Note that the small ACBP-like sequences identified in β-, γ-, δ-proteobacteria, bacteroidetes and archaea cluster in distinct branches next to the eukaryotic ACBDs implying an evolution from a shared early ancestor protein. In contrast, the few sequences found in α-proteobacteria (α) and actinobacteria (Ac) align with sequences from different branches, suggesting that they might have arisen by lateral gene transfer or result from contaminated DNA. Numbers at the branch nodes represent branching probabilities. *Several sequences from other early branching fungal groups are included in the Mucoromycota section. i.s., incertae sedis. [file mmc4.pdf]
