# Supplementary material for: The diversity of ACBD proteins – From lipid binding to protein modulators and organelle tethers
Source: Biochim Biophys Acta Mol Cell Res. 2020 May;1867(5):118675. doi: 10.1016/j.bbamcr.2020.118675 (PMC7057175; doi:10.1016/j.bbamcr.2020.118675)
Supplement: Supplementary Fig. S3 — Alignment of selected small ACBD proteins from animals, plants, fungi, choanoflagellates, eubacteria and archaea. Conserved residues are indicated by asterisks, neutral amino acid substitutions by colons/points. Note that the major amino acid residues involved in acyl-CoA binding (Lys33, Lys55, Tyr29, Tyr32, Tyr74, Phe6, Leu26, [36]) are conserved in all species. Alignment was performed with ClustalW 2.1 [184]. [file mmc3.docx]

Rhizopus_oryzae -------MPSQQ**F**TT**A**AEEVQK--LSTKPSNDEL**L**EL**Y**GLF**K**QATV**G**DNETSK

Sphagnum_fallax ------MGLKED**F**DQ**A**AKDALT--LPESTTNEDK**L**IL**Y**GLF**K**VATV**G**KPETSR

Homo_sapiens ----MEMDLKEE**F**EK**A**SETVMN--LSERPSNEEL**L**KL**Y**SFY**K**QGTE**G**DVSGKR

Aquabacterium_parvum_beta -----MSDLQAQ**F**EK**A**LADSKL--LPAKPDNNTL**L**KI**Y**SLF**K**QGSV**G**DVQGDR

Lokiarchaeota_archaeon MSVDNGKTLKSE**F**EE**A**IARSDK--LPKQP-VDTQ**L**EL**Y**GLY**K**QALF**G**DVTGER

Monosiga_brevicollis -------MTEAQ**F**NK**A**VWLIRNGPAVGDSSNETK**L**SF**Y**KYY**K**QATV**G**DNNESQ

. :***** ***** . : ***** :***** :***** . *****. .:

---HHHHHHHHHHHHHHH--------HHHHHHHHHHHHHH---------

Rhizopus_oryzae **P**-TFDIKGRY**K**WDA**W**TKLKGMSQEE**A**EQK**Y**IELVEKLKASQ-----------

Sphagnum_fallax **P**GIFDPKGRA**K**WDA**W**KKVEDKSKDE**A**MQE**Y**IVKVTQLKEA------------

Homo_sapiens **P**GMINLKGRA**K**YDA**W**AKLEGMHAQE**A**QKN**Y**VELVANLLGK------------

Aquabacterium_parvum_beta **P**GMMDFVGRA**K**YDA**W**AELKGKSQDA**A**KQA**Y**IDLIESLKK-------------

Lokiarchaeota_archaeon **P**GRLKVKDRA**K**FDN**W**ESRKGMLKED**A**MKA**Y**ITLIEKLEQEKK----------

Monosiga_brevicollis **P**WAVQLEASA**K**WKA**W**NSVRGMSKED**A**MKA**Y**VDLLAKDDPNWEQHPALKDYKA

***** .. *****:. ***** . .. : * : *: : .

-----HHHHHHHHHHHH-----HHHHHHHHHHHHHHHH--
