# Supplementary material for: The diversity of ACBD proteins – From lipid binding to protein modulators and organelle tethers
Source: Biochim Biophys Acta Mol Cell Res. 2020 May;1867(5):118675. doi: 10.1016/j.bbamcr.2020.118675 (PMC7057175; doi:10.1016/j.bbamcr.2020.118675)
Supplement: Supplementary material 1 — Sequences used for Fig. S1. [file mmc6.docx]

>sp|Q5T8D3|ACBD5_HUMAN

MFQFHAGSWESWCCCCLIPADRPWDRGQHWQLEMADTRSVHETRFEAAVKVIQSLPKNGSFQPTNEMMLKFYSFYKQATEGPCKLSRPGFWDPIGRYKWDAWSSLGDMTKEEAMIAYVEEMKKIIETMPMTEKVEELLRVIGPFYEIVEDKKSGRSSDITSVRLEKISKCLEDLGNVLTSTPNAKTVNGKAESSDSGAESEEEEAQEEVKGAEQSDNDKKMMKKSADHKNLEVIVTNGYDKDGFVQDIQNDIHASSSLNGRSTEEVKPIDENLGQTGKSAVCIHQDINDDHVEDVTGIQHLTSDSDSEVYCDSMEQFGQEESLDSFTSNNGPFQYYLGGHSSQPMENSGFREDIQVPPGNGNIGNMQVVAVEGKGEVKHGGEDGRNNSGAPHREKRGGETDEFSNVRRGRGHRMQHLSEGTKGRQVGSGGDGERWGSDRGSRGSLNEQIALVLMRLQEDMQNVLQRLQKLETLTALQAKSSTSTLQTAPQPTSQRPSWWPFEMSPGVLTFAIIWPFIAQWLVYLYYQRRRRKLN

>sp|Q9BR61|ACBD6_HUMAN

MASSFLPAGAITGDSGGELSSGDDSGEVEFPHSPEIEETSCLAELFEKAAAHLQGLIQVASREQLLYLYARYKQVKVGNCNTPKPSFFDFEGKQKWEAWKALGDSSPSQAMQEYIAVVKKLDPGWNPQIPEKKGKEANTGFGGPVISSLYHEETIREEDKNIFDYCRENNIDHITKAIKSKNVDVNVKDEEGRALLHWACDRGHKELVTVLLQHRADINCQDNEGQTALHYASACEFLDIVELLLQSGADPTLRDQDGCLPEEVTGCKTVSLVLQRHTTGKA

>sp|Q8NC06|ACBD4_HUMAN

MGTEKESPEPDCQKQFQAAVSVIQNLPKNGSYRPSYEEMLRFYSYYKQATMGPCLVPRPGFWDPIGRYKWDAWNSLGKMSREEAMSAYITEMKLVAQKVIDTVPLGEVAEDMFGYFEPLYQVIPDMPRPPETFLRRVTGWKEQVVNGDVGAVSEPPCLPKEPAPPSPESHSPRDLDSEVFCDSLEQLEPELSSGQHLEESVIPGTAPCPPQRKRGCGAARRGPRSWTCGCWGQFEHYRRACRRCRRGCRAWRACPGPLSSLTLSVRLE

>sp|O75521|ECI2_HUMAN

MAMAYLAWRLARRSCPSSLQVTSFPVVQLHMNRTAMRASQKDFENSMNQVKLLKKDPGNEVKLKLYALYKQATEGPCNMPKPGVFDLINKAKWDAWNALGSLPKEAARQNYVDLVSSLSPSLESSSQVEPGTDRKSTGFETLVVTSEDGITKIMFNRPKKKNAINTEMYHEIMRALKAASKDDSIITVLTGNGDYYSSGNDLTNFTDIPPGGVEEKAKNNAVLLREFVGCFIDFPKPLIAVVNGPAVGISVTLLGLFDAVYASDRATFHTPFSHLGQSPEGCSSYTFPKIMSPAKATEMLIFGKKLTAGEACAQGLVTEVFPDSTFQKEVWTRLKAFAKLPPNALRISKEVIRKREREKLHAVNAEECNVLQGRWLSDECTNAVVNFLSRKSKL

>sp|Q9H3P7|GCP60_HUMAN

MAAVLNAERLEVSVDGLTLSPDPEERPGAEGAPLLPPPLPPPSPPGSGRGPGASGEQPEPGEAAAGGAAEEARRLEQRWGFGLEELYGLALRFFKEKDGKAFHPTYEEKLKLVALHKQVLMGPYNPDTCPEVGFFDVLGNDRRREWAALGNMSKEDAMVEFVKLLNRCCHLFSTYVASHKIEKEEQEKKRKEEEERRRREEEERERLQKEEEKRRREEEERLRREEEERRRIEEERLRLEQQKQQIMAALNSQTAVQFQQYAAQQYPGNYEQQQILIRQLQEQHYQQYMQQLYQVQLAQQQAALQKQQEVVVAGSSLPTSSKVNATVPSNMMSVNGQAKTHTDSSEKELEPEAAEEALENGPKESLPVIAAPSMWTRPQIKDFKEKIQQDADSVITVGRGEVVTVRVPTHEEGSYLFWEFATDNYDIGFGVYFEWTDSPNTAVSVHVSESSDDDEEEEENIGCEEKAKKNANKPLLDEIVPVYRRDCHEEVYAGSHQYPGRGVYLLKFDNSYSLWRSKSVYYRVYYTR

>sp|P07108|ACBP_HUMAN

MSQAEFEKAAEEVRHLKTKPSDEEMLFIYGHYKQATVGDINTERPGMLDFTGKAKWDAWNELKGTSKEDAMKAYINKVEELKKKYGI

>sp|Q8N6N7|ACBD7_HUMAN

MALQADFDRAAEDVRKLKARPDDGELKELYGLYKQAIVGDINIACPGMLDLKGKAKWEAWNLKKGLSTEDATSAYISKAKELIEKYGI

>EAW90652_ACBP-L5_Homo_sapiens

MGDAGATAAALRPAHNLRPAPPTASAAHAQSSRTSAPSAQRRLPAEPSHQPSGTRTSLTRPLRTVPCPAPLPGSQRVGLMIPRPRIHPHPLLAPGTASTTPCAKWSSSCAALKQLKGPVSDQEKLLVYGLYKQATQGDCDIPGPPASDVRARAKWEAWSAKKGASKMDAMRGYAAKVEELTKKEVGGVEREQRGVQDGRHEGLRGQSGGADEEGRASKMDAMRGYAARVRR

>XP_005597701_ACBP-L5_Equus_caballus

MCQVEFELACAAIKQLKGPVSDQEKLLVYSFYKQATQGDCNIPAPPATDVKAKAKWDAWNDKKGISKMDAMRIYVAKVEELKKNDTG

>XP_001487927_ACBP_Equus_caballus

MSQAEFDRAAEEVKNLKTKPADDEMLFIYSHYKQATVGDVNTERPGMLDLKGKAKWDAWNALKGTSKEDAMKAYINKVEDLKKKYGI

>XP_003364405_ACBD7_Equus_caballus

MSLQAEFDRVAEDVRKLKRRPDDAELKELYGLYKQSVIGDIDIECPAMLDLKGKAKWEAWNLQKGLSKEDAMSAYVSKARELIEKYGI

>XP_005603601_ECI2_Equus_caballus

MAGAAVRLARRWAASAVRSPVQDARFPALKLHVGGTAMRASQKDLENAVNQMKLLKKDPGNEVKLKLYALYKQATEGPCNMPKPGIYDLIRKAKWEAWNTLGNLPKETARQNYVDLVSSLCSSSESSSQGKPGADREQLRYETLVVTSEDGITKIMLNRPSKKNAVNLQMYQEIVLALKAASQDDSTITVLTGNGDYYSSGNDLTNFTNTSPGEIQEKAKNSLILVREFVGCFIDFPKPLIAVVNGPAVGIAVTLLGLFDIVYASDRATFHTPFSRLGLHPEGCSSYTFPKIMGPTKATEMLIFGKKLTAEEACAQGLVTEVFPDRTFQKEVWTRLKAYAKLPSNVLRISKQVIRNREKEKLHSVNVEENSVLQGTVLSDEAVNAVMNFLSKRAKL

>XP_014595068_ACBD6_Equus_caballus

MGTPAEGIPPESRARGVQEPGRAVREGCGDLQGLMQVASREQLLYLYARYKQVKVGNCNTPKPGFFDFEGKQKWEAWKALGDSSPSQAMQEYIAAVKKLDPGWNPQSPEKKGKAANTGFGGPVVSSLYHEEIIREEDKNIFDYCRENNIDHITKVIKSKNVDVNMKDEEGRALLHWACDRGHKELVTVLLQYRADINCQDNEGQTALHYAAACEFLDIVELLLQSGADPTLRDQDGCLPEEVTGCKAVSLVLQQHTTGKA

>XP_005597374_ACBD4_Equus_caballus

MGTENESPEPDCQKQFQAAVSVIQNLPKNGSYRPSYEEMLRFYSYYKQATMGPCLVPRPGFWDPIGRYKWDAWNSLGKMSREEAMSAYITEMKLVAQKVIDTVPLGEVAEDMFGYFEPLYEVIPDMPRPPETFLRRVTGWKDQVLNGDAGAALAPPCLPKEPAPPGPESQPPRDLDSEVFSDSLEQLEPELQVWEEQRGALGGEPDSRNSPVPPPEKDGLDGNLLGPQELDAWLVGTVRALQESMRDVQGRLQSLESVSGPRKQQRPQPSARPWPLRLSGPMLLFFLLWPFIVQWLFRHFRTQKR

>XP_014592505_ACBD5_Equus_caballus

MLFLSFHAGSWESWCCCCLIPADTPGDRGRRWRLEMADTRSVHETRFEAAVKVIQSLPKNGSFQPTHEMMLKFYSFYKQATEGPCTLSRPGFWDPVGRYKWDAWSALGDMTKEEAMIAYVEEMKKILETMPMTEKVEELLQVIGPFYEIVEDKKSGRSSDLTSDLGNVLTSTPNAKTINGKAESSDSGAESEEEEAQEEVKGAEQSDNDKKMMKKSSDDKNLEIVVTNGYEKDNLVQDVQNDIHAPSSLNGRSAEEVQPVDPNLEPTGETTVCVHQDINDEHIEDVSGIQHLTSDSDSEVYCDSMEQFGQEESLDSITSNNGPFRYYLGGDPNHPLENSGFPEAVQISPGNGNVGDVQVAAVEGKGEVKHGGEDGGSNTGAPHREKRGGENEEFSNVRRGRGHRMQHLSEGAKGRQVGSGGDGERWGSDRGSRGTLNEQIALVLMRLQEDMQNVLQRLHKLETLTASQAKSSTLQTRKQPPSQLGDQRILFSQRPSWWPFEMSPGTLAFAIIWPFIAQWLVHLYYQRRRRKLN

>XP_005608025_GCP60_Equus_caballus

MHRLRYDQTAFEEQRLTLWSKATGKHWPGTLPAGFQAILSEKDGKAFHPTYEEKLKLVALHKQVLLGPYNPDTCPEVGFFDVLGNDRRREWAALGNMSKEDAMVEFVKLLNRCCHLFSTYVASHKIEKEEQEKKRREEEERRRREEEERERLQKEEEKRRREEEERLRREEEERRRIEEERLRLEQQKQQIMAALNSQTAVQFQQYAAQQYPGNYEQQQILIRQLQEQHYQQYMQQLYQVQLAQQQAALQKQQEVAVAGASLPASSKVNAAAPSDILSVNGQAKTHTDNSEKDLEPEAAEEALENGPKESLPVIAAPSMWTRPQIKDFKEKIRQDADSVITVGRGEVVTVRVPTHEEGSYLFWEFATDNYDIGFGVYFEWTDSPNTAVSVHVSESSDDDEDEEGRTMENISSEEKAKKNANKPLLDEIVPVYRRDCHEEVYAGSHQYPGRGVYLLKFDNSYSLWRSKSVYYRVYYTR

>XP_005513848_ACBD5_Columba_livia

MMLKFYSFYKQATQGPCNIPRPGFWDPIGRYKWDAWSALGDMSKEEAMIAYVEEMKKILESMPMTDKVEELLQVIGPFYEIVEDKKNRGSDLTSVQMEKVSKYLEDLSNVMNSTPNIKAVNGKAESSDSGAESEEEGLREEEEKELQLNGSDYKSVKPELAAVKDLESVVTNGCCKDGFIPDLQNDIQTKSALNGLNPEEEIKKTEPSPEVANNSAPQGANEENTEEASATQHLTSDSDSEVYCDSMEQLGLEEPLEIITSPKGSLKHSSHFLDVDHSLLLENTDFPRHACMTYGNTVEEAAQEQGEVKCGGEDGKASNGGPHKEKKGGEKVDFYNVRRGRGHRLQPVGDGSQGGQMGGGGDGERWGSDRGPRGSLNEQIAVVLMRLQEDMQNVLQRLHMLETVTASQAKSATLQSNDHPASSVKKPSWWPFEISPGILAFAVVWPFIAQWLVHVYLQRKRRKLN

>XP_005508306_ACBD4_Columba_livia

MAPAATTSCGVTSARSAGPRAPSEGRYGHIMQKHMSVRMYGYRHTGSYRPSYEEMLRFYSYYKQATAGRCQGPRPGFWDPIGRYKWDAWHSLGRMSKEEAMAAYVAEMKKVAQKIIDTVPMDETTEEMFKYFEPLYEVIHDMPRPPEAFFKRKGGSREPTAEPGQDEPASSPALEHPEDAVSVEQDEQHAPDPCLAGGEHDAGSVPKLSLELPPELEAHVVSTIRALQDDMQRVLERLSELETLTTGQGDTAGTDPGQPHALQETSPWPLAVSPRTLLFLLTWPFVTQWLLRRWQGRKR

>XP_005505169_ACBP_Columba_livia

MSEAAFQKAAEEVKQLKSQPTDQEMLDVYSHYKQATVGDVNTDRPGMLDFKGKAKWDAWNALKGMSKEDAMKAYIAKVEELKGKYGI

>XP_005505011_ACBD7_Columba_livia

MALQADFDHAAGDVRKLKTTPTDEELKELYGLYKQATIGDINIECPGMLDLKGKAKWEAWNLKKGLSKEDAMKAYISKANALVEKYGI

>XP_005506757_ECI2_Columba_livia

MTAATMQVSQKDFEKAQEQLKLLKNDPGNETKLKLYALFKQATEGPCNSPKPGMLDFVKKAKWDAWNSLGNLSQDDAREKYTELVSSLVSAESAGQHKDASPDENRLGGYETIIVTTKNNITKIMFNRPDKKNAINHKMYREIIKALEEAGKDDSTIAVITGNGDYYSSGNDLSNFTGVKPTEMKKMAEDGAVLLKEFVSHFIDFPKPLIAVVNGPAVGICVTLLGLCDIVYASDRATFHCPFSQLGQSPEGCSSYLFPKIMGLAKANEVLLFNKKLTAADACVQGLVTEVFPDRTFQKEVWARLEAYASLPKNSLTLSKQLIRSIEKEKLHAVSSKECEVLKERWLSDECINAVVTFFQKKSKL

>XP_005501225_ACBD6_Columba_livia

MATHKMGSRYGSAPNAMQQAGGGSVTWPRWPEEEEQVGVLPVWPRSPLGTQSCHLLRTGVEEGDGLDSCAREGGKLSVRSRYQPKYKPPLGWRLAGTPGEPAASKEQLLYLYARYKQVKCGTCNTPKPGFFDFEGKQKWEAWKALGDTSPHQAMQEYVATVKKLDPSWNPQTTEKRGKESKTAFGGPVVSSLYQEETIREEDKNIFDYCRENNIDYVTKAIRSKKVDVNVTDEEGRGLLHWACDRGHKELVSVLLQHAADVNSQDGEGQTALHYAAACEFLDIVELLLKSGADPTLRDQEGCLPEEVTDCKAITSALQQHTAGGT

>XP_005507416_GCP60_Columba_livia

MAAVLSSDRLEVSVDGLTLSPNAELPKVESEQNGLDLLVTMTADTCLWWHIACLGVTSLACENEDILCIAWETMLFSPLNPFKKKDGKAFHPTYEEKLKLVALHKQVLLGPYNPDTCPEVGFFDVLGNDRRKEWAALGNMSKQKAMTEFVKLLNRCCHLFSTYVTSHKIEKEEQEKKRREEEERRRREEEERERLQKEEEKRRREEEERLRREEEERRRIEEERLRMEQQKQQIMAALNSQTAMQFQQYAAQQYPGNYEQQQILIRQLQEQHYQQYMQQLYQVQLAQQQAALQKQQEAVVAATGTPLTTASKGNAPAPGDTPSINGQASAHADSPEKEMDPEALEEALENGPKDSVPVIAAPSMWTRPQIKDFKEKIRQDADSVITVGRGEVVTVRVPTHEEGSYLFWEFATDNYDIGFGVYFEWTDSPNTAVSVHVSESSDDEDEEEENTSSEEKAKKNANKPQLDEIVPVYRRDCHEEVYAGSHQYPGRGVYLLKFDNSYSLWRSKTVYYRVYYTR

>XP_005233948_ACBD5_Falco_peregrinus

SLGSFQPTNEMMLKFYSFYKQATQGPCNIARPGFWDPIGRYKWDAWSALGDMSKEEAMIAYVEEMKKILESMPMTDKVEELLQVIGPFYEIVEDKKNRGSDLTSDLSNVMNSTSNIKAVNGKAESSDSGAESEEEGLREEEEKELQLNGKDYKNVKPESAAAKDLESIVSNGCYKDSFIPDMQNVLQTKSALNGLNQEEEIKKTEPSLEIANNSAHQGANEENTEEVSAAQHLTSDSDSEVYCDSMEQLGLEEPLEIITSAKGSLKHSSHFLDVDHSLLLENMDFPRRACMTYGNLQPGNTVEGPAQEQGEVKCGGEDGKASNGGPHKEKKSGEKADFYGVRRGRGHRLQPLGDGSQGGQMGSGGDGERWGSDRGPRGSLNEQIAVVLMRLQEDMQNVLQRLHMLEAVTASQAKSAALHSNYQPASSVKKPSWWPFEISPGILAFAVVWPFIAQWLVHVYLQRKRRKLN

>XP_005234983_ACBD4_Falco_peregrinus

MEEAGCGAQFRAAVQVIQGLPRSGSYRPSYEEMLRFYSYYKQATAGHCQGPRPGFWDPIGRYKWDAWHSLGRMSKEEAMAAYVAEMKKVAQKIIDTVPMDEMTEEMFRYFEPLYEVIHDMPRPPETFFKKKGGSQERAADPSQDNPESSPAAECPEDTLPREQQNGQHVPGLWLTGSGPELTQGLLAELDARVASTVQALQDDMRQVLERLSQLETLTSAQGDAAGTDTGQPLAPQAMSSWPLTVSPRTLLFLITWPFVTQWLLRHWQGRKR

>XP_005230860_ACBP_Falco_peregrinus

MTEAAFQKAAEEVKQLKSQPTDQEMLDVYSHYKQATVGDVNTDRPGMLDFKGKAKWDAWNALKGMSKEDAMKAYIAKVEELKGKYGI

>XP_005239124_ECI2_Falco_peregrinus

MTAATMQVSQKDFEKAQEQLKLLKKDPGNETKLKLYALFKQATEGPCNAPKPGMLDFVKKAKWDAWNSLGNLSQDNARQKHTELVSSLVSAESAGQKKDASPEESGHDGYETILVTTKNNITKIMFNRPDRKNAINHKMYREIIKALEEAGKDDSTIAVITGNGDYYSSGNDLNNFTNIQPGEMEKMAKDGAVLLKEFVGHFIDFPKPLIAVVNGPAIGICVTVLGLCDVVYASDRATFHSPFSQLGQSPEGCSSYLFPKIMGSAKANEILLFNKKLTAAEACAQGLVTEVFPDSTFQKEVWAKLEAYASLPKNSLAVSKQLLRSVEKEKLHAVNSKECEVLKERWLSDECVNAIVSFFQKKSKL

>XP_005228918_ACBD7_Falco_peregrinus

MALQADFDGAAEDVKKLKTRPTDEELKELYGFYKQATVGDINIECPGMLDLKGKAKWEAWNLKKGLSKEDAMNAYISQAKAMVEKYGI

>XP_005231698_ACBD6_Falco_peregrinus

MAQGRPQHLGEVTESSQAEGWHTFILILTPGEGEHIHALSLVFYVTKGFDLRYSTIIKEVTVKFGTCNTPKPSFFDFEGKQKWEAWKALGDTSPHQAMQEYVATVKKLDPSWNPQTTEKRGKESKTAFGGPVVSSLYQEETIREEDKNIFDYCRENNIDYVTKAIQSKKVDVNVTDEEGRALLHWACDRGHKELVSVLLQHAADVNSQDGEGQTALHYAAACEFLDIVELLLKSGADPTLRDQEGCLPEEVTDCKAIRSALQQHTAGQT

>XP_005241919_GCP60_Falco_peregrinus

MLAGGVVPSPQDANFKSGFAAPCAVTTPAGSCSREPSNTQGVDLQRLSWESPVARFASSKYLRFWKKKDGKAFHPTYEEKLKLVALHKQVLLGPYNPDTCPEVGFFDVLGNDRRKEWAALGNMSKQKAMTEFVKLLNRCCHLFSTYVTSHKIEKEEQEKKRREEEERRRREEEERERLQKEEEKRRREEEERLRREEEERRRIEEERLRMEQQKQQIMAALNSQTAIQFQQYAAQQYPGNYEQQQILIRQLQEQHYQQYMQQLYQVQLAQQQAALQKQQEAVVAATGTPLTTASKVNAPAPGDTPSINGQASAHADSPEKELDPEALEEALENGPKDSVPVIAAPSMWTRPQIKDFKEKIRQDADSVITVGRGEVVTVRVPTHEEGSYLFWEFATDNYDIGFGVYFEWTDSPNTAVSVHVSESSDDEDEEEENTSSEEKAKKNANKPQLDEIVPVYRRDCHEEVYAGSHQYPGRGVYLLKFDNSYSLWRSKTVYYRVYYTR

>jgi|Capca1|125310|Capitella_teleta

MAATKEKFDAAVKVIQSIPPNGSFVPSQSMMLKFYAYYKQATEGPCNIPRPSFWEVIAKAKWDAWNGLSDMPPHEAMANYVEELKKIIEAMPHTEHVQGFVEKLGDFYEAIDELPPGFDKKPKYWMNSNGDLPKVTEVDVNGSSDSGIDGEELGEILSAGELH

>jgi|Capca1|161103|Capitella_teleta

MATNKEKFDLAVDIVATLPKDGPIKPSNDLKLKFYGFYKQATIGKCNAPQPWAVDMINRSKWNAWNDLDDMSADEAMKKYAEAFYQSIIDNADDGADAELKAKLEKFRAMMK

>jgi|Capca1|176947|Capitella_teleta

MTVNMVMFRALSRSTAFVQQGMASVGRRMIRTSTASFFPAHDAEFSAAKERLNTLSEDPGNMTKLKIYALFKQATVGKCNTQRPGMVDFVGKAKWDAWDSLGDISQDEAQLKYISLIDELVAKDPSSSASEAESSTATSGNILVEVKDKMQIITLNRPNKRNALTLEMYDEIADVLESTGKDKNITLTVFTGSGSYYCSGNDLANFLNVDPADLSAMAVNGGKVLQRFVGSFIDHPRPLCAVINGPAVGITVTLLGLFDAVYATDKATFHTPFSLLGQSPEGCSSYVFPKLMGNKASEMLLFNKKLTAQEAFECNLVSEVYPDHAFQSEIWKKLAEIAQLPPNSLKTSKNLIRDAEREILHATNERECQLLVDRWQSEECAQAVMNFFQGK

>jgi|Capca1|183192|Capitella_teleta

MATDDEGPEFRERFQKACAFVKIVSADLDSEQLLYFYARYKQANDGPCSTPKPGFFDFKGKQKWEEWNKVKEMTAMDAMTEYMDALTFIAPDWELSVSDHKSSVQLFQFWSQVDSGKVKLCMGQAVSTMRSGDNGKLKDEQKTLFDWCVEGHVAKVEECISRDRKCLTQKDDNGMTLCHWTCDRGHLDMLRLLLRNNVDVNSRDADGQTPLHYAASCEHEEVIELLLSHSADINIRDEDGILPSEATENPSIASLLSSVT

>jgi|Capca1|176715|Capitella_teleta

MSEAFNKAAEDVKVLKSTPSNEELLELYGLFKQATVGDVNTDRPGMFDLKGKSKWDSWKGREGLSKADAEAKYIETANGLIAKYGKA

>jgi|Capca1|178819|Capitella_teleta

MADTEAEKVESGANSLRLNGNSHLEESQDGSKSFIDTWGFPLEDVYRLALRFYKDKEGSKALQLTYRDKLRMVALTKQVAHGRYRPDASPDVGFLDVVGNDRRQAWQGLGELSREQSMEEFVSTLDKLCSLFKAYTQAHKAEKEEHERKLREEEEQLKREEEEAMRRKQEEEINLQQEQESGKQRLQEEQIRQALNQQTSIQFKQYAEQQCPADEQQQQTLIAQLQEQHFQQYMQQVYQQQLIHQQQQYEQLQAMQATPKDLPPPMGGVDTSASVEFSSETAADLTDAVPNAAMPLVNGDAVPPQDTEENQEQNQPELNDLPPVAAASMWTRKDVRDFKDSIRKDPDSVIKVGSGETVTVRVPTHEDGTCLFWEFATDSYDIGFGVYFEWTIAPSNQVSVHVSESSDEEDLDEDEAAGAARAEEKGDIEKGGDSKRSNHPPTDEIIPVYRRDCHEEVYCGSHMYPGRGVYLLKFDNSYSLWRSKTLYYRVYYTR

>NP_031856_ACBP_MOUSE

MSQAEFDKAAEEVKRLKTQPTDEEMLFIYSHFKQATVGDVNTDRPGLLDLKGKAKWDSWNKLKGTSKESAMKTYVEKVDELKKKYGI

>sp|Q5XG73|ACBD5_MOUSE

MLFLAFHAGSWGSWCCCCCVITADRPWDRGRRWQLEMADTPSVYETRFEAAVKVIQSLPKNGSFQPTNEMMLKFYSFYKQATEGPCKLSRPGFWDPIGRYKWDAWSSLGDMTKEEAMIAYVEEMKKIIETMPMTEKVEELLHVIGPFYEIVEDKKSSKSSDLTSDLGNVLTSSNAKAVNGKAESSDSGAESEEEEAQEELKGAEQSGSDDKKTLKKSADKNLEIIVTNGYKGSFVQDIQSDIHTDSSRSTRSSEDEKPGDESSQQTGHTIVCAHQDRNEDPSEDASGIHHLTSDSDSEVYCDSMEQFGQEEYYLGGDPTQHLESSGFCEDAQQSPGNGSIGKMWMVAVKGKGEVKHGGEDGRSSSGAPHRETRGGESEDFSSVRRGRGNRIPHLSEGPKGRQVGSGGDGERWGSDRGSRGSLNEQIALVLIRLQEDMQNVLQRLHKLETLTASQAKLSLQTSNQPSSQRPAWWPFEMSPGALAFAIIWPFIAQWLAHLYYQRRRRKLN

>NP_001103801_ECI2_MOUSE

MAAVTWSRARCWCPSVLQVFRLQVAKLHLGRPTMRASQQDFENALNQVKLLKKDPGNEVKLRLYALYKQATEGPCNMPKPGMLDFVNKAKWDAWNALGSLPKETARQNYVDLVSSLSSSSEAPSQGKRGADEKARESKDILVTSEDGITKITFNRPTKKNAISFQMYRDIILALKNASTDNTVMAVFTGTGDYYCSGNDLTNFTSATGGIEEAASNGAVLLRDFVNSFIDFPKPLVAVVNGPAVGISVTLLGLFDAVFASDRATFHTPFSQLGQSPEACSSYTFPKMMGSAKAAEMLLFGKKLTAREAWAQGLVTEVFPESTFETEVWTRLKTYAKLPPNAMRISKELIRKNEKEKLYAVNAEECTTLQARWLSEECMNAIMSFVSRKPKL

>NP_082526_ACBD6_MOUSE

MATPFLPSGATTGDSGGELSSGDDSGDMESFQTPEAEGTRSLAELFEKAAAHVQGLVQVASREQLLYLYARFKQVKVGNCNTPKPNFFDFEGKQKWEAWKALGDSSPSQAMQEYIAAVKKLDPGWNPQVSEKKGKEGSSGFGGPVVSSLYHEETIREEDKNIFDYCRENNIDHIAKAIKSKAADVNMTDEEGRALLHWACDRGHKELVKVLLQYEAGINCQDNEGQTALHYAAACEFLDIVELLLQSGADPTLRDQDGCLPEEVTGCKAVSLLLQRHRASKA

>NP_067269_ACBP-like5_MOUSE

MSQVEFEMACASLKQLKGPVSDQEKLLVYSFYKQATQGDCNIPVPPATDVRAKAKYEAWMVNKGMSKMDAMRIYIAKVEELKKKEPC

>sp|Q8BMP6|GCP60_MOUSE

MAAQLNVEQLEVSLDGLTLSPDSEERPGAEGAPPQTPPSSAPGNGLGSGASGQQREPGEAAAEGAAEEARRMEQHWGFGLEELYGLALRFYKIKDGKAFHPTYEEKLKFVALHKQVLLGPYNPDTSPEVGFFDVLGNDRRREWAALGNMSKEDAMVEFVKLLNKCCPLLSAYVASHRIEKEEEEKRRKAEEERRQREEEERERLQKEEEKRKREKEDRLRREEEERRRIEEERLRLEQQKQQIMAALNSQTAVQFQQYAAQQYPGNYEQQQILIRQLQEQHYQQYMQQLYQVQLAQQQAALQKQQEVVMAGASLPASSKVNTAGASDTLSVNGQAKTHTENSEKVLEPEAAEEALENGPKDSLPVIAAPSMWTRPQIKDFKEKIRQDADSVITVRRGEVVTVRVPTHEEGSYLFWEFATDSYDIGFGVYFEWTDSPNAAVSVHVSESSDEEEEEEENVTCEEKAKKNANKPLLDEIVPVYRRDCHEEVYAGSHQYPGRGVYLLKFDNSYSLWRSKSVYYRVYYTR

>sp|Q9D258|ACBD7_MOUSE

MSLQADFDQAAQDVRKLKSRPEDEELKELYGLYKQSVIGDINIACPAMLDLKGKAKCEAWNLQKGLSKEDAMCAYISKARELIEKYGI

>NP_080264_ACBD4_MOUSE

MGTEKEEPDCQKQFQAAVSVIQNLPKNGSYRPSYEEMLRFYSYYKQATMGPCLVPRPGFWDPIGRYKWDAWNRLGKMSREEAMSAYISEMKLVAQKVIDTVPLGEVAEDMFGYFEPLYQVIPDMPRPPETFLRRATGWKEPLVQREDQAAPEPSCVPKEPVPPSPESRPPRDLDLEVFCDSVEQLEPELVRLPVLSPVAAEPELCHLPPGIWDSSAQQVWAEQKEAAGRELTTRSSPESTGEKEGLGGGLIGPQELDTWLVGTVRAMQESMKDVHRRLQSLESKPQPLKQRSPRTRPWPLGLSVPTLLFFILWPFVVQWLFRQFRTQKR

>XP_003410752_ACBD5_Loxodonta_africana

MLFLSFHAGSWESWCCCCLIPSDRSWSRGRRWGLEMADTRSVHETRFEAAVKVIQSLPKNGSFQPTNEMMLKFYSFYKQATEGPCKMSRPGFWDPIGRYKWDAWSSLGDMTKEEAMIAYVEEMKKILETMPMTEKVEELLHVIGPFYEIVEDKKSGRSSDLTSDLGNVLTTTNAKTMNGKAESSDSGAESEEEEAREEVKGAEQSDNDKKMMKKSADHNNLEIIVTNGYDKDSFVPDVQNDLHASSSLNGKSTEEVKPVEQNFEETVKTTICIHQDTTDDHVEDVSGIQHLTSDSDSEVYCDSMEQFGQEESLDNFSSNSGPFRCHLNADPNQRLESSGFPEDVQVSPANGNTGDMQVVAVEGKGEVKRGGEDGRSNSGAPPHEKRGGENEELSNVRRGRGHRMQHLGEGAKGRQVGSGGDGERWGSDRGSRGGLNEQIALVLMRLQEDMQHVLQRLHKLETLAASQAKSSALQTSSQPSSPRPCWWPFEMSPGTLTFAIIWPFIAQWLVHIYYQRRRSFKNNSSMAEEDTLCEAEHERPHVAVSSLQFAFLWKPLAWSHNCPPPL

>XP_003414344_ACBD4_Loxodonta_africana

MGTENEGPEPDCQKQFQAAVSVIQSLPKNGSYRPSYEEMLRFYSYYKQATMGPCLIPRPGFWDPIGRYKWDAWNSLGKMSREEAMSAYIAEMKVVAQKVIDTVPLGEVAEDMFGYFKPLYQVIPDMPRPPETFLRRVTGQKEQILNSNVGANLEPPYPAKEPAPPNPESQQPRDLDSEVFCDSLEQLEPELVWAEQRGAPGGEPDTRTSPKSPREQEGLGGSLLGPQEFDTWLVGTVRALQQSMQDVQGRLQSLESMPCPHKQSPQPPPSARPWPFRLSGPTLLFFLLWPFVVHWLFRQFRIQKR

>XP_003417885_ECI2_Loxodonta_africana

MEYVKAAYRNSQRETDHPETTPRPSPTCCSASSASHQSEPRGTGFGSLPLGFALPTLASSQSAISRQGPAPVSGPAESRMVPCVARFASARQSRANSLHLQLAMAWVVARLAWRCPPRYAGSPLQVSSFLVQQMHMSQTAMRASHKDFETAVNQMKLLKKDPGNEMKLKLYALYKQATEGPCNMPKPGVLDLISKAKWDSWNALGSLPKETARQNYVELVSSLSSSESSSQGKPGADSKQPGYETLVVTSEDGITKIMLNRPTKKNAITTQMYQDIVLALEAAGKDDSTITVLTGNGDYYCSGNDLNNFTDIPPGGIEEQAKNNAILLRNFVGCFIDFPKPLVAAVNGPAVGISVTLLGLFDIVYASDRATFHTPFTHLGQSPEGCSSYTFPKIMGPAKAAEMLIFGKKLTAREACAQGLVTEVFPDSTFQKEVWTRLKAYSKLPPNAMRTSKQLIRNVEREKLHATNSEEISALQGRWRSEECTNAVMNFLSRKVKL

>XP_003411021_ACBD6_Loxodonta_africana

MASPFLPTGATMGGLGGELSSGDDSGDVGGSLQSPETEASRSLTELFEKAAAHLQGLIQVASREQLLYLYARYKQVTVGSCNTPKPSFFDFEGKQKWEAWKALGDSSPSQAMQEYIAVVKKLDPGWNPQTPVKKGKEANTGFGGPVVSSLYHEEIIREEDKNIFDYCRENNIGHITKAIKSKNVDVNMKDEEGRALLHWACDRGHKELATVLLHYRADINCQDNEGQTALHYAAACEFLDIVELLLQSGADPTLRDQDGCLPEEVTGCKAVSLMLQQHTASKA

>XP_003416725_ACBP_Loxodonta_africana

MSQAEFDKAAEEVKHLKTKPDDNEMLFIYSRYKQATVGDVNTERPGMLDFKGKAKWDAWNELKGTSKEDAMKAYVDKVEELKKKYGI

>XP_003410775_ACBD7_Loxodonta_africana

MTLCIKQLTPPRKTFGKADFDRAAEDVRKLKARPDDENLKELYGLYKQSVIGDIDIERPGTLDLKGKAKWEAWNLQKGLLKEDAMRAYISKAKELIEKDGI

>XP_003417017_ACBP-L5_Loxodonta africana

MGQVEFELACAAVKELKGPVSDQEKLLVYSFYKQATQGDCNIPAPPSTDVKAKAKWEAWNENKGMTKLDAMRIYIAKVEELKKKEGG

>XP_003410977_GCP60_Loxodonta_africana

MAAVLNAERLEVSVDGLTLSPDPEERPGAEGAPLLPPPLPPPSPPGSGRGPGAAGEQPEPGETAAGGAAEEARRLELHWGFGLEELYGLALRFFKEKDGKAFHPTYEEKLKLVALHKQVLMGPYNPDTCPEVGFFDVLGNDRRREWAALGNMSKEDAMVEFVKLLNRCCHLFSTYVASHKIEKEEQEKKRKEEEERKRREEEERKERLQKEEEKRRKEEERGFRREEEERRQIEEERLRLEQQKQQIMAALNSQTAVQFQQYAAQQYPGNYEQQQILIRQLQEQHYQQYMQQLYQVQLAQQQAALQKQQEVAVAGASVPLSSKVNAAAPGDRMSINGQAKPHTDSSKKELEPEAAEEAMENGPKDSLPVIAAPSMWTRPQIKDFKEKIRQDADSVITVGRGEVVTVRVPTHEEGSYLFWEFATDNYDIGFGVYFEWTDSPNTAVSVHVSESSDDDEEEEENINSEEKAKKNANKPLLDEIVPVYRRDCHEEVYAGSHQYPGRGVYLLKFDNSYSLWRSKSVYYRVYYTR

>XP_005617034_ACBD5_Canis_lupus

MLFLSFHAGSWGSWCCCCCLIPADRPWDRERGRRWRLEMADTRSVHETRFEAAVKVIQSLPKNGSFQPTNEMMLKFYSFYKQATEGPCKLARPGFWDPIGRYKWDAWSSLGDMTKEEAMIAYVEEMKKILETMPMTEKVEELLHVIGPFYEIVEDKKCGRTSDLTSVRLEKISKYLEDLGNVLTSTPNAKTVNGKAESSDSGAESEEEEAQEEVKGAEQSDNDKKMMKKSADHKNLEVIVTNTYDKDHSAQEIQNDIHASSSLNGRSAEEIKAVDENLEKTAICIHQDINDDHVEDVSGIQHLTSDSDSEVYCDSMEQFGQEESLDSFTLNNGPFRYYLGGDPNKPLENSGFPEDGSPGNGNTGDMQVVAVEGKGEVKHGGEDGRSNSGAPHRERRGGENEFSNVRRGRGHRMQHLSEGSKGRQVGSGGDGERWGSDRGSWGSLNEQIALVLMRLQEDMQNVLQRLHKLETLTASQAKSSTLQTSNQPPSQRPSWWPFEMSPGALTFAIIWPFIAQWLVHLYYQRRRRKLN

>XP_548051_ACBD4_Canis_lupus

MGTKNESPEPDYQKQFQAAVSVIQNLPKNGSYRPSYEEMLRFYSYYKQATMGPCLVPRPGFWDPIGRYKWDAWNSLGKMSREEAMSAYITEMKLVAQKVIDTVPLGAVAEDMFAHFEPLYRVIPDMPRPPETFLRRVTGWREQVLNGDVGATPEPPCLPREPEPPSPESQPPRDLDSEVFCDSLEQLEPELVWTEQKGAPGGEPDTRNSSMLPAEKEGSLMGPQELDTWLVGTVRALQESMRDVQGRLQSLESMPSLPEQRTPPRARPWPLRLSGPTLLFFLLWPFIVQWLFRTFRTQKR

>XP_535873_ECI2_Canis_lupus

MQALSLHMSKTAMGASQKDFESAMNQVKLLKKDPGNEVKLKLYALYKQTTEGPCNTPRPGVFDLINKAKWDAWNALGNLPKETARQNYVDLVSDLSSSDSSSQVKPEADRKQPGYETLVVTSEDSITKIMMNRPAKKNALTIQMYREIMLALEAASKDDSTIIVLTGNGDYYSSGNDLMNFMNIPPGEMEKEAKNGAILLRDFVGCFIDFPKPLVAVINGPAIGISVTILGLFDLVYASDRATFHTPFTHLGQSPEGCSSYTFPKIMGQAKAAEMLMFGKKLTAREACAQGLVTEVFPDSTFQKEVWTRLKAYSKLPRNTLHISKQSIRNLEKEKLHAVNAEENSVLQERWLSDECINAVMSFLSRKAKL

>sp|Q9TQX6_ACBP_Canis_lupus

MSQAEFDKAAEDVKHLKTKPADDEMLYIYSHYKQATVGDINTERPGLLDLRGKAKWDAWNQLKGTSKEDAMKAYVNKVEDLKKKYGI

>XP_850165_ACBP-like_Canis_lupus

MSTVTKALSLWAGPVNMMGYHSQDFIILYGKVPKPHNPASMSQAEFDKATEDVKHLETKPADDEMLFIYSHYKQATIGDVNTEWPGLLDLRGKAKWDAWNHLKRTSKENAMKAYINKVEDLKKKYGM

>XP_537152_ACBD6_Canis_lupus

MASPFLTAGATTGDSGGELSSGDDSGDVESRQSPETEASGSLAELFEQAAAHLQGLVQVASREQLLYLYARYKQVKVGNCNTPKPSFFDFEGKQKWEAWKALGDSSPSQAMQEYIAVVKKLDPGWNPQSPEKKGKEANTGFGGPVVSSLYHEEIIREEDKNIFDYCRENNIDHVTKVIRSKNVDVNMKDEEGRTLLHWACDRGHKELVTVLLRYKADINCQDDEGQTALHYAAACEFLDIVELLLQSGADPTLRDQDGCLPEEVTGCKAVCLVLQQHTTGKA

>XP_848899_ACBD7_Canis_lupus

MSLQADFNKIAEDVRKLKARPDDEELKELYGLYKQSVVGDINIECPGMLDLKGKAKWEAWNLQKGLSKEDAMSAYIPKAKELIEKYGI

>XP_537760_ACBP-L5_Canis_lupus

MCQVEFEMACAAIKQLKGPVSDQEKLLVYSFYKQATQGDCNIPAPPATDVKAKAKWEAWNQNKGMSKMDAMRIYVAKVEELKKKDTG

>XP_854705_GCP60_Canis_lupus

MLQDDGGQAAARLSPVKALSADPHLILSMLEATPHLLHAWPWASGPTRGSLIVEAFHGTLKGSDERQGLDERFLGPYSVSRSFEALEEAGGSGELTGTCEARRRSVMMLTVLPGADSPRASYFVLALQVPGEATAAVLPIDGPNSLPHLGGGHRAAPAAGLESSPAEKDGKAFHPTYEEKLKLVALHKQVLMGPYNPDTCPEVGFFDVLGNDRRREWAALGNMSKEDAMVEFVKLLNRCCHLFSTYVASHKIEKEEQEKKRKEEEERRRREEEERERLQKEEEKRRREEEERLRREEEERRRIEEERLRLEQQKQQIMAALNSQTAVQFQQYAAQQYPGNFEQQQLLIRQLQEQHYQQYMQQLYQVQLAQQQAALQKQQEVAVAGASLPTSSKMNAVAPGDRMSVNGQAKTHTDSSEKELEPEAAEEAVENGPKESLPVIAAPSMWTRPQIKDFKEKIRQDADSVITVGRGEVVTVRVPTHEEGSYLFWEFATDNYDIGFGVYFEWTDSPNTAVSVHVSESSDDDEEEEENVSNEEKAKKNATKPLLDEIVPVYRRDCHEEVYAGSHQYPGRGVYLLKFDNSYSLWRSKSVYYRVYYTR

>XP_003771913_ACBD5_Sarcophilus_harrisii

MADTRSVHETRFEAAVKVIQSLPKNGSFQPTNEMMLKFYSFYKQATEGPCNISRPGFWDPIGRYKWDAWSSLGNMTKEEAMIAYVEEMKKILETMPMTEKVEELLHVIGPFYEIVEDKKSSRSSDLTSVRLEKVTKYLEDLGNVLSSTPKVKTVNGKAESSDSGAESEEEENQEEVKEAKQSKNDTKEAIPEFTDNKELEIVFTNGYDQDNILQDIPNDIHASSSLLNGSSTEEVKNGDQNLKQTGQTTVSIHQGICDDHVEDISGIQHLTSDSDSEVYCDSMEQFGQEESLEIFSSNKGPFRYSSHYLGDHNQLLENSGLSEEVCIASGNLPVGDIAIEGKGEVKHGGEDGRPSSGAPQREKRGGEKEEFSGVRRGRGHRMQPLGDGIHSGHMGSGGDGERWGSDRGSGGSLNEQIAVALMRMQEDMKNILQRLHTLETLAASQSDHNVCXLVTSLFSFSQRPSWWPFEMSPSALTFAIVWPFIAQWLVHLYYQRRRR

>XP_003768479_ACBD4_Sarcophilus_harrisii

MGTERGSSEPGCREQFHAAVTVIQSLPKNGSYRPSYEEMLRFYSYYKQATQGPCRVPRPGFWDPIGRYKWDAWHSLGRMSQEEAMAAYITEMKVVAQKVIDTVPLGEVDDGMFDYFVPLYEMIPDMPQPPETFLKKLTAQKEKMPNGDVRNALEPLPPPEESVPQTSEAQPPSVPSIRGRASMDLDAEVFCDSLEQLEPEQGEWLQKELKEALEGETDISSDHLLPRQREMVGGAPQGPQEFEAWLASTVRALQESMQDVQGRLRSLENLPHLTQMPPSQQGRRALELSLPTILLLFLWPFVVQWCYRQFRRQKR

>XP_003763843_ACBP_Sarcophilus_harrisii

MSQAEFERAAEEVKNLKAKPNDEEMLFIYSHYKQATVGDINTERPGMMDFRGKAKWDSWNSLKGKSKEEAMKAYIAKVEELKKKYGI

>XP_003760261_ECI2_Sarcophilus_harrisii

MPAATLRACSRPPLPGSLWKRQLPAVGFPALQLHMTNMALRASQEDFERAKGQIKLLKEDPGNEVKLKLYALFKQATEGPCTSPKPGMLDFVNKAKWDAWNALGSLSKDTARQNYVDLVASLVSSQSLSQETSIDKKSEYETLVVTREDNITKIMLNRPAKKNAINNKMYNEIMLALEAADKDDSSLTVLTGNGDYYSSGNDLSEAAKVPPDDIEKKIKESFVLLRTFVDHFIDFSKPLVAVVNGPAVGISVTLLGLCDIVYATDRATFHTPFIQLGQSPEACSSYTFPKIMGPIKAAEMLIFGRKLTAQEAYAQGLITEVFPDSTFQREVWTRLKAYAQLPPKAMMFSKQLIRSFEKETLHKVNFEECTLLCERWVSDEFMNAVVNFINRKSKL

>XP_003771952_ACBD7_Sarcophilus_harrisii

MSLQNDFHNAAEDVRKLKTRPNDEELKDLYGLYKQSIVGDIDIECPGMLDLKGKAKWEAWNLQKGLSKEDAMSAYISKAKELIEKYGI

>XP_003767802_GCP60_Sarcophilus_harrisii

MAGVLSAERLEVSIDGLTLSPDPEERPSAEGAPPLPAPPGGSRGPGPGGAPSPGPGDSGGXDSLCLEEFWGFGLEELYTLALRFFKDKDGKAFHPTYEEKLKLVALYKQVLWGPCNPDTYPEVGFFDVLGNDRRKEWAALGNLSKEDAMLEFVKLLNRCCYLFSTYVTSHKIEKQEQEKRRKEEEEQRRREKEEKERLQREEEKRIQEESERLKREAEERRRIEEEKLRLEQQKQQIMAALNSQTAVQFQQYAAQQYPGNYEQQQILIRQLQEQHYQQYMQQLYQVQLSQQQAALQKQQEAATTGASLPAASKVNAATPGEMLSVNGQAKPHPENSAKELEPEISEEVLENGPKESVPVIAAPSMWTRPQIRDFKEKIRQDADSVITVGRGEVVTVRVPTHEEGSYLFWEFATDNYDIGFGVFFEWTESPNTNVSVHVSESSDDDEEDEENSEEKAKRKSNKPQLDEIVPVYRRDCHEEVYAGSHQYPGKGVYLLKFDNSYSLWRSKVVYYRVYYTR

>XP_003770143_ACBP-L_Sarcophilus_harrisii

MAQVEFELACAAVKQLTGPVTDEEKLVVYSYYKQATIGDVNIPCPEVTDFKAKAKWEAWNCRKGMSKLDAMRVYVSKVEELKSRQC

>XP_003767530_ACBD6_Sarcophilus_harrisii

VKVGNCNTPKPSFFDFEGKQKWEAWKALGDSSPSQAMQEYIAMVKKLDPNWNPQIPEKKGKEVNTGFGGAVVSSLYQEETIREEDKNIFDYCRENNIDHITKAIRSKKVDVNMKDEEGKALLHWACCRGGLHKLTIKVLFSYKPGLHCRDNEGQTALHYASACEFLDIVELLLKCGADPTLRDQEGCLPEEVTGCKAVSLVLQQHTAGKA

>XP_001367254_ACBD5_Monodelphis_domestica

MCVGAWGGPGMRGWCLPLVERRRPSHPVGHLLCQCSLPLGEPWSLAIGAFRPRAGPGAGGGEEEGRTSRAGQGWRLEMADTRSVHETRFEAAVKVIQSLPKNGSFQPTNEMMLKFYSFYKQATEGPCNISRPGFWDPIGRYKWDAWSSLGDMTKEEAMIAYVEEMKKILETMPMTEKVEELLHVIGPFYEIVEDKKSSRSSDLTSDLGNVLSSTPNVKTVNGKAESSDSGAESEEEEAQEEVKEAKQSKSGKKVMPEPGDDKDLEIVFTNGYDKDSLLEDVPDDIHANSSLNATSTEEVKNVDPNLKQSGQTPVGIYPGTYDDHIEDISGIQHLTSDSDSEVYCDSMEQFGQEESSEILSSNKGPFRYSSNYVGGDHSQLLENSGLPEEVCIASGNSSLQMIAVEGKGEVKHGGEDGRTNSGAPQREKRGGEKEEFSSVRRGRGHRMQHLGDGIPSGHMGSGGDGERWGSDRGAGGSLNEQIAVALMRLQEDMKNVLQRLHTLETLAASQAKSSALQTDNRPPSLKPSWWPFEMSPCAFTFAIVWPFIAQWLVHLYYQRRRRHLEYCHVEKLALNVTKLCCKAKTIDAVTQPQIDFRSGLKADVVRLLKRFLSAEVIALTQMARIQIFGAVCVYVCLPTCKALFLFP

>XP_001375520_ACBD4_Monodelphis_domestica

MWTERDRPEPDHQKQFHAAVSVIQSLPKNGSYRASYEEMLRFYSYYKQATQGPCHMPRPGFWDPIGRYKWDAWHRLGRMSQKEAMAAYITEMKVVAQKVIDTVPLSEVDDGMFNYFVPLYEMIPDMPQPPEVFLKKLTARKEKIPNGDMKMAFDPLPPPEEPAPQTSGAQLPRVPSIHGRASMDLDTEIFCDSLEQLEPEQGEWLQEELKESLVGEPDVSSDHLLSREREMLRTPQGPREFEAWLASTVQALQESMQDVQGRLRCLESLPRPAKSPQPQQGPWALELSTPTLLLLLLWPFVVQWIYRQFRRQKR

>XP_001368082_ECI2_Monodelphis_domestica

MISAISQLARWRPPIPGSLWKRQLPAVSFPALQLHTTSMAMRVSQEDFEKAKEQVSLLKKDPGNEVKLKLYALFKQATEGPCTTPKPGMLDFVKKAKWDAWNALGSLPKDAARQNYVDLVSSLVSSESLSQKKTSFNDTQSEYKTLIVTKEDNITKIMLNRPSQKNAINIQMYKDITLALEAAEKDDSSLTVITGNGEYYSSGNDLSEPLKIPPDEIQKKLEENMQILRTFIDHFIDFSKPLVALVNGPAIGISVTLLGLFDIVYATDRATFHTPFIHLGLCPEACSSFTFPKIMGSVKAAEILIFGRKLTAQEAYARGLVTEVFPESTFQKEVWTRLKAYSQLPPVSMKKSKQLIRSFEKETLHRVNLEECNLLQTRFTSDECLNAIVNFLNRKSKL

>XP_001367923_ACBP_Monodelphis_domestica

MSQAEFDKAAAEVKVLKSKPNDDEMLYIYSHFKQATVGDVNTARPGITDFKGKAKWDAWNSLKGKSKEDAIKAYIEKVEELKKKYGI

>XP_001374542_ACBD6_Monodelphis_domestica

MELARGPWVSPYPASGATTGDSGGELSSGDESGDVADSPPGPESEAPKSLVELFEKAAAHLQGLVQVASREQLLYLYARYKQVKVGNCNTPKPSFFDFEGKQKWEAWKALGDSSPSQAMQEYIALVKKLDPNWNPQMPEKKGKEVNAGFGGPVVSSLYQEETIREEDKNIFDYCRENNIEHITKAIRSKKVDVNMKDEEGRALLHWACDRGHKELVTILLQYTADINCQDNEGQTALHYASACEFLDIVELLLKCGADPTLRDQEGCLPEEVTGCKAVSLVLQQHTAGKA

>XP_003340221_GCP60_Monodelphis domestica

MAGVLSAERLEVSIDGLTLSPDPEERPSADGAPLLSPSLVGSGPEAASERRREAGEKEAAAKGPARAAAPASSLSLEEFWGFGLEELYGLALRFFKDKDGKAFHPTYEEKLKLVALYKQVLWGPCNPDTYPEVGFFDVLGNDRRKEWAALGNLSKEDAMLEFVKLLNRCCYLFSTYVTSHKIEKQEQEKRRKEEEEQRRREKEERERLQREEEKRIQEESERLKREAEEKRRIEEEKLRLEQQKQQIMAALNSQTAVQFQQYAAQQYPGNYEQQQILIRQLQEQHYQQYMQQLYQVQLSQQQAALQKQQEEAAAGASLPAASKVNATAPGEILSVNGQAKPHPDSSVKELEPEISEEALENGPKDSVPVIAAPSMWTRPQIKDFKEKIRQDADSVITVGRGEVVTVRVPTHEEGSYLFWEFATDNYDIGFGVYFEWTDSPNTSVSVHVSESSDDDEEDEENSEEKAKRKANKAQLDEIVPVYRRDCHEEVYAGSHQYPGKGVYLLKFDNSYSLWRSKIVYYRVYYTR

>XP_001370361_ACBP-L5_Monodelphis_domestica

MAQVEFELACATVKQLTGPVSDEEKLLVYSYYKQATVGDINIPCPEVTDFKAKAKWEAWNCRKGMSKLDAMRIYVSKVEELKKKQSS

>XP_001506917_ACBD5_Ornithorhynchus_anatinus

MADTTSVHETRFQAAVKVIQSLPKNGSFQPTNEMMLKFYSFYKQATEGPCNISRPGFWDPIGRYKWDAWSSLGDMSKEEAMIAYVEEMKKILESMPMTEKVEELLRVIGPFYEIVEDKKTNRSSKLTSELGNVLTSTPSAKTVNGKAESSDSGAESEEEEALEEVKQVKERENELKVEQESIANKDLEDVFTNGYDKDSLIDASSSINGTSTDEEVKKNQNLEQTGIATVWPQQDSNDDHVEDVSGIQHLTSDSDSEVFCDSMEQFGQEESLELLTSNRGALRYSPHCVAVDHSQLLENSGFPEEDRVSSENFRVEDMQERAGEGKGEVKRGGEDGRTNSGAASHREKRAGEKEEGSGFRRGRGHRMQHLSDGTQGGQMGSGGDGERWGADRGLRGSLNEQIAIVLMRLQEDMQNVLQRLHTLETLTASQAKSSALQSANYPPSSKPSWWPFEISPSALAFAIIWPFIAQWLVHLYYQRRRRKLN

>XP_001508493_ECI2_Ornithorhynchus_anatinus

MHTSGTAMQVSQEDFEKAKDQVKLLKKDPGSEVKLKLYALFKQATEGSCNTPKPGMLDFVNKAKWDAWRALGSLPKENARQNYVDLVSSLVSSESPQQAKQPSGGEHQQYETLRVTTENNITKIILNRPERKNAISTQMYEELILALEAAGKDDSAIAVLTGNGDYYSSGNDLNNFMNVSPDKIEQKAKDGAVLLKNFVGHFIDFPKPLIAVVNGPAVGIPVTLLGLFDIVYATDRAKEMLIFGKKLTAQEAWANGLVTEVFPDSTFKEEVWARLKAFASLPRNAMRISKQLMRSVDREKLHAVNSQECQILEERWLSDECMTAIMNFFSKSSKL

>XP_001515833_ACBD6_Ornithorhynchus_anatinus

MAAFEFGPGKQPRRDAGVKVKVGNCNVPKPGFFDFEGKQKWEAWKALGEASPKQAMQEYVAAVRKLDPTWNPQAPEKKEKEGKVGFGGPVISSLYQEETIREEDKNIFDYCRENNIDHVTKAIKSKKVDVNIKDEEGRALLHWACDRGHKELVSILLQFTADINSQDNEGQTALHYASACEFLDIVELLLQAGADPTLRDQEGCLPEEVTECKAVSSVLQQHTAGRA

>XP_001512836_GCP60_Ornithorhynchus_anatinus

MDLFGEKDGKAFHPTYEEKLKLVALHKQVLLGPYNPDTCPEVGFFDVLGNDRRKEWAALGNLAKEDAMTEFVKLLNRCCHLFSTYVTSHKIEKEEQEKKRKEEEERRRQEEEERERLQKEEEKRRREEEERQRREEEERRRIEEERFRLEQQKQQIMAALNSQTAVQFQQYAAQQYPGNYEQQQILIRQLQEQHYQQYMQQLYQVQLAQQQAALQKQQQEVAAAPGATLVSVTKGAAPGQGELPSINGQAPAHSDSSEKEPEPEAAEEVLENGPKEPVPVIAAPSMWTRPQIKDFKEKIRQDADSVITVGRGEVVTVRVPTHEEGSYLFWEFATDNYDIGFGVYFEWTDSPNTAVSVHVSESSDEEDEEEENTSSEEKAKKNANKPQLDEIVPVYRRDCHEEVYAGSHQYPGRGVYLLKFDNSYSLWRSKTVYYRVYYTR

>XP_003222145_ACBD5_Anolis_carolinensis

MAEMEAESSTSLSVHETRFEAAVKVIQSLPKNGSFQPTNEMMLKFYSFYKQATQGPCNSQRPGFWDPIGRYKWDAWSALGDMPKEDAMIAYVEEMKKILESMPMTDKVEELLHVLGPFYEIVEDKKKRISDLIGVSGNIMMSVPAVNGKSESSDSGAESEEEPHQEEIIELQEIEKEEPESLEQDCSTDKELGNVVANGCCDDVPDINLHNGIQTKSALNGISVDEEIKKGAKYLELPVKSNYGSPHQGTDLKEDNTEEISGIQHLTSDSDSEIYCDSMEQFGQEEMLEINTSTKEFSKHPFHFSEGDQSLLETSGFLDLGNYKVEGINEAAVEGKGEVKCGGEDGKASDGGPHKEKKGGEKVDFYGIRRGRGQRMHPLGDSAQGGQMGSGGDGERWGSDRGPRGSLNEQIAVVLIRLQEDMQNVLQRLHALETLTASQARTATLHSNDQQTSPVKRPSWWPFEISPNTLAFAILWPFVAQWLVHVYLQRRRRKHN

>XP_003222574_ACBD4_Anolis_carolinensis

MGKKAEEPDCQKQFEAAVRVIQGLPKNGAYRPSYEEMLRFYSFYKQATVGQCQISRPGFWDPIGRYKWDAWNRLGKMSKEEAMAAYVAEMKKAAQKVIDTVPLDESSKDMFVYFESLYDVIDDMPRPPESFFRKKAGPGYMDNAIKSNQVTTDAENEVFCDSLEQVEPDQAKRLSAEPSLPLNNAQRVEVEDKNLQRGPGEESLIQSPEAIAGCPAGALGQREVDRQVSNTLQALQRDMKEVMERLSSLESWAALQV

>XP_003223610_ECI2_Anolis_carolinensis

MQFSQEDFEKAKDQLKLLEDDPGNEVKLKLYALYKQATEGPCKTPKPGMLDFVKKAKWDAWSSLGSLPQDSARQKYIELVSSLVSADSSMASGSKSDYQTLQVTTKDNITKIVLNRPKKKNAISTKMYNEIIEALEEAAKDDSVITVVTGNGDFYSSGNDMNNYINISPDDVEKKAKESAKMLKSFVGSFIDFPKPLIAVVNGPAVGIAVTLLGLFDIVYATDRATFHTPFSSLGLSPEGCSSYTFPKIMGLAKATEMLIFNRKLTAAEACSQGLVTEVFPDNTFQKEVWARLKAYASLPRNSLKLSKQLIRRTDKEKLNEANSKECACLQEIWASDECMNAVMNFFQKKSKL

>XP_003219917_ACBD6_Anolis_carolinensis

MASPFPAEEGGRGDQPSSGDEAEEGERAAAVAAALLGESPGPGSDSESPSPPGLAEQFERAAERVPGLVAVASKEQLLALYARYKQVKCGSCNTPKPGFFDFEGKQKWEAWKALGDTSTEQAMRDYVATVKKLDPAWNPQITEKKGKEGKGGFGGPVVSSLYQEETIREEDKNIFDYCTGTTNDHVTKAIRSKKVDVNTKDEKGRALLHWACDRGHKELASVLLQHTANVNSQDDEGQTPLHYASTCEFSDIVELLLKSGADPTLRDIEGYLPEEVTDCRVITSMLQEHTAGKA

>XP_003222139_ACBD7_Anolis_carolinensis

MERCKWKEARPGCKCSIKIVASLILLIIFLSHQADFDSVAEKVKKLKTRPTDDELKELYGLYKQVTVGDINIESPGALDLKGKAKWESWNQKKGMSKEDAMKAYISKANGLIQKYGL

>XP_003227602_ACBP_Anolis_carolinensis

MTSMPAFRVQLFQPPLGTATTQAEFDKAAEEVKKLKTQPADEEMLFIYSHFKQATVGDVNTERPGMLDFKGKAKWDAWNGLKEASERYQVSAVLSQVLRQGQE

>XP_003216136_GCP60_Anolis_carolinensis

MAAAAALSSERLEVSIDGLTLSPDSEEAPRGAQAQPRRPLPALVASHGGREEGEEQEETALAEEEPEREACEEEEEEEEEEEEEGGDRGVRSLERRWGFGLEELYGLALRFFKEKDGKAFHPTYEEKLRLVALHKQVLLGPYNPDTCPEVGFFDVLGNDRRKEWAALGNMTKQDAMTEFVKLLNKCCHLFSTYVTSHKIEREEQEKKRREEEERRQREEEERERLQKEEEKRRREEEERLRREEEERRRIEEERFRMEQQKQQIMAALNSQTAVQFQQYAAQQYPGNYEQQQILIRQLQEQHYQQYMQQLYQVQLAQQQAALQKQQEAAATVGASVTSMSKANSAVTGEIPSVNGQASPHIENPEKDLETDIAEEALENGPKESVPVIAAPSMWTRPQISDFKEKIRQDADSVITVGRGEVVTVRVPTHEEGSYLFWEFATDNYDIGFGVYFEWTDSPNTAVSVHVSESSEDEDEDEENANSEEKAKKNANKPQLDEIVPVYRRDCHEEVYAGSHQYPGKGVYLLKFDNSYSLWRSKTVYYRVYYTR

>XP_007424768_ACBD7_Python_bivittatus

MTLQADFETVAENVKKLKSKPTDDELKELYGLYKQATVGDINTECPGVLDLKGRAKWEAWNLKKGMSKEDAMKAYISKANEMIQKYGM

>XP_007420819_ACBD5_Python_bivittatus

MAETESGGGGSVHATRFEAAVKVIQSLPKNGSFQPTNEMMLKFYSFYKQATQGPCNSSRPGFWDPIGRYKWDAWSALGNMPKEDAMIAYVEEMKKILETMPMTDKVEELLHVLGPFYEIVEDKRNRGSDLIADPCNAVTSVPAVNGKSENNDSGAESEEEPHQEEITDVPQIDKGVSEAEDVKQNSSPDKDLDSIVINGCCNDVSNVDASNGTQTKSALNGINVDEEIKARDKSLELPVISNSDSSHQGLNEDATEVSGIQLLTSDSDSEIYCDSMEQFGQEEILEISSPTKEFSRLPFVFSEVDHNNLLETSGFLELGNHKAEGIKEAATEGKGEVKCGGEDGKANDGGPQKEKKSGEKVDFYGIRRGRGHRMYPLGDGAQGGQMGSGGDGERWGSDRGPRSSLNEQIAVVLIRLQEDMQNILQRLHALETLTTSQATLHSDNQPDLPVKKPSWWPFEISPGTLAFAILWPFVTQWLVHIYLQRRRRKQN

>XP_007441213_ACBD4_Python_bivittatus]

MGLKTEETNCQKQFEAAVQVIQGLPRNGSYRPSYEEMLRFYSYYKQATVGQCQIPRPGFWDPIGRYKWDAWSSLGKMTKEEAMAAYIMEMKKAAQKVINSVPMDEASEDMFVYFEPLYEVIHDMPRPPESFFKKKSVKLDSESASLQNSSLNGLAEICPNPPAYEEYKQAGLGLANKAMRSSQMTSDSENEVFCDSLEQVEPHQARRLSAKQSFPLSNPPSSQAAEKRGSAMDRSAPSHLTHLHTAPAKISGSLEAIKESQAGVLTQKDVTLQVMSTIQALQQDMKKVMERLSYLESWAALQHHTSMLNPCLPAKVPDAKELPSWPVRLSPHTWFFLLAWPFVAQWLLWCFQRQKR

>XP_007425599_ACBP_Python_bivittatus

MTQAEFDKAAEEVKKLKTQPTDAEMLDIYSHYKQATVGDVNTERPGMLDFKGKAKWDAWSALKGMSKEDAMKAYIAKVNELKDKYGMQ

>XP_015744757_ECI2_Python bivittatus

MFLAAVLKFLRPNPLRSCSPLCGISKVYGVHFPVLQLHTTNSTMQFSQEDFNKAKEQVKVLQDEPSDEVKLKLYALFKQATLGQCNTPKPSMLDFVNKAKWDAWHSLGSMTQDNARQSYIELVSSLVSAESSPVNEIPPGSKSNYETLQVTTKDNITKITLNRPKRKNAINVKMYNEIMEALDEAANDDSALTVLTGNGDYYSSGNDLNNFANISAGGMEESAKNGAVLLKNFVQCFIDFPKPLIAVVNGPAVGISVTVLGLFDIVYASDRATFHTPFSNLGQTPEGCSSYTFPKIMGLTKATEMLLFNKKLTAAEACSRGLVTEVFPDSTFQKEVWTRLKAYANLPKKVSVSKIAYIISCITWCYYILLRWNGIAISNVFSTVKFSNLNNKLDAKLNETVVDVKQLNDKLVILENNMDNKGMEEERDLDTLLDFKEKEPYFGLPVTSEWPNDDLILDLQKDEF

>XP_007438397_GCP60_Python_bivittatus

MAAAAALSSERLEVSIDGLTLSPDSEDARPGQADTRPLPLAGSRGDRVGTCGQPAQARDEAGGDSDGGGGLSLERRWGFGLEELYGLALRFFKEKDGKAFHPTYEEKLRLVALHKQVLLGPYNPDTCPEVGFFDVLGNDRRKEWAALGNMAKQEAMTEFVKLLNRCCHLFSTYVTSHKIEKEEQEKKRREEEERRQREEEERERLQKEEEKRRQEEEERLRREEEERRRIEEERFRMEQQKQQIMAALNSQTAVQFQQYAAQQYPGNYEQQQILIRQLQEQHYQQYMQQLYQVQLAQQQAALQKQQEAAATALVTSVSKAKSATAGQISSVNGQANAHAGSPEKDLEIDALEEALENGPKESVPVIAAPSMWTRPQIADFKEKIRQDADSVITVGRGEVVTVRVPTHEEGSYLFWEFATDNYDIGFGVYFEWTDSPNTAVSVHVSESSEDEDEEEENASSEEKAKKNANKPQLDEIVPVYRRDCHEEVYAGSHQYPGRGVYLLKFDNSYSLWRSKTVYYRVYYTR

>XP_015744972_ACBD6_Python_bivittatus

EAWKALGETSLEQAMREYIDTVKKLDPTWNPQISEKKGKEGKAVFGGPVISSLYQEETIREEDKNIFDYCRENNIDHVTKAIRSKKVDVNTKDEKGRALLHWACDRGHKELATVLLQHTADVNSQDDEGQTALHYASTCEFSDIVELLLQSGADPTLRDTEGYLPEEVTDCRLITSMLQKHSAGKA

>NP_001008180_ACBD5_Xenopus_tropicalis

MADTKPLHQTRFEAAVSVIQSLPKNGSFQPSNEMMLKFYSFYKQATLGPCNTPRPGFWDPVGRYKWDAWNSLGDMSKEDAMIAYVDEMKKILETMPVTEKVEELLQVIGPFYEIVEDKKHGRGSGVTSELGSVLTSTPNGKAVNGKAESSDSGAESDEEQAATKEVREEDEEEESEHSEQEDKDVEQQPGHEKPAESIVNGLTRNHRELVTEEPTPLPSKCLSEPGDKVAIPDTHSPVNDPEADREEDCTEDIAAMQHLTSDSDSEIFCDSMEQFGQDEADHSLLLQDAMLNGDITETSAGGELKDGGEDGKQSGHGAQRKTWSEKSEHFGSRRERPSRMQPGGDGSRSGQIGSGGDGDRWGSDRGPNGSLNEQIAVVLMRLQEDMQNVLQRLHSLEVQTASQAQFLLRESNNQPMEKKPSRWPFGISPGTLALAVVWPFVVHWLMHVFLQKRRRKQT

>NP_001072822_ACBD4_Xenopus_tropicalis

MGTDQEETGYQKQFNAAVSVIQNLPKNGSYRPSYEEMLRFYSYYKQATVGPCNIARPGFWDPIGKYKWDAWNRLGRMSQEDAMCSYIQEMKLVAQKVIDTVPLEDTSPEMFEHFRPLYEVIPDMPRPPDSFFSMSFEETTFREAEEKQQEEGNEELSYSLTQNSFSGGTNVSQAGESQSISTARERRGDPVGEDSHQAQGEERRGQILGAETEDSSDSLEQLDLDKDFNDTVTWIYPHLFRPSSDVIPADSNLQRQKEDTLKVSDHGCERDAWQMNPSREHNLDSAKRSQSRWRKSQHQLNPQIMATVQSLQNSIQVLSDRLESMEKALKEQQRVECSRRYKPQKPWFSFPAPSRTLFFILVWPFIVNWLLHRYYRRKR

>NP_001135696_ECI2_Xenopus_tropicalis

MQARQEDFEKAQSNLKLLKNDPGNEVKLKLYALFKQATQGPCNVPKPGMLDFVNKVKWDAWKSLGSLPKDDARQSYVELVSSLVSSESSTKSNADPGIGHKKYETIHVSCEDNIIKIFLNRPEKKNAITLTMYKEIGEALEEAGKDESVFAVLSGFGDYFCSGNDLNNFTNIPPEGKEKMAKDSADLLETFVSKFIDFPKPLIAVVNGPATGISVTILGLFDLVYATDRATFHTPFSQLGQSPEGCSSYTFPRIMGLGKATEMLLFNKKLTAQEACNLGLVAEVFPDSSFQKEVWERIKDYSTLPKNSLAFSKQLIRVNEKEKLHAVNIQECERLKERWLSEECMNAIISFFQKRAKL

>sp|Q66JD7_ACBD6_Xenopus_tropicalis

MASPGVLEESSSGEACSGGCPEQWDEKTEEELQGQFEQAAKHVQNVASVASTEQLLFLYARYKQVKVGRCNTPKPGFFDYEGKKKWEAWKALGDYSCQQAMNEYIETVKKLDPDWSPQALEEPHKEPKTTFGGPVVSCLYKVQETLREEDKDIFDYCRENNISRVSHALSTGAIDVNVADDEGRCLLHWACDRGHTQLVSVLLFHNAHINMQDSEGQTPLHYASACEFPDIVDLLLDHGADPSLVDNDGFQPHEVTDSKNIAAMLQQHASYGEHNKPASLLLEMPQ

>NP_988874_ACBP_Xenopus_tropicalis

MSQEAFDKAAEEVKQLKSTPTDEEMLETYALYKQATVGDVDTARPGMLDFKGKAKWDSWKKKEGTSKEDARAQYVDWVEKLKAKYGS

>NP_001165067_ACBD7_Xenopus_tropicalis

MSPQADFDKAAEDVKKLKTRPTDEELKELYGLYKQSTVGDINIDCPGMLDLKAKAKWDAWNLKKGLSKEEAMHAYISKTNELVEKYGL

>NP_001096198_GCP60_Xenopus_tropicalis

MAVVSGAERLEVSIDGLTLSPDSEELGRPGLGDVPLGEEGEGLSLELRAQTEAAALSMEQRWGFSLGELYGLALRFFKEKDGKAFHPTYEEKLRLVALHKQVLLGPYNPDTCPEVGFFDVLGNDRRKEWAALGNMDRQEAMTEFVSLLNRCCHLFSTYVTSHKIEREEQERRRREEEERRRKEEEERERLQREEERRRKEEEERLRREEEERLRAEDERFRMEQQKQQIMAALNSQTAVQFQQYAAQQYPGNFEQQQILIRQLQEQHYQQYMQQLYQVQLAQQQAAIQKQQEAAVARSCSPLTSPSKMHSVGAAAPGETPSVNGQSHSHLDSNEPEMESDPLEDLLENGPKESAPVIAAPSMWTRPQIKDFKEKIRQDADSVITVGRGEVVTVRVPTHEEGSYLFWEFATDNYDIGFGVYFEWTDSPNTAVSVHVSESSEDEEEDEGNENPNSEEKAKKNANKPQLDEIVPVYRRDCHEEVYAGSHQYPGRGVYLLKFDNSYSLWRSKSVYYRVYYTR

>XP_018422996_ACBD5_Nanorana_parkeri

MADTKPLHQKRFEAAVSVIQSLPKNGSFQPSNEMMLKFYSFYKQATQGPCNIPRPAFWDPVGRYKWDAWNSLGDMTKEDAMVAYVDEMKKILESMPMTEKVEELLQVIGPFYEIVEDKKHGRGSGVTSVRLDKVAKSLEELSTALTSTPNCKAVNGKAESSDSGAESEEENTGRREEEEEEEDDDDEGETEQSEQEEKTGMPDSAPEPCPGGTVANGLLINHSYYIPDIPNHSPTKSDLNKMEEEVTGKQHNTEDGGSNDVDGKKDNDCTEDVAAMQHLTSDSDSEIFCDSMEQFGQEDVDHSQLIEEAMLTEGSSDVWAGGEVKDGGEDGKASGRTPQKEKSGQDKAEYYSSRRGRANRMHPVGEGSRGGQMGNGGDGGDRRGSDRGPNSSLNEHIAVVLTRLQEDMQNVLQRLQTLEALTASQARSIQLESNFQQLSNQKPSWWPFKISSGTLAFAIVWPFVAHWLIHLYLQRKRRKPT

>XP_018410673_ACBD4_Nanorana_parkeri

MGTDQDESGYQRQFNAAVSVIQGLPKSGSYRPSYEEMLLFYSYYKQATVGPCNITRPGFWDPIGRYKWDAWNKLGQMSQEDAMGAYIREMKKVAQKIINTVPLEDKTPEMFEPFRPLYEVIPDMPRPPDSFFKTYTEEETFEVDKYQVLKEEKEEQYVSTPEQTDPGAHWGQGKEEERSDPVGPERMKDHDPATVSPSLGHACLKNPGESSSSDMSSGSQNTCRSSSHHLSPQIAATVESLQASVQGLSQQLEVLERILQDQQQYIRERIQRSSKEPNPQRSGIGQSQTLLFIVIWPFFVNWLLRRLYGRKS

>XP_018425955_ECI2_Nanorana_parkeri

MAASLLYVTNCLRLSPFRAVSKLRSVSVLGVHTTARHMQASQEDFEKAQNDLKTLKKDPGNEVKLKLYAFFKQATQGACNVPKPGMLDFVNKAKWDAWNSLKDLPKEKARQSYIELVSSLISAELPVKDPTSTSANKKYETLEVFSQDNITKIFLNRPEKKNAITLQMYEEIGLALDEAAKDDSVITVLTGHGDYYCSGNDLNNFTNIPPEGKEKMASDSALILESFVGKFIDFPKPLIAVVNGPAVGISVTILGLFDVVYATDRATFHTPFSQLGQSPEGCSSYTFPRIMGLSKASEVLLFNKKLTAHEACQLGLVTEVFPDSTFQKEVWERLNSYSSLPKNSLAFSKQLTRAPEKEKLHAVNREECERLKERWLSEECMNAIISFFQKRVKL

>XP_018431086_ACBP_Nanorana_parkeri

MDEARRCKRIGGRVRVTPPVAERSEIPPPCNPEWKQEIYRIHQSGDGRRLLCVLLSQAEFEKAAEEAKKLTKKPADDEMLKLYALYKQATVGDVNTARPGMLDFTGKAKWDAWESKKGISQEDARAQYIALVEELKGKYSS

>XP_018424658_ACBD6_Nanorana_parkeri

MASPEVLEEYSSGEACSGGCGDLGDFLLEDRTEEELASHFEQAAKHAQKLAHVATTDQLLYLYARYKQVKVGKCNTSKPGFFDYEGKRKWEAWKSLGDCSPQQAMRDYIETIKKLDPDWSPKSFEDPVEEHKSSFGGRVVSCLYQVPETLRDEDKDIFDYCRENDISRISQALATGAIDVNVADDEGRCLLHWSCDRGHSELVSTLLFHNAHINMQDSEGQTPLHYAAACEFIDIVDLLLDHGADPSLVDNDGFQPHEATDSKQISDMLKQHATFGEHDKPPSLLEISK

>XP_018422372_ACBD7_Nanorana_parkeri

MAPQADFDKAAEDVKKLTKRPTDDELKELYSLYKQSTVGDINIACPGILDLKAKAKWDSWNLKKGLSKEDAMSAYVSKAHELIEKYSH

>XP_018423874_GCP60_Nanorana_parkeri

MAVVSGVERLEVSIDGLTLSPDQEEEPPQGAGEVGEEDEAGQISMEQRWGFSLEELFSLALKFFKEKDGKAFHPTYEEKLKLVALYKQVQLGPYNPDTCPEVGFFDVLGNDRRKEWASLGNISKQEAMTEFVGLLNRCCHLFSTYVTSHKIEKEEQERKRREEEERRRQEEEERQRLLREEEKRRREEEERLRREEEERMRAEDERFRMEQQKQQIMAALNSQTAVQFQQYAAQQYPGNFEQQQLLIRQLQEQHYQQYMQQLYQVQLAQQQAALQKQQEAGLGGSGSPVNSPTKGSTAASGETPSVNGQSHPHIDGTETEMEPDPLDDILENGPKENTPVIAAPSMWTRPQIKDFKEKIRQDADSVITVGRGEVVTVRVPTHEEGSYLFWEFATDNFDIGFGVYFEWTDSPNTVVSVHVSESSEEEEEDEGWLEENPNSEEKTKKNSSKPQVDEIVPVYRRDCHEEVYAGSHQYPGRGVYLLKFDNSYSLWRSKSVYYRVYYTR

>NP_001018483_ACBD5A_Danio_rerio

MEGDSNPLYEQRFNAAVKVIQNLPPNGSFQPSHDMMLKFYSYYKQATQGPCNIPRPGFWDPVGKAKWDAWSSLGEMPKEEAMAAYVDDLKLILESMPVSSEVEELLQVIGPFYELVDEKRKITQVSDLSTGFGNLLSSPPKCVTKSIIRTMEMNGNLEGYPIKTAETLKVKSIDLEDREDDDDEDEEGERDEVEEFKEVEKASQPKKRVSAGRPKGPVSNGSISQHKGLSNGTHGSKSDLNRQESEENTEHMNHDGGIVELNGHLNSEKDKEEDVSSSHHVASDSDSEVYCDSVDQFGGEDGSEIHMNRSLEVLEESHSTPSSTGDIRSQDDELLGREEGVQHGGEDGRGSRGGAQRRELPVKRSDSSVVRRGRGSRSPASGSGSAGPQQGSGGDGERWGADGPMTENLNEQIICALARLQDDMQSVLQRLHTLEALTASQARSLALPSDYLTTPANRNKKKPSWWPFDVSLGTVAFAVVWPFVVQWLIRVYVQRRRRRIN

>NP_001093558_ACBD5B_Danio_rerio

MILRGMEDSKSAQKRFEAAVKVIRSLPEDGSYDLSDDMLVLFYSYYKQATEGPCNTLKPNSWDPIGKAKWEAWKDLGNMSKDQAMTEYVQEIQLIIETLPVTDRMAELLDALDPFYEIVEDDDDDDDEGVSKAAPLFTGSTNADKDAERDEEVESESKEGNLDDYMELVEKQKDLSSTTGEKGSLLVFNRSEENSISSLTDGTHSSLNTVDDEEELVYDGSDDEMDSDSMDKPATPEKGSGVRSVRLADGSVVGANMQHGGNREPQCGSQDGKPQGLISPVPHPPTLGTVRNDRISACSGRERGCQGDGGQRGETADRMDKQAINTQITTILSELEDNMQDVLRRLTTLEQLTASQAEISPSKTWHSDKPKKRLSWWPLNSSPFTAVLTVLWPFAVHWLVQFYLQRRRRR

>NP_998260_ACBD4_Danio_rerio

MPALTMSEIEDCQRRFQAAVDVIQSLPKNGTYKPSYEVMLRFYGLFKQAVCGPCTLSRPGFWDPVGRYKWEAWSNLGEMSRETAMAAYVDEMKKVAQDVIDTMQINEKNASYFHYFEPLYHVIHDMPRPPEALFSLSSDVNVKEPTGGVFKHNVDSATQEPRLQQDTETSLNSELQPQSTEQRMSECQGPISDTESEVFCDSLEQMDNIKLTAVPDGNSPFQNGRTCIEISGIQESHYSAPSQLVQIGVGHGEGAEDRHDPPMRMTTAGREWLQQNWRDSPGFMAHHSAGRLSAAGSGLVDRDNSKSGSGQLQQQIFLALRKLREDMQSVMERLDVVESLATANAQNSHWISHLQRTAGESEEKWWPFEISGRTLMLLLVWPFVTQWLSFLLRWKKRQIHGCI

>NP_001002645_ECI2_Danio_rerio

MASLIKHVSPWRFARFVRSSKTAFIPCVQLHSTAVMGASVEDFNKAKDKLNTLKKDPGNEVKLKIYALFKQATVGPCNTPKPGMLDFVNKVKWDAWKGLGSISQEEARQQYVDLISSLVGAEAPAVAAQPTGGGKTFQTLLVSTEDNITTIRLNRPDKKNAITVEMYNELIEALELAGKDDSVITVMTGNGDYYCSGNDLNNFTKIPEGGVEKMAKDAGELLRRYVKAYIDFPKPLIGVINGPAVGVSVTLLGLFDVVYATEKATFHTPFSQLGQSPEGCSSYLFPKMMSAAKASEVLLFNKKLSATQACELGLVSEVFPESSFQSEVWSRLKAYAKLPKNSLALSKQLIRGLEEEKLHAVNDAEVERLTERWLSDECMQAIMSFFQGKSKL

>NP_001020626_ACBD6_Danio_rerio

MASRSPSSSPDSATGSGTDPARPDTGEPLGGGSDSDSDFGLGKFDCSAGDASARLEGADLENEFESAADRVRDLVQTASREQLLYLYARFKQVKVGKCNTSKPGFFDFEGQRKWSAWKQLGDMSAEQAMQEYVTCVHALDPEGSQKSSERRGGEKRTGFGGPAVSSLYQEEKIREEDKNIFDYCRENNIEHVSKAISSKTVDVNTRDEEGRALLHWACDRGHKDLVSLLLQNNADINSQDDEGQTALHYASACEFAEIVELLLKAGADPSIKDQEGSLPEEVTESSAISSLLRQYTAPKG

>NP_955902_ACBP_Danio_rerio

MSEAEFQKAAEEVKQLKAKPTDAEMLEIYSLYKQATVGDVNTARPGMLDFTGKAKWDAWDAKKGTSKEDAVKAYIAKVEELKGKYGI

>NP_001122240_ACBD7_Danio_rerio

MTLKAEFDQYAEDVKKVKTRPTDQELLDLYGLYKQAVVGDINIDKPGMIDLKGKAKWDAWDSRKGMSTEDAMKAYITLAKQAIEKYGK

>NP_998162_GCP60L_Danio_rerio

MATEVQSGDLNSANSSRLEVSIDGLTLSPDSEGEQEQAETPDGQSAEQSHENPEQSVDGEEGEPTSSANIERKWGFALVELYGLALRFFKEKDGKAFHPTYKDKLRLVALHKQVLQGPYNPDASPEVGFFDVLGNDRRKEWASLGNMEKEESMLEFVRLLNKCCNLFAPFVASHKIEKEEEEQRRREEEEYERLRQEEERRRRVCMLKGS

>NP_001082856_GCP60_Danio_rerio

MEFDGEDQGSDRLEKTSSENGINTETHALDTQEDHKTCQDNETPQKSWSLDRNWGFTLEELFRLALKFFKEMNGKAFNPTYEENLRLVALHKQITLGPYNPNSCPDIGFFDVLGNDRRKEWLSLGSMAKEDAMEDFVKLLNSCCSLFAPYVTSHKIEKEDQERRQKEEEERLRLEREEQERRRLEEEERRREEEERRAIEEEQRRKEEAQRLQIERQKQQIMAVLNAQTAVQFQQYAKQQYPDNPEQQQLLIRQLQEQHFQQYVQQALRLQQMASQKQQEESTVIQTSEALPAPISNEAVSICNPNSQSNSLDNHKQQKDQKNSHLVVEGETGVVPIVAPSMWTRPQIKEFKEKVLHDEDSVITVGRGEVLTIRVPTHPDGSYLFWEFATDHYDIGFGLHFEWKDLTAPTSSNNGGESTTETKEGTAQAEEEKTEQGRIPLVNEVAPVSRRDSHEEVYAGSHQYPGEGVHLLKFDNSYSLWRPKVVYYRVYYTR

>XP_018620717_ECI2_Scleropages_formosus

MAVQLLLYPARLVQVLRTPVLRLHVTARTMGATVEDFNRAKDQLGTLKKDPGNEVKLKIYALFKQATQGPCNTPKPSMLDFVNKAKWDAWKSLGSVSQEEARQKYVELIRLLVGDEESIQAAPTSAGSSAIFKTLLFNTEDNITTICLNRPEKKNAITVPMYSEIVKALEMASKDDSVITVITGSGEYYSSGNDLNNFTSVPEGDIEKKAKDAAELLKGFVKAFIDFPKPLIAVVNGPAIGISVTLLGLFDVVYATERATFHTPFSQLGQSPEGCSSYTFPKMMGAAKASEVLLFNKKLTATEACERGLVTEVFPDATFQIEVWKWLKAYSKLPRNSLALSKQLMRAVEKEHLYAVNAQEVQRLAECWQSDECLNAIVNSFFQAKAKL

>XP_018587841_ACBP_Scleropages_formosus

MSQAEFDKAAEEVKHLKVKPTDAEMLEIYSLYKQTTVGDVNTARPGMLDFAGKAKWDAWEKKKGMSKEDAMKAYIAKVEELKEKYGI

>XP_018618608_ACBD7_Scleropages_formosus

MSLKAEFEQIAEDVKKVKTRPEDQELLDLYGLYKQVIVGDVNTEKPGVLDLKGKAKWEAWNSRAGMSKDDAMTAYIALAKEVINKYGM

>XP_018596665_ACBD5_Scleropages_formosus

MMMMSMTDERTVHERRFDAAVKVIKSLPANGPFQPSNEMMLKFYSYYKQATQGPCSIPRPGFWDPVGKAKWDAWNSLGDMPKEDAMIAYVEEIKVSGPACEVWILQILESMPVTDEVEELLHALGPFYELVDEKKKITQVSDLTTGFGSMLSSPPKSVTKNIVRSLTMNGTLENHTRRAESDGRKPKAAEDGEMALEEGVLHLTPENEILTGKQNGQLETAITPLSNGYPESVPSLTNGTHSSKSALNSDESEEELLHTSTADHKELNGHITEHSEDPMGLHHMASDSDSEVYCDSMDQFGLEESAEVLVSHTLESDHSLAETCALVDGCLQTESEGVQCGGEDGKPSGNVPPRERLSAGRADSSLGRRGRGPRFAGDVSQGGLAGGGGDGERWGPEGGPRGSLNEQIVTALSRLQEDMQSVLQRLHTLEALTASQARSLALQSNYPSFPPAKKKPSWWPFDVSPGTVAFAVVWPFVVQWLIRLYLQRRRRKMN

>XP_018613115_ACBD5-L_Scleropages_formosus

MAEGNSVHECRFDAALKVWRNLPDDGPVQLSDDLKLRFYGFFKQATTGPCNIPKPGFWVSEQKAKWEAWSSLGDMSKEEAMMAYVEEMKTILESLPITDEVEELLDVLGPFYEVEEEEAMPDDESFPKATKGSDGILGDEERNGNVASSDTDFESDISESEEEEDDEEEGDDEQQEEKKAEEGYVCTAEDTTTGLSSDHRAPFSVSSASSRAHSSLDGENMEEELTRIAPAQEDNLCVHCEDTSGAFHLTSDSDSEVYCDSIEHFGTVLDSVEFFNQSLQLTGETQCPPESSAELSRVEKMVVVKGLQEGDRGNGEMRRNRSQSDRFSTGEEESIPARRRGSMLSGSSSQSEAQLGLRSPGRDGDHRGKEGRAVGGVDKQIATVLLRLEEDMQHILQKLHNLETLSEEQARSIAAVQNYPTFPRHKVQHENSVISHCVFTL

>XP_018582476_ACBD4_Scleropages_formosus

MPPDSRTLRARPSFTVAAAMTQPEEECRRRFRAAVDVIQSLPKNGSYRPSYEVMLRFYGLYKQAVFGPCRSSRPGFWDPVGRYKWDAWSRLGEMSGEDAMTEYVEEMKKVAQEVIDSVPVNKKTASLFHYFEPLYLVIHDMPRPPEALLSLRAELEAGVQTASPAQGDSQEKMERDPEQAAKESACGPDVGSRLSGGLRGSAATATFSAVEPAPPQGSVGTSDSESEVFCDSLERLDRKAARVGAGAGEERAPPPRGRGFGLEARRGQREETRGGPRGGGRRAREGASGRHVRRDGAPGGCGTDGCRTGSIL

>XP_018582219_ACBD6_Scleropages_formosus

MSVEQAMQEYVSCVQALDPDSSHMFSEKRAKERRTGFGGPAISSLYQEEMIREEDKNIFDYCRENNIERVTKAISSDKVEVNAKDEEGRALLHWACDRGHKELVIVLLQNNADINSQDNEGQTALHYASACEFADIVELLLQAGADPSIKDQEGSLPEEVTESKDISSLLRQYAASKG

>XP_018590566_GCP60_Scleropages_formosus

MMAAELQRPDTNSDSSSRLEVSIDGLTLSPDSEGEQEQVERLNPLVREEESALQSKARGNDGEDEPETENAKTMERKWGFSLQELYGLAVKFFKEKDGKAFHPTYEEKLRLVALHKQVTLGPYNPDACPEVGFFDVLGNDRRKEWASLGNMEKEDAMVEFVRLLNKCCKLFAPYITSHKIEREERERKRKEEEEQRRREEEEQERRRQEEERLRQEEEQRLRREEEERRQMEEERLRVEQQKQQIMAALNAQTAVQFQQYAAQQYPGNVEQQQVLIRQLQEQHYQQYMQQLYQVQLAQQQAALQKQQEEAIVSSSSEGGLCAARPSAMDSPALNGQPSAHAECLDRESEVEPAEELIENGPVDVAPVIAAPSMWTRPQIKDFKEKIRQDADSVITVGRGEVVTVRVPTHQEGSYLFWEFATDHYDIGFGVYFEWTDSANAAVSVHVSESSDDEEDESGDGPSEEEKAKKNASKPQVDEIVPVYRRDCHEEVYAGSHQYPGRGVYLLKFDNSYSLWRSKTVYYRVYYTR

>XP_003457227_ECI2_Oreochromis_niloticus

MAVSTECRGVCGYLTANDICQTQVSHRDGLRLTGALLALKAEKYQLLYKQLAAASHDLCSTFRHDLKMRLSFILCSFSERVTVRSNIPSLKFHTTASPMMGVTVEQFEQAKSKLSTLKNDPGNEVKLKIYALFKQATQGPCNTPKPGMLDFVNKAKWDAWKSLGSISQDEARQKYCDLIGSLVEAESGSSAQVSAQPAGSGATYETLLVTKEDDITTIKLNRPAKKNAITTEMYNEIIAALEQAATDDSVITVVTGAGDFYCSGNDLTNFTKIPENGVEEMARHGADLLRKYVNAYIDFPKPLVAVVNGPAVGVSVTVLGLFDLVYATERATFHTPFSQLGQSAEGCSSYIFPKLMGAAKASEMLLFNKKLTAVQACELGLVTEVFPDSSFQSEVWTRLKAYAKLPRNSLALSKQLIRSVEKERLHAVNDAEVERLMERWTSDECFNAVMSFFQAKAKL

>XP_003456254_ACBP-L_Oreochromis_niloticus

MAELQTKFDEAAAEVKQLKAKPTDEEMLQIYSLFKQATVGDVNTSRPGMFDFTGKAKWDAWEKQKGKSKENAMNEYISLVEELKQKYGI

>XP_003450922_ACBD7_Oreochromis_niloticus

MTLQAEFDKAADDVKKVKAKPTDEELLFLYALYKQAVVGDINTERPGMLDLKGKAKWDAWESRKGMSKEDAMSAYIAKAKEVISKYGV

>XP_003439326_ACBD5_Oreochromis_niloticus

MEVESVRVEDEGRLTQMRFDAAVKVIKSLPPNGPFQPSNDMMLKFYSYYKQATVGPCNIPRPSFWDVVGKAKWDAWNSLGEMSKEEAMSSYVDEMKLILEGMPMTDEVEELLKVLGPFYELVDEKKKITQISDLSTGFGTMMNSMASNSVAKSIVRNMEMNGTLETRPARLKQKEVRSEEDALEEDDEEEDEEEEDEEEEEEEEEEMVTREVRKDKKSGPSQPKKKTFGRRQKAPLSNGKVANGVTHLTNGSHSRAALNSDKTQEGSLGEPLLNGHNPDPSVAASAPNHLTSDSDSEVYCDSVDQFGQEENSEHNRSLDDLDEEQNHLQPPLEERRETREDVQGPLQVVRCGGEDGEAGGAMSQRLNVNVPGGSSARRGRGKGSRSPGLRPGSQMPMHSAGDGDGGRWGGAGTPGANLNEQIVVALARLQEDMQSVLERLHTLEALTASQARSVALSPTYASPPVNNRSQKPSWWPFDISPTSLAFALIWPFVVQWLIRLYLQRRRRRIN

>XP_003458607_ACBD4_Oreochromis_niloticus

MIEPVVDHQKRFQTAVDVIQKLPKNGSYRPSYKVMLRFYSLYKQAVCGPCTVPRPGFWDPVGRYKWDAWNQLGKMSSESAMAAYVDEMKKVAQEVIDIMPIDENTASLFKYFEPLYLFIDDMPRPPESLLKLREAESAVLEVMPLTSDSESEIFSDSVDSLELHNIKTRSDSFYNDHVSLESSPAQSQKEGRKVGAGQGGEGAEDGKDPVRRGRHTGQETSQRDWRERAPQGSLGWSPGGGSGHGRGGGHSSEGGAEMLREAHLQQQIILALQRLREDMRSVMDRLEVLERLAATHAHDSDLRSRLQCSLQQEQMSWPFDMSGQTVFLLLWPFVAHCLVYLLRKKSRMSS

>XP_003440905_ACBP-like_Oreochromis_niloticus

MTEAFHKAVEEVKVLKQKPNQQEMGDLYGLYKQATVGDINTERPGFLDFTGKAKWDAWNARKGLSKEEAMVKYVTLVEELKEKYGI

>XP_003440039_ACBD6_Oreochromis_niloticus

MASGSPSSSPDTATGSGTDPARPDTGEPLGGAGSDSDSDLGLSKFDCSAMEVGDRLEGEELEREFESAADRVKDLIQTASRDQLLYLYARYKQVKVGKCNTPKPGFFDFEGQRKWQAWKQLGDMDQEQAMQEYISCVNILDPEGSTKSNERRGAERRTGFGAAVSSLYQEEMIREEDKNIFDYCRDNNIEYITNAITSQNVDVNIRDEEGRALLHWACDRGHKEMVSLLLQHKADINIQDDEGQTALHYASACEFVEIVDLLLKSGADPTITDQEGCLPEEVTESRAISLLLRGYTTSKG

>XP_003449949_GCP60_Oreochromis_niloticus

MATEVQSGDLDSATSSRLEVSIDGLTLTPDPEGEQSQAEEPESIPTEPSRDKPGGAEGEDGETAKCAIERKWGFPLLELYGMALKFFKDKDGKAFHPTYEEKLRLVALHKQVLLGPYNPDASPEVGFFDVLGNDRRKEWASLGNMEKDDAMVEFVKLLNKCCNLFAPYVTSHKIEREEQERKRREEEERLRREEEERERQRLEEERRRLEEEERLRREEEERRQAEEERLRIEQQKQQIMAALNAQTAVQFQQYAAQQYPNSPEQQMSLIRQLQEQHYQQYMQQLYQVQLAQQQAALQKQQQSDSMVQGTLESSSFHTGEPIPASSAGPLCAAALPQTGEEAPTINGGQSDSYSESMDREPEPEPAEEVSENGPLLAESPPVIAAPSMWTRPQIKDFKEKIRQDADSVITVGRGEVVTVRVPTHEDGSYLFWEFATDYYDIGFGVFFEWTDAASASVSVHVSESSDEDEDDEELVNCEEKAKKEAGKPQVDEIVPVYRRDCHEEVYAGSHQYPGRGVYLLKFDNSYSLWRSKSVYYRVYYTR

>SINCAMP00000011604_ECI2_Callorhinchus_milii

SVWCFLPILILHLHCRLIQSFPAVKLHLTGAVMRATEVEFDKAKEHLKTLKNDPGNDTKLRIYALFKQATQGSCSSPKPGMLDFVNKAKWEAWNSLGNISKEDARQKYVDLVGTLISSEAPIQKEATTAGDPKSSYQTLQVTTENNITTILLNRPEKKNAISRKMYEEIMQALEQAGKDDSVLTVMTGSGDYYCSGNDLSNFTQVGAEGVEKLARDSGELLTRYVSHFIDFPKPLIAVVNGPAVGIAVTGLGLFDVVYATDRATFHAPFSQLGQSPEGCSSYTFPKMMGTAKANEILLFNKKLTAAQACDLGLVTEVFPDNTFQQEVWKKLRAYAKLPKDSLRYSKQLIRGMEKEKLHAVNAQECVRLQERWLSEECMNAIMSFFQNKSKL

>SINCAMP00000019993_ACBP_Callorhinchus_milii

MTEVLGQWRCSFTGKVILLCYKRIAFQKAAEEVKHLTTVPTDEEMLAIYSLYKQATVGDVNTERPGMLDFKGKAKWDAWSKLKGTSKEEAMKLYIAKVEEMKVIYGMAE

>SINCAMP00000010803_ACBD5_Callorhinchus_milii

MSARRKPYSLAPINVSAAGKALWDATLGSFQPSNELMLKFYSYYKQATLGPCNTPRPGFWDPIGKYKWDAWNSLGDMSKEEAMIAYVEEMKTILESMPMTEKVEDLLRVIGPFYELVDDKKQTLSPLAISADLGNVLSSAQNCDKMNGNIESNDSSAESEQEEGEEDDDEDATDDDEKEENEVVKLQNLTQGPAVDMKAELVLPNGTVGEKLIPCAYSTFQNSKSSLNGEETADKKTVLQMGPATDINGEIDGMENHLEDISGIQHITSDSDSEDYCDSMEQLGLEEISKHISDGEMLNSSPKISAVAYSRLSGSTRWNIQPELEHSAGTIELQGEHKGREGGVAHGGEDTKPSGSGSYKEKIATEKTESRGLKRMRGNRMQYLGDSSQVAQQGSSGDGERWNSDWVQNRSLNQQIAAALSRLQEDMQDVVERLHTLEALTVSQANSENLQPNSRSDTFVKKSSWWPFEVSGHTLAFAVLWPFIAQLLVHLYFQRRKRKLN

>XP_007883608_ACBD4_Callorhinchus_milii]

MAETPDDHQRRFQAAVSVIQSLPKNGSYRPSHEVMLRFYGYYKQATVGHCNISRPGFWDPIGRLKWDTWKALGNMSKVDAMTAYVEALKKVAGEVIESTAVTGTDAAAREHFHLFEPLYAVVDDLPRPAGFPLKATDGHSESLLTPTLRVKVNGIPRDGESPAPRASEGHRTANGFCRGEGEEGGSDWSLSDGAIPRDGEPEEEAEISEPGPGLKTERSRGLEESPSAPRSTRGDCAERTTRPSRVSSDLENEVYCDSA

>SINCAMP00000022751_ACBD6_Callorhinchus_milii

LRSEAKFGQCNVPKPGFFDFEGRQKWEAWKEMGDISGRQAMQEYITAVKELDPEWNPKASVKDSDLNIRFGGPVVSCLYQEEMIREEEKTIFDHCRENNIDHVSKALISKNVDVNLKDEEGRALLHWACDRGHKDQVSMLLQQKAEINSQDDEGQTPLHYASACEFTEIVELLLDAGADPTIQDGEGCLPEEVTDCKDISQMLRQHTTSKG

>SINCAMP00000013248_GCP60_Callorhinchus_milii

MLRMMAAADGSAGSNRLEVSIDGLTLSPEPERQEVEAASASASVHSHNRLNSLPDGPTPGSGDGEGGGSAVAAANDVDPELEPEQTPREAEKMENAWGFPLRELYCLALKFFKEKDGKAFHPTYEEKLKLVALQKQISAGPYSPDTCPEVGFFDVLGSDRRRVWTALGSMQKEDAMVEFVQLLNKCCCLFQPYVTSHKIEKEEQERKRREEEEKRRQEEEERERLRQEEERRQLEEEEKKRREEEERRRLEEERLRMEQQKQQIMAALNAQTAVQFQQYAAQQFPGNLEQQQLLIRQLQEQHYQQYMQQLYQVQLAQQQATLQKQQEPIVAATHSGVGEMPTVNGQTPGQMDSLEKEPEPETAEEVLENGPKDGIPVIAAPSMWTRPQIKDFKEKIRQDADSVIAVGRGEVVTVRVPTHDEGSYLFWEFATDNYDIGFGVYFEWTDSPNTAVSVHVSESSDDEEEDEESPSGEEKVKKSARKVQVDEVVPVYRRDCHEEVYAGSHQYPGRGVYLLKFDNSYSLWRSKTVYYRVYYTR

>XP_014349528_ACBD5_Latimeria_chalumnae

MAETKSIHEKRFEAAVNVIQSLPKNGSFQPSNEMMLKFYSYYKQATQGPCNIPRPGFWDPIGRYKWDAWSGLGDMSKEDAMIAYVEEMKKIIETMPMTEKVEELLHVLGPFYEIVEDKRLNKIVNIASDLGNVLTSTPNCKKVNGKADSSDSGAESEEEEDEDDQEEEDEAEQEEKDETSVKNLKNMMHDSAVDLKPDGMLTNGPSEEKHGLCTVNGIQDKLPVNDEKQAEELHKTENSIMLPEISKVNGYLEPNEDHCEEISSPQHLTSDSDSEVFCDSMEQFGQEESSEVLSSGRALLNHSGEMPAVHPDKLLENVNFKQVNSQKSSVSAVEMAPGVEGEVKHGGEDGKPSGSGPHKEKMSSERTEFPGPRRGRGSRTQPLGDGAHGGQLGIGGSGGDGERWGTERGPKSSLSEQIAVALMRLQEDMHSVLQRLHTLEVLTASQTRSVCFQPNNPAASSANKV

>XP_014353953_ACBD4_Latimeria_chalumnae

MAQAEADSHKQFQAAISVIQNLPKKGFYRPSYKAMLHFYSCYKQATTGPCNIARPGFWDPVGQYKWDAWSKLGNMTKEDAMMAYVTEMKKVAQEVIDTMQMTEKAEEMFQYFEPLYEVIQDMPRPPESFFKKRLNKRKESMESNINIRLNQVLENHESEDFCVKQTKEKQEAEADGSDVTHLNIKNERTEPMLNCRDACSSSSRSVENSCDSRVELLNFPATRRPLELMECKEVKKSAHQFLKPDSKGADRMEESTQCNQVTSDSESELYCDSVEQLEQEKGSQLFANQNLSFGAVDSSQFISNTVPERQMVVQMEASQGGERLRQSGSPRRKTNSGGSGSRLQHQRGMELPSGPPNQQISGSNEGEKKQPDWKQPGDLNNQIGLLLLRLQEDMRIVLLRLNT

>XP_005991125_ACBP_Latimeria_chalumnae

MSQAEFEKAAEEVKNLKSKPNDQDMLDIYSLYKQATVGDINTARPGMLDFAGKAKWDAWEARQGMSKEAAMKAYIQKVEELKGKYGI

>XP_006010031_ECI2_Latimeria_chalumnae

MATALLGISRWQLLRKARSEFSPPANVLQLEAGFHSGTWRPSKRFVQALYPPRLQAHTTGAAMGATQEQFEKAKEQLGLLKNDPGNEVKLQIYALFKQATQGPCNVPKPGMLDFVGKVKWEAWNSLGNLSKEGAREKYVGLVSSLVSSESSPQAEAVSVAGKQLYKTLQVTTEENITTIRLNRPEKKNAISIVMYNEIMQALEEAAKDESTITVITGTGDYYCSGNDINNFTMIPPEGIEKMAKDSAQLLKNFVLHFIDFPKPLIAVVNGPAVGVSVTLLGLFDAVYATDRATFHTPFSQLGQSPEGCSSYIFPKLMGSAKATEVLSFNKKLTAREACDLGLVTEIFPDASFQKEIWLRLRGCAKLPKMSLVYAKQLSRGPEKEKLYAVNIQECERLEERWLSEDCRNAIAKFFTRSKI

>XP_005994698_ACBD5L_Latimeria_chalumnae

MEESTQCNQVTSDSESELYCDSVEQLEQEKGSQLFANQNLSFGAVDSSQFISNTVPERQMVVQMEASQGGERLRQSGSPRRKTNSGGSGSRLQHQRGMELPSGPPNQQISGSNEGEKKQPDWKQPGDLNNQIGLLLLRLQEDMRIVLLRLNTLEARTVSQTEPTDSQSAELQIPLPSTKLLHDATKSCCRHVSTTW

>XP_005998554_ACBD7_Latimeria_chalumnae

MQGVAASAKPSVELHLSTEIPAAHCSSAMTLQAEFENMAEDVKKLKTRPTDDELRDLYGLYKQAIIGDINIECPGMLDLKGKAKWEAWNARKGTSKEDAMTAYISKAKELIEKYGI

>XP_014351918_ACBD6_Latimeria_chalumnae

MASFPGRLEREEGTEEAGGDSSSDSALGDAEPQKEETELQAEFERAADHLRGLLASASKEELLYLYARYKQVKVGKCSAPKPGFFDFEGKQKWEAWKAIGDTSPKQAMQEYIATVKKMDPNWNPKVSEKRGKDQILGFGGPVVSSLYQEEMIREEDKNIFDYCRENNIELVTRAIRSKKVDVNLKDEEGRVLLHWACDRGHKELVSVLLQHEADINSQDDEGQTALHYASACEFADIVELLLKAGADPSIRDQEGCLPEEVTESKYISSLLRQHAASKG

>XP_006011704_GCP60_Latimeria_chalumnae

MMAEVSSERLEVSIDGLTLSPDPEDGAGDRAASAGRGPEQNSLPAAVTQEKEERPAEEEEEEEESREAEEPVSMERKWGFSLQELYGLALKFFKDKDGKAFHPTYEEKLKLVALHKQVSVGPYNPEACPEVGFFDVLGNDRRKEWAALGNMSKDDAMVEFVNLVNQCCTLFSPYVTSHKIEKEEQERKRREEEERRRQEEEEQERLRLEEERKRREEEEKKQREEEERRRIEEERLRMEQQKQQIMAALNAQTAVQFQQYAAQQYPGNFEQQQVLIRQLQEQHYQQYMQQLYQVQLAQQQAALQKQQEAAVVASVGAAMVNSAVPGESPAVNGQANAHTDSPEKELEPESSEEVLENGPKDTTPIIAAPSMWTRPQIRDFKEKIRQDADSVITVGRGEVVTVRVPTHEEGSYLFWEFATDHYDVGFGVCFEWTDSPNSTVSVHVSESSDEEEEDEEHPSDEEKAKKNANKPQVDEIVPVYRRDCHEEVYAGSHQYPGRGVYLLKFDNSYSLWRSKTVYYRVYYTR

>jgi|Brafl1|224202|ACBD5-B_Branchiostoma_floridae

MAAPKARFDAAVKVIQSLPKNGSITPSHETMLTFYGYYKQATIGPCDISRPGFWDVVGKAKWEAWNRLGNMPKEEAMDNYVDTLKKIIEALPQDKEMQDFMHVLGPFYELVD

>jgi|Brafl1|101421|ACBD5-A_Branchiostoma_floridae

MAAPKARFDAAVKVIQSLPKNGSITPSHETMLTFYGYYKQATIGPCDISRPGFWDVVGKAKWEAWNRLGNMPKEEAMDNYVDTLKKIIEALPQDKEMQDFMHVLGPFYELVDLESPYQAGKSSLDQSEPSISDQPVLAIKENGEIPTNHVGESETLDDLQTNNNKITEKSTENSEENETEVTNQMQDRETGNTGVQEEETNHRGGDVSTNHREEEVVTNQREAEALTNHKQAQGDSSQSEDREVPEVEVEEVKEEVQPGKGIYDGRSPYGNQVTTPPVTKVMLMEIPTPPVIIDTPLPAKHHGKGPNLPDEDGREDSLPRRLNGHSYPAHHVNSDSDTDSELYQDTVDTPAIPDEMPGLISRQAGATSPSSQSPEGLPRSNSFVTQAEIHHDSNGLPSHGSMQQTEGMAPFSIGSPLANDSSGMEDSHNSSLSMSPLVGQGRSGDVERRLELESPAVIGRGGGEMGGQGKGQGGQMGSRRQSGDSTSSGGRGARDRGGTPGRRGAGGYYGGGGGDDGERGRGRGQGSSDVNEHIAVALDRLQQDMNFVLARLSTLEALSTSQARGGIVAPAVQAVQPQQQQESSWWPFSNLSGKTAFFILVWPFIVNWLIKLYLKKKSKAHRRSPFM

>XP_002594078_ECI2_Branchiostoma_floridae

MGRTAFGAHDAAFEAAKERLNTLKEEPDNNVKLQIYALFKQATKGPCNTPKPGAFDFVGRAKWQAWSGLGDISQDEAQKQYIDIINGLAGEESPAETEQAGEEASSYKEIKVTKENKVCSILLNRPAKKNAITWLMYNEIVQALDDASKDDSVTVAVITGAGDYYCSGNDLGNFMNIDPKDMPKMARDGKELLRRFVTAFIDFPKPLIGAVNGPAVGVSVTVLGLFDAVYATDKATFHTPFTELGQSAEGCSSYVFPKLMGNTKANEMLLFNKKLTAHEACERGLVTEVLPHDSFQKEVQTRVEYVAQLPPQSVREGKKLVRDQEREDLHKANEKECEVLEGRWLSEECVRAIMSFFTKKARL

>XP_002592536_ACBD6-L_Branchiostoma_floridae

MAASADDSSSNLADVFQQAVDHVATIAPQLSADQLLYLYARYKQVNVGRCNTAKPGFFDFQGKQKWDAWNSLGGMAQDQAMTEYVDGVRKADPTWEPREGGDSKKTSFMGPVVSTMSFEEEYISDKNKTAFDWCKEGNTKQMARLLSNGDADINQQDSEGLSLLHWACDRGHEDVVRELLKHKANINIQDEDGQTPLHYAATCEFLSIVQLLLDSSADPSIPDSEGSLPLDSTDSKQIKELLTQWTVKSNT

>XP_002602095_ACBP-L_Branchiostoma_floridae

MSEADFEKAAEEVKNLKSSPTDEEKLEIYSLFKQAKIGDVNTARPGMLDFTGKAKWDAWESKKGMSQEDARAKYIAKVEELKGKYGV

>XP_002586469_GCP60-L_Branchiostoma_floridae

MAEERDDVSRLDVAVDELSLAEKENGSPEGEEVTANGGEDGGLFEEKWGFPTEELYKLALTFFKEKEGKAVHLTYGDKLKLVAYTKQVQVGPYDAEKMPPVGYFDVIGNDRRKEWQALGDMERHRAMCEFCLLLDNLCPTFQPYVEAHRRDLEEKERLKREEEERLQREAEERERQRLEEEARKMQEDERIRQEQQKKYKHTDRRCFLEVMSASSIMNVLNQQTAAQFQQYAAQQFPGNPEQQQLLIRQLQEEHYQQYMQQVYQQQMQQLQQQHLQQQQQQAPPLNNHVDVEAGQGSAAAPTLDSTGDGEEKIVENGPEEEYPSIAAPSMWTRPQIQEFKEQIRKDKDSVITVGRGEVVTVRVPTHEDGAYLFWEFATDSYDIGFGVYFEWTIAPSNAVSVHVSESSDEDDEEEDEGQDVEKGARSDRPPTDEIVPIYRRDCHVEVYAGSHMYPGRGVYLLKFDNSYSLWRSKTLYYRVYYTR

>XP_001122639_ACBD4-5_Apis_mellifera

MTTEEQFQAAVNVIRNLPKNGAYQPSNEIMLRFYAYYKQATEGPCQQPKPAFWEVVKKAKWDAWTRLGNMSRTEAMNNYVEELKKIVETMSYTDKVANFLDSLDSFCESVPPEDLELLLGPVLERMRSQPGMPLSGSPLASRETSPHRVCNITRHIASSLETSPATSHGTSPPDTDGEEEFIDTVESAPERIQKDTIKTSNVIQKMTNGLNISTEKINELIPQKENSSELINRYTNINGYTEPLTENKQERNKQRTKKDEKSNDEFFNQIATTMQNLQRDLDRITTRVRSLEGQTLQALAPSTVHTQPTSYPEWWPLPECSPRLFTILMIWPFITQFLISLIHRYRQRRL

>XP_003250509_ACBP-like_Apis_mellifera

MTLDEKFKKAAEEVKELSAPASDADLLELYSLYKQATIGDCNTSKPGMLDFKGKAKWDAWDKRKGMSQDAAKEQYIHKVEELISIIGKNTTTTGSTETCECIERSKVS

>XP_394773_GCP60_Apis_mellifera

MAGTNGDIFSVGKKIEKLTISGEESTKNEDDSENGKKLVPRLWGFETKELYKIAVNFYKEKEGKAVHLSYEDKLKLVAFTQQVTHGKCTAENAPPLGVLDVIGKDRRLAWQNLGDISKEQAMEGFIVLLDKLCPLFRTVVEAQKRDIEEKLRLKKEEEAKKLEEERRLQELNEQKKKEEEARLKEEIQRRQIQDALNQQTYYQFKMYAEQQYPGNPEQQGVLIRQLQEQHYHQYMQQLRQNQLLIEDQTPEMTTATIENTEKEEEKETSETAQLNEEDSDDLQDYPPIAPPEMWTRGGVEEFKNTIRREDSDAVIKVGHGETVTVRVPTHEDGSYLFWEFATDNYDIGFGVFFEWSKPETNQVSVHISESEDEDEEDDDYETRDDLESGIANGSIPSDYKPSPPPTSMVVPTFRRDSQDEIYAGSHRYPGQGVYLLKFDNSYSLWRSKTLYYRVYYTQSGYVQN

>NP_608729_ACBD4-5_Drosophila_melanogaster

MAAIEERFQAAVNVIKGLPKNGPYQPSTSMMLKFYGLFKQATEGRPDVDKKPGFWDIVGKAKWQAWNDNRHLTKEEAMQRYVESLQEIIETMSFTENVQNFVGSLDSLGNISLDELELVSPGMKELAESHPNSPFHSRTNSPQHGSSCNGEPEPEPQATSTAPLATSETIKENGHSSPPLTNGYGPKPTASYTQHDTHNFTSNSSVAIVDPSDDEYDDPYDLSHELTQAIGQNTDLLRQIQAAITRMNSDVGAVQQRVRSLEQSLNELRSGQKAAKGTVAQPRSLPAWWPFRNISPLWFAVLILWPFLVRRFARMLQSNPQRRH

>NP_609187_CG8498_Drosophila_melanogaster

MSELQEFNQAAEDVKNLNTTPGDNDLLELYSLYKQATVGDCNTDKPGFLDFKGKAKWEAWNNRKGMSNTDAQAAYITKVKALIAAVGLKS

>NP_001027085_ACBD6_Drosophila melanogaster

MSDSDTDTVDELFHLATEHVAKQSNSIGSADLLIFYGYYKQATNGPCKEQSPGLLQLQAKSKWQAWRNLGTMSQSAARQAYVQKLQELQPNWRSRRNPGWVVHSIESVPLEDQRLDSEKTLFDHVKENNLDRLRELLQPSDLVKLDEHGMALIHWATDRNAVEIIQFLVRSGASVNQRDAEQQTPLHYAASCGHLEALQCLLELHASLELRDSDGQTCYDVADDEQICQVLQTERERLAGSAS

>NP_648081|CG8629_Drosophila_melanogaster

MVSFEEAAELAKNFSKKPTDSEFLEFYGLFKQATVGDVNIDKPGILDLKKKAMYEAWNAHKGLSKDAAKEAYVKVYEKYAPKYA

>NP_648083|CG8628_Drosophila_melanogaster]

MVSFEEATELANKFTKKPTDAEFLEFYGLFKQATVGDVNIEKPGALALKDKAKYEAWSSNKGLSKEAAKEAYVKVYEKYAPKYA

>NP_648255_CG5804_Drosophila_melanogaster

MADFNAILEKTKAFSKKPPTEVYLEFYGLYKQFQEGDINIEKPADAEGAAKYDAWLSRKGLSVDDAKAAYVALYEKYNPIYG

>NP_648082_CG15829_Drosophila_melanogaster

MPTFEEIVEKAKNFKNLPSKEEFLEFYGYYKQATVGDCNIEEPEDEEKKARYNAWKSKAGLTADDAKAYYIEVYKKYAPQYE

>NP_729218_ACBP_Drosophila_melanogaster

MVSEQFNAAAEKVKSLTKRPSDDEFLQLYALFKQASVGDNDTAKPGLLDLKGKAKWEAWNKQKGKSSEAAQQEYITFVEGLVAKYA

>NP_608348_GCP60_Drosophila_melanogaster

MASGTSGTSSSETAQSTDMAPSSAEKWGFPLIELYRLAFTFYKRNSGKAIHLSYEDNLKLIAFKQQAALGPFNTSRAPALGVLDVIGRDRQQHWQLLGEITREQAMEGFVDLLDTMCSAFRPYIEAVRQDRDETLRKELRLMEEKKEARERQENAQQELLEEGYKEELQRRQLQDALNKQTYQQFKLYAEKQFPGNPEQQAVLIHQLQREHYHQYMQQLHLQNQNQNQNQNTEDKGHQEAEHVPGNNNNSSTDLPNAMEGLKLGEVETQQHQQLAEQQDGHVEQTLEGVGQEQHGEEEYDDYVMICPAKIWTRPDIEQFKTEVSAGDGDGVITIGHGDTVTVRVPTNMNGKCIFWEFATDSYDIGFGIYFEWAKPVTNEVTVHVSDSDEDEDCVDEDYLSTTEDLESGSLSQERGAVNNPTAAPKAPISIIVPIYRRECYNEVYVGSHSYPGEGVYLLKFDNSYSIWRNKTLYYRVYYER

>XP_001811313_ACBD4-5_Tribolium_castaneum

MTTEEKFNAAVNVIRSLPKNGSYQPSNDLMLRFYAYYKQATLGPCTGRRPAFYDVVGRAKYDAWKSLGEMSKSTAMAKYVDELHTIVETMSYSDKVANFLEAPTNEIECVNMIMDDLELVAGDVLEKVRSQPNSPLASREASPIRMFRSRDVSPVSTPSSAPEESDHSDDEYIDTIEFPESKKVNSEQMSNGYIPKEHVSRSRTKPQTSVDISQEVSRAVQSLKADVERLTSKINTLERSNVAVSKHRSTGLSLEAIAFIIAWPFVASFILNRYVFNRK

>XP_970549_ACBD6_Tribolium_castaneum

MATSLDSFNDLKELGIDCEDSDSLTDAFNKAANHLPNLLPNVDNHTLLSLYGYYKQGSQGPCNTPKPSWFDMKAKSKWEAWSKLGDMPQNKAKQIYIETIKTLDPTFNVPEKESWVSVSVPQNLEGFKEAKEKTVADFVKEENYLEVAKLLESPSIAKDLINKLDEEGLGLIHWAADRGSVDILELLFKCGANVDLQDSDGQTALHYASSCGHLECIKILLTCNARRDIVDGEGLSPEDVACDDAVKELLVNFK

>XP_974824_ACBP-like_Tribolium_castaneum

MSLDERFKKAADDVQKLKSKPSNDDLLEIYALFKQGSVGDCNTDRPGMLDLKGKAKWDAWNGKKGMSQDKAKEEYIAKVESLIQSIGLQ

>XP_974813_ACBP-like_Tribolium_castaneum

MSLDEKFKSACDQIRQFTKRPSDSDMLEVYSLYKQATVGDINTPKPSEAKAKAKWEAWSGKKGLNANVAKEQYVAKIKALAPTYA

>XP_972065_GCP60_Tribolium_castaneum

MAAESDSSQLLENFSKLEVKTAKRKSSVNNASNDTLEYGLPLTEVYKLAFSFYKEKEGKAVHFSYEDKLQLVAFSQQVLHGPLSEAINKLPPLGTLDVVGKDRRLAWQKLGKLSTDQARAGFVELLSRRCPLFSAYVEAHRREKKEQERKAKEEEKRRLIEEEEKQKKEEEAKLIQEQLTKEEAIKRQIQQALNEQTFDQFRKYAEQQFPGDPEKQGALIRQLQDQHYIQYMQQLQAAQRGEQIKSVDEEKTDPDWNNQSDEINLNESSSNSLIPASMWTRSGIDEFKQTVAQAEGDGVVRVGHGETVTVRVPTHPHGTRLFWEFATDHYDIGFGVYFEFGTPTSDQVSVHVSESDDEDLEDIEEEIYDEEAIQTGDLEAGSVAVNGISKPLLTEIVPVYRRDCQSEVYAGSHQYPGQGVYLLKFDNSYSLWRSKTLYYRVYYTQ

>KDR11486_ACBD4-5_Zootermopsis_nevadensis

MNEAHSIHEQGSYQPSHELMLRFYAYFKQATEGQCVAPRPAFWEVVKKMKWEAWNKLGNMSREEAMNNYVEELKKIVETMSYTDNVATFLGSLGSFYDSVPVEDLELLVGNVIERIRSQPGSPLGGSPLVSRESSPNRTSAISAANGRITSSLETSPASSYSASPLPPNPDDDEEEEEFIDTVETEPERIVKENSTVGRSLSGSKKPNYYSNGVPMIPKNDSTEIIPNGHINGVAIKNHVNGHHSHIAEDAVPPAMDGLGEEGGRETVNSRGRSRERHSPRVHRAFPVPTRISQTELQIAMVKCCPNSAVMTPPRLSESLPVNDISDQIREAVLHLQRDLDRVTARVRSLEVSALSGSLHNSSVWSNERHPSWWPFPDLSPRTFMFVILWPLFVHAVILWLRKRRSARAQLL

>KDR10827_ACBD6L_Zootermopsis_nevadensis

MLELSDMFERSANHLKSIIGKLEPGHLLELYGFYKQATVGPCNISKPRWYEMQAKQKWEAWQALGDMSREIAMKNYVQLISELDHGWRDANLNSEGSGISGRSGWVTVSCMSSTDEYLDDADKTIFDWVKEGNAEKVWEFAQKSSLADCNIIDSEGMGLIHWAADRGNIAMLKCLIKDLMADVDLRDGDGQTALHYAASCGHADVVKFLLDNGANPNVADTEGMLPIDVASGSEILESLNKAD

>KDR20996_ACBP-L_Zootermopsis_nevadensis

QKFNEAAESVKNFKKRPSDDELLQLYALFKQATDGDNHKEKPGMLDLKGKAKWEAWSDKKGQSKEVAMEAYVALANKLEARYA

>KDR10690_GCP60_Zootermopsis_nevadensis

MTMAADREACRVYYNGDRLSIGSEDSLNHSNIEGDFNKENEESIQKWGFRLKDLYRLALRFYKEKEGKAVHLSYEDKLKLVAYICQVSHGKFNPDTLPPLGVLDVIGRDRRLAWQALGGANQYEAMAGFVELLDKLCPMFRHFVEAHKWDVEERKRVAREEEAARQEEAARLRLEEEERQREEEDRAKQEAQRRQIQEALNQQTYHQFKLYAEHQYPGNPEQQGVLIRQLQEQHYHQYMQQLYQQQCCKQKLQQFSTVEGPMDETAHLLSNGGVDALERDMESEEDGTQGEFPTISAASMWTRKDIKEFKDSVREEGGDAVIKVGHGETVTVRVPTHEDGTCLFWEFATDSYDIGFGVYFEWTTSASSQVSVHISESEDEDDDDDDDDDDDGEDLEGGSGLAGTKATDLSNRPPLSVIVPVYRRDCQEEVYAGSHVYPGQGVYLLKFDNSYSLWRSKTLYYRVYYTR

>KZS12634_ACBD4-5_Daphnia_magna

MSTEDAFRAAVNIIRSMPKDGPFQPSPDLMLRFYGLYKQATEGKNNQPKPSFWAVITKAKWDAWKCLGNMPKEEAMKKYVDELKQIIETMSYSDSVANFMHVLGPFYEAVYDGQEPENLRRNPSPQLSEGMDKDQQEDESGYSSSINGTGAEETESADLETENNNKPVVVEEEDESVEEEGEPVLVTTLSPATSSASLYASEKFYSDAESDDEYKDPLPDFYPQSKSLHSAMSKSSSADSGEQLHVTMAIQRLQADLQSINSRLEALELHRKEVTLMDNGRQATTKHSTVEQVGSFAVTYGSIPSVNQLSLPSWFPFKNVNVATIAVILGWPVLAQYLFARLHHRHSAV

>JAN53111_ECI2_Daphnia_magna

MIIPTAANSFRSQCLAAMLLKRSFVNSPNQRTFLAPANGIKHMSTSISQQFDEAQKRLQTLKTSPGNEAKLKLYGLFKQATAGAVNTKRPGMTDFVGKAKWDAWNSLGSMSQEEAKNKYIEFVDSLVGPANSTESLNVVESSSPGFDVTIDGKLRIITLNKPTTKNAFTLGMYVGFAKLLKEAAEDPNTTLVAVTGAGNIFSSGNDLTSFTSFTGTMREAAEEGKRCLSIFVGSLIDFPKPIIGVVNGPAVGIACTILGLMDVVYATDRGWFQTPFSALGQSPEACSSHIFPKLMGSLKANEMLFFNKTITATEACKLGLVTSVLPDANFQSEVWPKLKEWSELPQKVSDRRKHFIDSAIKKLTSLFQSLVHSKELSRQFDRELLHKVNAAECDRLLERWQSSDCMEAVMKFFSKNAK

>KZS11387_ACBP-L1_Daphnia_magna

MSLDEKFNKAAESIRSMTTSPSDDEMKEIYALYKQSTVGDVNTARPGMLDLKGKAKWDSWESKKGMSADEAKEAYVTKTEELVAKYSASSA

>JAN71011_ACBP-L2_Daphnia_magna

MVVMAYKSVDANRRPQKFTVDYQERLPLRSTMFWGVLIETSSNDKQLQYFTFFALLSFPFIFFSQLTHIMSLDQKFIKAAEDIKALPATPTNDELKDLYALFKQATVGDVNVARPGMLDLKGKAKWDSWESKKGMSSDAAKEAYIAKSAELVAKYIS

>KZS16298_GCP60-L_Daphnia_magna

MDINSIGTDSSKPTHKHFFFSCPDNPAETLAVIIPEQLHASYGMYTWPCAPVLAWYLWSQRVGLVGKHVIELGAGTSLPGVVAAKCGANVTLSDCSRFTKCLENCRISATTNGVGDKVNIIGLTWGTFEPQLLKLQPVDLIISSDCFYDPAVFEPILVTVSYLLNKNPSASFVCSYKERSSDWSFEPYLSKWKLCCRTLEVDSAFHEASVTVKQFLNVDKATMLRLYGLYKQATQGECNISKPGILSYTARQKWEAWNSLGSTSVADAKSQYIELVNSLTPNDKQEAQSVNKASAFGVSVSCMAKTEQELNDSDKTVFDWLKEGSLENVSSSLTTNPSLISYRDESKMALIHWAADRGDIAMIQLLAKKGADVNMTDGDGQTPLHYAFACGHDECIRLLETLGANRNVRDSNGMVPSDLAES

>KZS16298_ACBD6-L_Daphnia_magna

MDINSIGTDSSKPTHKHFFFSCPDNPAETLAVIIPEQLHASYGMYTWPCAPVLAWYLWSQRVGLVGKHVIELGAGTSLPGVVAAKCGANVTLSDCSRFTKCLENCRISATTNGVGDKVNIIGLTWGTFEPQLLKLQPVDLIISSDCFYDPAVFEPILVTVSYLLNKNPSASFVCSYKERSSDWSFEPYLSKWKLCCRTLEVDSAFHEASVTVKQFLNVDKATMLRLYGLYKQATQGECNISKPGILSYTARQKWEAWNSLGSTSVADAKSQYIELVNSLTPNDKQEAQSVNKASAFGVSVSCMAKTEQELNDSDKTVFDWLKEGSLENVSSSLTTNPSLISYRDESKMALIHWAADRGDIAMIQLLAKKGADVNMTDGDGQTPLHYAFACGHDECIRLLETLGANRNVRDSNGMVPSDLAES

>KZS07345_ACBP-L3_Daphnia magna

MSLDEKFNKAAEDIKALTARPTDDELKEIYALFKQATIGDVNVARPGMLDFKGKAKWDSWESKKGMSSDAAKEAYVAKTEELLAKYSA

>JAN37855_ACBP-L4_Daphnia_magna

MTTSPSDDEMKEIYALYKQSTVGDVNTARPGMLDLKGKAKWDSWESKKGMSADKLRKLTSPRRKSWWPSIVHQVHXSIA

>XP_018019205_ACBD4-5_Hyalella_azteca

MTTEEKFQAAVNVIKGLPKDGPYQPSNEMKQIFYGLYKQATEGPCTEPKPPFYQVIAGYKWRAWNSLGNISKEEAMEKYVAELKKIIETMSYSADVASFMEALGPFYDYVEMPSKEVSIQSNTIIQNTAFHQHEQPAPESDSFRQDIAADATGPLQYEVAEKLVTNGIHEHKVDNADAVVYCHEQDKWNKVPLKNGFISNGDVKRNGVVSKSYFMGPNSGELPSVASSAQNNLSSAQRQDGVRPNKSAAVYSDTDSEEEYAMPPEELSGDECVVINGNGLPNKVTNRKGFPTLNCNSREQHHPSGLTGDESASDSSSICELNNHIPTGTDQQFAEVSAASPASAQCTSRGRAWCASVTNKGVAPQSLPGLVTYEQGQGGGGGDDVVIVLRQMQGDLQAMTRCLQAHLNDISQCLVALNRTSIALQAHSDGAAGGGGGGGGSSPDSEVAQRRQRQWWPFPELSPRSTFWLLLWPVLLHVALSAAKLAIKIRRRRKANVLNFRPAS

>XP_018023004_ACBP-L_Hyalella_azteca

MSTETLFQEAAEKVKKLAKQPTDDELKEIYGLYKQATIGDINTERPGMLDFKGKAKWDSWESKKGMTKEAAMTAYVAKVEELVAAYGLQ

>XP_018015935_ACBD6-L_Hyalella_azteca

MSEINESPVENIFNAAAKHFPSLISTLPKERLLFFYARYKQATEGDCNVAKPGFLDQSGRRKWEAWKSLQGTSKAKAMEEYICGVKELDPDWREDATNTSGWARVSRMPAPEDATITKFVEAVQENQLDVLKQLDLATEVQKRFDEGMTGLHWAADRGHRDVAELLLDRGADVNAQDEGGQTPLHYAASCGYEALVCLLLSRGADSTIRDVDQLTPRQVADDAAVASLFPQ

>XP_018025342_ECI2-L_Hyalella_azteca

MVAKSFLAASIPFRRCFFNSTANHVVNVQACRNLGPSFIGGAPRYMSSSIAQQFEEVKHRLASLKEDPGNEVKLKMYALYKQGMEGKATGKRPGVMDFVARAKWDAWNSLADMSKEEALKAYLAIVDELSAAQGATSTEEPSILVTVEDGLRIIKLNRPKKKNALNPEMYFRWTELMHEAAKDDKTVLTAITGAGDFFCSGNDLGNFMNIPPGGEAELANRTKEFLYQFIDAFIEFPKPLIGVVNGPAVGVSVTTLGLYDAVYSSDQAWFQTPFSQLGQTAEGCSSYVFPRLMGPGIASEMLMFNKKLSAHEAQRYRLVTEVFPHDRLQQEVWPRLQALAKLPARCLIYSKELTRAADKEILKKTNRAECERLCERWQSEDCKNAIMNFFSRQRK

>XP_018011999_GCP60-L_Hyalella_azteca

MASDTSDGANKLSNGINNLNIMGTSENESISNHHPQNDVENKTEVDLESVKAEWGFDLQELYKLVVKFYKEKSGKAIQLTYSERCHLVALTQQVIHGPYDDAAAASQTLGIMDVVGRDRRLAWQSLGKMSSDEAKLEFIEQLHRLAPTLKPYIEACLADHLHHLQLQEEQRLQQEEEHRLQLQKEQERIQQEEIRRQQEQTKREIQAALNQQTFAQFRSYAEHQFPDNPDQQAVLIRQLQDQHYQQYMQQVYQQQLQRQQQQAALLQVRTRRCRYERDDAEAGDVIAAAAMWTRRDVAEFKRAVQAEGPNTVLRVGHGETVTVRVPTHQDGSCLFWEFATDAYDIGFGLYFEWTAAEDDAAVTVHVTESDEDDDDDDEDGENCTGDADVERGGGKTGGGRRGGQSRVTGPPQSVIIPVYRRDSHQEVYAGSHTYPGTGVYLLKFDNSYSVWRSKTLYYRVYYTQ

>XP_015906206_ACBD4-5_Parasteatoda_tepidariorum

MDISPVVDRFNSAVEIIQNLPKSGCIQTSNDTKLKFYSYYKQATAGPCELSKPGFWDVVNRAKWDAWHKLGNMSKEEAMKGYVQEFVKVIQETPLESLDDPSLVNDFIGMMGPYIEYAPEEIKKKHLKINGKANGLNNNSEDYVEDFSNSRLPQSSNDYSNRMSDDGSENYASPVNGHSLNGGNESDSDIFSDTLEGVNEDDPEEVFASNSNPMSNNPMTQSKGPSIINVRGGDHRLGTRNGNSPANASGSRGNQSTQQSASSNWALQATSSSTYLGSAGGSRRSGGGLPTELASDVNEQLALAVIRLQHTMEQVVLRLDSLETLLTQRSNIPNQVAEKKSARWSIFGMSPKLAVLIFAWPLIVQFLIYYIRRRQRHL

>XP_015922765_ECI2_Parasteatoda_tepidariorum

MSIIFSSIRKLSSSLRPSIFLPYSKSSKSIYLLKSFSTSVRMNSSLTFQQACDKAKTLEKDPGNDVKLKMYALFKQATEGPCSQTKPSVFDMVGKAKWEAWNTLGSLSQDEAKSQYVSLINGLFDQKQQSEQSAEIQELKYEGIKLSVSGDITELRFNRPEKKNAITTKMYKDIALALKEASENDTAITILTGEGDYYSSGNDLSNFASVDQDISAAAKKAGQLLNEFVGAFIDFPKILVAAVNGPAVGIPVTLLGLCDVVYASDKATFQTPFSFLGQSPEACATYTFPKIMGYSKANEVLLFNKKFDAVEAKSMGLVSEIFPNDTFHEDVKRKLEFLSQASKQSMILSKELIRKFEKDTLHTVNDEECKLLVERWQSQDCMEALMRFFTRKSKL

>XP_015921159_ACBD6-L_Parasteatoda_tepidariorum

MEETDFAGDENSVEETFQRAIDFVTGNASVMQQDDLLYLYGRYKQALCGPCKEPMPGILKFKSRSKWTSWHNLGNMSKEEAMQQYIDCVTNVSSDWDSNPKESAKKSRIGPVNSSYLKTDPEIKEELKTAFDFVKEGNLNQIKLLLSSNSAIKDSLDENGLTLLHWACDRGHVEIANYLVSSCGIDVDCQDTDGQTPLHYASSCGHEEVINFLLKNGASVDIYDVDGLSPADVAYNTSIKEILLNKHV

>XP_015907443_ACBP-L1_Parasteatoda_tepidariorum

MSLEEKFTKAAEDVTKLKTKPTDEELLEIYALYKQASIGDVNTTRPGLFDLKGKAKWDAWNSKKGMDQTEAKEAYVTKVNHLIEIYGLA

>XP_015904839_ACBP-L2_Parasteatoda_tepidariorum

MSLDEKFNTAATSVKDMKSRPSDSELLELYSLYKQATCGDCSADKPGALDLKGKAKWEAWTGRKGMAQDAAKEAYVTLANTLITKY

>XP_015930884_GCP60-L_Parasteatoda_tepidariorum

MDASVNGDPVNVCVQNNINSIEKDTLNSISPSNDSESSDEFLETWGFNTDELYSLATKFLKEKEGKAFPLSYKDKLSLVAYTQQVLHGKFIEEKYPAVGYLDVIGRDRKLAWQSLGDMSKKDGKAHYIKLLNEVCHLFRPFVLAHKCDLEEKERKRIEEEEKRRLEAEEEERLRLEEEEEERLEEERLREEKERQKQNNQKRAIQEALNRQTFQQFKAYAEKQFPGDPEQQAVLVRQLQEQHYQQYMQQVLQEQLSPPVLSHKSEVKMTQVPQDNGLQPTRESSPEPSEDEIADPLPVAAAHMWTRKDITQFKEAIRKEGGDGIIKVGHGETVTVRVPTHDDGSCLFWEFATDSFDLGFGVYFEWTRSPDAQVTVHISESEDEEDDDDELEQVRSDIEKGVTLQDKPPLSVIIPVYRRDCHEEVYAGSHNYPGQGVYLLKFDNSYSLWRSKTLYYRVYYTR

>NP_499531_MAA-1_Caenorhabditis_elegans

MSVFETAVFIVQNLPKDGPIKTSTDEKLNFYALFKQATHGKCDLPKPSFYDIQGVYKWNAWNKLDNMTMDEAKQAYVDSIVQKIREVQKEYKTEEWMKGDTYELLAPKFEVLGVLEGKDQAVEHPKKTENKETENETDNVETPNSSACILSDNEYADAIDDEIQSRSSSFTEPHNSFNRRISRQSSLKSSCHRLEKELKVITESIDKLGKAVEERHNLLIGLMKKATVYVLVPRVTSWKTIIFFVFWPFVTKFLLRWLRRFLRLLM

>NP_498609_ACBP-4_Caenorhabditis_elegans

MGKSLDEQFEAAVWIINALPKNGPIKTSINDQLQMYSLYKQATSGKCDTIQPYFFQIEQRMKWNAWNQLGNMDEAEAKAQYVEKMLKLCNQAEAEHNLMEFLSDPTIADLLPKQNQLREHFATLGRTTVKGFEGETVEINGVSISF

>sp|Q20507.2|ACBP3_Caenorhabditis_elegans

MSLQEKFDAAVEIIQKLPKTGPVATSNDQKLTFYSLFKQASIGDVNTDRPGIFSIIERKKWDSWKELEGVSQDEAKERYIKALNDMFDKIAEELDVAAWLEQIDPVIKTNLALIGK

>NP_496330_ECH-4_Caenorhabditis_elegans

MLRNLTLAARSVLYNPQVSVRSFSAQADFEKAQKNLKTLKEEPDNDVKLQLYGLFKQATAGDVQGKRPGMMDFVGRAKYDAWNTLKGQTQDEARANYAKLVGGLISEEASAAPEPTGPSIEGLENVDGLSVTREGKVFKIALNRPKKFNALTLEMYQGIQKALEVSNNDKSTSITVITANGSYYCAGNDLTNFKAAAGGTKEQIADMANTAKVIMKDYVNAYINHEKPLIALINGPAVGIAVTVLGMFDYVIATDKASFHTPFAPLGQSPEGVSSYTFPLIMGSLRASEMLLVCKKISAQTAKDYGLVNEVVPDAEFQSHAQKTVEAFSQLPPETLRINKKLLRSLHKEKLLEVNNIEADQICERWQSKECHQAIAAFMTKGAKK

>sp|O01805.1|ACBP1_Caenorhabditis_elegans

MTLSFDDAAATVKTLKTSPSNDELLKLYALFKQGTVGDNTTDKPGMFDLKGKAKWSAWDEKKGLAKDDAQKAYVALVEELIAKYGA

>NP_499817_ACBP5_Caenorhabditis_elegans

MIIAWAVSSFQDHPKNENQEEEPIDTLLEAKFDAATTRLPGFLTKIDQKTILKFYGLYKQAVEGPADSKKGPYWFETVARKKFNSWLANSQMSRSRAMEAYCELMAQLDTSWDPDAETVKKSGLWEKMPSTMGVIEPEMFDDAVVHPPTKLETETEKKWFAAMRVSDIDTMRTLLRNEPEIIEAKDQYLAMTALLWATDLGCDPVVQFLIENNVDVNAVDGCLQTALHFAAQCHRPLLAELLLQAGADRSALDADGLTPSECCDDEDLRKKLTP

>NP_496552_ACBP6_Caenorhabditis_elegans

MPISELEKKWTGLTFEIAAEEMRRLKSEPTDRERLKLYALYKQALHGDIPNEDVYPVPAGDEVGRKKYAAWKSQKGANSEKCRADYVAIAEEMIKKYGRNIVRCKWNSEVWSVDY

>NP_001033359_ACBP7_Caenorhabditis_elegans

MENALEDFKRIRAQFVQRKVLKYEFELFTKFENHTRHVWGLYQQAIVGDVNVPKLNYMEIDEGEKSWMWRWINGNEKWHAWNKCRGLTKEEASEQYVEAVQKLKIDIQQLLINWKIEISNEPTAAMNNNINNIA

>NP_001041025_Y41E3.7_Caenorhabditis_elegans

MADENHVRLSEVQLIQSEFGHSLEECYKLAVQYYKKEHVGKQEPVGYEDRIKLLSLSKQVQHGEISDEFDNAGWLDITGNDVNKAWRELGSLSRDEAMASFVFLVDRVCPPFKGFIADKKAIKDAELKEFAPQVTEQSAQPPSLQQVDQRLFEDQRKQIQEALNAQTFHQFSAYAQEQFPGQPEQQTTLIRQLQEQHYQQYMSQVYAQQSTTPNGAEMNPEESHQHQIRRDDDSDVSDDEAGEDLPSNPAISPASLWNRQDINEFKANIKKDGHEGIIKVGHGETVTVRVPTHENGSCLFWEFATDHYDIGFGVYFEWTVADSNQVSVHVSESDDEEDYDEALEAEQAEAGGGGGGAAGQGGPGDVEAGAMQTRRVDPNKPRQDEIIPVYRRDCHEEVYAGSHRYPGRGIYLLKFDNSYSLWRSKTLYYRVYYSK

>XP_780031_ECI2_Strongylocentrotus purpuratus]

MAALFSLKCGTRRLFNICNTCTTRLHAPVLKRQIQNTACMMSFSDAEFTAAKDRLNSLKEDPGNEVKLQIYALFKQATAGTCNTPKPGAFDFVGKAKWSSWNSLGNMSQDQAKEKYVGIVDDLVAQEGGEAETASTTQGSLSFTGLKYTVDNGVATITLNRPNKKNAVTTEMYSEWTAALKMAGEDDRVVLAVITGAGDYYCSGNDLNNFMKIDPSVLHEESVKGSDLLEIFVNGFIDFPKPLICAVNGPAVGISVTTLGLMDVIYASDKATFHTPFAALGQSPEGCSSYTFPKIMGTAQANECLLFGKKLTAQEAFDRGLVTEVIPDAQFRETVDKKVKEYAQLPRNALRLAKNLIRETEKERLHKVNRAECDLLVDRWTSDECTQAIMNYFSKSKL

>XP_783245_ACBD4-5_Strongylocentrotus_purpuratus

MASVGEKFDAAVTVIKKLPKNGPFQPSFELMKRFYGLYKQATEGPCHETKPAFWNVVQTQKWQAWSVLGSMESEEAKRLYVEELLQTILDNSKARDIPHMYKIIETMPQTDEVVTFLKVMGPFFELVHEGEPEHVPASSRLQVTKVNGHVNGGVKMKETKVDERGDQARTLQNGDLSDVSDYTNGYLEDDHDLIERSKGKTEQLESSSTEAEVNGHSEEGEVTEEEMRESSYVRVVSTHQGENSAELSHPILISKSQLEAAEEHLPSTSESDSDGDEFCDSVDEPLISRISVAHISKTDSLPSVMEEEEDQRKHLTSTPFSGDHAVVTRAEVHNDVDAVILPQYTSSSSSAPTISTPSMRGGGEVTEGSKGHQGQDSALNQSGQGHLSGYVSSDSFQNLRSSGSQDSSDGGGRRREGGEGGAGRGTPGGAGSPLRSPLPDGMVEQITISLERLQRDMNSVLVRLNTLETIAISRHQLETQQAYMREGITLPKESAPIRTPPQPATPSWWPFSTLPVRTVFILLVWPFIVQWILRFLARHRRARPPPR

>XP_784299_ACBP-L_Strongylocentrotus_purpuratus

MSDAFNTAAAEVKDLATSPSNEDLLKIYSLFKQVTVGDCNTDRPGMLDYKGKAKWDAWNGLKGKSTADAEKEYIELVGQLKTSCGMK

>XP_783927_ACBD6_Strongylocentrotus_purpuratus

MFNFQGKQKWDAWNALKDMSGEQAMMEYIEAMDEVDPDWSLKVKGQKSSSRGFGLGVSTMLVEEEEELSDQEKSIFDWCKEGDVDQVRLLLSNEGTNIEQVDEDGLTLLHWACDRGHLEVVTLLLEREVNVNALDQDAQTPLHYACTCEHLPIIEALVKHGGNVNAKDADGNLPADNTYRKDIHQLLAISRDVT

>XP_011664907_GCP60-L_Strongylocentrotus_purpuratus

MLQFCELLESRCPQLQPYIEAHRREKEEQERKRIEEEERRKREEEEREQERLEEERLRKEEEARQAERLREEQQRLQFKESVYQQLEPQLRPLAVEQYPHNPQQQQVFLQQLQEHYYQQYMQQIQEAQNQQQHNHIAPSLTMENQTEGVSPQDGTSPNKLAQDLSHLELGGEGDTAPPNGDINNIPLNSDEDQYPVICKASCWTRKNIKEFKKELVSEKESVIKVGHGETVTVRVPTHQEGKYIFWEYGTDSYDIGFGLYFEWTEAESNAISVXVSDSSEDEEEFAYEEGGGGKSDIERGTKDRGPPMDEIIPIYRRDCHQEVYAGSHKYPGRGIYLLKFDNSYSLFRSKTLYYRVYYTRDTQPGRTPPDAVET

>XP_002121967_ACBD4-5_Ciona_intestinalis

MADVESQFKAALEVIQNLPKDGPYKPSPNMMLLFYSYYKQATEGPCTKPKPWSFDVVNKAKWDSWNKLGKMGREVAMKNYVDELTKIIEVLPATDAASDFLQTLGNFYEVVDVRTAEAEKVKSLMKQRAKEELPNGNHLSNGDNDHCYITTNSIKNKQVTSDSEDEFCDTSPDVQDTLSHNSSSSEPSTLSHESYANQQFQDVNILKSSKQTPITHGGESGTHGLGEGKPLQHIKESVPPHSNHGSPPFEPPTGQGVPPSGSGGGGGSYNGDSDNRIQERIASVLLQLQRDMNSVLTRLTTLETITVARQDTHHCNHCCIHGNNPQLSNPSWWPFPGLHPRVAVFILIWPFLARIIVASIRRKFQRR

>XP_002124332_ACBD6_Ciona_intestinalis

MDFNSVLEEQFLAAANHLQSMVANHPEKASEADLLYLYGRYKLCTEGLCQTPKPGFLSFAARKKWTAWNNLSNLSRDKAMEEYVIKIKQLDPNWSQNGRKLTPSGMGVSVSTLNHCSRDEKDIIDTEKDLFDWCKEGSLSNISKNKPLTWPKDSEDRTLLHWACDRGHKNIVEYLIREGHDINCQDSDKSTPLHYASSCDREDLVDLLLHQGADPSISDADGDTSQDCCSSSKVKELYEEYFKNRKNNCTN

>XP_002126596_ECI2_Ciona_intestinalis

MLSSKVDGDFETAKQRLNTLKQEPGNDVKLQIYALFKQATIGANNTKRPGTFNFVGQAKWNAWNDLGPMSKDEAKEEYVKIVKDLSDAEGEAEEEVGDVSSSTDYQNLIVTKQNNYTKIVLNRPTKKNALTREMYEEIIVALNEAGKDDTAVTVMTGAGDYYCSGNDLGNFMVIKPEEMHQVAKESGDLLRRYVNAYIDFPKPLVAAINGPAIGVSVTALGLFDLVLASENATFSTPFSRLGQSPEGCSSYTFPKIMGHAKACDMMLFNNKLTATEAKECGLVTKIFPKESFETDVMSQVEAIAKLPVKSLIYSKALMRDPELDLLHKVNEAECDRLVERWPSEDCINAIMKFFQEKNK

>XP_002130733_GCP60_Ciona_intestinalis

MAEVESVTEKLNQVAVVTNGKDDFWGFPLFEAYKMAIKFYKDNEGKRTFEIEYDNKVLLMALTKQVSHGPMNQQSSLPDIGYLDMFGHDRRKVWESLGDISSNDARRAFIEKLESCVPVFGPFMVAHQKEKEEQELKRKQKEDEERKKLEEEEKVKQEEQRKEKELAEKAAEENKLKQDQILQKQQIMQALNAQTHAQFQQYAAQQYPGNPEQQAVLISHLQEQHYQQYMKLYQQQQQQQQQQTQTDSTKGQQEQRQEELESTESEAAGSEGSATPVEDASMWTRPQITEFKEQIKRDPESVLTVGRGEVVTVRVPTHEEGAYVFWEFATDSYDLGFGVYFEWTDSEDQQVSVQIGESSDEETDEELSDPEKGSNSPHRPPTDEVVPIYRRDAHLEVHAGSHRYPGRGVYLLKFDNSYSLWRSKTLYYRVYYTR

>XP_015773089_ACBD4-5_Acropora_digitifera

MLTRVLRGCVEFYALYKQANEGACHESKPGFWDIVRKAKWEAWNKLGQMSKQEAMESYVSELKDVMESLPSEDGAEELFTDVLKSFYKEVYQGQDEPLPTVLKAFKDETPQNCKIEEFNLNGHNTQVKSELDKVPFQNGLVELPDQNGSEKDFPLSTDCGFPHRDSLHLAKIPQKYKKNGMGPFSKGVNSFLIISPDDLIKGSSKEKVEGEEEDNDEHQDRTSEVPPLKPHNTSMPNAQNSVVLTSDDSEDDEFCDTVDPEHLQEPQNGDQLKENELYLDDTLQVEDHLPSSTSSSSPEHATNSTLTESENSDLENDMKSSELLTSTPFAKHVTFAVESNVSADSSSVVNSELSKVIPSDTEESFVEVTPLLEGECISEAVAVLDASENNLVTPIENGDQKRTGILKSHHPVRECGGGEASQQPPGRGSVSRTRHTQGLRNKDSSGASGVCRLPRGTKGGGDSDSEDEFTSDHTSGPDYDSAGDELNDRVLQALERLHQDMKNVLRRLSSIEETISQSQLEVSRQSNQPWWKSFVPSKPLVFLILWPFIVNLVFYHLKQRKSSQNR

>XP_015747223_ACBD6-L_Acropora_digitifera

MYAMYEDTNCTSKPVTPNMAEYSQEVLVHGEDLQGLFQAATEFVRTLKNISDEQKLMFYGLYKQAKEGACTTSRPGFWDPVGRAKWDSWKKLGHMPQQKALECYIQELCEIDPEWEEKYAVTDDLTLKDPGKPAQKQTMGLAVSTLRGQENHEDEIISDANKSVFDWCKEGNVKKLDFLLTREGNVNTKDDQGMTLLHWACDRGHVDIVRYLIKNKADINAQDADGQTPLHYAVTCDFLSIVKELLQCGADCSITDSDGCQPIDVAESSKVKELLNS

>XP_015747922_ECI2_Acropora_digitifera

MAFCLRSLLHAKKLSFRPTPFTFSILRQIQIGNIRQVEGDKDFQAAVAKVKTLKQDPGNDNKLKLYALFKQASAGKCNEPKPGAFDFVGKAKWTAWNDLGDLSKNDAQQKYIIYVNDLVDKFGSTDEESNVVSNSEEKTTNMGSKYKELEVTLENGVQKIRMNRPAKYNAITWEMYHEFMTALEEGGKNDACVVAMVTGTGDYYCSGNDLSNFTRIPPEGPQKMARDAREILKKFVAHFIDFPKPLIAAVNGPAVGISVTIMGLFDLVYASDQATFHTPFMELGQSPEGCSSFLFPRIMGPAKANEMLLAGRKLTAMEAHQCGLVTDVIPHNNFQGEIERRVQALGKLPPKSLRLSKELIRDSSRDLLHEANEKEADLLEERWLSEECMQAIMNFMARKSK

>XP_015749156_ACBP-L_Acropora_digitifera

MSEAFLKAAEEVKTFKTEPSDSDKLELYAFYKQATVGDCNTDRPGMMDFKGKAKWDAWNGKKGTSKDAAETTYIAKVEELKKTCS

>XP_015767538_GCP60-L_Acropora_digitifera

MADGQRSTETITENSHNLTITEDRNFAADISQDQEQEAKEPSYKYGYSLPEYYKLCLQYYHKEKQIIKLTYDDKVELTAYWKQISCGPYDPEKYPDVGYFDVIGNDRRRAWEGLGNMQAKEAMKKFCSLLGTCSPELRPWMEAQQKEKEERERLQREEEERLRREAEEAAERERQRLLAEEMQRQAEEEERRKQEEIRKQQEQVLQQQQEQQQQQTLQQHQQQNVGQQEKTMAVNGTSRIAPASLWTRPKVQEFIQHVKSDPSSVLVVGRGETLTVRVPTHEGGNCLFWEFATEYYDLGFGVSFEWSQPYSKNVIVAVNESDDEEFAEEDKSSETESNPTKPRTDEILPVVRRPCNEEVIVGSHMYPGRGVYLLKFDNSYSLFRSKTLYYRVYYTR

>XP_006819673_ACBD4-5_Saccoglossus_kowalevskii

MATVNYESQFHAAVSVIKSLPKNGPFQPSYEMMKKFYGLYKQATEGQCTKPKPAFWDIIGRQKWDAWSNLGDMSKQEAMSCYVNELKKTLKKYNDPDKINKMLEKFPESGKMMEMMHVYQSDPRPEKIKEIVEAMPHTPEVALFVKNLGPFFEAITDNGPMELNANEKPLLPNGNATIGNSESSENETHPFDVSLSMLETTGQENGTHKFDLEGTEEESLTEEEEDEDDDVEEEDEEEVRSEEEELENGEMKEDSMNDGRDDGRDGGNSGIDENGGEEVALNEDLHIVDNKAYVMEVKPIDKSAVSVKKEKKDSESDEDGFVRVNGEYMNGGSSPKLTVAEPTFPHALTSDTDSGDEIFCDSVEKQYIEDEEIPKLIPSPDMHDVVTSTPNKSSFTVQAEVYDKPSVRFRKPEISHIMDGRDSVVRHGGGGGGGGDERGGSHGNNESHSRESQGGMRGGHRGSPHRTPLSGKPSRGTGATGGSGGRGNRQNSPPKGDVNEQIAVALERLQQDMNSVLIRLNTLETLSLRQQTETQQGGMWAGARYPTSQEQTPSSWWPLSGMSGKTVFFIIVWPFICNWVIKYLARRRQGRRL

>XP_002741676_ECI2_Saccoglossus_kowalevskii

MAFCRRIGVLAFDSVVMKTSRFSVIRPVSTSGKNFAAIDTEFSAAKERLTTLKEDPGNEIKLKLYALFKQATVGKCNAKKPSTFDFVGKAKWTAWNSLGVLSQEDAQKQYISTVNDLVAVEESASEEVASGTAGDYKQIKVTVEDGVCTILLNRPAKKNAINLEMYNEIGVALNEIGKDPKVVLAVVTGAGDYYCSGNDLENFMNIDPSKIHEFAKEGADVLRQFVASFINFPKPLIGAINGPVVGVAVSTLSLFDVVYATDRATFHTPFTALGQSPEGCSSVLFPRIMGQGKVCPGF

>XP_002730708_ACBD6-L_Saccoglossus_kowalevskii

MEEGELGYRELADRFELATDHVRTLAHRLSNDKLLYFYARFKQAKEGVCNTSRPGFFDFEGKQKWDCWKKLGTMSEMQAMKEYINELLRVDPSWHADAETTKTTTGMGVAVSTLYKEEVQIRDDQKTIFDWLKEDNISKVQSLMQNCNEDVTNQVDDQGLTLLHWACDRGLEKMVNLLLKYNANINAKDEDGQTPLHYAVTCEFVPVIKLLLDHGADVSIEDNDGCLPSANTYNKDIISLLGCP

>XP_002741767_GCP60-like_Saccoglossus_kowalevskii

MATDLGRLEATVDGLTINVEEDPPSTASSTTLTDMPDFEDKWGFTVKEMYRLSLQFYREMEGKVVHLSYKDKLKLVAYCKQVSHGKCDPNSSPPVGVLDVIGNDRRKEWLSLGDMSKETAMQSFCILLDKRCPQLNPWIEAHKREKDEQERKRRIETERRRHEEEEQKRSMLEDQSRKQAEEERLRQEQQRIQIKQMLDQQTYPQFAQYALQQYPNNQEQQQVLIKQLQENYFQQYMQQVYQQQLQLQQKITTGMNGELESPQQVQTEDHQKLQSSPATVEKTCQSVAMGAGDMPNAVMNGQHHHHDDEDVMQSANPQVNSVEQTELHVHDARCSHDRAEGHDDVQGTDGSSCEESYPPIANASMWTRPQIKEFKDQLRKDPESVIKVGRGETVTVRVPTHEDGTCLFWEFTTDHYDVGFGLYFEWSIAPSNAISVHVSESSDEEDDLDDEEGERGDLERGGRKDDRPPTDEIIPVYRRDCHEEVYAGSHVYPGRGVYLLKFDNSYSLFRSKTLYYRVYYTR

>XP_002736654_ACBP-L_Saccoglossus_kowalevskii

MPSDAFNKAAEDVKNLKTRPSDAEMLKLYALFKQTSVGDCNTDRPGMLDFKGKAKWDAWNEKKGTSQADAEAKYIELVEELKGKYGMA

>jgi|Lotgi1|234927|Lottia_gigantea

MADTTAKTKFDAAVKVIRGLPKNGTFQPSHELMLKFYGFYKQSTEGPCTQSKPGFWDVVNKAKWEAWNKLGDMPKEEAMLSYVDELKSIIEAMPQTKEVGHFLDKLGFFYEVVDEKDEVRSSSMKKLGNGDIQHDGQDSEGEEQEIETESNVSWNQDAELDPDFNHSTAVEEIMRKSEFINGNQKHITVSEYQTTHSKVSFDNQRPNINCKREEIHVNGGMNGVEESGSESDEFCDTSDEPIQDPVVNGDISMQLNTSTPISKTGHHVRFSDVVDTPVLHSNQTSRNSQHRDLRLGLLSNSVPFAAKTKKEAMLSDSLLVNATSNDSLNLSTDSQLGVSLHHPDNSILNQSECESVMSRGGGNVQKGQGSFPVSSDSFNDSVGSRGNSGNVSGSRRGLFNTNSGGSGEDGDDVLRGRNYSDLNEQIAVALVRLQQDMNGVLSRLTNLEAMTERQNQERERKELQKKSQWWPFGNLSKRTAAFIFVWPFVAHFIIALVCKRKRYHWF

>jgi|Lotgi1|178218|Lottia_gigantea

MPAHDAEFNKAKERLNTLSEAPGNDIKLKIYGLFKQATEGKCNKPKPGMMDLVGKAKWQAWNGLGDISQDDAQKKYIDLVNELIAADSPEPTSTTDSKFKTILVNKENKIYKITLNRPKKMNALNHLMYEEIMQALDEAGKDNSVLTVITGTGNYYCSGNDLENFTSTGGADIPAMAKQAREILYRFVGSFIRFPKPLIALINGPAVGISVTTLGLFDVVYCTDRATFHTPFSSLGQSPEGCSSYIFPQLMGSAKASEVLLFNKKLTAQEALDRNLVTEIFPDASFEKETMARVTKYAQLPPQSLQKSKNLMRQMQKDKLDKVNSEECDLLVERWQSDECMNAIMNFFKRKESKL

>jgi|Lotgi1|84981|Lottia_gigantea

LTESFELATEFVRVVAAKLKSEDLLYLYARFKQANEGRCKTPKPSFFDFQGKQKWEAWKKLEDMPTLTAKKDYIEYLSKIIPGWQHQEARDVNGGGDTAGLGIAVSRMVCEDDDIDDCDKTVFDWCKDGNVDKVKTCLNENTLNVDKLDEE

>jgi|Lotgi1|205113|Lottia_gigantea

MSAEFTTAAEEVKNLKEKPADAEMLEIYALYKQATVGDVNTTRPGMLDFTGKAKWDAWEAKKGTSKEDAEKAYIAKVAELKGKYGMK

>jgi|Lotgi1|115642|Lottia_gigantea

MADTQTLVNGVESLSVTNETNNEQNENQNADEYTAKWGFPLEELYKIALKFYKEKEGKALQLTYKDKLKLVAYTKQVVFGKFRNDASPEVGFLDVVGNDRKQAWQSLGDMSRECAMEEFIKFLNSSCNLFQPYVEAKKAEKEEQIRQQKEEEERIKREEVERKQRELEEEVLKQQELDKQRQVEQQMQIRQALNQQTQVQFRQYAEQQYPGNVQMQDELIRQLQEQHFQQYMQQVYQQQLLQQQQQQQALQSTTIQTTATENPTPNHGVTHQNNINTHLPRGRTPEDGEGLYHSLVLSIENDLPPLAAASMWTRKEIKELKESLKNDKDSIIKVGSGETVTVRVPTHEDGSCLFWEFATDHYDIGFGLYFEWTESANNNVSIHVSESSDEEDLDEEGVPVEGHGKKRDDRPPTDEIIPVYRRDCHEEVYCGSHTYPGRGVYLLKFDNSYSLWRSKTLYYRVYYSK

>XP_011405312_ACBD4-5_Amphimedon_queenslandica

MAATGKGPKERPLIERFETAVTVIRSLPKDGPFQPSDMVKLKFYGLYKVVKEGPNNTSKPAIWDPTGRAKWEAWKMCSSLDKEKAMTMYIQELEQIIETMPQTKQVEDFMKAIGPFYEFADDEGEVKEEESGGGDEGETMPSSAKKVTFNDQPSSNEDSDGDLEFEDSYDKLPSNITPPKPSQSLSERRPTPYVHSQATSLTDQKPDALSTNLFQSPNTSDLVNPQPTSSSADSQTLHSLLSQLIHNTQVLTQQFRCLLDQNVTQSSSQNKNKRIIFSVIGSFVINIIFKYWGARFTTDSSLYWLTMTACHIIFNLLTPCVC

>XP_003386304_ACBP-like_Amphimedon_queenslandica

MSNSEAFLKAAEDIKTLTTSPTNEEFLDLYKFYKQATVGDCNTDRPGMFDLKGKYKWDAWNSLKGTGKEEAEKKYVDLVTELLTKYAN

>XP_003385934_ACBD6-L_Amphimedon_queenslandica

MVEQIDVEDVDCLFQRAAERMRSNTNLQLSDEQRLELYGYYKQATEGPCTSRKPGFFDFTGKAKWEAWNKLGTLSSSKAKEQYVRLVDGIDPRWRDSDKDEEKSHSSGNRSNLAGPVVSRMAVKEGEEDKLTELQKKIIDWSTEGNVERVKECIANGESTETKDENGMTPLHWACDRGYIDIVECLIQNGADVQAKDNDEQTPLHYATSCGHRDVALFLIKSGANVTAIDNTGQSPVDVCEQNIKDIFN

>XP_003384577_ECI2-like_Amphimedon_queenslandica

MSLVFARRVLQGRAPLLLSSSSSAVPSIHMLWRRSFSLSNEDKEKLEAAKGKVSQLTTDPGNDKKLELYALYKQAVEGPCDTPKPGVMDFVGRAKWNAWNSLGQLSQSEAAVKYSELVDSLVGSSTSNETASPSNPVKLSSDDLLVTEEGGVQTITLNRPSKKNAITVKMYEDITSLLNSSASNPAIKATVVTGNGDYYCSGNDLSNFMNIPPEGPEKLAADSAILLRNFVSSLIRYPKPLVACVNGPAVGISVTTLLLFDLVYAADNATFHTPFMQLGQSPEACSSFLYPRIMGPAKSNELLILGRKISAEEAFERNMITRVFPKDELQERVKEIVRELSELPHQSVVKSKALIRSSFTDLLEDANAKECELLRERWLSEECMQAIMKFLEKRK

>XP_002110753_Trichoplax_adhaerens

MEEEFKSAVDKINNLASKPSNEDLLEIYGLYKQATVGDCNTDRPGFFDQKNRAKWDSWNSKKGMSTEEAKQAYIKKANSL

>XP_002113528_Trichoplax_adhaerens

MDSNTSLNASIDINSSIGGDLTNSSLHIDVTDIQAQGKWGIPTTDAYQLALQFYHKEKASLKMSYNDKLRLIAFWKQVECGPFDHKTAPEIGYLDVIGNDRRQAWIALGSTSQEEAMIGFSDLLSGVAPKFEKWMTGIVENARQQQMKRAESIRIRLQVEQEEERRRLEEEALRQKMKQIELEKAQAQTTRGNEGNVHQDPIGESVQNLNGNAQTTTQTPSPELSHVNVRTDPSIKSEQSNTTPEIQTAAASLPQQMQGVENKITNPTPSQQQNVTVPISEKTVTELNKGDDVDNAVEPKHIMLKDIPQPSLWTRPAVHEFINQLKDDAESKLVVSRGETITIRVPTNPEGAKLYWEFATDYYDIGFGVSFEFSDPGYMPVFETDTGANMEYGDDDEYDSEDEEGYRGSGDEDDIELAGISYFAQFIRDNWESHQTFSNYNTAFEYFHRVLMNEPYQIFCEYFYYPPYSKY

>XP_635850_Dictyostelium_discoideum

MTDAQIENKFKLAVDYITNNSGKLSNIKNEEQLYLYCNYKQATIGDCNTKAPPFYDYIGKSKWNSWNSLKGVEKIVAMNSYISLVNALSPGWDSNIKVERATQSVFLNDEEFKELEKKEKEEKEELKKLEEENGGEIEKPKSKWMGPVLSKFSLVDDETLEKLEKNTKQDLGYWVSVNDIERVKKEIENDKNIINEVDEDGRTALIWACDRGYFEIAKLLIENGSNVNVQDGEGMTPLHYAVVCDQFEICKLLLSQSSIDKSIKDNSDSIPSDFIDSSTSENIKSLFK

>XP_646321_Dictyostelium_discoideum

MTTFEEAAQKVKEFTKKPSNDELLSLYGLYKQGTDGDCNISEPWAVQVEAKAKYNAWNALKGTSKEDAKAKYVALYEQLATKYA

>XP_001749243_Monosiga_brevicollis_ACBD6

MAAFVDEAAFTAAAQRIRDARHLALDNDAKLALYGLYKQGSEGPCNMPKPGIFDFAGRAKWAAWQELGSLDQAVAQAQYVALVNQHLGLAHVADAAAGESSSGSESESESAASDSDGCDVEDETVQKSNNEDDKGATSLGAVQSTLLPPESGRLPADMSLHELAADGQLPELQARWAASDLDIDVRDEDGMTMLHWATDHGQTAVGIWLIEHGANVNLPDAEGTTALHNACYAQRQELVSRLLAAGADRTAVSGDGELAEDIWPAGFAKS

>XP_001744739_Monosiga_brevicollis

MTEAQFNKAVWLIRNGPAVGDSSNETKLSFYKYYKQATVGDNNESQPWAVQLEASAKWKAWNSVRGMSKEDAMKAYVDLLAKDDPNWEQHPALKDYKA

>XP_001744081_Monosiga_brevicollis_ACBD3

MATSASSSATSAAVAAAVKQAEVATAAANRGMVKKSTEDVVQPQQQQQQQQRQGQGPDAATTLAEAKQAQPTAAQLRQAASTKLLFELSLIFYKQHREQLNYDAKVKLAALFKQATHGPFDPTKSEAPGFFDFVGHERRAAWQALGQLSQEEAEQAFTAMLEETRSDFADWLDKRLMQKFNQEQEERQRRHRELAEAERRRQEALRQEALRQQQMEAERRSQQRAEAQAAKRAQQQESILAVSKTPRATLTSPAYIEEFKKSLQDKPDCEADVSSGEVLTVRVPKPDHGRMQVTWQFHTLDYDIQFGVDFEHKAEDGTLETETVAAVSRLNSHLEVVTGSHVADREGTWLLKFDNSYSYFRGKKVLYRVLCATI

>XP_001011628_Tetrahymena_thermophila

MISKRLNNQDNASKKFRYALLILNLAIPFKKRGKKRYQLINIINQFVKLQTIKFQKETKVVKNQQKNKQMTSLEERYNKALEFIKNPPADYPPIDMDNTQRLTFYAIFRQIKDGPCKGPQPSRLKVVERAKYDAWKALGKMSKEDAMKKYITEITKLAPGWEKPTPKL

>XP_001024105_Tetrahymena_thermophila

MSQQAKDILLQTLKYSNIILTGLILAYPKKFINLVYTKCKRPTKVQLTAATQQQDQLSQMQGTVKEDVLLKFKAACKIIRKEQNTDPKDSLQLYGLYKQALVGDCNISQPSSLNYEAKLKYEHWTKFKGLPREDAMMLYVEYVIQMLPKYEEQIAKISQGANENDQEESEDDNNISEKDLFETESDQDANEGEESSFKNKKEQVGRGFGLSVSKMTQNIGEQFLGQKEAQEVTEETQIFFSLLDEQNIPEIEKSIKEGFDLCQKNEQGSTALHYCIENELLDTFHTLIESFPNINAQDSDGFTPLHVAALNEQNHLVLELLKKGADPNIQDNEGQTVFDIANNSTKKIINDFIQQQKQNLE

>XP_001012898_Tetrahymena_thermophila

MPECSLKTAGKFQNQQYLSKQIIKVKINKQSKKTKNQEMAENFELAAEEIANFFKNKGDTSDDNKLELYALYKQGTTGDNTTAKPGMLDFKGKAKWEAWNKKKGVSQNDAKAQYVVLANKVLATVGKKV

>NP_011551_ACB1_Saccharomyces cerevisiae

MVSQLFEEKAKAVNELPTKPSTDELLELYALYKQATVGDNDKEKPGIFNMKDRYKWEAWENLKGKSQEDAEKEYIALVDQLIAKYSS

>XP_001383919_ACB1_Scheffersomyces_stipitis

MPSQEFTDKANAVQNLSKRPNDDELLKLYGLYKQATVGDNTTDKPGIFDLKGKYKWQAWKDLEGTSQEEAEQSYIELATSLIEKYDS

>XP_001386809_Scheffersomyces_stipitis

MSDSVDRVFVKAITTIRALSTRSNYGSLPRPPAENRIKLYGLYKQATEGDVDGIMPRPVGFTPEDEGAKKKWDAWKREQCLSKTEAKRRYISYLIETMKVYASGTLEARELLSELEYLWDQIKDIHYESEDEHDHNIPLPSRSPSFSQAAQSDRYSNVTPSIVPNQQYRNNLQKIYSHSRRSTMFSINDYVQQQRLQQQAQPQDHDTNVNTGIAGVGTASLYSLPMVDPRVKPQSVHSLPGNTSVNNIPAGTIQEFKNWQGEVNQVINKLTREFVNSRREYSRYNHKENEPIEEEEQLDEVQLLKRRVYHILKIVGKHCVTFLKNFSISLITILFIVWCVKKNVLVDRTVVKQPLGGTSSKQKKELIIKMVINTDENKWFIRLLSFINSFVGFV

>EEQ46883_ACB1_Candida_albicans

MVSAEFEEKANQVSNLSKRPSDDELLKLYGLYKQATVGDNNTDKPGTFDFKGKYKWQAWKDLEGKSQEDAEKEYIDLATELISKYN

>XP_721711_Candida_albicans

MPRPVGFTAEDEGAKKKWDAWKREQGLSKTEAKKRYVSYLIETMRVYASGTSEARELLNELEYLWEQIKDLPSSDEETDHHHIPLPSRSPTFSQTDRFSNRTPSITGARTTGTSNLNNIYSHSRRNTTLSLNEYVQQQRMQHQNQQQLHDTTSQPGAPVGGGGGGGGSIYSLPGRMGANNVIEDFKNWQSEVNMVINKLTREFVNSRREVQGNENGDPSTGDRDEELDDVEIIKRRIIHILKFVGWNALKFLKNFAVSLITFMFIVWCIKKNVHVERTYVKQPTNNANKSKKELIINMVLNTDENKWFIRLLGFINRFIGFV

>XP_002143080_ACB1_Yarrowia_lipolytica

MPSAEFTAAADSVQKLPKTPSDDELLELYGLYKQATVGDNNTDRPGAFNFKAKYKWDAWDKLKGKSQEEAEQEYIALVQTLSDKYN

>XP_505020_Yarrowia_lipolytica

MSDSIDRVFVRAINTIRTLSSRPGYSSRPRPPIESRIQLYGMYKQATEGDVMGVMARPVGDSEIDVAGQKKWDAWKQQQGLSKTEAKRRYVSFLISLMNTYASDTHEGRELLAELEYMWDQIKDVASDSEGAEEELYEDAVEYPSRSALGRAEVGTSPSHVSHGSGGGAHGSGVGGSSRAQQGSNLTHGSGNSRSHHTAIAPHPQSNLVQSQSQHQHQQHTPPIPSDRFKSDMAWALRTINEEMADMAKQYRPADPSEPRRNWRRRVLLLLRAIGVHVGIDMVLFAILAAILKARRGGKGPAVSRGVVSPVHTFPTRVLSLASLGDHISYHIIQMADGVYGYAVAWVSMS

>NP_596820_ACB1_Schizosaccharomyces_pombe

MSSTFEQAAADVKELKETPNSDELLKLYALFKQATVGDNNTEKPGLLDLKGKFKWNAWEELKGKSKEDAASEYISFVDELKTKYGMK

>jgi|Spore1|4105|Sporesorium_reilianum

MSSADVIDALFVKTVDMVQSLPKSGPIQTSYEEKLALYSLYKQATEGDVSSKRPGMLDMLGRAKCDAWSKVSGLPPRDAKQMYVESMLRILRRFSDRPQAASLIQELENFSGQVAQCVMDGSLAQVDSDEESITDTESDDQQRLDPTALHQQPSRAAQSNLPRHLAPNAPSVAGTSTAATRGGGVGDAASAISPGRTPIASESEQDSSNDDRLGSQPGPFRSAPRHSSSHAPPSVQSYRSQPRAGSISATEGYNRGGPPSRSQVSTSAQYDNPQALRSRGPPSLQGGYSRPQYAPQSTAASVAGSNYPRQPEYQRSTYAASSVGGRSIGTRTGPPSIYPPRPPSNAGTAQHQPRAEMESALQSIQASLAALHERLNRVESRSPAGGADDPRRSRPGSGQSSAAYFYNAIMNVVDDFKQLLGLNGVARGQRSEALAPSFLAQGGGGGSGDRSNAAINLLTSPVRLLVALVNLSARLALDVISLVLVSSFFLYAFKRITGRGDPLLMIRLLKRLQQAVLRRSPAAKQLVAEASV

>jgi|Spore1|5942|Sporesorium_reilianum

MSVEAKFNKAVSIVGSLPKDGPVQPSQDDQLKFYGLYKQATIGDVNTKKPGMFDLAGKYKWEAWNKNQGLSKEEAQQAYVDALLAILKKHEDEGDSAEHIKAIESA

>jgi|Elmca1|748264|Aporpium_caryae

MFGTMMVSSTGLSILAPAHGAHSARDAHALYAELLGNAPRPTTTSSGPRPQRKGSKTSLLSKLASFSNSRALFWASVRRCCYDARAGVFHAWPLRLGSYSHTPTNYWTQRCAPSVISRIPTTTTDYYKEDSSLRQAWSCIYGSVAKTRQNPDTLSVLSIAHSESDTMDVSRLIDIQFRRAVEIVQGLPKTGPIQTGYEEKLAMYRWGSGLISDTQTSGIATVGNVKTPRPGIWDMLGRAKWDAWAKHKDLPQHEAKWLYVLRKYSSQTVAMDLVRELEAFQGDPSNLVLSSSYLSSRRPSSESGDSSEDDQPRGMPLPFAPQPVPAHMLNNQRQPAFMQMQHQGGDATTDEDTDGEHPTQPPLVRPMSSVSSAHRYRTPMAMSTAPTTAFSPPPLPPLPDSQPQPHFSASSAFSPTIDSMPTGSLPSSSYPTSLQYAPRSSGAPTPTSYPSVSLARGSPLYGPVQMPMQMPMGMIAVRAEMHLALEEAVQRLQASTAALHERLAVLEDRAGGHARSNSRGGDAAAAAWDAQRYGAWALLLDPLARARAALDERAASPALLIFRRLLLDASFVIFVLWIARQTWRRSGTRRRQFLGVLRALVHLFTGAPRPARILVDRGV

>jgi|Elmca1|251068|Aporpium_caryae

MSEAKFNKAVAIVQSLPKDGPVKPSQDEQLQFYAQYKQATVGDVNTSRPGLLDFVGKAKWDAWKAVEGTSKEAAQAKYVELLLAVLKAQDSEEAKKYLKELEETN

>jgi|Elmca1|732012|Aporpium_caryae

MSQATFDSAAKYLSSSPALSNAPNAVKLELYALFKVVTVARRPETKRPSIFDLTGRAKWDAWDAVGREEQNTDLATLRDRYIAKALELGWNGDAQPTTAATKEEEPIDWDNDEVPSSSGRGNNNVSTMTTDGLLPDLDTLHGCVHANDTDRLLILLQRDMNMVNALDEFGFTALHLAADRGNMDMVKLLLQHGADPTIKVSVIVLFRPLHLSHVRRIQMTKPHCHLLRSLIMPLL

>jgi|Clapy1|869520|Clavicorona_pyxidata

MASYTSSSAFDEAASYLSNASSLSQVSSSVKLELYGLFKSLTVSPTPNTSRPSFFDMTARAKWDAWALAGKDYGERLADAEKKYLEIARTLGWKEGAIPGVADETDETVDGHSGGGVGLGNSVSVMSAPDEESASSSHLHDLAVRDDAQLLSDFLNGKSDPEIDVNAKDEYGYTALHLASDRGNRAAVEVLLKAGADKSQQDVDGYTAVELAKIAGHSDIVALLEGS

>jgi|Clapy1|1844844|Clavicorona_pyxidata

MDTHTLIDAQFDRAVEIIQSLPKTGPIQTGYEEKLTMYSLYKQATVGNVKTPRPGIWDMLGRAKWDAWAKHKDLDSYEAKWLYVDALLKVLRKYSDKTVARDLVRELESYEGDPSNIIMSRTLSNRSPDSDSSGSESAAGVPGSGYHSGQLPAHLKNQLALQRSQQTVPEEGTSEDETSEEELHEVRVPRAASTHERSQTVVNRPQSSLSSHRYRTPMASSMLMSPPTIPTSQPLPGFETPSAFAGPSSASLPSSSYQATTPYSGQFARQLASPPTYPPQHGYQPALGTRQHALPGGLPPRPPSRVALERAIESVQAHLAALSERFEVLESRSIPAWTGYASPGPAKSGPSPTFAGRGSPSGRGRDILDWNFDQMGLWSIVLKVLSRVLGSMRQLATFLAHNENHSPTLIILRRLFLDVSFILCVLALMKLVWRKTGIRRREVSAAFRALWWALVGRSPPRVMNDRGV

>jgi|Clapy1|1795290|Clavicorona_pyxidata

MSASKFEKAVSIVQGLPKGGPIQPSQDEQLYFYSYYKQATVGDVNVPRPGMLDFVGKAKWDAWKGVEGTSKEEAYSKYVQKLIEVLTAADTAEAKGYIAEIEAA

>XP_001874747_Laccaria_bicolor

MSSHELIDAQFDRAVEIVQSLPKTGPIQTDYDEKLTMYSLYKQATVGNVRAPRPGIFDMLGRAKWDAWAKHKDLDPFEAKWLYVDALLKV

>XP_001880619_Laccaria_bicolor

MSEAKFDKAVAIIGALPKEGAIKPTQEDQLYFYKYYKQAKIGDNTTVRPSGFLDFAGKAKWDAWSEVKGTSKETAWKLYVDKLLEILKKVDDEESKKYIAELEAA

>XP_001873760_Laccaria_bicolor

MSSPSFNNAAAYLSSAPSLSKTSSTVKLELYGLFKYLTSSKSPTTSRPSIFDLTGRAKWDAWSAAGEKYEKGKDAESRYIEIARTLGWTEGAPEARPVVDTAAEDIWDDDVDEGASGSRSGGSGGMGLSVSTMPVPPKVDDRTIHGLAVSNDFAGLTALLAQHPETDLNELDEFGYSPLHLACDRGSREIVRLLLDKGADATIKDPDGLSPRELANVAGHNEIEAILASSN

>jgi|Lacbi2|698396|Laccaria_bicolor

MSSHELIDAQFDRAVEIVQSLPKTGPIQTDYDEKLTMYSLYKQATVGNVRAPRPGIFDMLGRAKWDAWAKHKDLDPFEAKWLYVDALLKVLRKYSGKTIAMDLVQELEAYGGDPANIVTSRTFTKSPASDASGSTVSDVAPAHYTRENALASSQNEITHASEADSISEEESDDEPGDLPPANSRELAFENRPQSSLSSHRYRTPMAGSLVMSPPPTHGIPPAQPHPGYSTPSTFADPTSVPSSLYPTTGSYAGHYSQSSRAELVSPPHVYQSHPAYNQTSQYGPGRPASRPTLERALESVQVQLAALTERLESVETRSVLPSRVSLSPRGIGSPAWATGRGSPHDNNPQWDIDDLGMWSIVINPVTRAISVLRDLTVFFARNENRSPTMIIVRRLFLDVSFLVCVIAVIGALWRKSGVRRREVKAALVILWRAIVGAKPERVMLNRGV

>XP_001836410_Coprinopsis_cinerea

MSEAKFNKAVEIVRSLPKDGPIQPSQDDQLFFYKYFKQGTIGDNTTTRPGMFDFTGKAKWDAWESVKGTSKEDSWKAYVEKLLEILNKVGDEDSKKYVAEIEAAA

>XP_001836994_Coprinopsis_cinerea

MATHELIDAQFDRAVQIVQSLPKTGPIQTDYEEKLQILYKQATVGNVQPPRPGIFDMLGRAKWDAWAKHKDLDSYEAKWLYVEALLKVLRRYSDRTIARDLVQELESYGGDPSNLVLSHTFSKSPGSETSGTTDSEVGDMHTGQPQIHWRLGQPHASQDEEEETGSEEDETDDEARDLPPSRTPIHYDHNRPQSSHSVHRYRTPAGSLLMGTPPAEHQRVPHAQPLPGFETTSAFATPPPPTHAPFPHPQPPSHGSYPETIREREPLHSPQTTYPYRPGQYGPSRPASRPTLERAIENVQAHLAALNERVENVETRLISRSHPSISPKGGNSPRWFGGAGSPAGGRGPHWDIEELGLWSSVLVPLSRGLERVREWAVFFARDENRSPAMIIVRRLCLDVSFLVVVVAIIGSLWRRSGVRRREIRAALGVLWRALIGSKPPRAMAEKGI

>XP_001828602_Coprinopsis_cinerea_ACBD6

MTSGTYTPSPSFNAAVAYLSSPSYRGSPSNATKLELYALFKYTTTQSAKPTSSRPGIFDFTGRAKWDAWAEIGSKYRSVEEAERRYVELARELGWDGVVPVEASSSSSSGPERSSERPKAEEGEEEEIQWDSSDDDESSQPPKAGGSGGGGRGGGQGLGVTVSAVARPEEESSGDTKSLHGVVLNGDLSLLQDLLNKDPTLNLDAPDEYGYTPLHLAADRGHVEIVKFLLSKGANPTLKDPDEFTPLELAQVSGNEEIVAALKEAT

>jgi|Croqu1|132120|Cronartium_quercum

MTADKKQVEFEKAVEIVQGLPKDGDEKPSQTEQLAFYALYKQATIGNVNIPRPGILDFTGKAKWDAWKEKEGLSKEDAQAEYVKLLREYLEKFSDKEQATKLLAQLDSA

>jgi|Croqu1|588603|Cronartium_quercum

MPSQPPLGSTSSPFLPRHHRAYMRPTPTGVEDQYHIDFINPLFQTASHIVQTRLPASGPIQPTYKEKLLLYSFFKQAVNGDVQQISPRPGVFDMLGRAKWDAWKKLEGLQPVDAKKLYVDTLLKMLKKHAETPEARTLIEGLEGFTPSVARSDLTSTPSGSSSTLVRPTQHEDEPETGEEDLVTSDSEKDSSFQDNDEAPSSLGPYVTRAQLPDPVNMSQADPVVAETYPSKANQTRDSRIIPHHQLRMSDRPASRAPTELSVGSRDRALPDDTRSPANNRRLSAIQLAALISRPRRFDELDSLRSRGRPISRPPSAVTNDVMSSALDEIAEQDRSEHVTVAADAYLFRGPEGETLYRKLIAYKDLSQNYKLDLIS

>jgi|Croqu1|651440|Cronartium_quercum

MSKPDSSPQLSHLTTAEKLRFYALFKLVTTSGAGPLRAPSFWDFEGKAKADIWKATKLELEKTSQNGSCIEIVELAALEYIEKASRLGWVDSSREDPLQPHEREEQQQQKLVFELSKFLAEEGWDALQITPDETERDSGDENDEEDSAESESTTSERDEFGLRLDPNRESRPTTCEKTSDLKEHPIINMAPVSRMLDVTALNDPSSGEPSLHDLVIDNDQSGVERYLSLVLKQGGNGHVERLVNQYNTDGYTPLHLASDRGNFEMVRILINSGSDPSLKDEHDCLTARELACEAGMIVVENYLTQLEIQA

>jgi|Pucgr2|11284|Puccinia_graminis

MSEIDKKFDRATELVKSLPSDGDDKPSQEEQLEFYALFKQATVGDVNSSRPGMMDFTGKYKWDAWKAKEGMKSEEAKTKYVELLKSKLEKSSNQEQAKKILEQLEAA

>jgi|Pucgr2|1489|Puccinia_graminis

MPTRTGGNPSGSRPLSSSSRLENFPKNGLGQPYTHKQQHSSSIGSSSTSLLAKSSVLPPSNPETSNESPPSNHSHDNSQSDHEEAFIEPLFQTALHIVQSSLPRSGQIQPTYNDKLLLYSLFKQAVHGDVKSTISKRPGVFDMLGRAKWDAWKAREGLSCSEAKRLYVESVLKILRSHRDRPEARLLIESLSGYEAYLATVDHMSDSDEDLYSERKIEIPASMRGSIHTLSGQEQAQEGAEEEERSEENESAGSKSDEKSADQLEEEEEDIQTSGLGPYKSSSQLPDPINSSTETHILPPHSPRAIEPSSAASSESLSQSPSPKDDSSPAQASQESIQSTHIEPSEPRFTYAQVQRLVDQTPQTLPFHSLRSPLNHPTHSHPDLSRLTLPQSSHDPYSSSLALLQSNLEKLNQTIDLLHTLISKIHPPQHHSSTSSPPPPPSALNKNILVATPSSSPSSNPHHKSYSLLSLSRLRTVLNHPWVKRLFLDLSLFFLLLKARKFFNKRPV

>jgi|PleosPC15_2|1090291|Pleurotus_ostreatus

MSAKFEKAVSIVQSLPKDGPIKPTQDDQLNFYKLYKQATVGDVNIPRPGMLDFTGKAKWDAWDSVKGLSKEDAMAQYVEKLIEILKKADTEEANKWIAEIEAA

>jgi|PleosPC15_2|1075883|Pleurotus_ostreatus

MDSHQLIDAQFDRAVAIVQALPKTGPIQTDYEEKLEMYRCQATVGNVKQPRPGIFDMLGRAKWDAWAKHKDLEPYEAKWLYVEALLKVLRKYSDKTVAQDFVRELESYGGDPSNIVSSRTFSHSHGSESSGSTVSDDVPPSRVPQGLPRRPQSSLSSHRYRTPMAGSLAMSPPPIHPAVPETQPLPTYETPSAFATRPASRLAIESALENVQAHLTALNERLETLESLSFNPSRSRISISPRNSGNISPYGRRTPDDNEDRLEWDVNDMGLWSLILNPLAHGVHQLKNFGRFFIRNEDRSPALIILRRLCLDVSFLLFVLAIVRAIWRKSGVRRRELQIAKGSEKSKVLPPASKSWDRVYMQGSRIRM

>jgi|Amamu1|72566|Amanita_muscaria

MTSDEGAKDSIDVQFDRAVEIVQGLPKTGPIQTDYEEKLVMYSLYKQATVGNVRSPRPGIWDMLGRAKWDAWAKHKDLDSPEAKRLYIEALLKVLRRYSDKTVAKSLVDELESYQGGPSKILRSRNNGRTSDSNSSGTSVSTGSPRSQYSRETLLNRQDADNENDLEEERGEPGDLPNTNTMPSVSHLGRPTSSMSSNRYRTPLAGSHITSPPPSQSIPNLQPRPSFETPSAFADPRYSRSPPGSTLFQSSYTGQYEEPIHSGLITPPITTYPPPQHRENPQLLPRLRGGQQVSRITLERAVEHLQTQVAALQERLETLEASRYLSKSLTLPASRGSSPNGGRGLQWDINDLGLWSVVLNPLINSIDTVRSMARFLARDENRSPLAAILRRLCLDFSFLLCAIAITRFVWKGSSARRREVRVALKILWRALLGTKPQNVLVGEGV

>jgi|Amamu1|183488|Amanita_muscaria

MSTYFPSPTFYEAATYLSGASSLSKVSSSVKLELYGLYKYLMASPVPTTTRPFVFDITGRAKWDAWDAAGKKYSNPDDAEHRYIEIAESLGWTEGMSTERRPSQEEEIDLDALDDEPEGKGKEKEKSSGVPEVGLGHAVSKVQMLLDSMQDLSIHGLALENDVAGLSALLKKNPNVDLNVCDEFGYTPLHLAADRGNTDIVKLLLEHGADATLKDSDGFSALELANVAGHQDVKTMLSNALST

>jgi|Amamu1|262412|Amanita_muscaria

MSEVTKAKFDKAVQIIESLKNVPNDGFKLETDDLLFFYRYYKQATIGDVNTKKPEGMFASLTANLVDFRETAKRNAWESVKGTPVEVCYKKYVEKLIELLKKAGDSKSLELVQELEAA

>jgi|Saico1|60794|Saitoella_complicata

MPSAEFTKAAEEVKNLSKKPTDDELLCLYGLFKQATIGDNNTDKPGMFDLKGKYKWEAWNKLKGTSQEDAEKQYIALVEELKAKNA

>jgi|Saico1|30257|Saitoella_complicata

MSSDSVDRCFAHALATVRRIPGGPTRPPFADRLELYGLYKQATEGDVHGLMPRPTGNSDEEASARAKWDAWAAQAGLSKNEAKRRYISKLIDNMQRYASPHPATTELISELEFVWSQIKDAPASSATTSRESSPARSPEHLRFGSSSLGQYFRHSRTHTLEGDDAGPWEEGSSRMVGRGAVRTSTSRWKEDVERALEGLRVEVTALSEELQASRAPPPPPPSSRTSPLTKISKGFLMFLSKHALFDGIVLILAWLWFQKGEAFRGLLRRVFSDWLWRKILKARSKTRRQHAAE

>jgi|Boled1|908066|Boletus_edulis

MDSRQLIDAQFNRAVEIVQSLPKTGPIQTGYEEKLTMYSLYKQATVGNVTSPRPGIFDMLGRAKWDAWAKHRDLDQYEAKWLYVDALLKALSKYSDKTVAKELVDELHSFGGHSENLVSSYTLTRAGSRSSSSSSSTTSEDNAPIPPSYGLHDFQHPSSHQHISTVALSESSDDGEAGDETRDLPAMREVPQQQRPPSSISFSSRYKTPLMGSFALSSPPPRRTSVPPMQPLPNFETPSAFADPNTVTSVATSSTYTSSVPYAAPSSSRDLAPRIQSALTSRHSTTSLAHLRPPSRAISVSLEHAMENMQAHLAALTERIDTLESSLETHTHSQAQNSTSLSSHSSGRGSPAGSRSVSQVSEWDVDDMGLWTLVLKPFLRHFSSLQRLLLFFLRSRQDRSPALIVVRRLCLDISFVMVVLGLLRTVWIKSDTRRREVGVALRILWGAVTGRRRILGGKHLNLRL

>jgi|Boled1|936533|Boletus_edulis

MKYTPSPSLSEAAAYLSSASSLAKVSNTTKLELYGLYKYLTVAPVPQNPRPSLFDLTGRAKWDAWSNTGKVYADNGSDAERRYLDLARGLGWVPGAAVSVPQNEGEVWDEDLGVSISLSSTQGKGGMGGSVSVMTASRDEGNTGGHILHAFAIQGNVIKISQFLAENSLVDVNGRDEYGYTALHLACDRGSLPVVQVLIKHGADVTIKDPDDLTGMELAHVAGHDEICAFLRTKLH

>jgi|Boled1|964172|Boletus_edulis

MTLTLQEKFDKAVKYVQSLPESGPLKPDQDTKLKYYAYYKQATIGDVNTGRPGIMDFVGKAKWDAWNAVKDTSKEDAQKAYVELLVMHLEKITDKSEEVKELIQELKE

>jgi|Morco1|559946|Morchella_conica

MSDSVDRVFAHALQTVRKIPKTGSARPPADDRLRLYGLYKQSMEGDVKDVMDRPTNYGPESRPEIEKWDAWNAQSGLSKTEAKRLYIATLIEVCARDLVAELEFVWDQIRSNQSSLSAASPPHDRTQYPPELGVVSPNPFGEEEDEDSGVDADHRKWRRRIEQALAKMATEVTALRERMEDDAAFRRRGRLRGWFWWALGVLGRHVVVEVVFWGLLWVWMQKRGDRETVQALTRVVGWGRERVGEVGRWRRWVR

>jgi|Morco1|607810|Morchella_conica

MTSAEFDAAVTAVNSFKKMPSQDDALLFYAYFKTAKFGKPTDGRPGLFDMKGRYKYDAWAKAADELSPEEAQKKYIELYESKKADYEN

>jgi|Ascni1|177825|Ascodesmis_nigricans

MTMADSVDRVFAHALQTVRKIPRTGSTRPPPDARLKLYGLYKQSMEGDVGGVMERPVLESLEHASEEDRAEVEKWDAWYSQRGLSRTEAKRRYITTLIETMHKYAYGTAEARELVSELEFVWDQIRSNISPTSTAPSSPALRAISPLPPATDNGLSSLPLPTISPHPYTSADDDALPPTDALPDHSPAWRLRVEKALAKMTTEVAALREQLESRRMREARRKSDFAMWVLYLGWAVVRHLIVEAVFWAIVVLWMRKRGDRRAEEAVRVVGRFVREGLRELGFGRRRGVPSAPSTVGSTRG

>jgi|Gyresc1|520122|Gyromitra_esculenta

MSELPKVPKSPEFERATAAVRSFVQMPNEKEALKFYGLYKFGCHGKNSEGPPLFPTFYAGKKWAAWNTIAESGITPDDAQKQYVAFYEANKEKYEAGHV*

>jgi|Gyresc1|536723|Gyromitra_esculenta

MTTLPEHPQSAEFQEAAAGLHGLAKRPSNHELLEFYGLYKVGRHGKNITPQPWTKLSMEGQKWESWTKYSKKGITPEEAQAEYVEKYNAIKEAQNNASK

>jgi|Gyresc1|573429|Gyromitra_esculenta

MSDSVDRVFAHALQTVRKIPKTGSARPPAEDRLRLYGLYKQSMEGDVEGVMDRPEVFGEESRAEREKWDAWNAQSGLQKTEAKRLYIATLIETMHKYASGTQEARELVAELEFVWDQIRSNQSSLSAASSPHASGTLANRSNLAPASSYPGELGLVSPNPFGEEEFEEDISGMDTDHRGSRKWRRRVEQALAKMATEVTALREQMEEERASRRRGKLRQAGQWFLWAMLATGRHVVFETVFWGCVFLWMRTRGDTKAQEALQLVVAFVKERLADFGFGRGKKITKLIR

>jgi|Pyrco1|12411|Pyronema_confluens

MLFTPEPPLSPRLNTTTPVTSASLIHPTSTNNALTESRVIQQSLGSHSMRGMRRQSTMSDSVDRVFAHALQTVRKIPRTGSSRPPPEARLKLYGLYKQSMEGDVTHVMPRPPDPVPPDVDIAPDENLRAEIEKWDAWYAQRGIPKTEAKRRYISTLIETMHKYASFTPEARELVSELEFVWDQIKTNTATSSDDPPSPPTQAPLTTSNLTASNLFNNASTSFVVPGSAYQTPRLNANPDSDDEDDAGPQGLGFISPAPYAASAASSSPDPPGPGNPAGGNKIWRSRVEKALSKMTTEVAALREQLESRREERRKAKSRRVWVWFLWGCWAVARHLVLEAVIWGVVLFWMRRRGDRRAEEALRVVGRFLRNGLREMGFRGRRGIQR

>jgi|Kalpfe1|694171|Kalaharituber_pfeilii

MGAEFEKAAEDVKNLESKPSDDELLKLYGLFKQATTGDNTTDKPGLFDFIGGYKWKAWKALEGTTKEDAEKQYIAFVNELKVKHGFQMYEG

>jgi|Kalpfe1|848121|Kalaharituber_pfeilii

MSDSIDRVFAHALQTVRKIPRATASNNVPGSPSLNAGGSTGTASSSGPSIRRPPAEVRLRLYGLYKQSMEGDVLGVMERPTDKAEADKWDAWNAVRGMGKTEAKRRYIECLIETMKKYASDTPDAKELVSELEFVWDQVKSNSRHHSANGSNASNTYTPRSAEPTQPQKQQQTQVQLQMHYQTLSQRPPPSPTSAPVSKYRQEPSRPPDYLDEPDYRGYQSSGDHEHLISPGQPYYEHSHDAGEPRRYLPLKRMKVGSRGTRSIRSGDDGDQTGDEEAREADTAEEDEDEEEEDDSEDDELFEEPSMTRLHHNYDQDDDEEYDEDGFRTRLVLGRDTSPDLGTMRWQLRVERALTKMSGEIAALREQLEIRNAHVLQNSSSWFWPHSDSSRRQSMTTTGGKKDVAGVPKTFGKVIWQVIWNVLRHLMVDAVVVGIVVWWMGRKEDRRAGEIWKWLTKIIGSRRRRRGDGLQTGIEGS

>jgi|Artol1|1519|Arthrobotrys_oligospora

MPSPEFDAAAAAANAFTKKPSDDELLKLYGLFKQATVGDVNTDRPGMMDFKGKYKWDAWKSEEGKSQADAEAEYIAYVEGLKAGFA

>jgi|Artol1|10651|Arthrobotrys_oligospora

MADSVDRVFVHALNTVKKLPRSGSSRPPPEDRLKLYGLYKQSMEGDVDGVMPRPTGLSDEEVTEAKKWDSWHEQKGLSKTEAKRRYITTLIDAMHRFAVTTPDAKELVAELEFVWDQIKSNQTSSSSSTTAAPAQQQQALLSSAATTPRRQPASGLSHSQNISATGLQILPPMSPTEEGRRFTNFPPVGMGVQTDSEETGNRPDTGDDDEDEDEHYETMDEFDENGLPKVTVINEPAGFKEGGSTGNLIRSINTQTAVGGGGGSGVESDILRQKKQHIKHLSSSQFTTTKKSLAALSSTGSMPRTNPQQQQPTNGQTSRENAHYTRWKQRVEVALTKMTAEVAALREQLDVARNLNGGGMNLSRGRLNWIRRWIGWGVWTVLKHIVIDAILIGVLVVWMRKRGDKRVEGVLGLLGGFLSERLRLKKDVPDVSRTFVIA

>jgi|Monha1|10055|Monacrosporium_haptotylum

MVSAEFQAAADKVNNLTSKPTDEQLLKLYGLFKQANVGDVNTSRPGMMDFKGKAKWDAWKSEEGKSQEAAEAEYIAYAEEVVASVA

>jgi|Monha1|4485|Monacrosporium_haptotylum

MADSVDRVFVHALNTVKKLPRSGSSRPSPEDRLKLYGLYKQSMEGNVDGVMARPTGMSDEDITEARKWDAWHEQKGLSKTEAKRRYITTVIDAMNRYATTTPDARELVAELEFVWDQIKSNQTSSSSSITQQPLQSTISSSATTTPRRQTLGGLSHSQNVSAAGLQIIPPMSPTEEAAGRFMNFPPVGMGVQTDDEDAVAAAGADDDDDDDDGDEHYETMDEFDENGLPKVAVISEPVTFMEATGSGSLVKNANLPQEDTSKRKHAKNMSSSQYTATKKSATAAAFSSGTSSPHTHTQQQSTGNQHYTRWKQRVEVALTKMTAEVAALREQLDVARTLNAGNVAITRGRLGWLRRWISWGVWMLVKHLVIDALLFGLLVVWMRKRGDRRFENLLGLIGGFLGDRLRRKADVRDVRTFVVA

>jgi|Phybl2|124466|Phycomyces_blakesleeanus

MPSANFEAAAAAAQAFTSKPSNDELLALYGLFKQATVGDNETTRPGAFDFKGKAKWDAWTAKKGLSSEDAEAQYIALVEELKAKQ

>jgi|Phybl2|104584|Phycomyces_blakesleeanus

MSAIPSHVSERYINSRYNKALNIVQHLSASSSVQPTKEEKLELYALYKQVSHGNVNTPRPGMFDLVGKAKWDAWKNQESMAAIEAKYRYVDLLLRVASEVSVIKGFFD

>jgi|Phybl2|110945|Phycomyces_blakesleeanus

MIPPHYTDRYVDQRYNKALMIVQNLPASSSFQPTKEQKLELYALYKQVSHGSIDTQRPGIFDVVGRAKWDAWKKLEGLNDLEAKHRYVDTLLRSATEVY

>jgi|Phybl2|189028|Phycomyces_blakesleeanus

MESAQLDDWFESAYIYLSTHEKISISNDNKLKLYALFKQVIIGDCNGPKPNLFEFVARAKWDAWKSIYGMPANEAKEGYVSFVESLKVGWSRAGEYKYEGVDEETRGFGTSVSTLSYDTEEIVEEDLFSLTCAGDVEKVRERVTKYNDSVNSKDDEGLTPLHHACDRGFPDMAEALIELGANINAQANTLETPLHYAYISEQLETAKILHNHQCDISLRD

>jgi|Absrep1|452230|Absidia_repens

MDFITSLNSNAEIPQMELWFESAFDYLNTHTVTVSKDNKLKFYGYFKQATTGDCTIAKPNLFEFVARAKWDAWNNLKGMESPVAMEAYVELAESLDIGWTRQGEYQYEAKDSNKDTEETGHVLSSLAHADDDDEDNKHDLIGYTKANKLDQVREQVKEQKEPVNTKDEEGLTALHHACDRGYTDIVQLLLDLGADINATTNDSETPLHYACISEQAAAAQLLIDKGCNLTIKDADDQTAFDLVDSTFLSLLNYTS

>jgi|Absrep1|407177|Absidia_repens

MSSIPSHYSERYVNQRYNKALHIVQHLPASSSFQPNKDEKLQLYSLYKQVSHGDIDTQRPGIFDVVGRAKWDAWKKLKGLSSLEAKHRYVDTLLKICAEAYKKPAAKVQVQQIIQAFATIRPMDDDDSDDTEESDDSNDEEGHDDGETSESVDEEEQAYLMDIQQSVPAMTPSKSASTMGSQASDSYHSLRSPVLRSSTSTRTKQSNRLSWHGKQPRQLRRAPSIDSTQSMVTAPNAPTRRLSRSSSITTTNFNNAAGMRPPSTMSQRSDVNQQSQYYSRTRRTNLPHAGLAAQESTALGGHTSTNIREPLFDEDHLDNSVNPWATTQGTGGGSNVIPNRRYRMGDDSSDDSIDEEYKQNYLYAPPLAHNLTSAPPSSGTGPSTTTGGMMTGIASSSSVASHYYQQTNRPQQHPHQQQHQQQQQQMNQFDLPMQSPLLGGSTTSSVTATGAFSDSQRQNQHHRQQQPLHSRENRLSTDQQYTSVVALGPATKRALEILQAEILALNERIDGLRQELIDRDQKDLQHKKKKSILSGRTLSQRGDDDEIWDAWGWVFKAALKHTTVNLITIFVIVLLLYKRGNPIADVIIGSLYKQWHKFKLSLAKNNRIV

>jgi|Absrep1|411144|Absidia_repens

MPSPAFEQAAKDAKNFTKLPEDEELLQLYGLYKQATLGDNNTDKPLLDFKGRYKWDAWNDNNGIPQVEAEVQYIALVESLKTKYQ

>jgi|Absrep1|411342|Absidia_repens

MPSAEFNTAAEEVKTLAQTPSNDVLLELYALFKQATVGDNTTDRPGVFDMKGKAKWDAWTKIKGTSQEDAEKQYIALVQKLKN

>jgi|Absrep1|406421|Absidia_repens

MTSAIPPHYSNRYVQQRYNKALQIVQHLPSSSFQPTKEQKLQLYAFYKQVSIGNVNTARPGLFDMVGRAKWDAWKKLEGFNKLEAQHCYVDILLQAVSEAYQKPASKIQAQQIIQTFSMMKPIGDDDDTTDDEMTTTTEDGDHADNGSSVASVDEEEKAYLFDIQQNAGRSTPSTLFGQSELTTSTSPRKPLGQSNIDQRSWNGKQRQFLRPKSSASLDRIISSASPNLNRTTNINNDLNKPMTGTTSRAASSASRYRTHVLPRDSSTFNDHHFDDTMNPWASGTQQPLHHSTLSDYQQHPQLLRTDSRSTNHTNMSAAPMLPSPVMGSGRLRTLQSPFHGSSAASSVTATASNVRTLQQNQQLGRRQEIQPTPTPSVISQHSVALGPATKQALESLQTEVIALNDRIDDLRHELIWRDRRRAEQRLLDDKSEGNGEGDGWQWVIKAAIKHAAVNLLTALIILLILYKRGNPAAYAAVGQFSKLWYRLRGWLRVTTVVV

>jgi|Absrep1|410904|Absidia_repens

MSSSFLHTIHLRFNRALVVVRSIPEHEVGLQPIPMDRLKFYGLYKQATLGECNTPKPSSRKVVEYAKWKAWYRVRHLSSMEAQNLYVNALVELLVEFINRYPNNQYIEFAKEALHSLESDLPAESYVEETGHEAYADAWEPTALTGDGHDPLTHIEPHHLTNYTHLVTSPTISSFFTNDSMASNSPSLHASTTPSSVTHTNDPQTPLLSPQQHKQHPRHYDWQKWSTTNPTIMDVASPLSETARLKNNNDNNKPTTTGTTLMSSPSLSSSESCSQLLPQNDAHNNKFPERTLENLQTEVTALTEQMDRLRQELKDKEMMKQHFGIGWLLRTLIKHLLANSLIAGILFCILWRRRSPLAVALVDHLTPQIKMLLRSMIRSTVKKVMFWKLTV

>jgi|Absrep1|82996|Absidia_repens

MSASLPYLIHLRFNQALIIVRSIPEQEVGLQPIPSDRLKFYGFYKQATFGECNTPKPSSRNVVEYAKWKAWYRVRHHSCMEAKNLYVNALVELLVEFINRYPNNQHHEFAKQALQSLEFNAATASESKCIYMR

>jgi|Absrep1|413211|Absidia_repens

MTSQQRLSTNSSSSFQTQIQFSRALTVVRSLPSGNTHWQPSGQDKLLFYGLYKQALEGDCHISRPSSRLVVAYSKWKAWDQLRQWTPVEAQKHYVTLLLDMLSSFVERYPQHELTRSLRESIHYIQLDTDMDPSITSSLIDEKDSSSYASSHHHHHHHQPPAVHLSNTSPVTPDDNTLSFPSKQQQQQQHSWHESNRLLLQPLAADYYDGMSDLDTIDREVAAATSGLGLMAPEQHPYHHYHHHHQHQQQEQQNQSELVESVSERALESLQTDVAALTEQLGHLRTSLAQRRHDKLRWTWLWLGKTVVKHASINLVILFLVFLVLLKRKSPIAYAIIGYVGSRIQNILHYLAQKMYFWKVTV

>jgi|Mucci2|146146|Mucor_circinelloides

MSFIPTHYSNRYINQRYNKALYIVQHLPSSSNVQPTKDQKLELYAFYKQVSHGDIDTQRPGIFDVVGRAKWDAWKRLEGISEMEAKHRYVETLLQAAKEVQRWQRALVECPC

>jgi|Mucci2|145636|Mucor_circinelloides

MASIPPHYTDRFILQRYNKALHFVQHLPATSNFQPTKSQKLELYALYKQVSEGDINTQRPGLFDVVGRAKWDAWKKLEGISTLEARHIYVEALLRV

>jgi|Mucci2|113048|Mucor_circinelloides

MPSAEFNTAAEEVKNLSTKPSDNQLLELYGLFKQATVGDNTTSKPTFDIKGRYKWDAWTKLKGTSQEDAEKQYIALVESLKAAQ

>jgi|Mucci2|110863|Mucor_circinelloides

MPSPAFEQAAKEVHDLTSKPSNDDLLKLYALYKQATIGDNETTKPTFDIKGRYKWQAWEDLKGILPVEAETQYIALVQELKTKHQ

>jgi|Mucci2|19069|Mucor_circinelloides

TTQLKFAQALSIIRAIPQNSTLQPIASEKLQFYGLYKQATEGDVNIPRPSSRQVVEYAKWKAWSRMKGMSPIDAQKLYVESLVQLL

>jgi|Mucci2|108156|Mucor_circinelloides

MPFTNTTPSESLYVTQIRFCRALAVVRSLPTKTNAAFQPSLSDKMHLYGLYKQAIAGDCSLKRPNARETLLYAKWRAWDSMKGKNPIEAQVTYIHALVELLEEVFIRRETSTLGDLNESLNYLIEVEDDSYEAEIWDASDQYYTQQEQFPSSQSTPTTLDFPLTPQASEYIHKHHKDDYAHTLYEDLFDQQRSYNTPISMLQSEVTALCREMRSLQFISSKSIFQWHWLWFLKSVAKHAIFNCLSSILLFFVLWRRKSPIAYAVISYVGPRVRDVLQYLTSRIIA

>jgi|Blatri1|415280|Blakeslea_trispora

MEDDFEMCQEDKWFEQAFTYMNQHAIPLPNDKKLHFYGLFKQATVGDCNIDKPGLFDFVARAKYDAWQGFKGLNFREARNQYIDSVEDLKVGWSRQGEYEYIPSLQELKEAEGISNVAVSSMAIEKEEEESQDVFGYTRLCDLEKLSKVIKSDPEAINTKQDGLSPLHIAADRGYTDVLKLLIEAGADINSKTDDEDTALHLACIAEHLDAAQFLISKGCNTTLLDSEGKTAFEQADASFSEKLK

>jgi|Blatri1|382870|Blakeslea_trispora

MPSEKFNAAAEEVQKLSTKPSNDELLKLYALFKQATVGDNETSKPTFDIKGRYKWDAWTEVKGLSKEEAEEKYIALVEELKAKQ

>jgi|Blatri1|415009|Blakeslea_trispora

MANIPPHYTDQYISQRYNKALHFVQNLPASSSFQPTKSQKLELYALYKQVSTGDINTQRPGLFDVVGRAKWDAWKKLEGMSVLEARHTYVEALLRIATEAYKKNMGRAEAQQIIHAFSNMKPSDTQDVDDASHHENISVASEEAEEQAYLQDIQQNAPSTISDKQPQQLRRPGSVASIQTAVTAPIIPYHNQLPSTSVHQVRETPSQKQRIAERRPLQATVIREDARTTPLGKRVFQEQDYVDASVNPWQHIPPFSIRNRQTMGDDNNSYSSSNNSSDENNHSHTRRARTPQSTSRLARTRSMYTRIASPSRSNVTYHPQRFQSPVFGSSTSSSVTTTPHNFQDHVKSRLAYQNTLELDQASSHLALSHSTKRAIEDLQHQLVILNERVDELRQELVEQDRQRIALGRQAAKSSLSSSPSTDDTNGNSWSWVIKAALRYAGVNLMTLFILFLALYKSGTLIAHITGLRNFKAFVELNHLDLRRLGWPIRVFA

>jgi|Blatri1|403679|Blakeslea_trispora

MNTNSYTTQLCFAHALSIVRAIPPDSTLQPTAADKLRFYGLYKQATEGDLNMPRPSSKQIVDYAKWKSWHRMKGLSPIEAQRLYVDSLIQLLAELIHRYPHHPQSDFLKDALNELQFDEQNTQLIEEEDSFQDAYDPAEFELQEHFLNHFEADQLMNENVLLSPPASSTQFSFKSPHRYSFSSVNATEYPLTPIASPGHHSHTGQWVQGQTEKQPKTHRMPIEIDTVSETDVLDKEVAKVTSNLSLSSPYGSLPVSSNHEIKKGIRPSPSQKTSLRKKSRATSSSTTHTNPSEPLSSSSLSSLSSSSASSDKQISNRAIEKLQTELTALTEQMDQLRRNIKTKEERDRRLRRSPWYVFQLLVKHLAINSAISLIVFYVLWKRKSPLADKVIWLLRPWIQNVIRNILRKIVFWKVTA

>jgi|Spifus1|1957861|Spinellus_fusiger

MASTQLDDWFESAYIYMSTHEGIKVSNDNKLKLYALFKQVIVGDCTTPKPGLFEFVNRAKWDAWQSAKGIPGEDAKTQYIALVESLGVGWSCQGEYAYQQSKEEEIHGLGACVSSLAQNEEEVYEEDLFGFTRTNNLDKVREQIKEFKQSVNSKDEEGLTPLHHACDRGYTDMARMLIDLGADINVQNNALETPLHYVTCLLSAYISEQFETARLLHQQGCDTSLRDEEGKTAYEQGDHVFVTELFAIIN

>jgi|Spifus1|1857028|Spinellus_fusiger

MPSAAFESAAVEIKQLTTSPSNDNLLKLYGLFKQATVGDNTTSKPTFDIKGRYKWDAWTELKGLSSQEAEDKYIALVKELTSQ

>jgi|Spifus1|861251|Spinellus_fusiger

MSSIPPHYSDTHVHQRYSRALSIVQQLPASSSFQPTKEQKLELYALYKQVSNGNVSTHRPGIFDLVGRAKWDAWKSLEGMNRLEAKHCYVETLLKSAAEAYKKPVSKAQAHQIIQSLATMHSVEEEEEEEEGDTESDYDRMDQDEGISFVD

>jgi|Spifus1|1832773|Spinellus_fusiger

MDILFISIVIAIAIAIVIIGAVMSASPFTSSIEKDVQRRYTMAVDIVQRMSASTTTLQPTKQEILELYALDKQLSYGNVHTPRPGLFDITGKSRWDAWKALENISSIEAKCRYVDILLAMAVKACNKEKSRSEARSILETFTTARPLQKTQIQYGNKTQEDVHHEAALHPLQTAPHGTKHTYISPRPQRSSSLYAPVIYENFERKNPWSNASHSSSQRYNRRQSDGSSEDSIDGSHLQRKPSFLSNTRPSLHDPSVSLRVASHVSGASTTTTTTSVMPKDKKDPLTYQPMSMDPMSDSVLGPTTKRAIDLIQSEILLLGKRIDDLTKELSEYTGRHPPPTQLMASPTKEEKRQDTSRKEWRWILIRIAQHIGIHFATAFVMLVLLYSSSAPFSQFITSRCLQLLHRIRMSMVMTRVVV

>jgi|Rhior3|10786|Rhizopus_oryzae

MDEICQDDQWFEQAFVYMNEHDIPLSNDKKLHFYGLFKQATIGDINKPKPGLFEFVARAKWDAWNSCKGLSFREARNRYIESVEALKVGWSRQGQYEYIPSPEELKKQELGGLGNAVSSMAFEEEEESDDIFGYARGNNLKKLATALEENKDLINTKDEDGLSVLHYAADRGHFEIAKYLIETGADLNVKTEDGETPLHLGKINQLFLNHLT

>jgi|Rhior3|15608|Rhizopus_oryzae

MYFRDAWKKLENMSQLEAMHSYVETLLKVATEAYRKNMGREEAQKIIHVFAAMNPSGDNDSSDGELSDIDDTSEASNEEERDYLRNIQSPSFRRPPSVASTQTMVTAPATPRQFPMRPPSSMSMTRRGLQTSGRDLLSNTFHEEEEDEQRMMNTNPWVQHPAIKERNVKTPTSVASSLQLPSARSINFTSQDHRRSVYTTQHEPFRALGPATKQAFESLQNEMITLSGRIDDLRIELVERDKKRPIQPKRDEQPKESDRGSEDDGWKWVIKAALKYAGVNFMTAFILFLVLYKTKSPIAYAILEQINPLTLEKESKWILECKS

>jgi|Rhior3|9713|Rhizopus_oryzae

MPSQQFTTAAEEVQKLSTKPSNDELLELYGLFKQATVGDNETSKPTFDIKGRYKWDAWTKLKGMSQEEAEQKYIELVEKLKASQ

>jgi|Conco1|56255|Conidiobolus_coronatus

MSGLNNPEFAKVAEDVKTLTTKPSNDVLLALYAHFKQATVGDNNTSAPGMFDLTGKAKWNAWNDIKGLSQEDATAKYIEIANKAIANQ

>jgi|Conco1|20929|Conidiobolus_coronatus

MEIDNIEESTEENSNDSTEADTEADTEDSSDSTNSENLEQKIEEIKSLYSQLPQLKQNPGYEMLLDLYSHYQQATLGDNTTEKPNILDFEGKSKWYSWYQLKGMTKGEAVEKFMQIYTLIMAKTLKKV

>jgi|Conco1|84662|Conidiobolus_coronatus

MVGATPCPPSRRKFDHAVTVVRHLPTSLKLQSDDRLKLYGLYKQASEEFTYKKKPRNRDWLSQAKWDSWIEVSHLTQEEAQEEYVKTLVEFLKSFPERQQAVDLVTGLERTLLLTDFQLGVYPTWPRTTTRAKRFPKSCSAELNQTKREAILQYCRDSAIYLPTTNSNGHTSILTDNPLDNSLTNEIIPDQYPQYSSQIHLIHNQSFNDNVVAPESPISNATEIPDDCSEDSYASSDDGESLSTVTVRACNHRNYRSSTVSDRHDSFDSGLSSDTELALESLQTQVAALNQKIDLLKQDLRQTDVIRAPKPFWQSFTMDWLKTAALNFFVGVIILTQYYRIRSVHHRSVLVYYFSYFNACCRRFIEAGRKLISREPY

>jgi|Maseb1|517905|Massarina_eburnea

MSDSVDRVFSHALNTVNKIRTGSQKPPSATRLKLYGLYKQAMEGDVDGIMERPEGGRDETHRAREKWDAWKQQNGLSRTEAKRRYITTLIDTMHKYASPSPDTRELVSELEFVWDQVKSNVPSSLSSSPLHKRDSTGSQMAASGYPKLDSGKGKGRADDDNEGPLQAKSPMSQSEEELEDEEADIDREEFVDAPDSQYNPNSEPPAEETDVKTDVKTGLQTQEPVPYATPQKPPGRSIGLLPALFTTPTAAPNGPSYETAADKKWRKRVEQALVKMTAEVAALREQLESRRLFTHSKKFIFFRFLFRLVWRSLKHVAIDVFILSIVLVWMRRKKDKRLEGAIRVLLGDAVAQVQKVGGKQFGKVKLPALVGGAKKVD

>jgi|Maseb1|107623|Massarina_eburnea

MPSAKFDEVYKKTRDIKSGPSNDDLLNLYAYAKVAQGEDIEKAAKPGMMDFTGKAKRKRWQEVVDAGTSKSDAEKKYIELGEGLISKHS

>jgi|Verda1|5407|Verticillium_dahliae

MADSVDRVFVHALNTVKKIPKTGASRPPPSDRLRLYGLYKQAMEGDVDGVMERPAAGAGLSGDELQREKDKWDAWNAQSGLGKTEAKRRYIEALVETMHRYATTPDAKELVAELEFVWNQIKNNSPSASSSKGSDQFVGRRFTQPTSGTEGPLRVLSPMSEDDAAERESRRQAIAEEDGEEEDDAPKRGSKWSRSVENALVKMSAEVAALREQITTGREWRFKKERSFPAWLGWLVWMIMKHLAIDIVLLAFILLWMRKRKDRRLEDLVRAGLKLAREYVRNVLPSR

>jgi|Verda1|497|Verticillium_dahliae

MSVPQSEAFKTAVVDSQKLTSKPSNDDLLQLYALFKIGNGEDIRQSPSPGMFDLKAKAKRSAWQKETDAGTTAEQAQAKYVELVNSLKESHGYDADKVPESVGA

>jgi|Xylhe1|258819|Xylona_heveae

MAQSPEFELAVKQVKQLTKPLGPEDMKRLYGLYKQATQPQTLDEFYANVKKPEGMFNFKEKGKYAGWEQAVKDAPTGEEAQKLYIEFVESLKEKYAFDPNKEPESVKS

>jgi|Xylhe1|262574|Xylona_heveae

MSDSVDRVFVHALSTVKKIPRTGSARPPPAERLKLYGLYKQSMEGNVVGIMDRPAGDNDEERAERAKWDAWNQQHGLSKTEAKRRYISTLIDAMHKYASSTSEARELVSELEFVWDQIKSNAPSSSSSPPLQTNDVPVAQSLSGQEKSGLKILNPVSPGDDEGHELREYVEAKTVDISDIDSDEYENVTERDESGSFARRKFTDEMRRPHRDWRRQVEQALVKITAEIAALREQIESSHRRSVKKRPPLVAFVLWAMGTSIRHLLIDTIIVGVLFLWMRRRKDPRLEDALKVIFNVLREYARRLRYRKRR

>jgi|Trigu1|1083200|Trinosporium_guianense

MAQSPEFEKAVKEVKQLTKPLGPEDMKRLYGLYKQATQPQSLDEFYANVKKPEGMFNFKEKGKYAGWEQAVKDAPTGEEAQKLYIEFVESLKDKYAFDPSKEPESVKS

>jgi|Trigu1|1053985|Trinosporium_guianense

MSDSVDRVFVHALSTVKKIPRTGSARPPPAERLKLYGLYKQSMEGDIVGVMDRPRGHNEEERAERAKWDAWNQQHGLSKTEAKRRYISTLIDAMHNYASSTPESRELVSELEFVWDQIKSNVSSSSSSSPLLTSDMPIPQSLIRQRESGMRIISPVSPNDDEDDEQREYLEARTGDTSDDEDPHEDSIAEVDQVARRTSNTDMVGWSQRGWRRKVEHALVKLAAEVAALREQIESSSQHTVKKSPPLLTRVFWGIGASIRHILIDALILGIVIVWMQRWRRRDPRLDETMNIVSTLVREYAGRLSLKRQRLRS*

>jgi|Clagr3|3556|Cladinia_grayi

MSDSVGKSSLCLRLSTSRCSTDNVNHTDRVFVHALNTVKRIPRTGSARPPPSDRLQLYGLYKQGTEGDVEGISPRPTGNDPDTIAEREKWDAWHAHNGLSRTEAKRRYISTLIETMHKYATTTPEARELVAELEFVWDQIKSNSVSSSSTSPGQLGKGSNAGMDQSYIDPSRRVSGEESTTAAGLRVLRPVSDGDEDDDEDAEFEDAVRHPTAGEEDEDDDAIAVAGTNLIRDPDLRNRKWRRRIERTLVKMTAEIAALREVVDAARTGDSRRRNGVWAWFNWLIWAGCRHVLVDLALLGLLVVWAKRKGDHRVEQGLALLWDYIRAKIRRIRIPGLKRGER

>jgi|Usnflo1|888853|Usnea_florida

MSDSVDRVFVHALNTVKRIPRTGSARPPPADRLRLYGLYKQSTEGDVQGISARPQSNDPESAAEQEKWDAWSANHSLSRTEAKRRYITTLIETMHKYATTTTEARELVADLVFVWDQIKSNPPSSSSSSPGQRVNRRRRQMLPSYAGLSGNSGIQVSDENGGEELRMLRPVSDADEEDDGGEVLEETRGGSFDDEEAIVAAAGQSRDLDIRNRKWRKKIERALVKITTEIAALREQIEAKKIGDGRRRNGLWAFLWITVRHVFVDLALLVLLVAWARRKGDRRVEQGLDLLVQYLRQRTRKVGLPDLSRLSAQQA

>jgi|Chalo1|504537|Chalara_longipes

MADSVDRVFVHALNTVKKIPKTGASRPPPSDRLRLYGLYKQAMEGDVDGVMERPMSIGEGEAEEEDVKKDRDKYDAWDSQRGLSKTEAKRRYIEALIETMHKYASTTADARALVDELEFVWDQIKNNSASSSGSSPGRGGIPSYTTQPRTFQQPLSGDDGPMRVLSPMSQDDEAEAESRRLGYNDEYDDNDEFNDGNKKVEKKTQKWRRTVEQALVKMTAEIAALREQIATGREYQGKRRKSFGRWLGWLIWGVVRHFLVDVVVLGIVLIWLRKRKDRRVEDLVREALKIGREYVRKILPAR

>jgi|Chalo1|435414|Chalara_longipes

MAVSSLNFITGNQTQKLIEQIQQDVAHLRTHADLQEDDADSIEEYSTRACASDDELSISSFQMPLRRYLEETESLLDPETASGRSESITGSADDTWRLNPTITSNISQNSEEADLKAFRRMTSECTAIDRVFIHALYTVKQMPKTGTSRPPKPNRLRLYAFYKQAMEGDVDDVMERPSSIWEGEDEKTMKSIREKYDAWKSQKGLSRTEAKRRYINSLIRTMHKYASTTADSRALLDELEFVWAQVENNIAFPSQSYPMGITVLA

>jgi|Chalo1|564282|Chalara_longipes

MSAFDTAVADSKKLTSKPSNDELLKLYGLFKVATGEDITKAENPGTFDLKGKAKKRAWQAVVDEGLTSDEAKEKYVTLVEGLKAKYGYDATKEPEAVGGGS*

>XP_570515_Cryptococcus_neoformans

MDSATAGLDAQFNRAVDIVQSLPKGGTIQTTYEDKLWLYSLYKQAIEGDVAVPRPGMLDLLGKAKWDAWNRQKGIDKPQAKRLYVSALVKILRKCADAKDVQRYLQILEHESGYEYPLHIPSCPASPASSASSYHSSQASPALLPQSSNSPIVPPHDISPLTPSLPPPDDAPNFIPPSSHHSRNPPSLSSMQEQRNEVLDAVGRTGRDGNMRDQRPSSAQNLTSESQIHAAMPLDRITGIEREAVSGKSGSVHSPRRQRRDSQASARLASPLPHPTVGSRDFLGTPETISSPFLALILRRDERRKSWFSWEGHESEELDNCENDQAKEMWGRTGVTTVTKVNAKKKSISMRVLWFLVKTLRRALMDVGIGMMVTLIVIMVLNGGWGRARWMILKYKERLRRFLTEQ

>XP_568458_Cryptococcus_neoformans

MVNTKAQFDKAVAIVKGLPEDGPVKPTQDDKLAFYAHFKQANEGDVSGPAPGMFDFVGKAKYNAWKKIAGMSKEDAMAKYVELLTEMLKKSDDEASKQYLAELEAAGASA

>XP_571618_Cryptococcus_neoformans

MSTISFASEDQFEAASSWLISTPPAAGLPNEIKLELYGLFKFITTGAGPTSSRPSLFYPTNRAKHDAWDSVSRKYLHLNSVPNQTKAMAQGRYVEIAKQIGWDGVIQEENEIDLERLSDSGSEDGKDGNEGAKDGWRKVSVMQGEDGLDNENDKASVGAWRRQEPEGKPWLLSDVL

>jgi|Aspnid1|5644|Aspergillus_nidulans

MILDRVFVHALNTVKRIPRTGTARPPATERLKLYGLYKQSMEGDVEGVMDRPVGNTADVYMEDAWYAQRGLSRTEAKRRYITTLVETMHTYASQTEEARELVAELEFVWNQVKSNIPSSTSSPVQSTGVPPISQPQSPYGSISAQLAQNNEYQYKTSTARGDSRLRVLSPVSQPDDIYQRRTARMGYDRDQGLDQGGDDESVNLDEDEEEEEYAEAQANLYEDDDEVEGEAGGAVDEDDDDDHHHHHQQVYSSHIPDNSPSRKRDHDIDSWRWRRRVEQALTKMTAEIAAAREQMEARTLAARRRSGVWAWLRWLVWVTLRQIIWDLALLGMLLIWMRLRQDRRLEEQLKAGWSVVKARLAGLKALRDLRKVYIFS

>jgi|Aspnid1|4403|Aspergillus_nidulans

MSVPTFTAALTASQNKEKYSAKVQELAAAINADALSSAIEAILSGGDDATVSDAEQSKALTAGFEYATELVKELKSSPGNDDKLKLYAFFKRSKNEEPAAPGAFSFEAKYKYNAWKEIKDISQQRAQALYIQKVNALLESIGTN

>jgi|Fusox1|17068|Fusarium_oxysporum

MADSVDRVFVHALNTVKKIPKTGASRPPPTDRLRLYGLYKQAMEGDVDGVMERPTAASGMASDDLQREKDKWDAWNLQKGLSRTESKRRYIEALIDTMHRYATTPDAEELVSELEFVWNQIKNNSPSSSLSSPRANRSAGASQPYEDAAQASDAEGPMKEIRPMSEYDEAELRSQKQLELEDDDIDGSQNHDRVSGRWQRKVERALTTMSAEVAALREQITTGREWRAKKERSLPALVKWFAWVVVKHLFADFVILTVVLLWLRKRKDRRLEDLVRAAVKLVREYVRNVLPSRG

>jgi|Morel2|14613|Mortierella_elongata

MACVSVCLGSLQPFNLELQTPEPSAEFDAAAKKIKELTVSPSNDNLLKLYALFKQATVGDNDTTRPGAFDFKGKAKWDAWTEKKGLSQEDAEKQYIELVKDLTA

>jgi|Morel2|19676|Mortierella_elongata

MLKDRFNSAVAYMSSRKDLKLPNPTKLALYADYKLATEGLCTQPRPSLLEFEKSAKWKAWKETGDTYSQELSSTTTTSEETDTAEFNISTKAMIAYIQKVEEGQWGWVFDPSTIADPSSTLAAVLPTGDSDLDELQAYLGVDKEEISAEELLARPYVPHENTQFDPSTMTAAGISTMAMPAEEEGEDALEAAKSGSVEALQKTLSANPELGMTMLHWACDRGSLKKVKLLVETYNVDVNALDDEGSTPLHYACLSGWPELATYLQSLPAVDQSIKDHSGMTAAEYLD

>um11226_Ustilago_maydis

MSSADVIDALFVKTVDMVQSLPKSGPIQTSYEEKLALYSLYKQATEGDVSSRRPGMLDMLGRAKWDAWSKVSGLPPRDAKQMYVESMLRILRRFSDRPQAAALIQELENFSGQVAQRVMDGSLAQVDSDEESITDSDPDDQQRLDPTGLHPHTSALPRATQPPLPHHLAPHPPSVTGTSTAATRGGGGATSIISPERTPISSDSEHDSSDDDRLGSQPGPFRGAPRHPDSCAPPSVQSYRPQPRAGSISTTEGYGRGPPPRSHVSTSAQYSRGPPSLQGSYSRSQYAPQSTAATVAASSHPRQGEAGHYQRSTYAASSVGGRSVGTRTGPVPVYPPRPPSNAGITRGQPRAEMETALQSIQASLAALHERLNRVENRSSAGGADDARRSRPGSGQWSSTYLYNAIMNIFDDVRQLIGLDAVARGPRTEALAPSFLAQGGGAGKRSNAAFNLLTSPVRILVALANLSARLALDVISLVLVSSFFLYAFKRITGRGDPLLMIRLLKRLQQVVFRRSAAAKQLVAEAGA

>um02959_Ustilago_maydis

MSAEAKFNKAVSIVGSLPKDGPVQPTQDDQLTFYGLYKQATVGDVTSKRPGMFDLAGKYKWDAWNKNQGMSKEDAQQAYVDALLEILKKHEDEGDSAQYIEQIQNA

>jgi|Basme2finSC|337109|Basidiobolus_meristosporus

MSNPPQPDPFTSARFQKALQIISSFPVDSSTLNPTIQERLEFYGLYKQATVGNCTISKPPIYDLVARSKWESWCSYKDLEKQHAKERYVELLVQFLKKFPDIPQAITLVDAFERSRRGSDPEMSNVGNEVLAKSESDTDSPVPLEEGDFYSDEDQMGQSGNMSELSGTYGNKKSRSLTNYQGQLFANPSYMPGNSNHLANSSSSIFSGYQGKGGYTQPTGRSRDDYRDDGGESSSNPEAERALEYLQTQVAALNERIEILRREIVDREKKKLQEKHGIKHVLKTIIKHAFIDFVIVAILFTYFYRKGHPLAFIIGTQLRQHLTQTLLTVVATLKDAPKLWKLIKWSPRQS

>jgi|Basme2finSC|346410|Basidiobolus_meristosporus

MSDAASTGNTAFANASEEVKTLATKPSNTQLLELYALFKQGIFGDNTAERPGMFDIQGKAKWDAWTGKKGMSKEEAQAAYIALVESLKN

>jgi|Picpa1|36433|ACBP-L_Pichia_pastoris

MVSQEFNDKAEAVKNLKTKPNNDELLKLYGLFKQATVGDNTTEKPGVFDFKGKAKWEAWDKLKGTSQEEAEQEYIAYVGDLEDKYN

>jgi|Picpa1|39979|ATG37_Pichia_pastoris

MSESIDRVFVKAIGTIRTLSSRTGYGGLPRPPIENRVKLYGLYKQATEGDVAGVMERPLGDSPEAEAAKRKWDAWRSEQGTSKTEAKRQYISYLIDTMKQFASDTTEARELLSELEYLWNQISDVSPNDSSDSESNAGPAQLLQNHAQLLSRDISVVDDPITSSGMDPMYNPSFQRHNSSRFINASTAERLNSLSNYYSNLNPTPPLSSRRYQGSVTPRNVDFIKWQNDINNSINKLNHDLQLLANRRLQSSASDPLYSKRGSDLTHDDFVNDISSSSSNRRFRARRNQPLVSKVLLGTISLLLKLIKTVIKHVAIDAVIIAVLVAVIKRSIIIPNLISNEISLQKIHHSELESNSSIKGDSNGGRLTIVLPFINGKDFFQENSLLGKLLKVFHDYVDHVSRIRLIKRN

>jgi|Ascru1|87612|Ascoidea_rubescens

MSDSIDRVFDQAIETIKALSTRSGYGSLPRPPVESRTKLYGLYKQATEGNIDRMLPVRPVGTSPYDEAAKRKWDAWKDQEGISRTEAKRRYISFLILTMKTYASGTQEARELLSELEFLWDQIKDIIPSPSPTNSQIYENPQSVKDPFIYNSISMLGSESNYNPLSSINKQQPQSQINLPTILQASFDENTLPKSSFQNLCYTNYQQHDYHHPSQLSTSTSIRNLPNYEYFKNLKKDVHSIPSNYDMPNRYRSLNDNSPNFNFFNFKFPLLSPGQNPNKNSNDNAITANNNNNRINNRSLIHNNFDNSSSHENESFITKIQIIIPKIKEIFTMVCHQIYKIYLPVSKSFFIATIIILILCKYLQKTGSLPIDKNFIDIPLTFKSTKTIDKFSKSNIYNLILKLVLFIFTNLNKFLNFNIKTIRLIVK

>jgi|Ascru1|77692|Ascoidea_rubescens

MVSQLFTEKAEAVKTLPKTPDNDELLELYGLYKQATIGDNKTDKPNAFNFKAKYKWEAWKDLEGTSQEEAEELYIKLVDKLIAKYTS

>jgi|Zygro1|529|Zygosaccharomyces_rouxii

MVSQLFEQKAKQANELPSKPDTDELLKLYALYKQATVGDNNKEKPGIFNMKDRYKWDSWEELKGKSKEDAEKEYIEFVEELQQKYSQ*

>jgi|Gloin1|343716|Rhizophagus_irregularis

MTDINRSAEFDAAAKEFEEVVKNHNPSDEDKLEGYALFKQGSFGDNTKPEPGFFARTDKAKWNAYNTKKGITPEDAQKQYVDFVAKMKEKYGS

>jgi|Gloin1|349696|Rhizophagus_irregularis

MTDTQSSLIFNNAAQDFATHAKANKFTAEEVLEGYGLYKQAIAGDIPSEKNNSDAVQDMAWSMKKGIARDDAASQYIAFVDKLKSKYSL

>jgi|Synplu1|692118|Syncephalis_plumigaleata

MAEATGNAAFIQAAKDVVTFKSADNNESKLKLYGLFKQATVGDNETARPGLFDPAGKYKWDAWTENKGISQAEAQQKYIDYVEELRTKESS

>jgi|Synplu1|775840|Syncephalis_plumigaleata

MTSIGASDQYTSLDRQFNLACRIVRECQRPARLSGESKDGKLPRLSLAESNSFYGLFKQGTKGDCSRSRPGRWDPLGRGKWESWKSLMGVSSVEAKQRYVDSVMMYLQKHINHPRATELLVQIKSFDTNASSDSSRPPPSQQEDIANVGTYNTINDVDISEHALHDGTDDSSPLESEEHLPTNDDTQLPYESSIISSTSTRTDNNVRDVESIDENVKNTLTALQERLEHVRQAEETLEKLQTELTAIQERIEQHKRESETRSRADRARGTGPRSWLRALLRHAFVHSTLLVFSLVVYRLTVARQRGYPGSTLVSSVANINAQQVASMDIESAVGRPAWPISILVRWIDSWLSQMSSLIRRLFRLR

>jgi|Synplu1|666192|Syncephalis_plumigaleata

MTSIGASDQYTSLDRQFNLACRIVRECQRPARLSGESKDGKLPRLSLAESNSFYGLFKQGTKGDCSRSRPGRWDPLGRGKWESWKSLMGVSSVEAKQRYVDSYLQNISIIQGTELLVQIKSFDTNASSDSSRPPPSQQEDIANVGTYNTINNNDNNVRDVEAIDENVKNTLTALQERLEHVRQAEETLEKLQTELTAIQERIEQHKPDRARGTGPRSWLRALLRHAFVHSTLLVFSLVVYRLTAARQRGYPGSTLVSSVANTNAQQVASMDIESAVGRPAWPISILVRWIDSWLSQMSSLIRRLFRLR

>jgi|Tapde1_1|1756|Taphryna_deformans

MSKADFDKAAAEVKNLPSKPSDDDLLNLYGLFKQATVGDNTEAKPGMFDLKGKYKWEAWNKRRGLSKEDAQKEYIDLVEKLKAK

>jgi|Tapde1_1|18|Taphryna_deformans

MNGIDRVFVSALNTVKRLPSSPSSPKPPLDERLLLYGLFKQSMEGDIPQSMLQTLQSDPIDEEDDANREKTEAWAEQKGTTKTEAKKLYITTLIRSMRQYGSKTETAKSLIDELEFVWNQVNGNNDESSPLHSRSNSGSHANLVGQQEDDDDDDDERRVDLDQALPIAYRTAPPSPSKALQPQKTGESRSFNPVTMLLGGSAKPSTTLSQDEKWKRQVEQALQQIRAELASVRESLLITSPSASSLFSKHNTYHQQSLLTQVLSRMSRLLRVSLTVALYDIIFLLGIWYFFRRRGDKRAKRVEEWVRNLYDWTLRKIRMKVRTGVRSIGDRR*

>jgi|Coere1|78996|Coemansia_reversa

MSNVNVELSAEEKARLENLNKEFLEAKKNSEVLPKKPSNDEKLKLYSLYKQGTVGDNDTEKPSAFSFERKYMWDAWTKLKGMKSEEAKQKYIDFVKELEEKFKKELE

>jgi|Linpe1|263816|Linderina_pennispora

MSEAASTGNAAFVTASQEVTKLSTAPSNDIKLQLYANFKQGINGDNTTPAPGFMDFTGKAKWNAWTALKGTSKEDAQAKYIALVEELKKTHA

>jgi|Blabri1|377594|Blastocladiella_britannica

MAETTGNTAFIKAADAAKTLTYQPTNDELLELYAFFKQGTVGDNDTPRPGMFDIKGKAKHDAWSAKKGLTKEDAQAKYVELVEALKLK

>jgi|Blabri1|345767|Blastocladiella_britannica

MTSPPAALAALFSRAVQAIQNLPPAGPTDAGLAPTNTEKLEFYALYKQATTGPLDAATTKRPGFFDPVGRAKYDTWLGKVRLTRAEAMRAYTDLFMAFLRR

>jgi|Blabri1|377593|Blastocladiella_britannica

MPSDAFNTAANEVKSLKTTPSDAQMLEVYALFKQATVGDVQGERPGMFDLKGKAKYDAWAAKKGTSTDAAEKEYIALVESLKASHGI

>jgi|Allma1|14327|Allomyces_macrogynus

MHGADPQLAARFARAVDLVQSLSPADAADAPLAPSNAEKLRFYALYKQATRGDATGPRPGLFQLVARAKYDAWTALRGTPRAQAMSEYVEMLLAVLRRFPDRPQAQAVLASFTEERASGETLKVPNGGSGLPRVPSAVSTGTAHHDERYFSADEPEPESDGELDDGEDEEEEDEDDDTARDPAATVDDLPVPRSRAWRPSSTPVADGASIASGVTVRAIVLPPPSPTLRSASPGSPPANPAADESWTRASASTTNSSRASLSPRPRTLDSEPTPPQPRTLAAALAVIADLRAQLAGAKAAASIAACPVAGRRTPSPRPMPAVARARHVIGWIARHVGVAVILAVVLAAVMRDLRRRQSPAYAWAVVAVRAVVEG

>jgi|Allma1|3154|Allomyces_macrogynus

MPSDAFTQAAEEVKSLAYQPSNDELLELYALYKQATVGDNTTARPGMFDLKGKAKWDAWAAKKGLASEEAEAKYIALVEEFKKK

>jgi|Rozal1_1|5921|O9G_001025_Rosella_allomycis

MSDPKSTNNPEFIAAADAAKSLKTTPSNDDLIKLYSLFKQAIEGDNTTPQPGMLDLKGKAKWNGWNSQKGKSKETAQKEYIQFVKELQSK

>jgi|Rhihy1|780965|Rhizoclosmatium_globosum

MPSADFEAAAAAAKSLSYNPSNDELLALYGLYKQATVGDNTTERPGLFDLQGKAKWDAWEKNKGTSQADAEAKYIELVKSLQAK

>jgi|Rhihy1|853591|Rhizoclosmatium_globosum

MDFDEATKLVAANPSAFSQQTLLRLYALYKVATVGPCNVPSPGFFAFEARAKWDAWNNLGSSHTPEMARLKYAELVEERFDFGYETSSSTRSSIYNNTDGDDGALGFTVSTLEKPDESALVVKEDDEKLLLDWVQTGDVSLLDAFLKKNPTIDLDQGRPDDGVTALHWACDREWKPIVELLVANGANLNVQDQEGMTALHYASLTENRDIYDFLIQAGADPSIRDISGELPSLKQ

>jgi|Neosp1|699273|Neocallimastix_californiae

MLSETDKKFAEAVHIFQNLPQELRPTQEEELEFYGLYKRAVVGKCDISKPNFDNILGLAKWESWKKLDSLSMVKAREIYVDQLIKLLERFPDRPDVVELLDTFTKLHLNENDDGISEKSAVKANTIPSNTSDSINNEYQMDNSHVEYSSRDINTNQSNSLNMNTSMNSSINITSPRLSRNNSNQYNPEELININTNNNNRQVRSRSRTDGSIYQALPADISLNLMHSEDPSSLRSETFLNSSMTNPSLNPKSTPLNPSMSPLPNMSTPNMNPSIGMGMGMNMGMGMGMGMNGLNGLNNINGSNKLPPTTPMSIVMLTRLRDLEIEVNSLNTRLNMLTNMIKNNTKNSLQNWLKFILKTFGANVVILLFMIIILGPNGFNKLISQFPAWLLKLQSRYIANSKPGSSGALTHV

>jgi|Neosp1|386106|Neocallimastix_californiae

MSGNEKFDTAAVEVKQLTYRPSDSELLELYSYYKQGTIGDNETDKPGIFDLKASAKWKAWNKVKGMSKEEAQSKYIELVEILKAKN

>jgi|Pirfi3|272368|Piromyces_finnis

MLSETDKKFAEAVHIFQNLPQELRPTQEEELEFYGLYKRAVVGKCNIPKPDFFNILALAKWEAWKKLDNLSMLKAREVYVEQLIKLLERFPDRPDVVELLDTFTKLHLNESDVEIVSTESQGKANTINSTTSDVVINNDYSLGDIDNQHVEYAPRGINNQSNSINLNNTSVNSSINGNLMSPGILHNSNSQSTDDMININTNTRSVPRNRSRTDGSIYNTTPPDISLGTLQPEDPTSFRSDNTFISPSMNMNNPSMNPKIASPLHPTMSPLNPNGMGMGMGMGMGMGMNMNNPNAKIAASTSMSIVMLTRLRDLEIEVSSLNTRLNMLTNIIKNNSRNSLQNWLKV

>jgi|Pirfi3|348160|Piromyces_finnis

MSGNDKFDTAAVEVKQLTYRPSDSELLELYAYYKQGTVGDNETEKPGIFDLKGSAKWKSWNKLKGMSKEEAQAKYIELVETLKARN

>jgi|Neucr2|3259|Neurospora_crassa

MADSIDRVFVHALNTVKKIPKTGAARPPPGDRMRLYGLYKQAMEGDVDGVMERPSAATAYGAAPEDIAREQDKWDAWNSQKGLSRTEAKRRYVEALIETMHRYANNTPNALELVAELEFVWNQVKSNSPSEQSLAGGGLQQQQQQPQQHQPYNNGGHGGSYGYGSVGPYGQSPGAGVRRFQHPLSGTEGGLRVLSPMSEEDESERRMREEAEQELFGADEFPGEYAKRNDKRTKRMERAIVRLSAEIAALREQISSGREWKTRKEKSFTNWIGWWFWVLVKHFIIDLFILVVLLLWMRKRKDRRLEDYVRGTLKLAREYARLVLPSR

>jgi|Malsy1|3373|Malassezia_sympodialis

MSSAKVIDALFERTVDIVQSLPPSGPIQTSYEEKLALYGLFKQATEGDIQTRRPGMFDMLGRAKWYVAHTHARDAWERQRGFSKQDAKQLYVESMIKILHRFEDRPVAISLMAELEAYSGDVAEQVMSGTLAETASIHSSSVGQSPRQRALNAVPSVDESSLADEPPRAPSRLPPVTSVEGDTHTGSGSHDTITGSDMPRPAPRAAPEKARRADRGVLPVRGAPATQRRAAPRPRASLARRDESMYSGAAASDRGSRSFASAAPSLPPSDVPLAPHAGGPLGRYASSVGGRSGGPVPPARSALAKGARAGAPGMGERAPELEESLRAIQASLTALTDRLDRAESRMVRRENALDVRRLHQYMQRMLHDIGACLGLVPGCPGEANAPSYDAWRARHHGLWALVRSPFKFAVLVASLAFRVLLDLASITLMVTLLVAALRRLSGRSDPWITLRLLGQLRGRLQWLASPANRQAALRALLTSMVLGGVAMESTRSLRS

>jgi|Malsy1|2953|Malassezia_sympodialis

MSDTQFQKAVEIIRNLPKNSPAKVTQAQQLRIYGLYKQATEGDVSTSRPGMLDFTGRAKWYVCFGIDSFYRDAWNSEKGKSSEQAKKDYVDLFIEVHDAEFNKYLEEVKNA

>jgi|Psean1|71310|Pseudozyma_antarctica

MSSADVIDALFVKTVDMVQSLPKSGPIQTSYEEKLALYSLYKQATEGDVSSKRPGMLDMLGRAKWDAWSKVSGLPPRDAKQMYVESMLRILRRFSDRPQAAALIQELENFSGQVAQRVMDGSLAQVDSDDESLTETDADDQQRLDPTGLHQPPSGPSRQQPPPPQLPAHLAPHPPSVTGTSTAATRGGGAASAISTQRTPLASESEQDSSDDDHLGSQPGPFRSVARHPTPHAPPSIHSYRPQPRPGSVSATEGYARGPPSRSNVPTSSVHEASTTLRPRGPPSLQGGYTRHTYAPQSTAASVAGSNYPRQPEPGHYQRSTYAASSVGGRSVGTRTGPAPVYPPRPPSNAGSSHQPPRPEMELALQSIQASLAALHERLNRVEGRASSAAADDARHPSSSNQPATARLYHALMNIVDDVRQLIGLNAVARGPRSEALAPSFLAEGGGATNRSGAAFHLLTSPVRILVALVNLGARLALDIISLALVSSFFLYAFKRVTGRGDPLLMLRLLKRLQQALLRRAPASAKQLVAEAV

>jgi|Psean1|70307|Pseudozyma_antarctica

MSVEAKFNKAVSIVGSLPKDGPVQPTQDDQLKFYGFYKQATIGDVNTKKPGMFDLTGKYKWEAWNKNQGMSKEDAQQAYVDALLQILKKHEDEGDSAQHIKDIESA

>jgi|Batde5|86602|Batrachochytrium_dentrobatidis

MSDPASTGNSDFIKAAADVKNLATKPNNEELLQLYALFKQSISGDNTSACPGMFDLQGKAKWSAWEKVKGTSKEDAQAQYIALVKQLQGKN

>jgi|Ganpr1|67034|Gonapodya_prolifera

MADLTAKFEQAAKDVNDFKSRPTDAELLELYGLYKQGTVGDVNTAQPGWLDFKGKAKWDAWKKLEGTSKEDAQKKYIEVVEGLKRRTFRGFNGVELVADAYGDPTKQCVLFLHGGGQTRHSWGGTAVHLAGLGFYAVSMDLRGHGDSGWDPKSDYTMPAFEEDVVSVCEELKGPIVVGASMGGTAAIWSAYRAKIIGIVLVDIAIHNEPVGISRIVGFMTSRTEFDSLDDAADYIAAYLPHRPRPKDTTGLAKNLRLKPNGKYRWHWDPAFMTSDRGRSVRDTNSDNSDTGDRINGLEARAKQIKCPCLLIRGKMSDVVSEKSAQQFLELVPHAQFADVSGASHMVAGDKNDAFTTAVVKFVKSFQSHV

>jgi|Pench1|32856|Penicillium_chrysogenum

MRNSQDASADEALARMGYKSELPRNLSMLSILGLSFAIMAAPFGLSTTLYITLADGLSVTIIWGWVFVSLISIAIAASLAEICAVYPTAGGVYYWSAMLSTRRWAPLMSFIDGWLTLVGNWTVTLSIIFSGGQLILSAISIFDESFVANAWQTVLMFWAVMLFCALVNIFLSRYLDLINKVCIFWTAGSVIVILVTLLTMADNRRDAKFVFAHYDASTSGWPDGWAFFVGLLQAAYTLTGYGMVAAMCEEVQNPHREVPKAIVLSVVAAGITGLFYLIPILFVMPNVQMLREVASGQPIGLLFKTVTGSAGGGFGLLFLVLGIMLFAGIGSLTAASRCTYAFARDGAIPGFKLWRRVNKKLDVPVWAVVLSAVVDGLLGLIYFGSTAAFNSFTGVATICLSTSYGLPILISLVRGRRDVKSSSFSLGRFGFAINCVTIAWIVLAVALFCMPVTLPVTPESMNYASVVFAGFAGISIFWYFVYARKHFTGPPISGDEARAATELMTGTPVDAEKPLETMKKLPSEANRTAYRVMSDSVDRVFVHALNTVKRIPRTGTARPPAAERLALYGLYKQSMEGDVEGVMDRPIGNTPEVHAECEKCFFSVSASDHHAYNVLARDAWYAQRDLSRTEAKRRYISTLINTMHEYASQTPEACELVAELEFVWDQIKFNAASSSGSSPLNAVGVPPLSRSAYGSIGSRLAQEDYDRPARRDDTSRRDRDSRLRVLSPVSQPEEEEYYRRRRGSVENSEDGGRHNEDEDEDSEEFEEARDSLYEERDQDDHDDDDPDHTNSVSNTHTHTDVSSHHRPKHRRNPASGISIDSSGNGLPHRDSHSDQPRKDSRWRRRVEQALTKMTAEIAAVREQMEARSVAQRRRSGLFAWLKWMIWVAVRQIVVDIAMLGMLLIWMRLRGDRRVEHRLKTGWAAVKVRLGKLKLLKIVDMPILP

>jgi|Cocca1|34333|Cochliobolus_carbonum

MSDSVDRVFSHALNTVNKIRTGSQKPPSAIRLRLYGLYKQAMEGDVDGIMDCPRGNDDQSQRAREKWDAWKQQNGLSRTEAKRRYITTLIDTMHKYASPSPDSRELVAELEFVWDQVKSNVPSSSSSSPLRHSDHIQSGYSQHELGESRRAAMEMSAPLRRNDETPTREAVRDMPLRVKSPISQSEENLEEEEAEIDRGEVFVDAPDSQYNPTSYPDENDENDDTNNLEDAGVQTDLQNLMRAEPIPIQTPTPAPKSRGMAVLGKPIQMPIPGFGSTPGPNNAAKESAADQKWRKRVEQSLMKMTAEVAALREQLEARRLFAHSAHYRLFRGIWRWVWACFKHVVVDAVVLGIVLLWMRRKKDKRLESTIREVLGDALAKLGRGGARIPFLGGRKEA

>jgi|Botci1|14893|Botrytis_cinerea

MPDRVFVHALNTVKKIPKTGASRPPPADRLRLYGLYKQAMEGDVDGVMMRPSSRGDGEDGEAGLQGEELKRERDKWDAWDAQRGVSRTEAKRRYIEALIDTMHKYASTTADARELVAELEFVWDQIKNNTPSSTGSSPGHRMPSYTTQTRQFLEPASGTDGAMRVLSPMSQDDEAEREFERRLVDNGEDEEVYTDGKNNKTKWRKNVEQVLVKMTAEIAALREQIATGREYQGKKSRSPRKWMAWFMWIAIKHFLVDMALMGLILLWMRRKKDRRVEDLVREGLRIGREYVRKVLPSR

>jgi|Maggr1|112258|Magnaporthe_grisea

MDNSRTRAVSVGCARIQPSRKSIPDRQPHRSYLVGVTTPLPPWRTLLTGATRPPPADRMRLYGLYKQAMEGDVDGVMERPAAASGIPVEDLQREQDKYDAWNMQKGLSRTEAKRRYIEALIDTMHRYATTENALELVSELEFVWNQIKGNSPSSSEQLSNDNSPRRFQKATSGTDGPLKVLYPMSEDDEAEHDYQRRMAAAALDENDDDDERYVKKGDKWSKKMERAIVRLSAEIAALREQITTGREFRSRRERSVGAWIGWFLWLVTKHLAADMAILAIVLVWMRRRKDRRLEDHVRSALRIFREYVRKVLPSR

>jgi|Tubme1|3798|Tuber_melanosporum

MSDSVDRVFAHALQTVRKIPKTGSARPPPEDRLKLYGLYKQSMEGNVNGVMERPSGYGNESKAERDKWDAWNSQAGLSKTEAKRQYITTLIETMHKYSSNTPEARELVSELEFVWEQIKSNVSSLSTPSSSTSPFAQHSSALLPMPSGSYPDFGGDSQHHLGLVSPNPFGEEEFDDEDTGMDTDRRSNRKWRRRVEQALAKMATEVAALREQLETARARERRGRLRNWVLWTIYVVGRHLVIEAVFWGLVFLWMRRKGDRVAQDALRLVIRFVRERLKFGSLRGKKGVKTAS

>NP_001056611_Oryza_sativa

MGLQEEFEEFAEKAKTLPDTISNEDKLLLYGLYKQATVGPVTTGRPGIFNLKDRYKWDAWKAVEGKSKEEAMADYITKVKQLLEEASASTS

>NP_001061062_Oryza_sativa

MGLQEDFEQYAEKAKTLPESTSNENKLILYGLYKQATVGDVNTARPGIFAQRDRAKWDAWKAVEGKSKEEAMSDYITKVKQLLEEAAAAAS

>NP_001050536_Oryza_sativa

MGLQEDFEEYAEKVKTLPESTSNEDKLILYGLYKQATVGDVNTSRPGIFAQRDRAKWDAWKAVEGKSKEEAMSDYITKVKQLQEEAAALKAVLSVEISRKFDRNILQTCKFSGHGCPEKLPGQKSSNSSEFLVAGAH

>NP_001051819_Oryza_sativa_Kelch

MASSGLAYPDRFYAAAAYAGFGAGGATSSSAISRFQNDVALLLYGLYQQATVGPCNVPKPRAWNPVEQSKWTSWHGLGSMPSAEAMRLFVKILEEEDPGWYSRVPEFNPEPVVDIEMHKPKEDPKVILASTNGTSVPEPKTISENGSSVETQDKVVILEGLSAVSVHEEWTPLSVNGQRPKPRYEHGATVVQDKMYIFGGNHNGRYLSDLQALDLKSLTWSKIDAKFQAGSTDSSKSAQVSSCAGHSLISWGNKFFSVAGHTKDPSENITVKEFDPHTCTWSIVKTYGKPPVSRGGQSVTLVGTTLVLFGGEDAKRCLLNDLHILDLETMTWDDVDAIGTPPPRSDHAAACHADRYLLIFGGGSHATCFNDLHVLDLQTMEWSRPKQQGLAPSPRAGHAGATVGENWYIVGGGNNKSGVSETLVLNMSTLTWSVVSSVEGRVPLASEGMTLVHSNYNGDDYLISFGGYNGRYSNEVFALKLTLKSDLQSKTKEHASDGTSSVLEPEVELSHDGKIREIAMDSADSDLKDDANELLVALKAEKEELEAALNREQVQTIQLKEEIAEAEARNAELTKELQTVRGQLAAEQSRCFKLEVDVAELRQKLQSMDALEREVELLRRQKAASEQAALEAKQRQSSSGMWGWLVGTPPDKSES

>NP_001054292_Oryza_sativa_Ank_N-term_TMD

MGGDWQELAQAAVIGLLFAFLVAKLISTVIAFKEDNLRITRSTPTFPSAADTPAAPAPPPASLDGGHGDTSDGSGSDSDSDWEGVESTELDEDFSAASAFVAASAASGTSVPEQAQLQLYGLYKIATEGPCTAPQPSALKLKARAKWNAWHKLGAMPTEEAMQKYITVVDELFPNWSMGSSTKRKDEDTTVSASSSKGPMGPVFSSLMYEEEDQGNDSELGDIHVSAREGAIDDIAKHLAAGVEVNMRDSEGRTPLHWAVDRGHLNSVEILVNANADVNAQDNEGQTALHYAVLCEREDIAELLVKHHADVQIKDEDGNTVRELCPSSWSFMNLAN

>NP_001049528_Oryza_sativa_C-term_ACB_N-term_TMD

MELFYELLLTAAASLLVAFLLARLLASAATASDPRRRAPDHAAVIAEEEAVVVEEERIIEVDEVEVKSARARECVVSEGWVEVGRASSAEGKLECLPEEEEAPAKAARELVLDAVLEEREEEGQVGEERCDLAAAVAEVVGVKPHELGVEAAPGEVSDVTLEEGKVQDVGVEQHDLVAEAAPREALDTGLEKQGVPIIEAVEIKRQDDLGAEVAPSDVPEVEFEQQGVRIIEAIDVNQHHRVALAAPAEVVDAGLEERVQAIEAGSSGLTSETVPEEVLDELSEKQEEQVIEEKEHQLAAATAPVAIPGVALAETEELKEEQSSEKAVNVHEEVQSKDEAKCKLHLVDQQEGSASKVELVGRNTDNVEISHGSSSGDKMIAELTEEELTLQGVPADETQTDMEFGEWEGIERTEIEKRFGVAAAFASSDAGMAALSKLDSDVQLQLQGLLKVAIDGPCYDSTQPLTLRPSSRAKWAAWQKLGNMYPETAMERYMNLLSEAIPGWMGDNISGTKEHEAGDDAVGSVLTMTSNTINQHDSQGNEDNTGMYEGHLTSSPNPEKGQSSDIPAE

>Glyma14g09680|Glycine_max_C-term_ACB_N-term_TMD

MELLTVVDDFFVTASLALLLTFIVLKLVEVINDTHAIPKRHVVHREPDRPVSHAEQRFTVQPAPAKTKVGFVSPVQEEFATCAVGTEHKIEKKAAVKPVRPRGKRFTVHPAQSKSGVETECNREEIAVEPERKIEEAVVEPVPVQTEVTVGLISPVQDDTCVGTAEKIEEATTVESDKNVEEEIAEGNEGTQELDDSAEKRNVKSVEEISVEPSTEIEASVTDSGVKENFDDDDDDWEGIERSELEKEFMAATKFVSGEENRLGGAGSNLRMELYGLHKVATEGPCREPQPMALKLAARAKWNAWQKLGNMNPEVAMEQYVSLLSDKFPGWMKDTSAGIGEHETTRPEVSESAASDLSTTLSNQQQMITTERELEQESDSKDRSPLTVSDLENSVNK

>Glyma11g05360|Glycine_max_C-term_ACB_N-term_TMD

MELLWELAFTIALSLLLPLVFLKLLSVTPNLEANEKVALLGRDHDHGIKSDSKSWETDKVVRIGGKIDEFRDKPIVGKLIVPEIVDVSCGSPKIHNSEKIDEYSVYDEIELVDLAEDPVVDEGNDGVVNIDEVEVELMECDSRENKVEEVEISQCERNYNEIDESSMNEEIGENKGSVVDEDDWEGIESTELERRFGAAVVFVGSKSNANLSNDVKMKLHGYHRIATQGPCHEPQPMALKFSARAKWIARRQLGIMSPEEAMEQFISLLSESIPDWIEENPYDNAKPASATNLVI

>Glyma06g13630|Glycine_max_Ankyrin_N-term_TMD

MAEWQSLLQSILVGLIFSYLLAKLISIVVSFKDDNLTVTRASAAETTATRAEQFEDALKRDDAVSSDARPFEEESVVAEHGSVRIDSDGDYDDDWEGVESTELDEAFSAATAFVAAAAADRLSQKVSSDVQLQLYGLYKIATEGPCSTPQPSPLKMTARAKWQAWQKLGAMPPEDAMQKYIDIVTEIYPTWLDGSSLRNKSGDSGGHGSEAKGPMGPVFSTFVYEEEYGSDSQMEAIHGFAREGDMANLLKCIENGVSMNLKDSEGRTPLHWAVDRGHLNVTELLVGKNADVNAKDNDGQTPLHYAVTCEREAIAEYLVKHNADIYSKDNDGSSPRDICESNWPCMQHVGEEVN

>Glyma04g14650|Glycine_max

MGLKEDFEQYAEKAKTLPPTQSNEDLLILYGLYKQATVGPVNTSRPGMFNMRDRAKWDAWKAVEGKSKDEAMSDYITKVKQLLEAAGMPA

>Glyma04g41220|Glycine_max_Ankyrin_N-term_TMD

MAEWQSLLQSIFVGLIFSYLLAKLISIVVSFKDDNLTVTRAAAAETTTTTRDDAVSSDARPFEEESMVAEHGSVRNDSDGDYDDDDWEGVESTELDEAFSAATAFVDAAAADRLSQKVSSDVQLQLYGLYKIATEGPCSTPQPSPLKMTARAKWQAWQKLGAMPPEDAMQKYIDIVTETYPTWLDGSSLRNKSGDSGGHGSEAKGPMGPVFSTFVYEEEYGSDSQMEAIHGFAREGDMANLLKCIENGVSMNLKDSEGRTPLHWAVDRGHLNVTELLVGKNADVNAKDNDGQTPLHYAVTCEREAIAEYLLKHNADIYSKDNDGSSPRDICESNWPCMQHVGGEVN

>Glyma17g35480|Glycine_max_C-term_ACB_N-term_TMD

MELLMVVDDFFVTASLALLLTFIVLKLVEAMNDTHAAPKRHVVHLEPDHPVSRAKTKVGLISPVQEEIATCAVGAKHGIEKKAAVKSVRPRGKRFTVNPAQSKVKVEPVPPAQSESTIGFVSPVQSAMCVETERKVEEAMVEPELVQLVKTEGTVGFISPIQADTCVGTEDKKEEATVEFDSHVVVESPVKSDTDIVVTEEIAEGNEGTQELDDSAEKRIVESVEEINVEPSVVDSDGKENEDDYDDDWEGIERSELEKEFMAATEFVSGEENRLGGVGSNLRMELYGLYKVATEGLCREPQPMALKLAARAKWNAWQKLGNMNPEVAMEQYISLLSDKFPGWIKDTSAGMSEHETTRPEVSGSDASDLSTTLSHQQMIITERELEQESDSKDRSPLTETDLENNVNK

>Glyma01g39920|Glycine_max_C-term_ACB_N-term_TMD

MELLWELAFTIALSLLLPLVFLKLLSVTPNFEANEKVAVIRRDHDHRVESDSNSWETDKVVQIGGKIDEFRDKPIVGKLVVPEIVDVSCGSPKIHNSEKIDGDRLHNEIELEDLAEDPVVDEGNEGVVNINKVEVELMECDSRENKVEEVEISQCERYYNEIEESSMNEEMGENKGSVVDEDDWEGIERTELERRFGAAVVFVGSKSNANLSNDVKMKLHGYHRIATQGPCHEPQPMALKFSARAKWIAWRQLGIMSPEEAMEQYISLLSENIPDWIVENPYDNAKPASAKLTL

>Glyma19g42270|Glycine_max_Kelch

MARASSGLQYPERFYAAASYVGFDGSSPTKSLTSKFPQSTALLLYSLYQQASVGPCNITEPSTWKIVEHGKWASWNQLGNMSSTEAMRLFVKILEEEDPGWYSRLSNSVVEPVVDVQMNHNSKVEPVIENGNSYPETKTISISTQNGSEVGTQDKDTVVEGFGSVEVYDQWIAPPVSGGSPKARYEHGAAVVQDKLYIYGGNHNGRYLNDLHVLDLRSWTWSKIEAKTGVESPTTSIPCAGHSLIPWGNKLLSIAGHTKDPSESIQVKVFDLQMATWSTPKIFGKAPVSRGGQSVNLVGKTLVIFGGQDAKRTLLNDLHILDLETMTWDEIDAVGVPPSPRSDHTAAVHVERYLLIFGGGSHATCYNDLHVLDLQTMEWSRPMQLGEIPTPRAGHAGVTVGENWFIVGGGDNKSGVSETIVLNMSTLAWSVVTSVQGRVPVASEGLSLVVSSYDGEDVLVSFGGYNGRYNNEVYVLKPSHKSTLQSKIIENSIPDSVSAIPNVTNVESEFEAGHDSNPPKSKGDIVSVLKAEKEELESSLIKEKHHALQLKQDLAEAESCNSDLYKELQSVRGQLASEQSRCFKLEVEVAELGQKLQTIGTLQKELELLQRQKAASEQAALNAKQRQSSGGVWGWLAGTPPPIQNADDA

>Glyma03g39640|Glycine_max_Kelch

MAMARATSGLQYPERFYAAASYVGFDGSSPTKTLTSKFAKSTALLLYSLYQQASVGPCNIPEPSTWKLVEHSKWASWNQLGNMSSTEAMRLFVKILEEEDPGWYSRASNSVVEPVIDVQMNQNSKVEPVIENGNSYPETKTISTQNGSEVGTQDKDIVVEGFGSVEVYDQWIAPPVSGGNPKARYEHGAAVVQDKLYIYGGNHNGRYLNDLHVLDLRSWTWSKIEAKTGVESPTTSIPCAGHSLIPWGNKLLSIAGHTKDPSESIQVKEFDLETAAWSTLKIFGKAPVSRGGQSVNLVGKTLVIFGGQDAKRTLLNDLHILDLETMTWDEIDAVGVPPSPRSDHTAAVHVDRYLLIFGGGSHATCYNDLHVLDLQTMEWSRPTQLGEIPSPRAGHAGVTVGENWFIVGGGDNKSGVSETVVLNMSTLTWSVVTSVQGRVPVASEGLSLVVSSYDGEDILVSFGGYNGRYNNEVYVLKPSHKSTLQSKIIENSIPDSVSAIPNVTNVESEFEAGHDADPPVCIIDADPPKSKGDLVSVLKAEKEELESSLSKEKQHALQLKQDLVEAESRNSDLYKELQSVRGQLASEQSRCFKLEVEVAELGQKLQTIGTLQKELELLQRQKAASEQAALNAKQRQSSGGVWGWLAGTPPPSRNADDA

>AT4G24230|Arabidopsis_thaliana_C-term_ACB_N-term_TMD

MEVFLEMLLTAVVALLFSFLLAKLVSVATVENDLSSDQPLKPEIGVGVTEDVRFGMKMDARVLESQRNFQVVDENVELVDRFLSEEADRVYEVDEAVTGNAKICGDREAESSAAASSENYVIAEEVILVRGQDEQSDSAEAESISSVSPENVVAEEIKSQGQEEVTELGRSGCVENEESGGDVLVAESEEVRVEKSSNMVEESDAEAENEEKTELTIEEDDDWEGIERSELEKAFAAAVNLLEESGKAEEIGAEAKMELFGLHKIATEGSCREAQPMAVMISARAKWNAWQKLGNMSQEEAMEQYLALVSKEIPGLTKAGHTVGKMSEMETSVGLPPNSGSLEDPTNLVTTGVDESSKNVSGER

>AT4G27780|Arabidopsis_thaliana_Ankyrin_N-term_TMD

MGDWAQLAQSVILGLIFSYLLAKLISIVVTFKEDNLSLTRHPEESQLEIKPEGVDSRRLDSSCGGFGGEADSLVAEQGSSRSDSVAGDDSEEDDDWEGVESTELDEAFSAATLFVTTAAADRLSQKVPSDVQQQLYGLYKIATEGPCTAPQPSALKMTARAKWQAWQKLGAMPPEEAMEKYIEIVTQLYPTWLDGGVKAGSRGGDDAASNSRGTMGPVFSSLVYDEESENELKIDAIHGFAREGEVENLLKSIESGIPVNARDSEGRTPLHWAIDRGHLNIAKVLVDKNADVNAKDNEGQTPLHYAVVCDREAIAEFLVKQNANTAAKDEDGNSPLDLCESDWPWIRDSAKQAD

>AT1G31812|Arabidopsis_thaliana

MGLKEEFEEHAEKVNTLTELPSNEDLLILYGLYKQAKFGPVDTSRPGMFSMKERAKWDAWKAVEGKSSEEAMNDYITKVKQLLEVAASKAST

>AT3G05420|Arabidopsis_thaliana_Kelch

MAMPRATSGPAYPERFYAAASYVGLDGSDSSAKNVISKFPDDTALLLYALYQQATVGPCNTPKPSAWRPVEQSKWKSWQGLGTMPSIEAMRLFVKILEEDDPGWYSRASNDIPDPVVDVQINQRAKDEPVVENGSTFSETKTISTENGRLAETQDKDVVSEDSNTVSVYNQWTAPQTSGQRPKARYEHGAAVIQDKMYIYGGNHNGRYLGDLHVLDLKSWTWSRVETKVATESQETSTPTLLAPCAGHSLIAWDNKLLSIGGHTKDPSESMQVKVFDPHTITWSMLKTYGKPPVSRGGQSVTMVGKTLVIFGGQDAKRSLLNDLHILDLDTMTWDEIDAVGVSPSPRSDHAAAVHAERFLLIFGGGSHATCFDDLHVLDLQTMEWSRPAQQGDAPTPRAGHAGVTIGENWFIVGGGDNKSGASESVVLNMSTLAWSVVASVQGRVPLASEGLSLVVSSYNGEDVLVAFGGYNGRYNNEINLLKPSHKSTLQTKTLEAPLPGSLSAVNNATTRDIESEVEVSQEGRVREIVMDNVNPGSKVEGNSERIIATIKSEKEELEASLNKERMQTLQLRQELGEAELRNTDLYKELQSVRGQLAAEQSRCFKLEVDVAELRQKLQTLETLQKELELLQRQKAASEQAAMNAKRQGSGGVWGWLAGSPQEKDDDSP

>PGSC0003DMG400021512|Solanum_tuberosum_long-N-term_TMD

MNLFQEFFVTAMVTLIFSCIIVKFVSAAVSKDGRVDKEKIVVEEVKITKGLVVRRRKSKKRVKFVEEGVVGNVDRVEREIPNKDGVEGFVEKNIEGVLRKGEDFKGEKGFGNLDLVEHCGGEREVEIEGVLKKGDDFKGEKVCESLELIEQCGGEREVSDGHDDADVCGKTGLVEEKDEVKKIEGVLKKGEDFTGEKVYENLDLTEHCGGEREMTDGHDDADMCGKIGLMEEKDEVKKIDELLVVKEKDTANAVVVDEVKVGENEVVEVGGGLSKGDDEKTDKMVMDEDSDDDWEGIERSELEEEFVKAVNFVDGGNGKNSSMENLGSELMMQLYGLQKIAMEGPCHEPQPMALKVYARAKWNAWQKMGSMNQEVAMEQYIKLLSDHVPEWTHHSKDDCEVGSSETKMHGDPDPISDSSNDTGKDERTHEMNRAAEGDEGEKE

>PGSC0003DMG400030867|Solanum_tuberosum

MALKEEFEAHAEKAKTLPESTTNENKLILYGLYKQATVGNVNTSRPGIFNMRDRAKWDAWKAVEGKSTDEAMNDYITKVKQLLEEGAASA

>PGSC0003DMG400018524|Solanum_tuberosum_Ankyrin_N-term_TMD

MADWQQYLQSIIFGLIFSFLLAKLFSIIFAFRDENLRITRADSSEAEEKRSPEEADIIGVSEDTEPLIQRNDGGLKGSSVSGDEDSDDDWEGVESTELDEAFSAATAFVAATAADRSHKVSNEVQLQLYGLYKIATEGPCTAPQPSALKMTARAKWQAWQKLGAMPPEEAMEKYLDIVTELFPSWLDGSANKKKGEESSGVHNADTRRPMGPVFSTFISEEDSENELKLDEIHAFAREGDDENLLKCIESGIPVDVKDSEGRAPLHWAVDRGHLNITKLLLSRNADVNAKDLEGQTALHYAAVCERGDIAEFLVKHGADVEIKDNEGDCPRDVCELHWPWLQQTTVHN

>PGSC0003DMG400024701|Solanum_tuberosum_Kelch

MAMARVSSGLAYPERFYAAASYAGFDGSSDSSTKGVSSKFSNDAALLLYALYQQATVGPCKIPKPRSWSPVEQSKWTSWNGLGNMASTEAMRLFVKILEEEDPGWYSRASNFVSEPAVQGEKNNETIAETVTENGNVLPEIKTIPAENGILSEPQDKDVVSEGSGAVGVYDQWVSPPISGPQPKSRYEHGAAVIDDKMYIFGGNHNGRYLSDLQALDLRSWTWSKVEVKTSGEAPVAPIAGHSLIPWGGNKLISIGGHTKDPSETMQVKVFDMQTHTWSNLKTYGKPPLSRGGHSVTPTGTSLVIFGGQDANRSLLNDLHILDLETMTWDEMDTL

>20979|Selaginella_moellendorffii_kelch

LPYPAKFHVAAAYAGFSADDDDYENVALPLGSLFTPEMALLLYGLYMQATVGPCTAAKPRMWNTQEHFKWKSWSEFKELPSIEAMRLFVRTLEEQDPDWYFKSQGREPVQDNDLDPSLQEEDIPAVNNSPPSPPLPSANGGIGTEVKMEENDVECSTIMEIDVRNEWLSPLVSGRRPAARYQHAAAVVHDKMFVIGGNHNGRYLNDVQVLDLRTLTWSKVEQKLPTSPLSSSMPPIPSNQILSPCAGHSLIRKNRMLFVVGGHSKNSPDSVSVHAFDTETFTWSLFPTYGQAPIARRGQSVSLIGSNLVMFGGEDSKRRLLNDLNIFDLETMTWEAVDAIGPPPSPRADHAAAVYAGHYLYIFGGGSHSSCFSDLHVLNLKTMEWSRKETEYTPTPRAGHAGATVGDLWYIVGGGDNKSGISETIVLNMKTLDWSLVTSVPARTYIACEGLSLVSACLNGEETLIAFGGYNGKYSNEVCCFFLFLRLSIISKEPENGGYNTPKLLELKRELEVALASCQDENNKLKSELSITANDLSMVFPRFVHETLVLQFLFTVQELQSVRGQFVAEQTRCFKLEVEVAELRQKLQSMEALQKEVDLLRRQKAASDQVAAQLVQQKQNASGGMWSWLAGTP

>92587|Selaginella_moellendorffii_Ankyrin_N-term-TMD

MARQADLQELAQTVFVAIVFAFMLSKLLSMFMAIRSSMRAGNMRVERLSPSEEAQERKEERGASSSSDEKEESIHSDDDGSSDWEGVESTELDEAFGAASSFIATQAGGLKVSSETQLQLYGLYKVATEGPCHTVQPSALKMTARAKWNAWQKLGNISQEEAMERYVELVSELSPDWASNQKSKGKEGTSGSKQKKGPVGPVFSTLAYDERSDDGSLDALHIAAREGNEPGLLELLSQGVPVDNFPDSDGRTALHWASDRGHLGLVELLLSRGAKLQAKDKEGQTALHYACVCEQEMVAKFLISKGADLQARDAEGSTPLNYRPANWDWMNE

>114584|Selaginella_moellendorffii_Ankyrin_N-term-TMD

MARQADLQELAQTVFVAIVFAFMLSKLLSMFMAIRSSMRAGNMRAITGHQSLATMEAGYEPSTSSSVEAEAGSNRSISAMAMDEDDDGSSDWEGVESTELDEAFGAASSFIATQAGGLKVSSETQLQLYGLYKVATEGPCHTVQPSALKMTARAKWNAWQKLGNISQEEAMERYVELVSELSPDWASNQKVRTSGSKQKKGPVGPVFSTLAYDERSDDGSLDALHIAAREGNEPGLLELLSQGVPVDNFPDSDGRTALHWASDRGHLGLVELLLSRGAKLQAKDKEGQTALHYACVCEQEMVAKFLISKGADLQARDAEGSTPLNYRPANWDWMNE

>270651|Selaginella_moellendorffii_ACBP

MATTEEQFKKAAEDALKLPPTTTDADKLILYGLYKQATVGNNETSRPGMLDFKGKAKWDAWKKCEGKSADDAMKDYIAKVEQLMEATA

>Pp1s22_375V6|Physcomitrella_patens_Long_N-terminus_mit_TMD

MGDWQEKMQTIIIGFVFTCMVVMLFSVISSFRAGNLRVARETDDEVPLLVPSEAAELSPAHEQNTPLVATDEETEADGDSELEDSAHDFSSLSSPDSEVETDATAACSPREIDSAINPTVSKVESKDNKVDATKDELAEFEKDEGAEDENIETKHVSISGEHSLKSLNEQANGDVEDELEVSRSATDDWEAIESTELEKRFGAASTYASTMVVSPNVKSSSEALLQLYAFYKIATEGPCSNPQPSILQPTARAKWNEWQKLGSMPQEEAMQKYIAVLTEVNSTCYETYQKSKEPARVVDQNDRDSSVRW

>Pp1s39_429V6|Physcomitrella_patens_Long_N-terminus_mit_TMD

MAEWQEKVQSMILGFVFAFMVMKLISVISSFRSANLRVTRAADEEGLVAGLGSVDEQNAPLVGTDEEVEADGGDEKEDHVDDSSSSESGTEDDAAPTSRPREVCTTDTAAVSEAESKDEEVKPKDDEEVEEIITPGAQSLKALSEQVADDVGAELEEENETDDWEGVESTDLERLFGAASTYADNMVAMFGVKPSTDAMLQLQALYKIATEGPCSTSQPSAFQPSARAKWNAWQQLGSMSQEEAMEKYISVLTEINPAWYESYQKTRETVKGSGEQTTTTPKGEHAD

>Pp1s7_333V6|Physcomitrella_patens

MGLDEDFQAAAAAAKELKTKPSDDDLLILYALYKVATVGKVDTSCPGMFDFKGKAKWNAWKKAEDKSPEDAKRDYILKVQQLQEA

>Pp1s36_294V6|Physcomitrella_patens

MGLDEDFEQAAKDAKALTAMPSNDDLLILYGLFKVATVGKNNTVRPGMLDLKGKAKWDAWKKVEDKSPEDAKRDYILKVQQLQEA

>Pp1s48_92V6|Physcomitrella_patens_Kelch

MDTAVMAQLPYPDRFYAAASFAGFSSSSTSSSSSSPASASGRSVSTKLLPENLPNETQLLLFALYKQATLGPCTLKKPWGWQVVEHAKWTSWNHLGNMNSLEAMRLFVRSVEEIDPNWCAKEQSQSVQVLEDTAAVESASKELTKLLLEKDMPSLPKVMNPIVTNGKHDQVEDLSFSEKVDFNQSETPRMPEMERPVAEGIDAITTYLEWVPVIVTGRKPLARYQHAAAVVEGKLYVIGGNHNGRYLNDVQVLDLKKLSWSKVDTKVPESPLSSHRDLQPWFPQCAGHRLIRWGELLLVVGGHAKPGADTVTVHAFDTHSLSWTKLEVYGQAPVSRGGHSVTLIGSQLYMFGGEDPKRRLLNDLNILDLETMTWEAVTASGACPSPRADHVATAYRDKCIFVFGGGSHSDCYNDLHALDLETMEWASVPTKGISPRPRAGHAGATHGDNWFIVGGGDNTGAISETLVLDMITQSWSIQGVIQGNSAVASEGLSVEVSGNALLAFGGYNGYFNHEVHAYVLESPQSQETGNRMLPADAGVEIKQNTTSLGDRDSSSDSGEIVEPSALDEESDATTMEANSVVEEDSPATSRRGTPRNDEFEQHFQMTSGSRNDGLEQLRMAARAAQAEVEKLKVENAAALSSLADVEQELLSVRSQLQGEQSRSFRLEVEIAELKQKLSSMDALQKELDLLQRQIPTSHKVASEAAQKESKSGVWGWLAGAPPAPRYD

>Pp1s326_16V6|Physcomitrella_patens_Ankyrin_N-term_TMD

MADWQWQEALQSIIIGIVFAYMCGRLFTLISSIRDQKFRLERGGQDTATPLLSTTFDEQEYAPAAELNVPYAPIEEELDADDGGDEQEVQGYDSSSSSDYEDHRSRDIHVTDAAGQSKVAFENDENKPKDTIDVKAPDLKSDDRVAVSSSDPLTVSEEREDAAKESEEGNETDDWEGVESTELEELFGAASTYVASLVSIPGVKPSSEAMLQLYAHYKIATEGPCSTSQPSAFQPSARAKWNAWQKLGNMPQEEAMQKYVGILTAIDPTWHQGQRKNKDPLVESTEELGRNSSQASNKKMGPGPVFSSLVGNEDGGEEASTMDPLHAYARDGDIESIAKLLDQGSNINVKDSDGRTPLIWAVDRGNLRAVEILVAKGAEIYAKDLEGQTALHYATVCELEEVAKYLFEHGADMNLPDNEGNTPLSQCPGHWQWVQRAAG

>Pp1s81_188V6|Physcomitrella_patens_Ankyrin_N-term_TMD

MADWEWQDAVQSLIIGIVFAYMCGRLFTLISSIRDQKFRLERGGENAAAPLFTTTFDEQEYAPAAELNVPYAPIEEELDAFDGGDEQVAHGYDSSSSSSDSDYGIERSHYLLATDSPSYSKITSETDGDQAKNIIEDTTSAQKLDDRVDVSDKDSSKVLADEEREDVAEEIEEGNVTDDWEGVESTELEELFGAASTYVASVINVPGAKSSREEMLQLYAYYKIATEGPCSTPQPSAFQPTARAKWIAWQKLGNMSQEEAMQKYVAVLTAIDPTWHQSHQKGKDSSVEATEELEGAFLHSSSKNMNPGPAFSSLAATDDVEGTSTMDALHTCARDGDLQGLSKLLEQGRSIDVKDSDGLTPLIWAVDRGNVSAMEVLVAKGAEIDTKDVEGQTALHHAILSNQEEVAKYLFEHGANINIADKDGNTPLSQCPAHWQWLQRTTG

>Pp1s68_32V6|Physcomitrella_patens_Kelch

MDTLPMAQLPYPDRFHAAAEFAGFSSSSSPSSPSSTSSRSLSTQRLSEVLPDETRLILFALYKQATLGPCTVKKPWGWNVVEHVKWTSWNHLGNMNSLEAMRLFVRTVEGIDPNWYAKERTLNLRALEDSAAVESASKELTKLLLEKNVPTLSKASDSVVSKEKHVQDEVMPLLEENNELNESEIPKLPEMDEPVAEGIDAITSYREWVPVNVTGRKPLARYQHSAAVVEGKLYVIGGNHNGRYLNDVQVLDLKTLTWSKVDTRVPQSPFSSKQDPLQPWLPQCAGHRLIRWKELLLVVGGHAKPAAETVTVHSFDIHSLSWTKLSVYGQAPVSRGGHSVTLIGTQLYMFGGEDLKRRLFNDLNILDLETMTWKSVIASGACPSPRADHVATAYRDSCIFVFGGGSHSDCYNDLHALDLETMEWALVPTKGIVPRPRAGHAGATHGDNWYVVGGGDNTGAISETLVLNMATLSWSVEDVVQVTSATASEGLSVEVAENALLAFGGYNGYFSNEVHAYILGPALLPEIVNQVPPVEATEQNTTSLNEEGSSSDSEEIIESSALDEESDAKVVSKEDSPSTSRREAPPNDEIEQRFRNDELEQLRMAVGAAQAEVEKLKVENAAALSSLADVEQELLLVRSQLQGEQSRSFRLEVEIAELRQKLLSMEALQKELDLLHRQMAVSERAANEASQKDSKVGVWGWLAGAPPASRYD

>Vocar20009951m.g|Volvox_carteri_Kelch

MTTQQCPLAYPDKFHAAAKFWKLRLAGAKTLSQENQLLLYALNQQATVGPNTTARPWGWNVVESTKWQGWKELGNMTTMEAMRLFVRTLDDEQPDWWVLLEQNEAKADSEGPVAAAAAPAPTRTVAQRALLGLWTHLEAATEAGKRPMPRYESAAAVLGGNMYVLGGNYGGRYLSDLWALDLAAGTWSPLQLQPAAGTGADPAAAAAAFPPTAGHTVTVWNGKLYVLGGHTKAKGDAAMVLRVLDPAASTVAEPVTSGQAPSARGGHTATLLGNKLWVIGGEDSARRALSDVHVLDLDTLSWSTPEISGKAPLGRSASCATAHQDRYIVIFGGGSVATCFSDLHMLDTHELTWTQLAQAGAKVTPRAGHAGAVLGGIWYIVGGGNNVKGCTDLLAADLSGLPASGTVTWHVVTSVALRDPLSSEGISLVVLPSDRVLVAFGGYNGKYQNTVNLFRAPEGSAALLAGTLAAAAEAVEEAKAKLANGGAPDGSAAAEVPAAEAQATKKTAAEPAAAATPPPLPADPLAELRLQVSELRAQLEGARSEAETAIRESAAAKESAAHELALLRKQLTAAQASLAESNKALEDTRSNLSAEQSKVLKLEAQVAELQAKLGQLGELEREMEKYRRAAREAAEKEAASKKGGGFWGYIAGSSGSS

>Vocar20003619m.g|Volvox_carteri

TSRRNPCPDTVKQETMGLKEDFEAAAAEATNSLPDTLSNDEKLELYALFKQAKEGDCNTSQPGIFDPKGRAKWNAWNGKKGTSQEDAMKQYIEYVAALKAKHGTK

>Vocar20000983m.g|Volvox_carteri_C-term_ACB no TMD

MVPSAAVSGVIAAAASTAAAAPKRDPDSAVALLHAAGDDQEALAEAIAEAAFLDTTPGDHRQKLRAARARLRQLNAAAAKADSADRSPHAKAEYTAEDFERLTGQYEKLNWRMVSKPGGATVKPDDFYRLYALHMQATQGDNATERPMWAERGGLDFEGRARWDAWSALRGTDPAKAQLRFVKLFHEFSPAALYKDTRGAVLAAGGQ

>XP_016568851_ACBP-L2_Capsicum_annuum_C-term_ACB_N-term_TMD

MIFFQEFFVIAMVALVFSCIIVKIVSALVSNDGRIDKERIVVEETKITKGLVVSSRNSKKRVKFVEQEVVGNVDHVVTQRANEDGVEKKIEGILKKGDDFKGEKVIENLGVIKHCGGEKEVSDDHDDVGFLGKIGLMEDIDVKKIEGILKKGDDFKVEKVTENLEVIEHCGDEREVSDVHDDADMCRKIEFVEKKDEVKKIEGVLKKGDDFKVEKVHENLGVIEQCGGEREVSDGHCDADICSTVGLLEEKDEIKKIEEKIEEIIKKGDDFKGEKVRENFDLIEHYGGEREVSDVHGDADMCSEIGLMEEKDEVKKIEKKIEDILKKGDEFKGKKVCENLDLIEHYGGEREVSDVHDDADMCRQIGLMEEKDVVKKIDELLVVKEKDIVNRMIVDEVKLGENEVVEVGGGLSKGDDDKTDMIAMDEYSDDDWEGIERSELEEEFVKAVNFVDGGNGKNSSMENLGSELMMQLYGLQKIAMEGPCHEPQPMALKVYARAKWNAWQKMGSMNPEVAMEQYIKLLSDHVPKWTHHSKDDCEVGSSETKMTRDPDSLNDTHKDERTQEMNHAAEGGDFSGGGKE

>XP_016566889_ACBP1_Capsicum_annuum_Ankyrin_N-term_TMD

MADWQQYLQSVIFGLLFSFLLAKLFSVIFAFRDENLRITRADYEDTEPKRSPEEEEDATAAAAQPLIERNDDRGGGGDLKGSEVSGGAEDSDDDWEGVESTELDEAFSAATAFVAATAADRSHKVSNEVQLQLYGLYKIATEGPCTAPQPSAIKMTARAKWQAWQKLGAMPPEEAMEKYLDIVTELYPSWLDGSAIKKKDEESSVARNADSRGPMGPVFSTFISEEQSENELKLDEIHAFAREGDEENLLKCIESGVPVNVKDSEGRAPLHWAVDRGHLNITKLILRVFNLFPISVGASVSCLCKYTELQILVILILKDLEGQTALHYAAVCEREDIAEFLVKHGADVEIKDNEGDCPRDVCELHWPWLQQATADN

>XP_016538619_ACBP4_Capsicum_annuum_Kelch_repeat

MAMARVSSGLAYPERFYAAASYVGFDGSSDSPAKGVTSKFSNDAALLLYALYQQATVGPCKIPKPRSWSPVEQSKWTSWNGLGNMASTEAMRLFVKILEEEDPGWYSRASNFVSEPAVLGEKNNVMIAEPVTENGNVLPEIKTIPGENGNLSEPQDKDVVSEGVGAVGVYDQWVSPPISGPRPKARYEHGAAVIDDKMYIFGGNHNGRYLSDLQALDLRSWTWSKVEVKTSGEASQVPVAPFAGHSLIPWQGNKLISIGGHTKDPSETMQVRVFDLQTLTWSNLKTYGKPPLSRGGHSVTPAGTSLVIFGGQDANRSLLNDLHILDLETMTWDEMDTLGVPPSPRSDHAAAVHAERYLLLFGGGSHATCFNDLHVLDLQTMEWSTPTQQGEIPSPRAGHAGVTVGENWFITGGGNNKSGVSETVVLNMSTLGWSIVTTVQGRVPLASEGLSLVLCSYNGEDILVSFGGYNGRYSNEINVLKPSHKSTLQSMETPVPDSVSAMQNATNATRDLESDIATVQEGKIREIVMDNIESEPMVNKVEETTERLIAALKAEKEELESSLSKEKLQTLQLKQDLTDAEARNTDLYKELQSVRGQLASEQSRCFKLEVDVAELRQKLQALESLKKELELLQRQKAASEQALNEKRRQSSGGVWGWIAGTPPNQQDDA

>XP_016542010_ACBP5_Capsicum_annuum_Kelch_without_ACBD

MRSFSFQVLNFDSFSWTTASSKLYLSPTSLPLKIPACKGHALVQWGKKILLVGGKTDPASDKVAVWAFDTETECWSLLETKGDVPVARSGHTVLRASSTLILFGGEDAKRRKLNDLHMFDLKSLTWLPLHCTGTGPSPRSNHVSALYDDKLLLIFGGSSKSKTLNDLYMLDFETMAWSRIRIRGFHPSPRAGCCGVLCGTKWYIAGGGSRKRRHAETLIFDVLKLEWSVAVASPASSITINKGFSLVLVQHKERDFLVAFGGFKKDPSNEVEVLIMEKNELPMGRRSSLSKATGNLLSENLLTSTGPASQPINGTTTSHVDSIARQNLASAVEHHGSGRKSLSESLLIDPSSVSGNVSLRKQFSNDEDACAKMTKTSGDGSPSQEHGAKQLDIGIKMSSSGGKTMAEEMSTISESGHLPTHYRQASANFFQDADDFVFQEGDSKTGLPASSGASQQYEAKLSSLMRKNGILEGQLAAALAGREAAEKNLSSALKSKQDMEKKMADAVKEMELLKGKLASVELAQEEANSLSNIVHSDNVRLEHDVAFLKAVMDDTQKELHTTRGVLAGERARAFQLQVEVFHLKQRLQSLENRSPTPRKPFHV

>XP_016557647_ACBP-L1_Capsicum_annuum

MALKEEFEEHAEKAKTLPESTTNENKLILYGLYKQATVGNVNTSRPGIFNMRDRAKWDAWKAVEGKSTDEAMNDYISKVKQLLEEAAASA

>XP_016572376_ACBP-L3_Capsicum_annuum

MPLKEEFEEHADKAKTLPESTTNENKLILYGLYKQATVGNVNTSRPGIFNMRDRAKWDAWKAVEGKPTDEAMNDYITKVKQLLEEAVASA

>Zosma48g00820_Zostera_marina_Kelch

MTPDLGMEREMGKSSSGISYPGRFFTAASSVGLGESAIGSISRFKNDVALMLYGLYQQATVGPCNAAKPRIWNTVELGKWTSWSGLGNMPSTEAMRLFVKLLEEEDPSWYSRVHQDNVDQSLGVEINKEEQEAKTPSENYPETKTISENGSLLETQDKDIILEGVGSVSIYDQWVPSSISGQCPKPRYQHGAAVLHDKMYIFGGNHNGRYLNDLQVLDLKSLTWSKVDAKTIGSEDPSKKTTMAPCAGHSLISWANKIISIAGHTKDLSEAIQVKEFDPQSCTWSIMKTYGKAPISRGGQSITLVGSNLVIFGGEDAKRTLLNDLHILDLESMTWDEIDAVGVSPSPRSDHSAAVHAERYLLIFGGGSHSTCFNDLHVLDLQSMEWSRPAQQGVVPSPRAGHAGITVGENWFIVGGGDNKSGASETLVLNMSTLVWSVVTSIQGRVPIASEGLSLVVSSCTGEDILVSFGGYNGRYNNEVYVLKPSHKSDVPSKMLENGASDSVAALVPETNETKDMESEVYHPDQGGRIREIAIYNTDLMPRNVGKNENNARIMEAFRVEKEELEVALSKERLESLRLKQELSDGDTRKADLTKELQSVRGQLASEQSRCFKLEVDVAELRQKLHTMEALQKEVELLQRQKAAAEQDNLSAAKQRQSSGGVWGWLAGSPPDEN

>Zosma261g00210_Zostera_marina_kelch

MAKPSSGVPYPERFYVAAEYAGLGDRAKLGNFSRFENDVALMLYGLHRQATVGPCNAAKPGLWNVVEHGKWTSWSELGAMASTEAMRLFVKLLEEEEPNWYSKASEFLLDPIHELEINKTKLDTDTQSENYPETKTIAENGCILETRDKDIILEGVGSVGTYNQWVALSISGEYPKPRYQHGAAVLQDKMYVFGGNFNGRHLNDFQVLDLKSLTWSKVENTTVDSQDASTITTLSSCAGHSMVSWENKIISIAGYTKDPSEIIHVNEFNKQTCAWSSMKTYGKAPISRGGQSATLVGTTLVIFGGEGAKKTLLNDLHILDLESMTWDEIDTIGVPPSPRSDHSSAIYNERYLFIFGGGSHSTCFNDLHILDMNNMKWSKPTQKGTIPSPRAGHAGITIGVNWFIVGGGDNKSGASETYVLNMSTLVWSIVTTVQERIPVASEGLSLVLSSCTGEDILVSYGGYNGRYSKEVYVLKPRCKSDVLSSKTIENNAPEDTQVLLPTSTLLNDVEIEIESSLQSGRAKEIIMDNIDSEHLDIRSDETNIKLLEDLRSEKGVLEAELAKEKSKSLHLKQKLDDAEAQKIILTQELQSVLQT

>Zosma26g01070_Zostera_marina_Kelch_no ACB domain

MSNIGGDDGTEIENGKQELGIRSMLSNEVPYDEWTLLHVSGQRPSARYKHAAEVINEKLYVIGGSRYGRYISDIQVLNLVSSTWSGIKLNQNPNNDESNGRLQDMLPAIAGHSLIKWKDVLLVVAGNPKDSIDTVSVWTIDPTTDNCVAVKTSGKIPIARGGQSVTLVGSKLIMFGGEDNRRKVLNDIHILDLETMSWDAIETSKNTPGPRFDHTASSYEDRYLLIFGGSSHSACFSDLHILDLYTMDWSKAQVEGHVINPRAGHACIVVDGIMYIVGGGDNKSGAFETIKLNMSKLIWSVTTKVSQRHPLASEGLSLCLAEIDGVNFFIAFGGYNGKYHNEVFVLKPKPSGPLAPPRRILQSSAAAAAAASVTVAYALASVENEKVSSTQVTDKNANEIKSEKSSHNNIEFTMEKKLDQSILDDIRNANHRLVDELKELCNSHGELSMELESVQAQLESERSRCSKLELQVEDIKKKLESQHLIEAELEILRHGNSSSHDHPDAKMQKSGVWNWMSGAV

>Zosma277g00110_Zostera_marina_Ankyrin_N-term_TMD

MVGNWQELIQTVVIGLIFSYLVAKLISTVVNFREENLKVVREDDVLAEEESSSAPSADADSLKIETPGVSEGKILEDVSEDDYEDRGKKEEEEDEEDDWEGVESTELDEVFSAASVFIAATVSDRSNKVSNDVQLQLYGLYKIATEGPCTVPQPSAFKMTARAKWNAWHGMGAMPTEEAMEKYVEIVIRLYPDWGSPSASKTSESGNASKTPMGPVFSSFVHEEASDDELKLDVVHVSAREGKAEDLIKHLENGVSVNLRDSDERTALHWAVDRGHLHLVDILISNGADINAKDNEGQTPLHYAALCEREAVAEFLVEHRADVNMKDNDGSTPADLIASSWNFMTCVH

>Zosma123g00500_Zostera_marina

MGLQEEFEEYAVKAKTLPENTTNESKLILYGLYKQATVGPVDTSRPGIFNQRDRAKWDAWKAVEEKSKDEAMGDYIIKVKQLLEESA

>Zosma74g01040_Zostera_marina

MELQEDFEEHAKKIMTLTEEPSNDDKLILYGLFKQASFGPVTTDRPGIFKLKERAKWDAWKAVEGKSKEDAMNDYIIKVKQMMEC

>Zosma32g00460_Zostera_marina_C-term_ACB_N-term_TMD

MMMELLWVLDELLMTSLFLFIVSVFIDRIFPTVLVDEIFLGVADAIAGLRSGDRIVVVGSDEEEENQEGGVGDIDFGKLVEEEEEDEAVDMVSEGEIGMDEAVGEIRLEDKEVKLSEHDDTGVDDDDDWEGIEQTPTGELFAIANDYFGSTVGKEELSGLGPDVQLQVYALSKVAIDGPCYGPQPLSLKPSSRARWHAWRNLGDMSPEQAMDEYLTLLCANISGWGESRSRIPANCSATSCNEQSN

>Sphfalx0016s0055_Sphagnum_fallax

MGLKEDFDQAAKDALTLPESTTNEDKLILYGLFKVATVGKPETSRPGIFDPKGRAKWDAWKKVEDKSKDEAMQEYIVKVTQLKEA

>Sphfalx0118s0024_Sphagnum_fallax_C-term_ACB_N-term_TMD

MSRKLVESLHGICFFYPPSGKLSTAFLRSFFACCLHRSVVFLVVSLLQGWWRREGKGDDKNDVLVKKKKKVQEKPCQKKTMGLKEDFDQAAKDALTLPASTTNDDKLVLYGLFKTATVGKPETSRPGIFDQKGRAKWDAWKKVEDKSKDEAMQDYILKVTQLKEA

>Sphfalx0295s0007_Sphagnum_fallax

MGLKKDFDQAAKDALTLPPSTTNDDKLVLYGLFKSATVGKPETSRPGIFDPKGRVKWDAWKMVEEKSKDEAMQDYILKVTQLKEA

>Sphfalx0240s0015_Sphagnum_fallax_Ankyrin+N-termTMD

MATADWQEAVQASFLGLMFAFMVAKLVSLVFSFWGKNLRVECAPVTAAARSFTTVDDGELTPADEFNAPLASWPAGEESAAAEEEEEEGHDKQHDRDFDDASSGEDTIETDHHHHLHSSFSDSATDTPVPAARVEEAVFRPDESGQLNEEGKVATDVDFKEDLKPLKLFAIDTVATVSEEHPLQALTEGQKEERIKKGIEEEEENETDDWEGVESSELEEQFGAASTYVASMVTLPGVKPSSEAMLQLYAYYKIATEGPCSTSQPSILQPTARAKWNSWQKLGNLPQEEAMQCYIDVLTEVAPSWNQNFEKSKEPTKQVLEETEGGNTSSKIGMGPVFSSLAMSEEGVGEEGTLEAIHACAREGDLQGLSQLLDLGIPVDLKESHGRTPLIWAADRGQLSAVEILLAKGAEINAQDIEGQTALHYATVCEQEAVAKYLAEHGADAKIADKEGTTPLKLCPPHWAWMQGTPT

>Sphfalx0067s0109_Sphagnum_fallax_Ankyrin+N-termTMD

MAMTDWQEVVQASILGLIFAFMVAKLVSVVFSFRGENLRVERDPAAPSFTINRELAPADELTAPLASLREEEESEFSGEEFKHSRGLGFGVSGDDEIEKHDDTLSSSSSDSETDSPRQALVVEAVSFPGSESHEEEEKAMNAVSDEDLDLEKPKAVDTATIVSDEHALQALTEEGRREETIEKGSEEGNETDDWEGVESSELEEHFGAASTYVASMVTLPGVKPSSDAMLKLYAYYKIATEGPCSTSQPSVLQPTARAKWNAWQKLGNLPPEEAMQCYIAVLTEISPTWHQNSQKSKKPMEEALEEPRGAIASSKMGTGPVFSSLAMSEEGVGEEGTLEAIHACAREGDLQGLGQLLDHGSPVDVKDSDGRTPLIWAADRGELSAVEILLAKGAEINAQDIEGQTALHYATVCEQEAVAKYLFEHGADVNIADKEGDTPLKLCPPHWVWLQSSPP

>Sphfalx0178s0028_Sphagnum_fallax_Kelch

MMTNVGMAAATLPYPDRFYAGAAYAGFSASSPGNVDSLKSSSLGLSDDTTLLLYGLYKQATVGPCNVPKPWSWNVIDLAKWTSWNQLGRMDSMEAMRLFIRTLEEEDPSWWSKAQEQDKEVVWNDPPPPVVDSEPAVQEETENEKLVVAVELNGEDKVDDEMPVVEAPPASNGLSKLNGVFNTTEVEEVAEGVRAIPVYNEWVLPTVTGRRPSARYQHAAEVVNNKLYVIGGNHNGRYLNDVQFLDLKTLEWSKVEKVPQSPLSEQQKQLSSWFPPCAGHSLIRWGTKLLAVAGHSKEPVDTVTVRAFDTHTMTWTILDVHGKTPIARGGQSVTLVGSTLVMFGGEDSKRRLMNDLNILDLETLTWKAVDTSGTRPSPRSDHVAAAHGDRYLFLFGGGSHSSCYNDLYVLDLDSMEWSQAQTGGTVPSPRAGHAGATIGNSLYIIGGGDNKSGISDTLVLNMDTLVWSVVATVKGRAAVASEGLSVVVVEDSLLAFGGYNGHFNNQVYVFRTFPAEKVQSKILESSAAAAAAVSAATAVAAPSPPAHSSLVNGKSSSSSPDTQETVVEPKEVSETVQDSMAVELPTEESEHVLADPATREKLALALKVNEELQATQEKLALALKENEELQVTREKLALALKVNEELQAATVAAQAECAKLQNELAAYQFSSSELEQELHSVRQQLAAEQSRSFRLEVDVAELRKKLQSMETLQKELDLLRQKAASDEAAVQAVQKQQSTGVWSWLAGNPLDAKGDED

>Sphfalx0069s0049_Sphagnum_fallax

MMAYGGMATALPYPDRFYAGAAYAGLSATSPGNVDSLKSSSLGLSDDTILLLYGLYKQATVGPCNLPKPWSWNVIDLAKWTSWNQLGRMDSMEAMRLFIRTLEEDDPSWWSKAQELDKEVTLDTEISKPPPPVADSEPVAQEEMDNEKLVVEVELNGEINVEDTPTAVVPSESNDLLKVNGIFSTAEVEDVAEGIRAIAVYNEWVSPTVTGRRPSARYQHAAGVVDNKMYIIGGNHIGRYLNDVQVLDLKTLEWSKVDKVPQSPLSLQQRQSPYWFPPCAGHSLVRWGTKLLAVAGHSKEPVDTVIVRAFDTHTMTWTILDVYGKSPIARGGQSVTLVGSTLVMFGGEDSKRHHLMDDLNILDLESLTWEAIETSGTRPSPRADHVAAVHGDRYLFLFGGGSHSNCYNDLYVLDLESMEWSQAQTQGTVPSPHAGHAGATIGNSFYIIGGGDNKSGISDTLVLNMDTLVWSLVASVKGQTAISSEGLSVLVVEDSLVAFGGYNGHFNNQVHVFRTFPPERLQSKILKSPAAAAAAAFAAPQPVLSSPLNGTSSLSPDPEETVSESRAVPESVQENLDGEQLREEPECALDDSATGKKLALVLKVNEELQEATLAAQAECAKLQNELAMAQFNSSELEQELHAVRGQLAAEQSRSFRLEVDVAELREKLQSIEILQKELDLLQHQKAASEEAVIQATQKQSTGVWSWLAGSPPDSNGVED

>Sphfalx0004s0071_Sphagnum_fallax

MSDAREEDFADAEKTWRAACYFLAKGPSATLQFLGDDDRARLYAFQAQALDGPCPPGQDGALAVDPALRRKQEAWRALDEMSRDEAKKNMVDFLSHLLPEWRDWFKKHAEATLNASEDNGEAHKLLRGFKARGNSGTPGIQYSRL
