# Supplementary material for: The diversity of ACBD proteins – From lipid binding to protein modulators and organelle tethers
Source: Biochim Biophys Acta Mol Cell Res. 2020 May;1867(5):118675. doi: 10.1016/j.bbamcr.2020.118675 (PMC7057175; doi:10.1016/j.bbamcr.2020.118675)
Supplement: Supplementary material 2 — Sequences used for Fig. S2. [file mmc7.docx]

>sp|O75521|ECI2_HUMAN

MAMAYLAWRLARRSCPSSLQVTSFPVVQLHMNRTAMRASQKDFENSMNQVKLLKKDPGNEVKLKLYALYKQATEGPCNMPKPGVFDLINKAKWDAWNALGSLPKEAARQNYVDLVSSLSPSLESSSQVEPGTDRKSTGFETLVVTSEDGITKIMFNRPKKKNAINTEMYHEIMRALKAASKDDSIITVLTGNGDYYSSGNDLTNFTDIPPGGVEEKAKNNAVLLREFVGCFIDFPKPLIAVVNGPAVGISVTLLGLFDAVYASDRATFHTPFSHLGQSPEGCSSYTFPKIMSPAKATEMLIFGKKLTAGEACAQGLVTEVFPDSTFQKEVWTRLKAFAKLPPNALRISKEVIRKREREKLHAVNAEECNVLQGRWLSDECTNAVVNFLSRKSKL

>sp|P07108|ACBP_HUMAN

MSQAEFEKAAEEVRHLKTKPSDEEMLFIYGHYKQATVGDINTERPGMLDFTGKAKWDAWNELKGTSKEDAMKAYINKVEELKKKYGI

>sp|Q8N6N7|ACBD7_HUMAN

MALQADFDRAAEDVRKLKARPDDGELKELYGLYKQAIVGDINIACPGMLDLKGKAKWEAWNLKKGLSTEDATSAYISKAKELIEKYGI

>EAW90652_ACBP-L5_Homo_sapiens

MGDAGATAAALRPAHNLRPAPPTASAAHAQSSRTSAPSAQRRLPAEPSHQPSGTRTSLTRPLRTVPCPAPLPGSQRVGLMIPRPRIHPHPLLAPGTASTTPCAKWSSSCAALKQLKGPVSDQEKLLVYGLYKQATQGDCDIPGPPASDVRARAKWEAWSAKKGASKMDAMRGYAAKVEELTKKEVGGVEREQRGVQDGRHEGLRGQSGGADEEGRASKMDAMRGYAARVRR

>XP_005597701_ACBP-L5_Equus_caballus

MCQVEFELACAAIKQLKGPVSDQEKLLVYSFYKQATQGDCNIPAPPATDVKAKAKWDAWNDKKGISKMDAMRIYVAKVEELKKNDTG

>XP_001487927_ACBP_Equus_caballus

MSQAEFDRAAEEVKNLKTKPADDEMLFIYSHYKQATVGDVNTERPGMLDLKGKAKWDAWNALKGTSKEDAMKAYINKVEDLKKKYGI

>XP_003364405_ACBD7_Equus_caballus

MSLQAEFDRVAEDVRKLKRRPDDAELKELYGLYKQSVIGDIDIECPAMLDLKGKAKWEAWNLQKGLSKEDAMSAYVSKARELIEKYGI

>XP_005603601_ECI2_Equus_caballus

MAGAAVRLARRWAASAVRSPVQDARFPALKLHVGGTAMRASQKDLENAVNQMKLLKKDPGNEVKLKLYALYKQATEGPCNMPKPGIYDLIRKAKWEAWNTLGNLPKETARQNYVDLVSSLCSSSESSSQGKPGADREQLRYETLVVTSEDGITKIMLNRPSKKNAVNLQMYQEIVLALKAASQDDSTITVLTGNGDYYSSGNDLTNFTNTSPGEIQEKAKNSLILVREFVGCFIDFPKPLIAVVNGPAVGIAVTLLGLFDIVYASDRATFHTPFSRLGLHPEGCSSYTFPKIMGPTKATEMLIFGKKLTAEEACAQGLVTEVFPDRTFQKEVWTRLKAYAKLPSNVLRISKQVIRNREKEKLHSVNVEENSVLQGTVLSDEAVNAVMNFLSKRAKL

>XP_005505169_ACBP_Columba_livia

MSEAAFQKAAEEVKQLKSQPTDQEMLDVYSHYKQATVGDVNTDRPGMLDFKGKAKWDAWNALKGMSKEDAMKAYIAKVEELKGKYGI

>XP_005505011_ACBD7_Columba_livia

MALQADFDHAAGDVRKLKTTPTDEELKELYGLYKQATIGDINIECPGMLDLKGKAKWEAWNLKKGLSKEDAMKAYISKANALVEKYGI

>XP_005506757_ECI2_Columba_livia

MTAATMQVSQKDFEKAQEQLKLLKNDPGNETKLKLYALFKQATEGPCNSPKPGMLDFVKKAKWDAWNSLGNLSQDDAREKYTELVSSLVSAESAGQHKDASPDENRLGGYETIIVTTKNNITKIMFNRPDKKNAINHKMYREIIKALEEAGKDDSTIAVITGNGDYYSSGNDLSNFTGVKPTEMKKMAEDGAVLLKEFVSHFIDFPKPLIAVVNGPAVGICVTLLGLCDIVYASDRATFHCPFSQLGQSPEGCSSYLFPKIMGLAKANEVLLFNKKLTAADACVQGLVTEVFPDRTFQKEVWARLEAYASLPKNSLTLSKQLIRSIEKEKLHAVSSKECEVLKERWLSDECINAVVTFFQKKSKL

>XP_005230860_ACBP_Falco_peregrinus

MTEAAFQKAAEEVKQLKSQPTDQEMLDVYSHYKQATVGDVNTDRPGMLDFKGKAKWDAWNALKGMSKEDAMKAYIAKVEELKGKYGI

>XP_005239124_ECI2_Falco_peregrinus

MTAATMQVSQKDFEKAQEQLKLLKKDPGNETKLKLYALFKQATEGPCNAPKPGMLDFVKKAKWDAWNSLGNLSQDNARQKHTELVSSLVSAESAGQKKDASPEESGHDGYETILVTTKNNITKIMFNRPDRKNAINHKMYREIIKALEEAGKDDSTIAVITGNGDYYSSGNDLNNFTNIQPGEMEKMAKDGAVLLKEFVGHFIDFPKPLIAVVNGPAIGICVTVLGLCDVVYASDRATFHSPFSQLGQSPEGCSSYLFPKIMGSAKANEILLFNKKLTAAEACAQGLVTEVFPDSTFQKEVWAKLEAYASLPKNSLAVSKQLLRSVEKEKLHAVNSKECEVLKERWLSDECVNAIVSFFQKKSKL

>XP_005228918_ACBD7_Falco_peregrinus

MALQADFDGAAEDVKKLKTRPTDEELKELYGFYKQATVGDINIECPGMLDLKGKAKWEAWNLKKGLSKEDAMNAYISQAKAMVEKYGI

>NP_001103801_ECI2_MOUSE

MAAVTWSRARCWCPSVLQVFRLQVAKLHLGRPTMRASQQDFENALNQVKLLKKDPGNEVKLRLYALYKQATEGPCNMPKPGMLDFVNKAKWDAWNALGSLPKETARQNYVDLVSSLSSSSEAPSQGKRGADEKARESKDILVTSEDGITKITFNRPTKKNAISFQMYRDIILALKNASTDNTVMAVFTGTGDYYCSGNDLTNFTSATGGIEEAASNGAVLLRDFVNSFIDFPKPLVAVVNGPAVGISVTLLGLFDAVFASDRATFHTPFSQLGQSPEACSSYTFPKMMGSAKAAEMLLFGKKLTAREAWAQGLVTEVFPESTFETEVWTRLKTYAKLPPNAMRISKELIRKNEKEKLYAVNAEECTTLQARWLSEECMNAIMSFVSRKPKL

>NP_031856_ACBP_MOUSE

MSQAEFDKAAEEVKRLKTQPTDEEMLFIYSHFKQATVGDVNTDRPGLLDLKGKAKWDSWNKLKGTSKESAMKTYVEKVDELKKKYGI

>NP_067269_ACBP-like5_MOUSE

MSQVEFEMACASLKQLKGPVSDQEKLLVYSFYKQATQGDCNIPVPPATDVRAKAKYEAWMVNKGMSKMDAMRIYIAKVEELKKKEPC

>sp|Q9D258|ACBD7_MOUSE

MSLQADFDQAAQDVRKLKSRPEDEELKELYGLYKQSVIGDINIACPAMLDLKGKAKCEAWNLQKGLSKEDAMCAYISKARELIEKYGI

>XP_003417885_ECI2_Loxodonta_africana

MEYVKAAYRNSQRETDHPETTPRPSPTCCSASSASHQSEPRGTGFGSLPLGFALPTLASSQSAISRQGPAPVSGPAESRMVPCVARFASARQSRANSLHLQLAMAWVVARLAWRCPPRYAGSPLQVSSFLVQQMHMSQTAMRASHKDFETAVNQMKLLKKDPGNEMKLKLYALYKQATEGPCNMPKPGVLDLISKAKWDSWNALGSLPKETARQNYVELVSSLSSSESSSQGKPGADSKQPGYETLVVTSEDGITKIMLNRPTKKNAITTQMYQDIVLALEAAGKDDSTITVLTGNGDYYCSGNDLNNFTDIPPGGIEEQAKNNAILLRNFVGCFIDFPKPLVAAVNGPAVGISVTLLGLFDIVYASDRATFHTPFTHLGQSPEGCSSYTFPKIMGPAKAAEMLIFGKKLTAREACAQGLVTEVFPDSTFQKEVWTRLKAYSKLPPNAMRTSKQLIRNVEREKLHATNSEEISALQGRWRSEECTNAVMNFLSRKVKL

>XP_003416725_ACBP_Loxodonta_africana

MSQAEFDKAAEEVKHLKTKPDDNEMLFIYSRYKQATVGDVNTERPGMLDFKGKAKWDAWNELKGTSKEDAMKAYVDKVEELKKKYGI

>XP_003410775_ACBD7_Loxodonta_africana

MTLCIKQLTPPRKTFGKADFDRAAEDVRKLKARPDDENLKELYGLYKQSVIGDIDIERPGTLDLKGKAKWEAWNLQKGLLKEDAMRAYISKAKELIEKDGI

>XP_003417017_ACBP-L5_Loxodonta africana

MGQVEFELACAAVKELKGPVSDQEKLLVYSFYKQATQGDCNIPAPPSTDVKAKAKWEAWNENKGMTKLDAMRIYIAKVEELKKKEGG

>XP_535873_ECI2_Canis_lupus

MQALSLHMSKTAMGASQKDFESAMNQVKLLKKDPGNEVKLKLYALYKQTTEGPCNTPRPGVFDLINKAKWDAWNALGNLPKETARQNYVDLVSDLSSSDSSSQVKPEADRKQPGYETLVVTSEDSITKIMMNRPAKKNALTIQMYREIMLALEAASKDDSTIIVLTGNGDYYSSGNDLMNFMNIPPGEMEKEAKNGAILLRDFVGCFIDFPKPLVAVINGPAIGISVTILGLFDLVYASDRATFHTPFTHLGQSPEGCSSYTFPKIMGQAKAAEMLMFGKKLTAREACAQGLVTEVFPDSTFQKEVWTRLKAYSKLPRNTLHISKQSIRNLEKEKLHAVNAEENSVLQERWLSDECINAVMSFLSRKAKL

>sp|Q9TQX6_ACBP_Canis_lupus

MSQAEFDKAAEDVKHLKTKPADDEMLYIYSHYKQATVGDINTERPGLLDLRGKAKWDAWNQLKGTSKEDAMKAYVNKVEDLKKKYGI

>XP_850165_ACBP-like_Canis_lupus

MSTVTKALSLWAGPVNMMGYHSQDFIILYGKVPKPHNPASMSQAEFDKATEDVKHLETKPADDEMLFIYSHYKQATIGDVNTEWPGLLDLRGKAKWDAWNHLKRTSKENAMKAYINKVEDLKKKYGM

>XP_848899_ACBD7_Canis_lupus

MSLQADFNKIAEDVRKLKARPDDEELKELYGLYKQSVVGDINIECPGMLDLKGKAKWEAWNLQKGLSKEDAMSAYIPKAKELIEKYGI

>XP_537760_ACBP-L5_Canis_lupus

MCQVEFEMACAAIKQLKGPVSDQEKLLVYSFYKQATQGDCNIPAPPATDVKAKAKWEAWNQNKGMSKMDAMRIYVAKVEELKKKDTG

>XP_003763843_ACBP_Sarcophilus_harrisii

MSQAEFERAAEEVKNLKAKPNDEEMLFIYSHYKQATVGDINTERPGMMDFRGKAKWDSWNSLKGKSKEEAMKAYIAKVEELKKKYGI

>XP_003760261_ECI2_Sarcophilus_harrisii

MPAATLRACSRPPLPGSLWKRQLPAVGFPALQLHMTNMALRASQEDFERAKGQIKLLKEDPGNEVKLKLYALFKQATEGPCTSPKPGMLDFVNKAKWDAWNALGSLSKDTARQNYVDLVASLVSSQSLSQETSIDKKSEYETLVVTREDNITKIMLNRPAKKNAINNKMYNEIMLALEAADKDDSSLTVLTGNGDYYSSGNDLSEAAKVPPDDIEKKIKESFVLLRTFVDHFIDFSKPLVAVVNGPAVGISVTLLGLCDIVYATDRATFHTPFIQLGQSPEACSSYTFPKIMGPIKAAEMLIFGRKLTAQEAYAQGLITEVFPDSTFQREVWTRLKAYAQLPPKAMMFSKQLIRSFEKETLHKVNFEECTLLCERWVSDEFMNAVVNFINRKSKL

>XP_003771952_ACBD7_Sarcophilus_harrisii

MSLQNDFHNAAEDVRKLKTRPNDEELKDLYGLYKQSIVGDIDIECPGMLDLKGKAKWEAWNLQKGLSKEDAMSAYISKAKELIEKYGI

>XP_003770143_ACBP-L_Sarcophilus_harrisii

MAQVEFELACAAVKQLTGPVTDEEKLVVYSYYKQATIGDVNIPCPEVTDFKAKAKWEAWNCRKGMSKLDAMRVYVSKVEELKSRQC

>XP_001368082_ECI2_Monodelphis_domestica

MISAISQLARWRPPIPGSLWKRQLPAVSFPALQLHTTSMAMRVSQEDFEKAKEQVSLLKKDPGNEVKLKLYALFKQATEGPCTTPKPGMLDFVKKAKWDAWNALGSLPKDAARQNYVDLVSSLVSSESLSQKKTSFNDTQSEYKTLIVTKEDNITKIMLNRPSQKNAINIQMYKDITLALEAAEKDDSSLTVITGNGEYYSSGNDLSEPLKIPPDEIQKKLEENMQILRTFIDHFIDFSKPLVALVNGPAIGISVTLLGLFDIVYATDRATFHTPFIHLGLCPEACSSFTFPKIMGSVKAAEILIFGRKLTAQEAYARGLVTEVFPESTFQKEVWTRLKAYSQLPPVSMKKSKQLIRSFEKETLHRVNLEECNLLQTRFTSDECLNAIVNFLNRKSKL

>XP_001367923_ACBP_Monodelphis_domestica

MSQAEFDKAAAEVKVLKSKPNDDEMLYIYSHFKQATVGDVNTARPGITDFKGKAKWDAWNSLKGKSKEDAIKAYIEKVEELKKKYGI

>XP_007504992_ACBD7_Monodelphis_domestica

MSLQDDFDNAAADVKKLKTRPNDEELKELYGLYKQSVVGDINIECPGMLDLKGKAKWEAWNLQKGLSKEDAMSAYISKAKELIEKYGI

>XP_001370361_ACBP-L5_Monodelphis_domestica

MAQVEFELACATVKQLTGPVSDEEKLLVYSYYKQATVGDINIPCPEVTDFKAKAKWEAWNCRKGMSKLDAMRIYVSKVEELKKKQSS

>XP_001508493_ECI2_Ornithorhynchus_anatinus

MHTSGTAMQVSQEDFEKAKDQVKLLKKDPGSEVKLKLYALFKQATEGSCNTPKPGMLDFVNKAKWDAWRALGSLPKENARQNYVDLVSSLVSSESPQQAKQPSGGEHQQYETLRVTTENNITKIILNRPERKNAISTQMYEELILALEAAGKDDSAIAVLTGNGDYYSSGNDLNNFMNVSPDKIEQKAKDGAVLLKNFVGHFIDFPKPLIAVVNGPAVGIPVTLLGLFDIVYATDRAKEMLIFGKKLTAQEAWANGLVTEVFPDSTFKEEVWARLKAFASLPRNAMRISKQLMRSVDREKLHAVNSQECQILEERWLSDECMTAIMNFFSKSSKL

>XP_003223610_ECI2_Anolis_carolinensis

MQFSQEDFEKAKDQLKLLEDDPGNEVKLKLYALYKQATEGPCKTPKPGMLDFVKKAKWDAWSSLGSLPQDSARQKYIELVSSLVSADSSMASGSKSDYQTLQVTTKDNITKIVLNRPKKKNAISTKMYNEIIEALEEAAKDDSVITVVTGNGDFYSSGNDMNNYINISPDDVEKKAKESAKMLKSFVGSFIDFPKPLIAVVNGPAVGIAVTLLGLFDIVYATDRATFHTPFSSLGLSPEGCSSYTFPKIMGLAKATEMLIFNRKLTAAEACSQGLVTEVFPDNTFQKEVWARLKAYASLPRNSLKLSKQLIRRTDKEKLNEANSKECACLQEIWASDECMNAVMNFFQKKSKL

>XP_003222139_ACBD7_Anolis_carolinensis

MERCKWKEARPGCKCSIKIVASLILLIIFLSHQADFDSVAEKVKKLKTRPTDDELKELYGLYKQVTVGDINIESPGALDLKGKAKWESWNQKKGMSKEDAMKAYISKANGLIQKYGL

>XP_003227602_ACBP_Anolis_carolinensis

MTSMPAFRVQLFQPPLGTATTQAEFDKAAEEVKKLKTQPADEEMLFIYSHFKQATVGDVNTERPGMLDFKGKAKWDAWNGLKEASERYQVSAVLSQVLRQGQE

>XP_007424768_ACBD7_Python_bivittatus

MTLQADFETVAENVKKLKSKPTDDELKELYGLYKQATVGDINTECPGVLDLKGRAKWEAWNLKKGMSKEDAMKAYISKANEMIQKYGM

>XP_007425599_ACBP_Python_bivittatus

MTQAEFDKAAEEVKKLKTQPTDAEMLDIYSHYKQATVGDVNTERPGMLDFKGKAKWDAWSALKGMSKEDAMKAYIAKVNELKDKYGMQ

>XP_015744757_ECI2_Python bivittatus

MFLAAVLKFLRPNPLRSCSPLCGISKVYGVHFPVLQLHTTNSTMQFSQEDFNKAKEQVKVLQDEPSDEVKLKLYALFKQATLGQCNTPKPSMLDFVNKAKWDAWHSLGSMTQDNARQSYIELVSSLVSAESSPVNEIPPGSKSNYETLQVTTKDNITKITLNRPKRKNAINVKMYNEIMEALDEAANDDSALTVLTGNGDYYSSGNDLNNFANISAGGMEESAKNGAVLLKNFVQCFIDFPKPLIAVVNGPAVGISVTVLGLFDIVYASDRATFHTPFSNLGQTPEGCSSYTFPKIMGLTKATEMLLFNKKLTAAEACSRGLVTEVFPDSTFQKEVWTRLKAYANLPKKVSVSKIAYIISCITWCYYILLRWNGIAISNVFSTVKFSNLNNKLDAKLNETVVDVKQLNDKLVILENNMDNKGMEEERDLDTLLDFKEKEPYFGLPVTSEWPNDDLILDLQKDEF

>NP_001135696_ECI2_Xenopus_tropicalis

MQARQEDFEKAQSNLKLLKNDPGNEVKLKLYALFKQATQGPCNVPKPGMLDFVNKVKWDAWKSLGSLPKDDARQSYVELVSSLVSSESSTKSNADPGIGHKKYETIHVSCEDNIIKIFLNRPEKKNAITLTMYKEIGEALEEAGKDESVFAVLSGFGDYFCSGNDLNNFTNIPPEGKEKMAKDSADLLETFVSKFIDFPKPLIAVVNGPATGISVTILGLFDLVYATDRATFHTPFSQLGQSPEGCSSYTFPRIMGLGKATEMLLFNKKLTAQEACNLGLVAEVFPDSSFQKEVWERIKDYSTLPKNSLAFSKQLIRVNEKEKLHAVNIQECERLKERWLSEECMNAIISFFQKRAKL

>NP_988874_ACBP_Xenopus_tropicalis

MSQEAFDKAAEEVKQLKSTPTDEEMLETYALYKQATVGDVDTARPGMLDFKGKAKWDSWKKKEGTSKEDARAQYVDWVEKLKAKYGS

>NP_001165067_ACBD7_Xenopus_tropicalis

MSPQADFDKAAEDVKKLKTRPTDEELKELYGLYKQSTVGDINIDCPGMLDLKAKAKWDAWNLKKGLSKEEAMHAYISKTNELVEKYGL

>XP_018425955_ECI2_Nanorana_parkeri

MAASLLYVTNCLRLSPFRAVSKLRSVSVLGVHTTARHMQASQEDFEKAQNDLKTLKKDPGNEVKLKLYAFFKQATQGACNVPKPGMLDFVNKAKWDAWNSLKDLPKEKARQSYIELVSSLISAELPVKDPTSTSANKKYETLEVFSQDNITKIFLNRPEKKNAITLQMYEEIGLALDEAAKDDSVITVLTGHGDYYCSGNDLNNFTNIPPEGKEKMASDSALILESFVGKFIDFPKPLIAVVNGPAVGISVTILGLFDVVYATDRATFHTPFSQLGQSPEGCSSYTFPRIMGLSKASEVLLFNKKLTAHEACQLGLVTEVFPDSTFQKEVWERLNSYSSLPKNSLAFSKQLTRAPEKEKLHAVNREECERLKERWLSEECMNAIISFFQKRVKL

>XP_018431086_ACBP_Nanorana_parkeri

MDEARRCKRIGGRVRVTPPVAERSEIPPPCNPEWKQEIYRIHQSGDGRRLLCVLLSQAEFEKAAEEAKKLTKKPADDEMLKLYALYKQATVGDVNTARPGMLDFTGKAKWDAWESKKGISQEDARAQYIALVEELKGKYSS

>XP_018422372_ACBD7_Nanorana_parkeri

MAPQADFDKAAEDVKKLTKRPTDDELKELYSLYKQSTVGDINIACPGILDLKAKAKWDSWNLKKGLSKEDAMSAYVSKAHELIEKYSH

>NP_001002645_ECI2_Danio_rerio

MASLIKHVSPWRFARFVRSSKTAFIPCVQLHSTAVMGASVEDFNKAKDKLNTLKKDPGNEVKLKIYALFKQATVGPCNTPKPGMLDFVNKVKWDAWKGLGSISQEEARQQYVDLISSLVGAEAPAVAAQPTGGGKTFQTLLVSTEDNITTIRLNRPDKKNAITVEMYNELIEALELAGKDDSVITVMTGNGDYYCSGNDLNNFTKIPEGGVEKMAKDAGELLRRYVKAYIDFPKPLIGVINGPAVGVSVTLLGLFDVVYATEKATFHTPFSQLGQSPEGCSSYLFPKMMSAAKASEVLLFNKKLSATQACELGLVSEVFPESSFQSEVWSRLKAYAKLPKNSLALSKQLIRGLEEEKLHAVNDAEVERLTERWLSDECMQAIMSFFQGKSKL

>NP_955902_ACBP_Danio_rerio

MSEAEFQKAAEEVKQLKAKPTDAEMLEIYSLYKQATVGDVNTARPGMLDFTGKAKWDAWDAKKGTSKEDAVKAYIAKVEELKGKYGI

>NP_001122240_ACBD7_Danio_rerio

MTLKAEFDQYAEDVKKVKTRPTDQELLDLYGLYKQAVVGDINIDKPGMIDLKGKAKWDAWDSRKGMSTEDAMKAYITLAKQAIEKYGK

>XP_018620717_ECI2_Scleropages_formosus

MAVQLLLYPARLVQVLRTPVLRLHVTARTMGATVEDFNRAKDQLGTLKKDPGNEVKLKIYALFKQATQGPCNTPKPSMLDFVNKAKWDAWKSLGSVSQEEARQKYVELIRLLVGDEESIQAAPTSAGSSAIFKTLLFNTEDNITTICLNRPEKKNAITVPMYSEIVKALEMASKDDSVITVITGSGEYYSSGNDLNNFTSVPEGDIEKKAKDAAELLKGFVKAFIDFPKPLIAVVNGPAIGISVTLLGLFDVVYATERATFHTPFSQLGQSPEGCSSYTFPKMMGAAKASEVLLFNKKLTATEACERGLVTEVFPDATFQIEVWKWLKAYSKLPRNSLALSKQLMRAVEKEHLYAVNAQEVQRLAECWQSDECLNAIVNSFFQAKAKL

>XP_018587841_ACBP_Scleropages_formosus

MSQAEFDKAAEEVKHLKVKPTDAEMLEIYSLYKQTTVGDVNTARPGMLDFAGKAKWDAWEKKKGMSKEDAMKAYIAKVEELKEKYGI

>XP_018618608_ACBD7_Scleropages_formosus

MSLKAEFEQIAEDVKKVKTRPEDQELLDLYGLYKQVIVGDVNTEKPGVLDLKGKAKWEAWNSRAGMSKDDAMTAYIALAKEVINKYGM

>XP_003457227_ECI2_Oreochromis_niloticus

MAVSTECRGVCGYLTANDICQTQVSHRDGLRLTGALLALKAEKYQLLYKQLAAASHDLCSTFRHDLKMRLSFILCSFSERVTVRSNIPSLKFHTTASPMMGVTVEQFEQAKSKLSTLKNDPGNEVKLKIYALFKQATQGPCNTPKPGMLDFVNKAKWDAWKSLGSISQDEARQKYCDLIGSLVEAESGSSAQVSAQPAGSGATYETLLVTKEDDITTIKLNRPAKKNAITTEMYNEIIAALEQAATDDSVITVVTGAGDFYCSGNDLTNFTKIPENGVEEMARHGADLLRKYVNAYIDFPKPLVAVVNGPAVGVSVTVLGLFDLVYATERATFHTPFSQLGQSAEGCSSYIFPKLMGAAKASEMLLFNKKLTAVQACELGLVTEVFPDSSFQSEVWTRLKAYAKLPRNSLALSKQLIRSVEKERLHAVNDAEVERLMERWTSDECFNAVMSFFQAKAKL

>XP_003456254_ACBP-L_Oreochromis_niloticus

MAELQTKFDEAAAEVKQLKAKPTDEEMLQIYSLFKQATVGDVNTSRPGMFDFTGKAKWDAWEKQKGKSKENAMNEYISLVEELKQKYGI

>XP_003450922_ACBD7_Oreochromis_niloticus

MTLQAEFDKAADDVKKVKAKPTDEELLFLYALYKQAVVGDINTERPGMLDLKGKAKWDAWESRKGMSKEDAMSAYIAKAKEVISKYGV

>XP_003440905_ACBP-like_Oreochromis_niloticus

MTEAFHKAVEEVKVLKQKPNQQEMGDLYGLYKQATVGDINTERPGFLDFTGKAKWDAWNARKGLSKEEAMVKYVTLVEELKEKYGI

>SINCAMP00000011604_ECI2_Callorhinchus_milii

SVWCFLPILILHLHCRLIQSFPAVKLHLTGAVMRATEVEFDKAKEHLKTLKNDPGNDTKLRIYALFKQATQGSCSSPKPGMLDFVNKAKWEAWNSLGNISKEDARQKYVDLVGTLISSEAPIQKEATTAGDPKSSYQTLQVTTENNITTILLNRPEKKNAISRKMYEEIMQALEQAGKDDSVLTVMTGSGDYYCSGNDLSNFTQVGAEGVEKLARDSGELLTRYVSHFIDFPKPLIAVVNGPAVGIAVTGLGLFDVVYATDRATFHAPFSQLGQSPEGCSSYTFPKMMGTAKANEILLFNKKLTAAQACDLGLVTEVFPDNTFQQEVWKKLRAYAKLPKDSLRYSKQLIRGMEKEKLHAVNAQECVRLQERWLSEECMNAIMSFFQNKSKL

>SINCAMP00000019993_ACBP_Callorhinchus_milii

MTEVLGQWRCSFTGKVILLCYKRIAFQKAAEEVKHLTTVPTDEEMLAIYSLYKQATVGDVNTERPGMLDFKGKAKWDAWSKLKGTSKEEAMKLYIAKVEEMKVIYGMAE

>XP_005991125_ACBP_Latimeria_chalumnae

MSQAEFEKAAEEVKNLKSKPNDQDMLDIYSLYKQATVGDINTARPGMLDFAGKAKWDAWEARQGMSKEAAMKAYIQKVEELKGKYGI

>XP_006010031_ECI2_Latimeria_chalumnae

MATALLGISRWQLLRKARSEFSPPANVLQLEAGFHSGTWRPSKRFVQALYPPRLQAHTTGAAMGATQEQFEKAKEQLGLLKNDPGNEVKLQIYALFKQATQGPCNVPKPGMLDFVGKVKWEAWNSLGNLSKEGAREKYVGLVSSLVSSESSPQAEAVSVAGKQLYKTLQVTTEENITTIRLNRPEKKNAISIVMYNEIMQALEEAAKDESTITVITGTGDYYCSGNDINNFTMIPPEGIEKMAKDSAQLLKNFVLHFIDFPKPLIAVVNGPAVGVSVTLLGLFDAVYATDRATFHTPFSQLGQSPEGCSSYIFPKLMGSAKATEVLSFNKKLTAREACDLGLVTEIFPDASFQKEIWLRLRGCAKLPKMSLVYAKQLSRGPEKEKLYAVNIQECERLEERWLSEDCRNAIAKFFTRSKI

>XP_005994698_ACBD5L_Latimeria_chalumnae

MEESTQCNQVTSDSESELYCDSVEQLEQEKGSQLFANQNLSFGAVDSSQFISNTVPERQMVVQMEASQGGERLRQSGSPRRKTNSGGSGSRLQHQRGMELPSGPPNQQISGSNEGEKKQPDWKQPGDLNNQIGLLLLRLQEDMRIVLLRLNTLEARTVSQTEPTDSQSAELQIPLPSTKLLHDATKSCCRHVSTTW

>XP_005998554_ACBD7_Latimeria_chalumnae

MQGVAASAKPSVELHLSTEIPAAHCSSAMTLQAEFENMAEDVKKLKTRPTDDELRDLYGLYKQAIIGDINIECPGMLDLKGKAKWEAWNARKGTSKEDAMTAYISKAKELIEKYGI

>XP_006636426_ACBP_Lepisosteus_oculatus

MSQADFEKAAEEVKILKTKPADKEVLEIYGLYKQATVGDVNTQRPGMLDFTGKAKWDAWDANKGMSKEDAMKKYIEKVEELKAKYGI

>XP_006636067_ACBD7_Lepisosteus_oculatus

MSLQADFEKMAEDVKKVKSRPEDKELLELYGLYKQATVGDNHIDKPGLFNPKADAKWEAWNSRKGMSKDDAMTAYNALAKEVINKYGM

>XP_015210254_ACBD2_Lepisosteus_oculatus

MAAAVSLLYSWRFMKSARLFQSFRLPALQLHTTGALMGATVEDFNHAKDKLSKLKKDPGNEVKLKIYALFKQAMQGPCNIPKPSMLDFVNKAKWDAWKSLDSLSPEEARQKYVDLVESLVAAETPAQPKTSSTGGQATFETLLVTTEDNITTIVMNRPEKKNAITVLMYNEIKQALELAANNDCVITVLTGSGNFYSSGNDLNNFTNIPAGGVEKMAKDSGDLLREFVETFIDFPKPLIAVVNGPAVGIVVTLLGLFDVVYATDRATFHTPFSQLGQSPEGCSSYVFPKIMGNAKANEMLLFNKKLTAHEACNLGLVTEVFPDSTFQKEVWTRLKEYAKLPKNSLAFSKQLIRGIEKEKLHLVNNQECERLVERWLSDECMNAIMSFFQAKSKL

>XP_007057109_ACBP_Chelonia_mydas

MSQAEFDKAAEEVKQLKSQPTDEEMLYIYSHFKQATVGDINTERPGFLDFKGKAKWDAWNALKGIPKEEAVKAYIAKVEELKGKYGI

>XP_007058509_ACBD7_Chelonia_mydas

MMKSSVALKADFDSAAEDVKKLKTRPSDDELKELYGLYKQSTVGDIDTECPGMLDLKAKAKWEAWNLKKGLSKEDAMTAYISKAREMIEKYGI

>XP_007057802_ACBP5L_Chelonia_mydas

MSQEEFEKAAAMVREMKVPNSDQEKLEIYSLYKQATVGDINIPCPAATDVIGKAKWEAWNGHKGMSKANAMKNYIAKAEELKKKHVAKN

>XP_007058913_ACBD2_Chelonia_mydas

MRVSQEDFEKAKDQLKLLKEDPGNEVKLKLYALFKQATEGPCSSPTPGMLDFVKKAKWDAWSSLGSLSKDNARQKYVDLVSGLVSSESSSQVKDTTPDSKHGYETLQVTTTDNITKIMLNRPEKKNAITTQMYREIIQALEEAAKDDSVITVITGNGDYYCSGNDLNNFTNIPPGGIEKMAKDAAVLLENFVNHFIDFPKPLIAVVNGPAVGISVTLLGLFDIVYATDRATFHTPFSELGQSPEGCSSYMFPKIMGLSKANEMLLFNKKLTAGEVCAQGLVTEVFPDRAFQKEVWMRLKAYANLPKNSLALSKQLIRGVEKEKLHAVNRQECELLTERMNFFGKKSKL

>jgi|Brafl1|224202|ACBD5-B_Branchiostoma_floridae

MAAPKARFDAAVKVIQSLPKNGSITPSHETMLTFYGYYKQATIGPCDISRPGFWDVVGKAKWEAWNRLGNMPKEEAMDNYVDTLKKIIEALPQDKEMQDFMHVLGPFYELVD

>XP_002594078_ECI2_Branchiostoma_floridae

MGRTAFGAHDAAFEAAKERLNTLKEEPDNNVKLQIYALFKQATKGPCNTPKPGAFDFVGRAKWQAWSGLGDISQDEAQKQYIDIINGLAGEESPAETEQAGEEASSYKEIKVTKENKVCSILLNRPAKKNAITWLMYNEIVQALDDASKDDSVTVAVITGAGDYYCSGNDLGNFMNIDPKDMPKMARDGKELLRRFVTAFIDFPKPLIGAVNGPAVGVSVTVLGLFDAVYATDKATFHTPFTELGQSAEGCSSYVFPKLMGNTKANEMLLFNKKLTAHEACERGLVTEVLPHDSFQKEVQTRVEYVAQLPPQSVREGKKLVRDQEREDLHKANEKECEVLEGRWLSEECVRAIMSFFTKKARL

>XP_002602095_ACBP-L_Branchiostoma_floridae

MSEADFEKAAEEVKNLKSSPTDEEKLEIYSLFKQAKIGDVNTARPGMLDFTGKAKWDAWESKKGMSQEDARAKYIAKVEELKGKYGV

>XP_027207005_ACBP_Penaeus_vannamei

MSLEENFNKAAEQVKNLQQQPTDDELKEIYSLYKQATVGDINTERPGMLDFKGKAKWDAWSSKKGMSKEAAMEAYVAKAEQLISTYGLKA

>XP_027214797_ACBD2_Penaeus_vannamei

MASLRCATRLASVYKRGIGAGFGVASHQLAPAWGAAATQVRLMSALSPEFESAKARLGTLSEDPGNEAKLKIYALFKQATVGAVNTKRPGMMDFVGRAKWDAWNSLGNMSQEEAQKNYINFVNSLAGAEEEQAKAQAESGQKYKNLLVTCENGLRTITLNRPAKMNAITVEMYEEWIAALKEAAEDPATVITVTTGAGNYYCSGNDLSNFANISPENMHEHSKNAGVLLNRFVSAFIDFPKPLIAVINGHAIGVSVTVLGLYDAVYATDKATFNTPFSNLGQSPEGCSSYTFPKIMGPGKANELLLFNKKVKLFANM

>XP_013771945_ACBP_Limulus_polyphemus

MSLDEKFNKAAEDVKNLKNKPTDEELLEIYALYKQSVIGDCNTDRPGLLDPKGKAKWDAWNSKKGTPQDSAKETYIAKANQLVESHGLK

>XP_013791191_ACBD2_Limulus_polyphemus

MALRRGVSQVYLNLKADRCTRKWPLTQFTKQHQNLFAQSSFFGCLGVRMCSNLNSRFDSAKEKLNTLKEDPGNKVKLRLYSLFKQATVGLCSGKKPPVFDFVAKAKWEAWNSLGSMSQEDAMKEYIKTVEELAGKEKVTSDNSQSSSNSSQYNGLQVSTQNGITTVKLNRPEKKNALTTQMYRDISEILSNTAKDKSSVITLFTGAGDYYCSGNDLGNFNLPPGVDIAQMAKDASVLLRDFVDSFITFPKPLVAAVNGPATGISVTLLGLFDVVYASEKATFHTPFSLLGQSPEGCSSYIFPKIMGPSKASEMLLFNRKITAAEAKSCGLVSEVFPDTTFEVDVSNQFKGLEEMSINSIIYAKKLVRDLDRDLLHKVNKEECERLVERWQSEDCMKAIMAFFQKRSKN

>XP_003250509_ACBP_Apis_mellifera

MTLDEKFKKAAEEVKELSAPASDADLLELYSLYKQATIGDCNTSKPGMLDFKGKAKWDAWDKRKGMSQDAAKEQYIHKVEELISIIGKNTTTTGSTETCECIERSKVS

>PSN52303_ACBP_Blattella_germanica

MKNRFNKAAEDVKNLKTQPTDEELLELYALFKQSTVGEVNTARPGGLLDFKGKAKWDAWSNKKGMSQDEAKEAYITKVQLLIDTYGTK

>NP_648081|CG8629_Drosophila_melanogaster

MVSFEEAAELAKNFSKKPTDSEFLEFYGLFKQATVGDVNIDKPGILDLKKKAMYEAWNAHKGLSKDAAKEAYVKVYEKYAPKYA

>NP_648083|CG8628_Drosophila_melanogaster]

MVSFEEATELANKFTKKPTDAEFLEFYGLFKQATVGDVNIEKPGALALKDKAKYEAWSSNKGLSKEAAKEAYVKVYEKYAPKYA

>NP_648255_CG5804_Drosophila_melanogaster

MADFNAILEKTKAFSKKPPTEVYLEFYGLYKQFQEGDINIEKPADAEGAAKYDAWLSRKGLSVDDAKAAYVALYEKYNPIYG

>NP_648082_CG15829_Drosophila_melanogaster

MPTFEEIVEKAKNFKNLPSKEEFLEFYGYYKQATVGDCNIEEPEDEEKKARYNAWKSKAGLTADDAKAYYIEVYKKYAPQYE

>NP_729218_ACBP_Drosophila_melanogaster

MVSEQFNAAAEKVKSLTKRPSDDEFLQLYALFKQASVGDNDTAKPGLLDLKGKAKWEAWNKQKGKSSEAAQQEYITFVEGLVAKYA

>XP_974824_ACBP-like_Tribolium_castaneum

MSLDERFKKAADDVQKLKSKPSNDDLLEIYALFKQGSVGDCNTDRPGMLDLKGKAKWDAWNGKKGMSQDKAKEEYIAKVESLIQSIGLQ

>XP_974813_ACBP-like_Tribolium_castaneum

MSLDEKFKSACDQIRQFTKRPSDSDMLEVYSLYKQATVGDINTPKPSEAKAKAKWEAWSGKKGLNANVAKEQYVAKIKALAPTYA

>XP_021917064_ACBP_Zootermopsis_nevadensis

MSLEAKFNEAAESVKNFKKRPSDDELLQLYALFKQATDGDNHKEKPGMLDLKGKAKWEAWSDKKGQSKEVAMEAYVALANKLEARYA

>XP_021917062_ACBP_Zootermopsis_nevadensis

MSLDERFIKAAEDVKTLKTSPNEDELLELYSLYKQGSIGDVNTPRPGGLLDFKGKAKWDAWNRKKGISQENAKEAYIAKAQLLIETYGTK

>JAN53111_ECI2_Daphnia_magna

MIIPTAANSFRSQCLAAMLLKRSFVNSPNQRTFLAPANGIKHMSTSISQQFDEAQKRLQTLKTSPGNEAKLKLYGLFKQATAGAVNTKRPGMTDFVGKAKWDAWNSLGSMSQEEAKNKYIEFVDSLVGPANSTESLNVVESSSPGFDVTIDGKLRIITLNKPTTKNAFTLGMYVGFAKLLKEAAEDPNTTLVAVTGAGNIFSSGNDLTSFTSFTGTMREAAEEGKRCLSIFVGSLIDFPKPIIGVVNGPAVGIACTILGLMDVVYATDRGWFQTPFSALGQSPEACSSHIFPKLMGSLKANEMLFFNKTITATEACKLGLVTSVLPDANFQSEVWPKLKEWSELPQKVSDRRKHFIDSAIKKLTSLFQSLVHSKELSRQFDRELLHKVNAAECDRLLERWQSSDCMEAVMKFFSKNAK

>KZS11387_ACBP-L1_Daphnia_magna

MSLDEKFNKAAESIRSMTTSPSDDEMKEIYALYKQSTVGDVNTARPGMLDLKGKAKWDSWESKKGMSADEAKEAYVTKTEELVAKYSASSA

>JAN71011_ACBP-L2_Daphnia_magna

MVVMAYKSVDANRRPQKFTVDYQERLPLRSTMFWGVLIETSSNDKQLQYFTFFALLSFPFIFFSQLTHIMSLDQKFIKAAEDIKALPATPTNDELKDLYALFKQATVGDVNVARPGMLDLKGKAKWDSWESKKGMSSDAAKEAYIAKSAELVAKYIS

>KZS07345_ACBP-L3_Daphnia_magna

MSLDEKFNKAAEDIKALTARPTDDELKEIYALFKQATIGDVNVARPGMLDFKGKAKWDSWESKKGMSSDAAKEAYVAKTEELLAKYSA

>JAN37855_ACBP-L4_Daphnia_magna

MTTSPSDDEMKEIYALYKQSTVGDVNTARPGMLDLKGKAKWDSWESKKGMSADKLRKLTSPRRKSWWPSIVHQVHXSIA

>ODN05914_ACBP_Orchesella_cincta

MADFETSAENVKNLVKKPSDDELLEIYGLYKQATVGDVNTARPGMLDLKGKAKWDAWNNLKGTSQEDAKTKYIALVQQLVEKYGLEKK

>ODN01204_ACBD2_Orchesella_cincta]

MNSSCRITKQLLLRNQSLTGFKRCFSNRPTSHKLSSSFLGNSYSPTPLAKSPVVTAWSAKNIIWTSQRNYSMSFEEAVKNSTSMKDPDNSTKLKMYALYKQATTGKPTGKRPGAMDFVGRAKYDAWNGLGDMSQEEAKKQYIELVQSGLGDSAGGGGASAGQSSSSDSSADGLDITVADGIRTIRLNRPDKKNALTHAMYRGILNSLKEANDDANTRVIVITGTGDYFCSGNDLGNFANVTDPMKMAKDGRDLLMEYVDAYIYCKKPLVGILNGPAVGISVTVLGLFDAVFTTDKATFTSPFSALGQSPEGCASYTFPRIMGYAKASELLYFNQKITAAEAEKLGLVTQVIPHDRLEAEAWKRVRDIAQLPLKSLVYSKDLVRGRERELLHQVNVAECDRLQERWTSEDCMNAIMKFFSKKSKV

>ODM92257_ACBP-L_Orchesella_cincta

MANTKEKFDRAVKIIQELPKDGPYQPTTDEKLKFYGLFKQVTVGKNNTKRPGLLDFVGKAKWDAWNGLGEMTADEAMEQYVEEFEKMEQKMREMGLVPA

>XP_021948487_ACBP_Folsomia_candida]

MSDFDTAAEQVKNLKGKPTDEELLDVYGLFKQATVGDVNTSRPGMLDLKGKAKWDAWNSRKGMSQDDAKSKYVDLVKTLVGKYGL

>XP_021960098_ACBP-L_Folsomia_candida

MSEDAELDAEFERVVQLVQNLPKEGPYQPSNGEKLRFYSLYKQATVGPCDTSRPGFFDLTGKAKWDAWNELGDMSKTDAKRKYIEAFAKMEQKMKDLGHTQ

>OXA46062_ACBD2A_Folsomia_candida

MGYRWKGIGEGIQNMYTLLGLKPLPASLEAKERKSAVVQNLTQQEWFQTPQSIHILNPLANTFPTIPHLCGYVTFDIVTFRSPKKVTQVDFDFLGIMFARSFFQRTGYKISSPVSVELRRLIRNHPFLRMSSSTNFSEAVANSSKIKSASNEEKLKLYSLYKQGTIGRNRTQQPGMFDLVGTAKWDAWNSLRDMSMDDAQKNYVTLVNGLLGVKIDDGEKIYSQSSGPVLVSGVNRIRIITWNRPEKKNAMNEEVQFTLGNNDGMGHFPQKSYLFHVNKPPLPLQSQNSNAPYVPQLQIIATNFQMYVTLTKLLRDFNNDETVDIVVITGSGDYFCSGNDLGNFMSVDMNDKENEVKKWIGIIE

>XP_021960613_ACBD2B_Folsomia_candida

MSSSTNFSEAVANSSKIKSASNEEKLKLYSLYKQGTIGRNRTQQPGMFDLVGTAKWDAWNSLRDMSMDDAQKNYVTLVNGLLGVKIDDGEKIYSQSSGPVLVSGVNRIRIITWNRPEKKNAMNEEMYVTLTKLLRDFNNDETVDIVVITGSGDYFCSGNDLGNFMSVDMNDKENEVKKWIGIIEDHIASYIDFKKILIGIVNGPAVGLGVTSLPFFDAVFATERATFNTPFTALGQTPEACSSFFLPRIMGYAKATQMLLFGTKITAREAEQCGLITEIFPDDVLKSNAWDRIHKMAELPINALLHAKELLRGRDRDLLHHVNKTEMVRMKERVNSEDCMDAVLKFFARKTKK

>XP_018023004_ACBP-L_Hyalella_azteca

MSTETLFQEAAEKVKKLAKQPTDDELKEIYGLYKQATIGDINTERPGMLDFKGKAKWDSWESKKGMTKEAAMTAYVAKVEELVAAYGLQ

>XP_018025342_ECI2-L_Hyalella_azteca

MVAKSFLAASIPFRRCFFNSTANHVVNVQACRNLGPSFIGGAPRYMSSSIAQQFEEVKHRLASLKEDPGNEVKLKMYALYKQGMEGKATGKRPGVMDFVARAKWDAWNSLADMSKEEALKAYLAIVDELSAAQGATSTEEPSILVTVEDGLRIIKLNRPKKKNALNPEMYFRWTELMHEAAKDDKTVLTAITGAGDFFCSGNDLGNFMNIPPGGEAELANRTKEFLYQFIDAFIEFPKPLIGVVNGPAVGVSVTTLGLYDAVYSSDQAWFQTPFSQLGQTAEGCSSYVFPRLMGPGIASEMLMFNKKLSAHEAQRYRLVTEVFPHDRLQQEVWPRLQALAKLPARCLIYSKELTRAADKEILKKTNRAECERLCERWQSEDCKNAIMNFFSRQRK

>XP_015922765_ECI2_Parasteatoda_tepidariorum

MSIIFSSIRKLSSSLRPSIFLPYSKSSKSIYLLKSFSTSVRMNSSLTFQQACDKAKTLEKDPGNDVKLKMYALFKQATEGPCSQTKPSVFDMVGKAKWEAWNTLGSLSQDEAKSQYVSLINGLFDQKQQSEQSAEIQELKYEGIKLSVSGDITELRFNRPEKKNAITTKMYKDIALALKEASENDTAITILTGEGDYYSSGNDLSNFASVDQDISAAAKKAGQLLNEFVGAFIDFPKILVAAVNGPAVGIPVTLLGLCDVVYASDKATFQTPFSFLGQSPEACATYTFPKIMGYSKANEVLLFNKKFDAVEAKSMGLVSEIFPNDTFHEDVKRKLEFLSQASKQSMILSKELIRKFEKDTLHTVNDEECKLLVERWQSQDCMEALMRFFTRKSKL

>XP_015907443_ACBP-L1_Parasteatoda_tepidariorum

MSLEEKFTKAAEDVTKLKTKPTDEELLEIYALYKQASIGDVNTTRPGLFDLKGKAKWDAWNSKKGMDQTEAKEAYVTKVNHLIEIYGLA

>XP_015904839_ACBP-L2_Parasteatoda_tepidariorum

MSLDEKFNTAATSVKDMKSRPSDSELLELYSLYKQATCGDCSADKPGALDLKGKAKWEAWTGRKGMAQDAAKEAYVTLANTLITKY

>XP_780031_ECI2_Strongylocentrotus_purpuratus

MAALFSLKCGTRRLFNICNTCTTRLHAPVLKRQIQNTACMMSFSDAEFTAAKDRLNSLKEDPGNEVKLQIYALFKQATAGTCNTPKPGAFDFVGKAKWSSWNSLGNMSQDQAKEKYVGIVDDLVAQEGGEAETASTTQGSLSFTGLKYTVDNGVATITLNRPNKKNAVTTEMYSEWTAALKMAGEDDRVVLAVITGAGDYYCSGNDLNNFMKIDPSVLHEESVKGSDLLEIFVNGFIDFPKPLICAVNGPAVGISVTTLGLMDVIYASDKATFHTPFAALGQSPEGCSSYTFPKIMGTAQANECLLFGKKLTAQEAFDRGLVTEVIPDAQFRETVDKKVKEYAQLPRNALRLAKNLIRETEKERLHKVNRAECDLLVDRWTSDECTQAIMNYFSKSKL

>XP_784299_ACBP-L_Strongylocentrotus_purpuratus

MSDAFNTAAAEVKDLATSPSNEDLLKIYSLFKQVTVGDCNTDRPGMLDYKGKAKWDAWNGLKGKSTADAEKEYIELVGQLKTSCGMK

>XP_022079880_ACBP_Acanthaster_planci

MADRRIGSDAFEVAAEEIKRLNKVPDNQELLDTYKYYKQVIIGDCNTARPGILDQKGRAKWDAWNGIKGMAKEEADKLYVEYVNKLKEKYGLQQ

>XP_022091979_ACBD2_Acanthaster_planci

MGVTDAEFSAAKERVGTLTQDPGNEVKLKMYGLFKQVAVGKCNAPKPGAFDFVGKAKWGAWNDLGDMSKEEAKEKYVDLVNDLVAKDPGAKATSAPAGTQEGTSAASSSTGGYENLKVTIDKGVCTIMMNRPHKKNAITRETYEEIIKALDSSGRDDSVVLSALTGAGDFYSGGNDLNNFMSITPDTMKQAAKDGGELLERFVAAFIDFPKPLVAAVNGPAVGIPVTSLGLCDVVYASDRATFHTPFSSLGQSPEACSSYLFPKIMGQAKSNEVLLFGRKLTAQEAYDRGLVTEIFPDATFRQDVQNKLDEFATLPKQSMRLSKTLIRSAEREKLHQVNKDECELLVERWTSEECIQAIMDFFAKKSKL

>XP_002126596_ECI2_Ciona_intestinalis

MLSSKVDGDFETAKQRLNTLKQEPGNDVKLQIYALFKQATIGANNTKRPGTFNFVGQAKWNAWNDLGPMSKDEAKEEYVKIVKDLSDAEGEAEEEVGDVSSSTDYQNLIVTKQNNYTKIVLNRPTKKNALTREMYEEIIVALNEAGKDDTAVTVMTGAGDYYCSGNDLGNFMVIKPEEMHQVAKESGDLLRRYVNAYIDFPKPLVAAINGPAIGVSVTALGLFDLVLASENATFSTPFSRLGQSPEGCSSYTFPKIMGHAKACDMMLFNNKLTATEAKECGLVTKIFPKESFETDVMSQVEAIAKLPVKSLIYSKALMRDPELDLLHKVNEAECDRLVERWPSEDCINAIMKFFQEKNK

>XP_015747922_ACBD2_Acropora_digitifera

MAFCLRSLLHAKKLSFRPTPFTFSILRQIQIGNIRQVEGDKDFQAAVAKVKTLKQDPGNDNKLKLYALFKQASAGKCNEPKPGAFDFVGKAKWTAWNDLGDLSKNDAQQKYIIYVNDLVDKFGSTDEESNVVSNSEEKTTNMGSKYKELEVTLENGVQKIRMNRPAKYNAITWEMYHEFMTALEEGGKNDACVVAMVTGTGDYYCSGNDLSNFTRIPPEGPQKMARDAREILKKFVAHFIDFPKPLIAAVNGPAVGISVTIMGLFDLVYASDQATFHTPFMELGQSPEGCSSFLFPRIMGPAKANEMLLAGRKLTAMEAHQCGLVTDVIPHNNFQGEIERRVQALGKLPPKSLRLSKELIRDSSRDLLHEANEKEADLLEERWLSEECMQAIMNFMARKSK

>XP_015749156_ACBP-L_Acropora_digitifera

MSEAFLKAAEEVKTFKTEPSDSDKLELYAFYKQATVGDCNTDRPGMMDFKGKAKWDAWNGKKGTSKDAAETTYIAKVEELKKTCS

>XP_013417380_ACBP_Lingula_anatina

MSNSPEFTQAATDAKNLKSKPTDAEMLELYGLYKQATIGDVNTDRPGMLDFTGKAKWDAWNERKGLSKEDAEKNYIDVVKKLQETYGMK

>XP_013391752_ACBP-L_Lingula_anatina

MKMTFEPEFLKAAEDVKNLKSKPTDAEMLELYGLFKQAMIGDVNTDRPGMLDFTGKAKWDAWEGRKGMSKENAMELYISVANNLVEKYGML

>XP_013404825_ACBD2_Lingula_anatina

MGLDEDFVSAKERLNALKEDPGNEVKLQIYALFKQATVGKCNTPKPGMMDFVGKYKWEAWNTLGDMSQDEAKSKYIEVVQGLAAAEGATSPQVADTESAGTGKYKELTVHRDGKMFVITLNRPKKKNAINYQMYEEWGLALAEAAESDATIAVVTGAGDYYCSGNDLSNFMNIPPDGIAAMAEEGGRILKKFVSAFIDFPKPLLAAVNGPAVGVSVTVLGLFDIVYTTDTATFHTPFSALGQSPEGCSSLTFPQIMGTAKANEMLLFNRKLTAAEAMERGLVTEVFPDHSFQTEVWTRLREMAKLPPVSLRTSKGLIRDVDRAALHATNQRECDLLVDRWQSDECMNAIMNFFQQKAKL

>XP_020911023_ACBD2_Exaiptasia_pallida

MAARLTNISKFPKFSAIRGIRTTVRPLFASKEFDLAKDRVNTLKEDPGNEAKLKLYALFKQATVGACNSPKPGAFDFVGKAKWTAWDSLGSISKEEAEQQYIQYVNDLAAKIGTTEDDAASSATEESDSGKKYKSLEVTVKDGVQTIKLNRPKKYNAITWEMYEEWISALEEGAQDKSCVVTLITGAGDYYCSGNDLGNFAQIPPEGPEKMARDGRNVLRRFVSAFIDYPKPLVAAINGPAVGISVTVLGLFDVVFASDRATFHTPFIELGQSPEGCSSYTFPAIMGPALACQVLLASRKLTAAEALESKLVSEVFLHGDFQREVESRITAMAQLPPKSLQLSKQLIREANKATLHEVNERECILLEQRWLSEECMQAIMKFMQRKAK

>XP_020893157_ACBP_Exaiptasia_pallida

MSAAFETAAAEVKNLNAKPTDEEMLEVYALYKQATVGDCNTARPGMIDFTGKAKWDAWDGKKGISKEDAEQKYIAKVEELKGKYGMK

>XP_002741676_ECI2_Saccoglossus_kowalevskii

MAFCRRIGVLAFDSVVMKTSRFSVIRPVSTSGKNFAAIDTEFSAAKERLTTLKEDPGNEIKLKLYALFKQATVGKCNAKKPSTFDFVGKAKWTAWNSLGVLSQEDAQKQYISTVNDLVAVEESASEEVASGTAGDYKQIKVTVEDGVCTILLNRPAKKNAINLEMYNEIGVALNEIGKDPKVVLAVVTGAGDYYCSGNDLENFMNIDPSKIHEFAKEGADVLRQFVASFINFPKPLIGAINGPVVGVAVSTLSLFDVVYATDRATFHTPFTALGQSPEGCSSVLFPRIMGQGKVCPGF

>XP_002736654_ACBP-L_Saccoglossus_kowalevskii

MPSDAFNKAAEDVKNLKTRPSDAEMLKLYALFKQTSVGDCNTDRPGMLDFKGKAKWDAWNEKKGTSQADAEAKYIELVEELKGKYGMA

>XP_025094826_ACBP_Pomacea_canaliculata

MGDEAFQKAAEEVKNLKQKPTDDEMLKVYALFKQGTVGDCNTDRPGLLDLKGKAKWDAWNAKKGTAQGQAKEDYVKLVEELKAKYN

>XP_025114892_ACBD2_Pomacea_canaliculata

MMLLRTVRRATFFRREVGAQLCEFHQSVLYRMPAHDEAFEAAKNRLNTLKEEPGNDVKLQIYALFKQASKGKCDAPKPGAFDFVGKAKWEAWNALASMSQDDAQQKYIQLINKLTAAEDVTPNPQGANADKYPGLQITKDNKALRILLNRPDKKNALTWQMYDNIAETLKEAADDKNIAVAVITGAGDYYCSGNDLANFTNVTPEQVPEMARKGKVVLQNYVKSFIDFPKPLIALVNGPAVGISVTVLGLFDAVYCTDRATFHTPFSQLGQSPEGCSSYIFPKIMGQAKAGELLFFNKKITAQEAEERNLVTRVFPENVFKKETDALVAYYSTLPPKSLEFSKILTRFAEREVLHRVNEAECDRLVERWQSEDCINAIMSFFSRKSNL

>XP_012379078_ACBP_Dasypus_novemcinctus

MSQAEFDKAAEEVKNLKTRPSDDEMLFIYSHFKQATVGDINTERPGMLDFKGKAKWDAWNQLKGTSKEDAMKSYIDKVEELKKKYGI

>XP_023442489_ACBD7_Dasypus_novemcinctus

MGHFCLRPSFSWSPHCLVIQCPKGWRTCFQRVKAPSALFLLLXADFDKAAEDVRKLRTRPDDDELKELYGLYKQSVIGDIDIKCPVMLDLKGKIKWEAWNLQKGLSKEDAMSAYISKAKELIEKYGI

>XP_004483852_ACBP-5L_Dasypus_novemcinctus

MCQAEFELACAAIKELKGPVSDKEKLLVYSLYKQATQGDCNIPAPPSSDIKAKAKWDAWKENKGMSKMDAMRLYIAKVEELKKNEAG

>XP_004473600_ACBD2_Dasypus_novemcinctus

MAALAVVLARGWRQSAGRSPLQVVVSPGPQVHTSSSAMRASQKDFENAVNQLKHLKEDPGNEVKLKLYALYKQATEGPCRVPRPGVLDMINKAKWDAWDALGSLPKETARQNYVDLVSSLGSPSESSSQAEPGGAVKQGGCEALVVTREDGITKIMLNRPTKKNAITLQLYQEIMLALEAASKDDSAITVVTGNGDYFSSGNDLNNFTESLPVSPEEKAKNSAVLLRDFVGHFIDFPKPLIAVVNGPAVGIAVTLLGLFDVVYASDKATFHTPFSHLGQTPEGCSSYTFPKIMGSAKASEMLLFGKKITAREACAQGLVAEVFPDSTFQEEVWARLRAYAKIPPNAMRFSKHTIRNREREKLHAVNSEECSILQERWLSKECMNAVVSFLSRRAKL

>jgi|Lotgi1|84981|Lottia_gigantea

LTESFELATEFVRVVAAKLKSEDLLYLYARFKQANEGRCKTPKPSFFDFQGKQKWEAWKKLEDMPTLTAKKDYIEYLSKIIPGWQHQEARDVNGGGDTAGLGIAVSRMVCEDDDIDDCDKTVFDWCKDGNVDKVKTCLNENTLNVDKLDEE

>jgi|Lotgi1|205113|Lottia_gigantea

MSAEFTTAAEEVKNLKEKPADAEMLEIYALYKQATVGDVNTTRPGMLDFTGKAKWDAWEAKKGTSKEDAEKAYIAKVAELKGKYGMK

>jgi|Lotgi1|178218|Lottia_gigantea

MPAHDAEFNKAKERLNTLSEAPGNDIKLKIYGLFKQATEGKCNKPKPGMMDLVGKAKWQAWNGLGDISQDDAQKKYIDLVNELIAADSPEPTSTTDSKFKTILVNKENKIYKITLNRPKKMNALNHLMYEEIMQALDEAGKDNSVLTVITGTGNYYCSGNDLENFTSTGGADIPAMAKQAREILYRFVGSFIRFPKPLIALINGPAVGISVTTLGLFDVVYCTDRATFHTPFSSLGQSPEGCSSYIFPQLMGSAKASEVLLFNKKLTAQEALDRNLVTEIFPDASFEKETMARVTKYAQLPPQSLQKSKNLMRQMQKDKLDKVNSEECDLLVERWQSDECMNAIMNFFKRKESKL

>XP_003386304_ACBP-like_Amphimedon_queenslandica

MSNSEAFLKAAEDIKTLTTSPTNEEFLDLYKFYKQATVGDCNTDRPGMFDLKGKYKWDAWNSLKGTGKEEAEKKYVDLVTELLTKYAN

>XP_003384577_ECI2-like_Amphimedon_queenslandica

MSLVFARRVLQGRAPLLLSSSSSAVPSIHMLWRRSFSLSNEDKEKLEAAKGKVSQLTTDPGNDKKLELYALYKQAVEGPCDTPKPGVMDFVGRAKWNAWNSLGQLSQSEAAVKYSELVDSLVGSSTSNETASPSNPVKLSSDDLLVTEEGGVQTITLNRPSKKNAITVKMYEDITSLLNSSASNPAIKATVVTGNGDYYCSGNDLSNFMNIPPEGPEKLAADSAILLRNFVSSLIRYPKPLVACVNGPAVGISVTTLLLFDLVYAADNATFHTPFMQLGQSPEACSSFLYPRIMGPAKSNELLILGRKISAEEAFERNMITRVFPKDELQERVKEIVRELSELPHQSVVKSKALIRSSFTDLLEDANAKECELLRERWLSEECMQAIMKFLEKRK

>XP_002110753_Trichoplax_adhaerens

MEEEFKSAVDKINNLASKPSNEDLLEIYGLYKQATVGDCNTDRPGFFDQKNRAKWDSWNSKKGMSTEEAKQAYIKKANSL

>XP_027297604_ACBD7_Cricetulus_griseus

MSLQADFDQAAQDVRKLKSRPEDEELKELYGLYKQSIIGDINIACPAMLDLKGKAKWEAWSLQKGLSKEDAMSAYISKARELIEKYGI

>XP_003499494_ACBP_Cricetulus_griseus

MSQAEFDKAAEEVKRLKTQPTDEEMLFIYSHFKQATVGDVNTDRPGLLDLKGKAKWDSWNKLKGTSKESAMKTYVEKVEELKKKYGI

>XP_003506775_ACBP_5L_Cricetulus_griseus

MSQVEFEMACASLKQLKGPVSDQEKLLVYSFYKQATQGDCNIPVPPATDVRAKAKWEAWNVNKGMSKMDAMRIYIAKVEELKKNEAG

>XP_007610896_ACBD2_Cricetulus_griseus

MRASQQDFENAMNQVKLLKQDPGNQVKLKLYALYKQATDGPCNVPKPGVFDLVNKAKWEAWNSLGNLPKETARQNYVDLVSSLSPSSEASSQGKRGADEKVQESKDIVVTSEDGITKIMLNRPAKKNALTFEMYKEIILTLKNASTDNSAIAVFTGAGDYYCSGNDLTNFTSAAGGMEEAANQGAVVLREFVNSFIDFPKPLVAVVNGPAVGIAVTLLGLFDAVYASDKATFHTPFSHLGQSPEACSSYTFPKMMGSAKATEMLLFGKKLTAREAWAQGLVSEVFPDSTFEKEVWTRLRAYAKLPPNSMRISKELIRNNEKEKLHAVNAEECTTLKARWLSEECINAIMNFVSRKAKL

>XP_003786841_ACBD7_Otolemur_garnettii

MSLQADFDRAAEDVRKLKSRPNDEELKELYGLYKQAILGDINIECPAMLDLKGKAKWEAWNLQKGLSKEDAMSAYISKAKELIEKYGI

>XP_003792680_ACBP_Otolemur_garnettii

MSQAEFDKAAEEVKHLKTKPADDEMLFIYSHYKQATVGDVNTERPGMLDFKGKAKWDAWNELKGTNKEEAMKAYINKVEELKKKYGM

>XP_023375016_ACBP_5L_Otolemur_garnettii

MTQVEFELACTTLKQLKGPVSDQEKLLVYSFYKQATQGDCDIPAPPASDVRATAKYEAWSQNKGISKMDAMRIYVSKVEELKKKEAS

>XP_023368075_ACBD2_Otolemur_garnettii

MSGTAMRASQKDFENAMNQVKLLKKDPGNEVKLKLYALYKQATEGSCTMPKPGVFDLVNKAKWDAWNALGSLPKETARQNYVELVSSLTSASQPSSQVEPGADRKPSAYETLEVTSEDGITKIMFNRPTKKNAITTQMYHEIMLALKAAGKDDSSVTVLTGNGDYYSSGNDLTNFTDLPPGGPEEKAKNSAILLRDFIDCFIDFPKPLIAVVNGPAVGISVTTLGLCDAVYATDRATFHTPFSHLGQSPEGCSSYTFPKIMGPSKATEMLIFGKKLTAREACAQGLVTEVFPDSTFQKEVWTRLKAYAKLPPNAMRISKEIMRSREKEKLRAINVEECNVLQERWLSDECMNAVMNFLSRKAKL

>XP_019593859_ACBD7_Rhinolophus_sinicus

MSLQADFDRAAEDVRKLKTRPADEELKELYGLYKQSVIGDVNIECPGMLDLKGKAKWEAWNLQKGLSKEDAMSAYISKAKELIEKYGI

>XP_019570472_ACBP_Rhinolophus_sinicus

MSQAEFDKAAEAVKHLKTKPADDEMLYIYSRYKQATVGDINTERPGMLDLKGKAKWDAWNELKGTSKEDAMKAYIDKVEELKKKYGI

>XP_019595000_ACBP_5L_Rhinolophus_sinicus

MCQVEFEMACAAIKQLKGPVSDQEKLSVYSFYKQATQGDCNIPAPPATDVKAKAKWDAWNENKGMSKMDAMRNYIAKVEELKKNDAG

>XP_019607918_ACBD2_Rhinolophus_sinicus

MAGYTNDVNTKKRISAQETTNTWNIVVASSPLQVISFPALQLHVSGTAMRASQEDFENAMNQVKLLKKDPGNEVKLKLYALYKQATEGPCSIPKPGVFDLVNKAKWDAWNVLGNLPKETARQNYVDLVSSLTASSKSSSQAKPGADRERQGYENLVVTSEDGITKIMLNRPIKKNAISTQMYYEIMLALKTASKDDSTITVLTGNGDYYCSGNDLTNFTDIPAGGVEEKAKNSAIMLRDFVGCFIDFPKPLIAVVNGPAVGIAVTTLGLFDVVYASDKATFDTPFGHLGLSPEGCSSYTFPKIMGPAKAAEMLIFGKKLTAREACAQGLVTEVFPDSIFQKEVWTRLKAYSKLPPNAMKVSKQVIRNTEKEKLHAVNAEECRVLRERWVSDECINAAMNFLSRRAKL

>NP_001106768_ACBD7_Bos_taurus

MSLQADFDKAAKDVRKLKTRPDDEELKELYGLYKQSVIGDIDIECPALLDLKGKAKWEAWNLQKGLSKEDAMNAYISKAKELIEKYGI

>NP_001106792_ACBP_Bos_taurus

MSQAEFDKAAEEVKHLKTKPADEEMLFIYSHYKQATVGDINTERPGMLDFKGKAKWDAWNELKGTSKEDAMKAYIDKVEELKKKYGI

>NP_847892_ACBP_5L_Bos_taurus

MCQVEFEMACAAIKQLKGPVSDQEKLLVYSYYKQATQGDCNIPAPPATDLKAKAKWEAWNENKGMSKMDAMRIYIAKVEELKKNEAG

>AAI02907_ACBD2_Bos_taurus

MAGLAGRFARRWFSGLLGSPLQVPALGLHVRGPAMLASQKDFNNAVSQVKLLKEDPGNEVKLKLYALYKQATEGPCNVPKPGMLDFINKTKWDAWNALGSLSKEAARQNYVDLVSRLSASSESPSPEAPAADRKQPESDSLVVTSEDGITTIRLNRPAKKNALTTQMYHDIIAALQAASKDESAITVLTGGASGEEAACQCKRHKRCGFDPWIRKIP

>XP_020026865_ACBD7_Castor_canadensis

MSLQADFDRATEDVRKLKTRPEDEELKELYGLYKQAIIGDINIACPVMLDLKGKAKWEAWSLKKGLSKEDAMSAYISKAKELIEKYGI

>XP_020013492_ACBP_5L_Castor_canadensis

MCQVEFELACAAIKQLKGPVSDEEKLLVYSFYKQATQGDCNIPSPPASDVKAKAKWEAWNVNKGMSKMDAMRIYITKVEELKKKDTG

>XP_020039672_ACBP_Castor_canadensis

MSQAEFDKAAEEVKRLKTQPSDAEMLFIYSHFKQATVGDINTERPGMLDLKGKAKWDAWNQLKGTSKENAMKAYIDKVEELKGKYGI

>XP_020030578_ACBD2_Castor_canadensis

MAFVAARLVRRWCLSPLQVVGFPVVQLHTSRTMRASQKDFENAMNQVKLLKKDPGNEVKLKLYALYKQATEGPCNMPKPGMLDFINKAKWDAWNAIGSLPKETARKNYVDLVSSLSPSSESSGRVDKEPGSESVVITSEDGITKIMLNRPAQKNAITIQMYQEILSALEATSKDDNSVITVLTGNGDYYSSGNDLTNFTNLPPGGIEEVAKTGSILLRDFVDCFIDFPKPLIAVVNGPAVGIAVTLLGLFDAVYASDRATFHTPFTQLGQTAEGCSSYTFPKIMGPAKAIEMLIFGKKLTAHEACAQGLVTEVFPDSTFQKEVWARLKAYAKLPPNAMRISKELIRHNEKEKLHAVNAEESTALQGRWLSEECMNAIINFLSRKSKL

>XP_006066056_ACBD7_Bubalus_bubalis

MSLQADFDKAAKDVRKLKTRPDDEELKELYGLYKQSVIGDIDIECPALLDLNGKAKWEAWNLQKGLSKEDAMSAYISKAKELIEKYGI

>XP_006057227_ACBP_Bubalus_bubalis

MSQAEFDKAAEEVKQLKTKPADEEMLFIYSHYKQATVGDINTERPGMLDFKGKAKWDAWNELKGTSKEDAMKAYIDKVEELKKKYGI

>XP_025136708_ACBP_5L_Bubalus_bubalis

MSQVEFEMACAAIKQLKGPVSDQEKLLVYSYYKQATQGDCNIPAPPATDLKAKAKWEAWNENKGMSKMDAMRIYIAKVEELKKNEAG

>XP_025120889_ACBD2_Bubalus_bubalis

MGSPAMQASQKDFNNAVSQVKLLKEDPGNEVKLKLYALYKQATEGPCNVPKPGMLDFINKTKWDAWNALGSLSKEAARQNYVDLVSRLSASSESPSPEAPAADRKQPESDSLVVTSEDGITTIRLNRPAKKNALTTQMYHDIIAALQAASRDESAITVLTGSGDYYCSGNDLTNFRNLPADGLEEMARSGAALLRDFVNCFIDFPKPLVAVVNGPAVGISVTVLGLFDVVYATDRATFHTPFSHLGLSPEGCSSYTFPKIMGSSKAAEMLLFGKKLTAQEACAQGLVTEVFPDSTFQKEVWARLKAYSKLPPNAMRISKQIIRNREKEKLHAVNAEESSVLRERWQSDECMNAIASFLSRKAKL

>XP_008693524_ACBP_5L_Ursus_maritimus

MCQVEFEMACAAIKQLKGPVSDREKLLVYSFYKQATQGDCNIPAPPATDVKAKAKWEAWNENRGMSKMDAMRIYVAKVEELKKNDTG

>XP_008684334_ACBP_Ursus_maritimus

MLFIYSHFKQATVGDINTERPGLLDLKGKAKWDAWNQLKGTSKEDAMKAYVNKVEELKKKYGI

>XP_008690250_ACBD7_Ursus_maritimus

MPLQADFDRIAEDVRKLKTRPDDEELKELYGLYKQSVVGDINIDCPGMLDLKGKAKWEAWNLQKGQSKEDAMSAYISKAKELIEKYGI

>XP_008691072_ACBD2_Ursus_maritimus

MSKTAMGASQKDFESAVNQMKLLKKDPGNEVKLKLYAHYKQATEGPCNTPKPGVFDLINKAKWDAWNALGNLPKETARQNYVDLVSGLSSSSDSSSQVKPEADRKQAGYETLVMTSEDGITKIMLNRPTKMNALTTQMYQEIMLALEAASKDDSTITVLTGNGSYFSSGNDLTNFVISPDELEERARRGAILLRDFVGCFIDFPKPLVAVVNGPAVGISVTILRLFDLVYASDRATFHTPFSHLGQTPEACSSYTFPSLMGPAKAAEMLIFGKKLTAAEACARGLVTEVFPDSTFQKEVWTRLKAYSKLPRNALRISKQMIRNHEKEKLHAINAEESRILQERWQSDECMNAIMNFLSRKAKL

>XP_019506093_ACBP_5L_Hipposideros_armiger

MCQVEFEMACAAIKQLKGPVSDQQKLLVYSFYKQATQGDCNIPAPPATEVKAKAKWDAWNKNKGMTKMDAMRIYVAKVKELKKNDTG

>XP_019484802_ACBP_Hipposideros_armiger

MSQAEFDKAAEDVKHLKTKPADDEMLFIYSRYKQATVGDINTERPGMLDLKGKAKWDAWNELKGTSKEDAMKAYVNKVEELKKKYGI

>XP_019518964_ACBD7_Hipposideros_armiger

MSLQADFDRAAEDVRKLKARPEDEELKELYGLYKQSVIGDVDIECPGMLDLKGKAKWEAWNLQKGLSKEDAMSAYISKAKELIEKYGI

>XP_019524068_ACBD2_Hipposideros_armiger

MVVALSPLQVIGFPALQLHVSGTAMRASQEDFENAMNQVKLLKKDPGNEVKLKLYALYKQATEGPCNIPKPGVFDLINKAKWDAWNVLGSLPKETARQNYVDLVSSLSSSSKSSSQVKPGTDRERQGYENLVVTSEDSITKIMLNRPTKKNAISTQMYHEIMLALKAASKDDSTITVLTGNGDYYCSGNDLTNYTDIPPGGVEEKAKNSAIMLRDFVGCFIDFPKPLIAVVNGPAVGIAVTTLGLFDVVYASDKVSEVLET

>XP_027703748_ACBD7_Vombatus_ursinus

MSLQNDFHNAAEDVRKLKTRPNDEELKELYGLYKQSIVGDVDIECPGMLDLKGKAKWEAWNLQKGLSKEDAMSAYISKAKELMEKYGI

>XP_027710468_ACBP_Vombatus_ursinus

MSQAEFDKAAEEVKNLKSKPDDQEMLFIYSHYKQATVGDVNTDRPGMLDFKGKAKWDAWNSLKGKSKEDSMKAYIAKVEELKGKYGI

>XP_027717274_ACBP_5L_Vombatus_ursinus

MAQIEFEMACAEVKQLTGHITDEEKLVVYSYYKQATVGDINIPCPEITDFKAKAKWEAWNCRKGMSKLDAMKIYVSKVQELKNRPPWPEGGKPQLSIEDTEEED

>XP_027700905_ACBD_Vombatus_ursinus

MSQAEFDQVTEEVKNLKSKPDDQEMFFIYSHYKQATVINTVINHPGMVYFKGKAKWDAWNSLKGKSREDSMKAYFAKGEELKGKYGI

>XP_027724917_ACBD2_Vombatus_ursinus

MAVSSYVPRGMISAVTLLACRRSPIPGRLRRRQLPAINFSALLLHSSTAMRASQEEFERAKGQAKLLKEDPGNEVKLKLYALFKQAAEGPCTSPKPGMLDFVNKAKWDAWNALGSLPKDTARQNYVDLVSSLVSSQSLSQEKTSSNDKKPEYENLVVTREGNITKIMLNRPTKKNAIDVKMYNEIMLALEAAVKDDSFLTVITGNGDYYSSGNDFSKFATILPNEIEKRIEDASMLLRKFVDHFIDFPKPLVAVVNGPAVGIAVTLLPLFDIIYATDRATFQTPFTQLGLSPEACSSYTFPKIMGPAKAAEMLLFGRKLTAQEAYAQGLITEVFPDSTFQKEVWTRLKAYSQLPPKTVMASKQLIRGFEKETLHRVNFEECALISERLCSDDSINAIVNFINRKSKL

>XP_002717480_ACBD7_Oryctolagus_cuniculus

MSLQADFDKATKEVRKLKARPSDEELKALYGLYKQAIIGDVNIACPAMLDLKGKAKWEAWSLQKGLSKDDAMSAYISKANELIEKYGV

>NP_001075582_ACBP_Oryctolagus_cuniculus

MSQAEFEKAAEEVKNLKTKPADAEMLFIYSHYKQATVGDVNTERPGMLDLKGKAKWDAWNELKGTSKESAMRAYVDKVEELKQKYGI

>XP_002718938_ACBP_5L_Oryctolagus_cuniculus

MAMCQVEFELACAAVKQLKNPMSDQEKLQLYSLYKQATQGDCNIPEPPTSEVKATAKWEAWNKNKGMSKMDAMRNYVAKVEELKKTEAG

>XP_017206006_ACBD2_Oryctolagus_cuniculus

MPGAARAWPGQSLEPGPLLEGQEPKSLSHPPGKLGWKQRLDLNLALCAAGCGSPRWQLDPPCHEACTCVCVERRLPAQTLLIGQGKGRFRTWLCTSASCHLCLVCSLLTYVPGPSSRGLPRSQHTRLQASPGLTAAPSPTAMRASEKDLEKAVSQLQLLKEDPGNAVKLRLYALYKQATEGPCTMPKPSVSDFISKAKWDAWNALGSLPKETARQDYVDLVSSLSSSSESSSPVQPGASSRSPGYETLVVTSEGDITKIMFNRPTKKNAVNTQMYHELMLALKTASEDDSILTVVTGDGDYYSSGNDLTNFTDVHSGGLEEKAKSSVTLLRNFVNCFIDFPKPLVAAVNGPAVGIAVTLLGLFDVVYASDKATFHTPFSQLGLIPEGCSSYTFPKIMGTAKVTLGSVTAAEVLIFGKKLTAAEACARGLVTEVFPDSTFQREVWTRLKTYAKLPKNAMRVSKELLRNLEREKLHAVNEEECRAIQGRWMSDESLNAVASFLSRKAKL

>XP_009235926_ACBP_Pongo_abelii

MPAFAEFEKAAEEVRHLKTKPADDEMLFIYSHYKQATVGDINTERPGMLDFTGKAKWDAWNELKGTSKEDAMKAYINKVEELKKKYGI

>XP_002820594_ACBD7_Pongo_abelii

MALQADFDRAAEDVRKLKARPDDGELKELYGLYKQVIVGDINIACPGMLDLKGKAKWEAWNLKKGLSTEDAMSAYISKAKELIEKYGI

>XP_024091354_ACBP_5L_Pongo_abelii

MCQVEFELARAALKQLKGPVSEQEKLLVYGLYKQATQGDCDIPAPPASDVKARAKWEAWGANKGMSKMDAMRGYAARVEELTKKEVGGMEREQRGVQDGRHEGPRGQSEEMRKKAAG

>XP_002816422_ACBD2_Pongo_abelii

MAYLPWRLAQRWCPSSLQVTSFPAVQLHMNRTAMRASQKDFENAVNQVKLLKKDPGNEVKLKLYALYKQATEGPCNMPKPGIFDLINKAKWDAWNALGSLPKEAARQNYVDLVSSLSPSLESSSQVEPGTDRKSTGFETLVVTSEDGITKIMFNRPTKKNAINTEMYHEIMHALKAASKDDSIITVLTGNGDYYSSGNDLTNFTDIPPGGVEEKAKNNAVLLREFVDCFIDFPKPLIAVVNGPAVGISVTLLGLFDAVYACDRATFHTPFSHLGQSPEGCSSYTFPKIMSPAKATEMLIFGKKLTAGEACAQGLVTEVFPDSTFQKEVWTRLKAFAKLPPNAVRISKEVIRKREREKLHAVNAEECSVLQGRWLSDECTNAVVNFLSRKSKL

>XP_026521196_ACBP_Notechis_scutatus

MTQAEFDKAAEEVKNLKTQPSDQELLDLYAHFKQVTVGDVNTERPGMFDLKGKAKWDAWNKLKGMSKEDAIKVYIAKVNELKEKYGMQ

>XP_026537162_ACBD7_Notechis_scutatus

MTLQADFEAAAEKIKKLKSKPTDDELKELYGLYKQATVGDINIECPGMLDLKGRAKWEAWNLKKGISKDDAMKTYISKANEMIQKYGI

>XP_026532429_ACBD2_Notechis_scutatus

MQFSQEDFEKAKEQVNLLQDEPSDDIKLKLYALYKQATVGSCNIPKPSMLDFVNKAKWDAWNSLGSMTQDNARQSYIELVSSLVPAEPSPVNEIPPGSKYETLEVTTKDNITKIMLNRPKRKNAISVKMYNEIMKALEEAANDDSTITVLTGNGDYYCSGNDLSNFTQISPGGMEESAKNSAELLKKFVQHFIDFPKPLIAVVNGPAVGISVTLLGLFDIVYATDQATFHTPFSNLGQSPEGCSSYTFPKIMGLTKATEMLLFNKKLTAAEACSQGLVAEVFPDSTFQKEVWARLKAYANLPKKTLAVSKQLIRNMEKEKLYEVNSQECECLMERWLSEECMQAVMSFMQKKSKL

>XP_015681099_ACBP_Protobothrops_mucrosquamatus

MTQAEFDKAAEEVKNLKSPPSDQELLDLYGHFKQVTVGDVNTERPGMFDLKGKAKWDAWSKLKGMSKEDAIKVYIAKVNELKDKYGMQ

>XP_015666688_ACBD7_Protobothrops_mucrosquamatus

MTLQADFETVAENVKKLKSKPTDDELKELYGLYKQATVGDINTECPGMLDLKGRAKWEAWNLKKGISKEDAMKAYISKANEMIQKYGI

>XP_015670603_ACBD2_Protobothrops_mucrosquamatus

MFRAAVLKFWRPKPLRFCSPLCGISKVHGVHFPALQLHTTNSTMQFSQEDFEKAKEQVNLLQDEPSDDIKLKLYGLYKQATVGPCNIPKPSMLDFVNKAKWDAWNSLGSMTQDSARQSYIELVSSLVPAEPSPVSEIPPGSKSIYETLEVTTKDNITKIILNRPKKKNAINVKMYNEIMQALEEAANDDSVLTVLTGNGDYYCSGNDLNNFSQISPAGLEESAKNSGAMLKKFVEHFIDFPKPLIAVVNGPAVGISVTLLGLFDIVYATDRATFLTPFSNLGQSPEGCSSYTFPKIMGLTKATEMLLFNKKLTAAEACSQGLVTEVFPDSTFQKEVWARLKAYVNLPKKTLTVSKQLMRNVEKEKLYEVNSQECECLIERWLSEECMNAVMSFMQKKSNL

>XP_006137690_ACBP_Pelodiscus_sinensis

MLFIYGHFKQATVGDVNTERPGFMDFKGKAKWDAWNELKGMSKEEAMKAYIAKVEELKSKYGI

>XP_025035090_ACBP_5L_Pelodiscus_sinensis

MSQVEFEKAAALLRQIKDPISDQDKLEISSLCKQATIRDINIPCPCATDLIGKAKWEAWNAHKGMSMADAMNYIAKAEELKKKNTECKITHSCSHVPLEGLLTPRCC

>XP_006119543_ACBD7_Pelodiscus_sinensis

MTLQADFDSAAEDVKKLKTRPSDDELKELYGLYKQSTIGDIDTECPGMLDLKAKAKWEAWNLKKGLSKEDAMTAYISKAREMIEKYGI

>XP_006138875_ACBD2_Pelodiscus_sinensis

MTGAAMRVSQEEFEKAKDQLKLLKVDPGNEVKLKLYALFKQATEGPCSSPKPSMLDFVKKAKWDAWNSLGSLSKDNARQKYVDLVLSLVSSEPSSKAKDTTSESKQGYETLQVTTTDNITKIMLNRPDKKNAITTQMYREIIQAVEEAAKDDSVITVVTGNGDYYCSGNDLNNFTNIPSGGIEKMAKDGAVLLEDFVSHFIDFPKPLIAVVNGPAVGISVTLLGLFDIVYATDRATFHTPFSELGQSPEGCSSYMFPKIMGLPKANEMLLFNRKLTAREACTWGLVTEVFPDKTFEKEVWIRLKAYANLPKNSLALSKQLIRGVEKEMLHKVNCQECERLKERWLSDECMNAVLKFFEKKSKL

>XP_019387201_ACBP_5L_Crocodylus_porosus

MADTVSVHATRFEAAVRVIQSLPQNGSFQPTNEMMLKFYSFYKQATQGPCNIPRPGFWDPIGRYKWDAWSALGNMSKEEAMIAYVEEMKKVSLFFTSEITYL

>XP_019409407_ACBP_Crocodylus_porosus

MSQAEFEKAAEEVKQLKSQPTDEEMLDIYSHYKQATVGDINTQRPGMLDFKGKAKWDAWNALKGMSKEDAMKAYIAKVEELKGKYGI

>XP_019388287_ACBD7_Crocodylus_porosus

MTLQADFDSAAEEVKKLKTRPSDEELKELYGLYKQSTVGDINIECPGVLDLKGKAKWEAWNLKKGLSKEDAMNAYVSKAKEMIEKYGI

>XP_019394377_ACBD2_Crocodylus_porosus

MKSLEKYPEDKDLLQRHRSSVTQEEVASTSSKCKYETLQVSTKDNITKIVLNRPMKKNAINLVMYREIKQALEEAAKDDSVITVVTGSGDYYSSGNDLNNFTENLEQIIKNNGEWIKNFVNHFIDFPKPLVAVVNGPALGISVTLLGLFDIVYATNRNRFLTKQLTRCFPLITRTVQAVSFPFVQLHMTGATMSVSQEDFEKASAQLKLLKTDPGNEVKLKIYALFKQATEGPCSSAKPGMLDFVKKAKWDAWNSLGSLSKGDARQKYVELVSSLVSESSSQVKDTTAERKGGYETIELTTKDNITKIMLNRPEKKNAINEQMYKEIMQALEEAAKDDSVITVITGSGDYYCSGNDLAGFINIKPSEMEKMTKYGGSLLEDFVNHFIDFPKPLIAVVNGPAVGVSATILGLFDIVYATDRATFHTPFSELGQSPEGCSSYLFPKMMGLGKANEMLLFNKKLTAWEACARGLVTEVFPDQTFQKEVWRRLKTFASYPRNSLALSKQLIRNTEKEKLHAVNSQESKLLCERWQSDEFMNAIVSFFQKKAKL

>XP_019378079_ACBP_Gavialis_gangeticus

MSQAEFERAAEEVKQLKSQPTDQEMLDIYSHYKQATVGDINTQRPGMLDFKGKAKWDAWNALKGMSKEDAMKAYIAKVEELKGKYGI

>XP_019363910_ACBD7_Gavialis_gangeticus

MTLQADFDSAAEEVKKLKTRPSDEELKELYGLYKQSTVGDINIECPGVLDLKGKAKWEAWNLKKGLSKEDAMNAYISKAKEVIEKYGI

>XP_019366855_ACBP_L_Gavialis_gangeticus

MSQAEFEKVAAMVLQMKIKVTDQELLEIYSLYKQATIGNVNISCPCAVDVKGKAKWEAWNGRKGMSKEDARKNYITRVQEIKNKYGV

>XP_019377593_ACBD2_Gavialis_gangeticus

MAALTRAAWTLRLRQLRTVQAVSFPFVQLHMTGATMSVSQEDFEKASAQLKLLKTDPGNEVKLKIYALFKQATEGPCSSAKPGMLDFVKKAKWDAWNSLGSLSKGDARQKYVELVSSLVSESSRQVKDTTAEHKGGYETIELTTKDNITKIMLNRPEKKNAINEQMYKEIMQALEEAAKDDSVITVITGNGDYYCSGNDLTGFINIKPSEMEKMTKYGGSLLEDFVNHFIDFPKPLIAVVNGPAVGVSATILGLFDIVYATDRAMFHTPFSVLGQTPEGCSSYLFPKMMGLGKANEMLLFNKKLTAWEACARGLVTEVFPDQTFQKEVWRRLKTFASYPKNSLALSKQLIRNVEKEKLHAVNSQESKLLCERWQSDEFMNAIVSFFQKKAKL

>XP_006017956_ACBP_Alligator_sinensis

MLDIYSHYKQATVGDVNTQRPGMLDFKGKAKWDAWDALKGMSKEDAMKAYIAKVEELKGKYGM

>XP_006031763_ACBD7_Alligator_sinensis

MTLQADFDSAAEEVKKLKTRPSDEELKELYGLYKQSTVGDINIECPGVLDLKGKAKWEAWNLKKGLSKEDAMNAYISKAKEMIEKYGI

>XP_006033149_ACBP_L_Alligator_sinensis

MSQAEFEKVAAMVLQMKIKVTDQELLEIYSLYKQATIGDVNISCPCAVDVKGKAKWEAWNGRKGMSKEDARKNYITRVQELKNKYGA

>XP_014374759_ACBD2_Alligator_sinensis

MCGGEAQLLGTVQAVCLPFVQLHVTGATMSVSQEDFEKASGQLKLLKTDPGNEVKLKIYALFKQATEGPCSSAKPGMLDFVKKAKWDAWNSLGSLSKEDARQKYVELVSSLVSESSSQVKDTTAERKGGYETIELTTKDNITKIMLNRPEKKNAINEQMYKEIMQALEEAAKDDSVITVITGSGDYYCSGNDLTGFISIKPSEIEKMTKYGGSLLEDFVNHFIDFPKPLIAVVNGPAVGVSATILGLFDIVYATDRATFHTPFSELGQSPEGCSSYLFPKMMGLGKANEMLLFNRKLTAQEACARGLVTEVFPDQTFQKEVWRRLKTFASYPKNSLALSKQLIRNTEKEKLHAVNSQECKLLCERWQSDEFMNAIVSFFQKKAKL

>XP_026699516_ACBD7_Athene_cunicularia

MTLQADFDHAAEDVKKLKTRPTDEELKELYGFYKQATVGDINIECPGMLDLKGKAKWEAWNLKKGLSKEDAMNAYISKAKAMIEKYGI

>XP_026707905_ACBP_Athene_cunicularia

MSEAEFQKAAEEVKQLKSQPTDQEMLDVYSHYKQATVGDVNTDRPGMLEFKGKAKWDAWNALKGMSKEDAMKAYVAKVEELKGKYGI

>XP_026698040_ACBD2_Athene_cunicularia

MGICHGLCGFSTKCDSSCFFLWQVQAVCIPSIHLHMTAATMQVSQQDFEKAQEQVKLLKKDPGNETKLKLYALFKQATEGPCNSPKPGMLDFVKKAKWDAWNSLGNLSQDNARQKYTELVSSLVSAESADLKTDASPEETRHGDYETILVTTKSSITKIMFNRPEKKNAISHKMYREIMKALEEAGKDDSTIAVITGNGDYYSSGNDLNNFANIQPSEMEKMAKDGAVLLKEFVGHFIDFPKPLIAVVNGPAVGICVTLLGLCDIVYASDRATFHSPFSELGQSPEGCSSYLFPKIMGLARANEILLFNKKLTAAEACAWGLVTEVFPDSTFQKEVWARLEAYASLPKNSLAVSKQLLRGMEKEKLHAVNSKECEVLQERWLSDECMNAIVSFFQKKAKL

>XP_004605392_ACBD7_Sorex_araneus

MSLQADFDRVSADVRKLKARPSDEELKELYGLYKQSVIGDINLGCPVLLDIKSKAKWEAWNLKKGMSRADAMRAYVSKAEELIEKYGI

>XP_004608214_ACBP_Sorex_araneus

MSQAEFDKAAEEVKNLKSKPTDEEMLFIYSHYKQATVGDINTERPGMLDFKGKAKWDAWNGLKGTSKDSAMKAYIEKVEELKGRYGI

>XP_004618871_ACBD2_Sorex_araneus

MRASQQDFENAMNQVKLLKKDPGNEVKLKLYALYKQVTDGPCNTPKPGMLDFVNKAKWEAWKELGSLPQETARQNYVDLVARLSSSEAPRPARPAAEGPRAGSDAVLVTSEGAITKITLNRPSKKNAITTQMYREIMQALKAAGDDDSAITVVTGSGDYYCSGNDLTNFTDIPPGGMEEKAKDGAALLRDFVACFIDFPKPLVAVVNGPAVGISVTLLGLFDVVYATDRATFHTPFSHLGQSPEGCSSYLFPKIMGSAKAAEMLIFGKKLTAGEAFAQGLVTQVFPDSTFQKEVWARLQAYAKLPPNSMRVSKELLRRTEKEKLHAVNAEECRALQARWLSDECVNAIMGFLSQKSRL

>XP_007528808_ACBD7_Erinaceus_europaeus

MSLQADFDKISAEVRKLKARPDDEELKELYGLYKQSIIGDINIGCPVLLDMKGKIKWEAWNLKKGLSKEDAMSAYISKAKELIEKYGI

>XP_007524821_ACBP_Erinaceus_europaeus

MSQADFEKAAEEVKNLKTKPTDEEMLFIYSRYKQATVGDVNTERPGMLDFKGKAKWDSWNEIKGTSKEDAMKAYIDKVEELKKKYGV

>XP_007522269_ACBD2_Erinaceus_europaeus

MSEIVRRIVRRWYHPSLLRSSGQYTWSPALQLHTSGTAMSVTQKDFENAMNQVKLLKKDPGNEVKLKLYALYKQATEGPCNIPKPGAFDFVNKAKWDAWNGLGSLPKETARQNYVDLVSSLSGPSSESSSQVKPGADSKQEGYENLLVTSEDRITKIFLNRPTKKNAITTQMYHEIMLALKAASQDDSVLTVLTGSGDYYCSGNDLTNFTDIPPGGVEEKAKNSSFLLRDFVNCFIDFPKPLVAVVNGPAVGISVTILGLFDIVYASDRATFHTPFSHLGQSPEGCSSYTFPKIMGSSKAAEMLIFGKKLTAGEACAQGLVTQVFPDSTFQKEVWTRLKAYSKLPPNAMRISKEIIRKNEKEKLHAVNAEECNVLLSRWQSDECMNAVVNFLSRKAKL

>XP_019471930_ACBD7_Meleagris_gallopavo

MGKRQARADFDGAAEDVKKLKTRPTDEELKELYGFYKQATVGDINIECPGMLDMKGKAKWEAWNLKKGISKEDAMNAYISKAKAMIEKYGI

>XP_003207735_ACBP_Meleagris_gallopavo

MSEAAFQKAAEEVKQLKSQPTDQEMLDVYSHYKQATVGDVNTDRPGMLDFKGKAKWDAWNALKGMSKEDAMKAYVAKVEELKGKYGI

>XP_010706708_ACBD2_Meleagris_gallopavo

MQVSQKDFEKAQEQLKLLKKDPGNETKLKLYALFKQATEGPCNSPKPGMLDFVKKAKWDAWNSLGSLSQENARQEYTKLVSSLISAESAGEKKDTSPKESSHGGYETLIVTTDSNITKIMFNRPDKKNAINHKMYREIINALQEAAKDDSTIAVVTGNGEYYTSGNDLNNFSNVQPSEMKKAAKDGAELLKEFVGSFIDFPKPLIAVVNGPAIGISVTLLGLFDVVYASDKATFHTPFSQLGQSPEGCSSYLFPKIMGSAKANEMLLFNKKLTAAEACALGLVNEVFPDSTFQKEVWARLKAYASLPKNSLAVSKQLLRNMEKEKLHAVNSQECEVLTERWLSDECLNALVTFFQRKSKL

>NP_001232582_ACBD7_Taeniopygia_guttata

MTLQADFDSAAEDVKKLKTRPTDEELKELYGFYKQATVGDINIECPGMLDLKGKAKWEAWNLKKGLSKEDAMNAYISKARAMVEKYGI

>XP_012430448_ACBP_Taeniopygia_guttata

MSEAAFQKAAEEVKQLKSQPTDQEMLAVYSHYKQATVGDVNTERPGMLDFKGKAKWDAWSALKGMSKEDAMKAYIAKVEELKGKYGI

>XP_004175572_ACBD2_Taeniopygia_guttata

MATPALTFVRRPCWRLRCGARPTQAARVPVIDLHMTAATMQVSQEDFQKAQEQLKLLKKDPGNETKLKLYALFKQATEGPCKAPKPGMLDFVKKAKWDAWNSLGNLSQDEARQKYAELISSLVSAESASQKKEASPEEGRHDGYETLIVTTKNNITKIMFNRPDRKNAINHQMYREIIKALEEAGKNDSTIAVITGNGDYYSSGNDLSNFTNVQPGEMEKMAKDGAVLLKDFVGHFIDFPKPLIAVVNGPAIGICVTVLALCDLVYASDRATFHSPFSQLGQSPEGCSSYLFPKIMGLAKASEMLLFNKKLTAAEACAQGLVTEVFPDRSFQKEVWARLEAYASLPKNSLAVSKQLLRSMEKEKLHAVNSRECEVLMGEVVI

>XP_027316899_ACBP_Anas_platyrhynchos

MAEAAFQKAAEEVKQLKSQPSDQEMLDVYSHYKQATVGDVNTDRPGMLDFKGKAKWDAWNALKGMSKEDAMKAYVAKVEELKGKYGI

>XP_005017941_ACBP_Anas_platyrhynchos

MTLQADFDEAAEEVKKLKTRPTDEELKELYGFYKQATVGDINIECPGMLDLKGKAKWEAWNLKKGISKEDAMNAYISKAKTMVEKYGI

>XP_005010768_ACBD2_Anas_platyrhynchos

MFGLTCSCPEKNRNKLGSTNCVEPSRIQEFTVDIKPRQAQAVCIPAVHLHMTTATMQVSQKDFEKAQEQLKLLKKDPGNETKLKLYALFKQATEGPCNSPKPGMLDFVKKAKWDAWNSLGNLSQDNARQKYTELVSSLISAESAGQKKDASPEESRHGGYETIIVTTKNNITKIMFNRPERKNAINHQMYREIIKALHEAGKDDSTIAVITGNGEYYTSGNDLNNFANVKPNEMEKMAKDGAVLLKEFVSSFIDFPKPLIAVVNGPAIGISVTLLGLCDIVYASDRATFHTPFSQLGQSPEGCSSYLFPKIMGLAKANEMLLFNKKLTAAQACAWGLVTEVFPDGTFQKEVWERLKAYASLPKNSLAVSKQLLRSMEKEKLHAVNSKECEVLKERWLSDECLNAIVSFFQRKSKL

>XP_009557368_ACBD7_Cuculus_canorus

MTLQADFDSAAEDVKKLKTRPTDEELKELYGFYKQATVGDINIECPGMLDLKGKAKWEAWNLKKGLSKEDAMNAYISKAKAMVEKYGI

>KFO71608_ACBP_Cuculus_canorus

AAFQKAAEEVKQLKSQPTDQEMLDVYSHYKQATVGDVNTDRPGMLDFKGKAKWDAWNALKGMSKEDAMKAYIAKVEELKGKYGI

>XP_009562413_ACBD2_Cuculus_canorus

MEFRRAQAVCIPAIHLHVTAATMQASQEDFEKAQEKLKLLKNDPGNETKLKLYALFKQATEGPCNSPKPSMLDFVKKAKWDAWNSLGDLSQDNARQKYTELVSSLISAESADQKKDDSPDEGRHDGYETILVTTKNNITKIMFNRPDKKNAINHKMYGEIMKALEEAGKDDSTIAVITGNGDYYSSGNDLNNFASALAGEAEKMAKDGAILLKDFVDHFIDFPKPLIAVVNGPAVGISVTLLGLCDVVYASDSATFHTPFSQLGQSAEGCSSYTFPKIMGLAKASEILLFNKKLTAAEACAKGLVTEVFPQRTFQKEVWKRLEAYASLPKNSLAVTKQLMRSMEKEKLHAVNSKECEVLQGRWLSEECVNAVVSFFSQRSKL

>XP_010883060_ACBD7_Esox_lucius

MSLKAEFELVATDVKKVKTRPSDQELLDIYGLYKQAIVGDINIDKPGLLDMKAKAKWEAWDSRTGMSKEDAMTAYITLAKEIINKYGM

>XP_010878526_ACBP_Esox_lucius

MSETDFDKAAEEVKNLKAKPADAEMLRVYALFKQAKVGDINTARPGMLDFTGKAKWDAWEKEKGKSQEAARKEYIDFVEELKGKYGI

>XP_019897597_ACBP_L_Esox_lucius

MSMVEFHRAAEELKGLPVLPTYAELAVVYGLYKQVTIGDVNIERPGIFDFQGKSKWDGWKAQEGKPKEDAMKEYIAYVENLKTKYLM

>XP_012994963_ACBD2_Esox_lucius

MALALRIFRPCGFVRFTRAVQVSHITTLKLHTTGSAMGATVEQFNHAKGQMGKLKNDPGNEVKLKIYALFKQATQGPCNTPKPGMLDFVGKVKWEAWKSLGSVSQEEARQQYVDLIDSLLAAEGPAVATQPTAGSSPFNTLLVSTKDNITTICFNRPEKKNAITVEMYNEIIKALEQAGKDESVITVITGSGDFFCSGNDLTNFTKIPEGGISKMAKDAGELLRAYVQAFIDFPKPLIAVVNGPAVGVSVTLLGLFDIIYATEKATFHTPFSQLGQSPEGCSSYTFPKMMGNAKASEVLLFNKKLTATQACAQGLVTEVFPDSSFQTEVATRLKAYAKLPRNSLALSKQLVRGTEKERLYKVNDQEVERLVERWQSDECMQAIMGFFQSKAKL

>XP_026865338_ACBD7_Electrophorus_electricus

MPLQAEFEQYAEDVKKVKTRPSDQELLDLYGLYKQAIFGDINIDRPGMLDMKGKAKWEAWDSRKGMSKEDAMTAYIALAKGTIDKYGM

>XP_026866041_ACBP_Electrophorus_electricus

MTEEAFQKAAEEVKQLKAKPTDADMLEVYALYKQATVGDVNIDRPGLFDFTGKSKWDAWKGKEGTSKEDAMKAYISKVEELKEKHGI

>XP_026869094_ACBD2_Electrophorus_electricus

MAASCIKVISPWRLFRASQVFRIPGVQLHTTGALMGASVEDFNNAKAQLGTLKKDPGNHVKLKIYALFKQATQGPCNTPKPGMLDFVNKAKWDAWKSLESVSQEEARQQYVDLIKSLVSAEGPAVATPPPESAQAFQSLLVTTEDGITTIRFNRPEKKNAITLDMYKELIDALHHAGKDDSVITVMTGSGDFYCSGNDLNNFTGIPQGGIEKMAKDSGELLKHYVKAYIDFPKPLIAVVNGPALGVSVTVLGLFDVVYATESATFHTPFSQLGQSPEGCSSYTFPKIMGNAKASEMLLFNKKVTATEACNLGLVTEVFPDGSFQSEVWTRLKAYAKLPPKSLALSKQLIRSKEKEKLHAVNDAEVERLIERWLSDECMQAIMNFFQAKPKL

>NP_001134589_ACBD7_Salmo_salar

MSLQAEFERVADDVKKVKSRPSDQELLDMYGLYKQAIFGDINIDKPGMLDMKGKAKWEAWDSRKGMSKEDAMTAYIALAKEIISKY

>XP_014005791_ACBPL_Salmo_salar

MSMAEFEKAADEVRRLTVQPSYAELAVVYGLYKQATLGNVNTKRPGIFDFQGKSKWDGWKAQEGKSKEDAIKEYIAFVEEMKAKYPM

>XP_014020239_ACBP_Salmo_salar

MSEADFDKAAEEVKQLKAKPADAEMLRVYALFKQAKVGDVNTARPGMLDFTGKAKWDAWEKEKGKSQEDARKEYIALVEELKGKYGV

>XP_014036105_ACBD2_Salmo_salar

MAVALRKISPWRFVRAVQVAHIPTLKLHMTGSMMGATVEQFNHAKSQMGTLKEDPGNEAKLKIYALFKQATQGPCNTPKPGMLDFVGKAKWDAWKSLGSVSQEDARQQYVDLIDSLLAAEGPAVAAQPTGSAATFETLLVSTEDNITTICLNRPQKKNAITVEMYNEVIKALEQAGKDDSVITVITGSGDFYCSGNDLTNFTKIPEGGIEQMAKNAGELLREFVKAFIDFPKPLIAVINGPAVGVSVTLLGLFEIVYATERATFHTPFSQLGQSPEGCSSYTFPKIMGNAKASEMLLFNKKLTAVQACAQGLVTEVFPDSSFQTEVATRLKAYAKLPRNSLALSKQLIRGTEKERLHTVNDQEVERLVERWLSDECMQAIMSFFQAKAKL

>XP_020565857_ACBD7_Oryzias_latipes

MSLEADFKTVAEDVKKVKTRPSNDELLTLYGLYKQSLVGDINIDKPGALDATGRAKWESWNSRKGMSKDDAMSTYITNAKEIISKYGM

>NP_001158334_ACBP_Oryzias_latipes

MAELQAQFDAAAAEVKQLKAKPTDEEMLQVYALFKQASVGDVNTARPGMLDFTGKAKWDAWEKQKGKSKEDAMKEYVKLVEELKQKYGI

>XP_023821390_Oryzias_latipes

MRAELQATEAGKKIQVRRRVGRVLLISLSASMAEAFEKAAEEVKVLKEKPNQSEMTDLYGLYKQATKGDNDTERPGFLDFVGKTKWDAWSLKKGLSKEKAMAEYVELVEKLKLKYGI

>XP_023805791_ACBD2_Oryzias_latipes

MAGFALRSSAAWRSVRLSSLLRTSVSILGLHTAASPLMGATVEQFEEAKSRLSTLKKDPGNEVKLKIYALFKQATQGPCNAPKPGMLDFVNKAKWDAWKSLGSISQDEAREQYCDLIGSLLTAEGQSAAQVAAKPTGGGAAYETLLVTTEDGITTIKLNRPAKKNAITTEMYNEIIAALDQAAKDDCVITVFTGAGDFYCSGNDLTNFTKIPEDGVEAMAQSAGDLLRRYVKAYIDFPKPLVAVVNGPAVGVSVTLLGLFDLVYATERATFHTPFSQLGQSAEGCSSYTFPKMMGNAKASEMLLFNKKLTAAQACSLGLVTEVFPDSSFQSEVWSRLKAYAQLPRKSLALSKQLIRSIEKEHLYAVNDAEVERLMERWTSDECFNAIMSFFQAKAKL

>XP_011604480_ACBP_Takifugu_rubripes

MSEEAFQKAAEEVKVLKNKPSDSELGELYGLYKQVTVGDVNTDRPGMFDFTGKAKWDAWNAKKGVSKEDAMAAYVALVEKLKAKEGV

>XP_003966134_ACBD7_Takifugu_rubripes

MSKQAEFEKIAEDVKKVKTRPTDQELLDLYGLYKQAIVGDVNTDRPGLLDLKGKAKWDAWESRKGMSQDEAMSDYINLGNEVISKYGI

>XP_011606473_ACBD2_Takifugu_rubripes

MALKLGSWWRLVKLSRAPCLTFHTTASSMMGVTMEQFEQAKSKLSHLKKEPGNDVKLKIYALFKQATQGPCDTPKPSMLDFVNKAKWDSWKSLGSTTPEEARQQYCDLIGSLLAAEGGDSTPASPADSSYETLLVSREDNVTTITLNRPLKKNAITNQMYNEIIAALEEAAKDESAITVFTGAGDFYCSGNDLTNFTKIPEDGIQEMAKRGGELLRKYVKAYIDFPKPLVAVVNGPAVGISVTVLGLFDLVYATDRATFHTPFSQLGQTAEGCSSYTFPKIMGPAKANEMLLFNKKLTAAQACDLGLVTEVFPDSSFQAEVWSRLKGYGKLPPNCLLFSKRLIRSTETERLHAVNDAEVERLVERWTSDECFNAIMSFFQNKSKL

>XP_017308942_ACBD7_Ictalurus_punctatus

MLQAEFDKIAADVKQVKSRPSDQELLDLYGLYKQVIAGDINIDAPGMLDVKGKAKWDAWNSRKGMSTEDAMTAYITMAKEVINKYGM

>NP_001187742_ACBP_Ictalurus_punctatus

MTEEAFQKAAEEVKQLKAKPADAEMLEIYSLFKQATVGDVNTARPGMLDFTGKAKWDAWDAKKGMTKEDAMKAYISKVEELKGKYGI

>XP_017335957_ACBD2_Ictalurus_punctatus

MAASVEDFNQAKVQLGTLKKDPGNELKLKIYALFKQATQGPCNTPKPGMLDFVNKAKWDAWKSLGALPQDDARQQYVDLISSLVAAEGPPAAEASPTGSEKAFQTLQVTTEDGITTIRLNRPQKKNAITVEMYNELMEALDLAGKNDSVITVLTGSGDYYCSGNDLNNFSKIPEGGVEKMAKDSGELLRRYVKAYIDFPKPLAAVVNGPAVGISVTVLGLFDIVYATECATFHTPFSNLGQSPEGCSSYTFPKIMGAAKASEMLLFNKKLTATQACELGLVTEVFPDSTFQSEVWTRLKAYSKLPPNSLALSKQLMRQVEKDKLHAVNDAEVERLVERWLSDECMQAIMSFFQGKAKL

>XP_020375563_ACBP_Rhincodon_typus

MAQGDFERAAEEVKQLKSQPSDDEMLTVYSLYKQATVGDVNTDRPGIFDLKGKAKWDAWNARKGTSKEEAMAMYISKVKELKEKYGMN

>XP_020386602_ACBD2_Rhincodon_typus

MIATEAEFEKAKNKLKTLKNDPGNEVKLQIYALFKQATQGPCDSPKPSMLDFVNKAKWDAWNSLGKTPKEEARQKYVDLIETLVSAEIPNQSEATTTRDVKSSFQTINVTSENNITTILLNRPAKKNAISVLMYEEIMKALEQAAKDDSTLTVMTGNGDYYCSGNDLSNFTDIGPEGIEQKSKNSGELLKRYVSHYIDFPKPLIGVINGPAVGVAVTVLGLFDAVYATDKATFHAPFSKLGQSPEGCSSYTFPKIMGTSKANEILLFNKKLTAAQACELGLVTEVFPDSTFQKEVWKKLHAYAKLPKNSLAFSKQLIRDVEKEKLHAVNAQECERLVERWLSEECMNAIMGFFAKKSKL

>XP_023961681_ACBP_5L_Chrysemys_picta

MSQAEFEKVAAMVREMKIPISEQEKLEIYSLYKQATIGDINIPCPCATDVTGKAKWEAWNGRKGMSKANAMKNYIAKAEELKKKYGA

>XP_005279908_ACBP_Chrysemys_picta

MSQAEFDKAAEEVKQLKSQPTDEEMLYIYSHFKQATVGDINTERPGFLDFKGKAKWDAWNALKGMAKEEAMKAYIAKVEELKGKYGI

>XP_008175866_ACBP_Chrysemys_picta

MTLQADFDSAAEDVKKLKTRPSDDELKELYGLYKQSTVGDIDTECPGMLDLKAKAKWEAWNLKKGLSKEDAMTAYISKAREMIEKYGI

>XP_005306038_ACBD2_Chrysemys_picta

MNRTVHAVRFPVIQLHTTGATMRVSQEVFEKAKDQLKLLKEDPGNEVKLKLYALFKQATEGPCSSPKPGMLDFVKKAKWDAWSSLGSLSKDNARQKYVDLVSSLVSSESSSQVKDTTPDSKHGYETLQVTTTDNITKIMLNRPEKKNAITTQMYREIIQALEEAAKDDSVITVITGNGDYYCSGNDLNNFTNIPAGGIEKMAKDGAVLLENFVKHFIDFPKPLIAVVNGPAVGISVTLLGLFDIVYATDRATFHTPFSELGQSPEGCSSYMFPKIMGLSKANEMLLFNKKLTAGEAYAQGLVTEVFPDWTFEKEVWIRLKAYANLPKNSLALSKQLIRGVEKEKLHAVNCQECELLRERWLSDECMNAIMSFFGKKSKL
