# Supplementary material for: The diversity of ACBD proteins – From lipid binding to protein modulators and organelle tethers
Source: Biochim Biophys Acta Mol Cell Res. 2020 May;1867(5):118675. doi: 10.1016/j.bbamcr.2020.118675 (PMC7057175; doi:10.1016/j.bbamcr.2020.118675)
Supplement: Supplementary material 3 — Sequences used for Fig. S4. [file mmc8.docx]

>sp|P07108|ACBP_HUMAN

MSQAEFEKAAEEVRHLKTKPSDEEMLFIYGHYKQATVGDINTERPGMLDFTGKAKWDAWNELKGTSKEDAMKAYINKVEELKKKYGI

>sp|Q8N6N7|ACBD7_HUMAN

MALQADFDRAAEDVRKLKARPDDGELKELYGLYKQAIVGDINIACPGMLDLKGKAKWEAWNLKKGLSTEDATSAYISKAKELIEKYGI

>EAW90652_ACBP-L5_Homo_sapiens

MGDAGATAAALRPAHNLRPAPPTASAAHAQSSRTSAPSAQRRLPAEPSHQPSGTRTSLTRPLRTVPCPAPLPGSQRVGLMIPRPRIHPHPLLAPGTASTTPCAKWSSSCAALKQLKGPVSDQEKLLVYGLYKQATQGDCDIPGPPASDVRARAKWEAWSAKKGASKMDAMRGYAAKVEELTKKEVGGVEREQRGVQDGRHEGLRGQSGGADEEGRASKMDAMRGYAARVRR

>sp|O75521|ECI2_HUMAN

MAMAYLAWRLARRSCPSSLQVTSFPVVQLHMNRTAMRASQKDFENSMNQVKLLKKDPGNEVKLKLYALYKQATEGPCNMPKPGVFDLINKAKWDAWNALGSLPKEAARQNYVDLVSSLSPSLESSSQVEPGTDRKSTGFETLVVTSEDGITKIMFNRPKKKNAINTEMYHEIMRALKAASKDDSIITVLTGNGDYYSSGNDLTNFTDIPPGGVEEKAKNNAVLLREFVGCFIDFPKPLIAVVNGPAVGISVTLLGLFDAVYASDRATFHTPFSHLGQSPEGCSSYTFPKIMSPAKATEMLIFGKKLTAGEACAQGLVTEVFPDSTFQKEVWTRLKAFAKLPPNALRISKEVIRKREREKLHAVNAEECNVLQGRWLSDECTNAVVNFLSRKSKL

>XP_005597701_ACBP-L5_Equus_caballus

MCQVEFELACAAIKQLKGPVSDQEKLLVYSFYKQATQGDCNIPAPPATDVKAKAKWDAWNDKKGISKMDAMRIYVAKVEELKKNDTG

>XP_001487927_ACBP_Equus_caballus

MSQAEFDRAAEEVKNLKTKPADDEMLFIYSHYKQATVGDVNTERPGMLDLKGKAKWDAWNALKGTSKEDAMKAYINKVEDLKKKYGI

>XP_003364405_ACBD7_Equus_caballus

MSLQAEFDRVAEDVRKLKRRPDDAELKELYGLYKQSVIGDIDIECPAMLDLKGKAKWEAWNLQKGLSKEDAMSAYVSKARELIEKYGI

>XP_005603601_ECI2_Equus_caballus

MAGAAVRLARRWAASAVRSPVQDARFPALKLHVGGTAMRASQKDLENAVNQMKLLKKDPGNEVKLKLYALYKQATEGPCNMPKPGIYDLIRKAKWEAWNTLGNLPKETARQNYVDLVSSLCSSSESSSQGKPGADREQLRYETLVVTSEDGITKIMLNRPSKKNAVNLQMYQEIVLALKAASQDDSTITVLTGNGDYYSSGNDLTNFTNTSPGEIQEKAKNSLILVREFVGCFIDFPKPLIAVVNGPAVGIAVTLLGLFDIVYASDRATFHTPFSRLGLHPEGCSSYTFPKIMGPTKATEMLIFGKKLTAEEACAQGLVTEVFPDRTFQKEVWTRLKAYAKLPSNVLRISKQVIRNREKEKLHSVNVEENSVLQGTVLSDEAVNAVMNFLSKRAKL

>XP_005505169_ACBP_Columba_livia

MSEAAFQKAAEEVKQLKSQPTDQEMLDVYSHYKQATVGDVNTDRPGMLDFKGKAKWDAWNALKGMSKEDAMKAYIAKVEELKGKYGI

>XP_005505011_ACBD7_Columba_livia

MALQADFDHAAGDVRKLKTTPTDEELKELYGLYKQATIGDINIECPGMLDLKGKAKWEAWNLKKGLSKEDAMKAYISKANALVEKYGI

>XP_005506757_ECI2_Columba_livia

MTAATMQVSQKDFEKAQEQLKLLKNDPGNETKLKLYALFKQATEGPCNSPKPGMLDFVKKAKWDAWNSLGNLSQDDAREKYTELVSSLVSAESAGQHKDASPDENRLGGYETIIVTTKNNITKIMFNRPDKKNAINHKMYREIIKALEEAGKDDSTIAVITGNGDYYSSGNDLSNFTGVKPTEMKKMAEDGAVLLKEFVSHFIDFPKPLIAVVNGPAVGICVTLLGLCDIVYASDRATFHCPFSQLGQSPEGCSSYLFPKIMGLAKANEVLLFNKKLTAADACVQGLVTEVFPDRTFQKEVWARLEAYASLPKNSLTLSKQLIRSIEKEKLHAVSSKECEVLKERWLSDECINAVVTFFQKKSKL

>XP_005230860_ACBP_Falco_peregrinus

MTEAAFQKAAEEVKQLKSQPTDQEMLDVYSHYKQATVGDVNTDRPGMLDFKGKAKWDAWNALKGMSKEDAMKAYIAKVEELKGKYGI

>XP_005228918_ACBD7_Falco_peregrinus

MALQADFDGAAEDVKKLKTRPTDEELKELYGFYKQATVGDINIECPGMLDLKGKAKWEAWNLKKGLSKEDAMNAYISQAKAMVEKYGI

>XP_005239124_ECI2_Falco_peregrinus

MTAATMQVSQKDFEKAQEQLKLLKKDPGNETKLKLYALFKQATEGPCNAPKPGMLDFVKKAKWDAWNSLGNLSQDNARQKHTELVSSLVSAESAGQKKDASPEESGHDGYETILVTTKNNITKIMFNRPDRKNAINHKMYREIIKALEEAGKDDSTIAVITGNGDYYSSGNDLNNFTNIQPGEMEKMAKDGAVLLKEFVGHFIDFPKPLIAVVNGPAIGICVTVLGLCDVVYASDRATFHSPFSQLGQSPEGCSSYLFPKIMGSAKANEILLFNKKLTAAEACAQGLVTEVFPDSTFQKEVWAKLEAYASLPKNSLAVSKQLLRSVEKEKLHAVNSKECEVLKERWLSDECVNAIVSFFQKKSKL

>jgi|Capca1|176715|Capitella_teleta

MSEAFNKAAEDVKVLKSTPSNEELLELYGLFKQATVGDVNTDRPGMFDLKGKSKWDSWKGREGLSKADAEAKYIETANGLIAKYGKA

>jgi|Capca1|176947|Capitella_teleta

MTVNMVMFRALSRSTAFVQQGMASVGRRMIRTSTASFFPAHDAEFSAAKERLNTLSEDPGNMTKLKIYALFKQATVGKCNTQRPGMVDFVGKAKWDAWDSLGDISQDEAQLKYISLIDELVAKDPSSSASEAESSTATSGNILVEVKDKMQIITLNRPNKRNALTLEMYDEIADVLESTGKDKNITLTVFTGSGSYYCSGNDLANFLNVDPADLSAMAVNGGKVLQRFVGSFIDHPRPLCAVINGPAVGITVTLLGLFDAVYATDKATFHTPFSLLGQSPEGCSSYVFPKLMGNKASEMLLFNKKLTAQEAFECNLVSEVYPDHAFQSEIWKKLAEIAQLPPNSLKTSKNLIRDAEREILHATNERECQLLVDRWQSEECAQAVMNFFQGK

>NP_031856_ACBP_MOUSE

MSQAEFDKAAEEVKRLKTQPTDEEMLFIYSHFKQATVGDVNTDRPGLLDLKGKAKWDSWNKLKGTSKESAMKTYVEKVDELKKKYGI

>NP_067269_ACBP-like5_MOUSE

MSQVEFEMACASLKQLKGPVSDQEKLLVYSFYKQATQGDCNIPVPPATDVRAKAKYEAWMVNKGMSKMDAMRIYIAKVEELKKKEPC

>sp|Q9D258|ACBD7_MOUSE

MSLQADFDQAAQDVRKLKSRPEDEELKELYGLYKQSVIGDINIACPAMLDLKGKAKCEAWNLQKGLSKEDAMCAYISKARELIEKYGI

>NP_001103801_ECI2_MOUSE

MAAVTWSRARCWCPSVLQVFRLQVAKLHLGRPTMRASQQDFENALNQVKLLKKDPGNEVKLRLYALYKQATEGPCNMPKPGMLDFVNKAKWDAWNALGSLPKETARQNYVDLVSSLSSSSEAPSQGKRGADEKARESKDILVTSEDGITKITFNRPTKKNAISFQMYRDIILALKNASTDNTVMAVFTGTGDYYCSGNDLTNFTSATGGIEEAASNGAVLLRDFVNSFIDFPKPLVAVVNGPAVGISVTLLGLFDAVFASDRATFHTPFSQLGQSPEACSSYTFPKMMGSAKAAEMLLFGKKLTAREAWAQGLVTEVFPESTFETEVWTRLKTYAKLPPNAMRISKELIRKNEKEKLYAVNAEECTTLQARWLSEECMNAIMSFVSRKPKL

>XP_003416725_ACBP_Loxodonta_africana

MSQAEFDKAAEEVKHLKTKPDDNEMLFIYSRYKQATVGDVNTERPGMLDFKGKAKWDAWNELKGTSKEDAMKAYVDKVEELKKKYGI

>XP_003410775_ACBD7_Loxodonta_africana

MTLCIKQLTPPRKTFGKADFDRAAEDVRKLKARPDDENLKELYGLYKQSVIGDIDIERPGTLDLKGKAKWEAWNLQKGLLKEDAMRAYISKAKELIEKDGI

>XP_003417017_ACBP-L5_Loxodonta africana

MGQVEFELACAAVKELKGPVSDQEKLLVYSFYKQATQGDCNIPAPPSTDVKAKAKWEAWNENKGMTKLDAMRIYIAKVEELKKKEGG

>XP_003417885_ECI2_Loxodonta_africana

MEYVKAAYRNSQRETDHPETTPRPSPTCCSASSASHQSEPRGTGFGSLPLGFALPTLASSQSAISRQGPAPVSGPAESRMVPCVARFASARQSRANSLHLQLAMAWVVARLAWRCPPRYAGSPLQVSSFLVQQMHMSQTAMRASHKDFETAVNQMKLLKKDPGNEMKLKLYALYKQATEGPCNMPKPGVLDLISKAKWDSWNALGSLPKETARQNYVELVSSLSSSESSSQGKPGADSKQPGYETLVVTSEDGITKIMLNRPTKKNAITTQMYQDIVLALEAAGKDDSTITVLTGNGDYYCSGNDLNNFTDIPPGGIEEQAKNNAILLRNFVGCFIDFPKPLVAAVNGPAVGISVTLLGLFDIVYASDRATFHTPFTHLGQSPEGCSSYTFPKIMGPAKAAEMLIFGKKLTAREACAQGLVTEVFPDSTFQKEVWTRLKAYSKLPPNAMRTSKQLIRNVEREKLHATNSEEISALQGRWRSEECTNAVMNFLSRKVKL

>sp|Q9TQX6_ACBP_Canis_lupus

MSQAEFDKAAEDVKHLKTKPADDEMLYIYSHYKQATVGDINTERPGLLDLRGKAKWDAWNQLKGTSKEDAMKAYVNKVEDLKKKYGI

>XP_850165_ACBP-like_Canis_lupus

MSTVTKALSLWAGPVNMMGYHSQDFIILYGKVPKPHNPASMSQAEFDKATEDVKHLETKPADDEMLFIYSHYKQATIGDVNTEWPGLLDLRGKAKWDAWNHLKRTSKENAMKAYINKVEDLKKKYGM

>XP_848899_ACBD7_Canis_lupus

MSLQADFNKIAEDVRKLKARPDDEELKELYGLYKQSVVGDINIECPGMLDLKGKAKWEAWNLQKGLSKEDAMSAYIPKAKELIEKYGI

>XP_537760_ACBP-L5_Canis_lupus

MCQVEFEMACAAIKQLKGPVSDQEKLLVYSFYKQATQGDCNIPAPPATDVKAKAKWEAWNQNKGMSKMDAMRIYVAKVEELKKKDTG

>XP_535873_ECI2_Canis_lupus

MQALSLHMSKTAMGASQKDFESAMNQVKLLKKDPGNEVKLKLYALYKQTTEGPCNTPRPGVFDLINKAKWDAWNALGNLPKETARQNYVDLVSDLSSSDSSSQVKPEADRKQPGYETLVVTSEDSITKIMMNRPAKKNALTIQMYREIMLALEAASKDDSTIIVLTGNGDYYSSGNDLMNFMNIPPGEMEKEAKNGAILLRDFVGCFIDFPKPLVAVINGPAIGISVTILGLFDLVYASDRATFHTPFTHLGQSPEGCSSYTFPKIMGQAKAAEMLMFGKKLTAREACAQGLVTEVFPDSTFQKEVWTRLKAYSKLPRNTLHISKQSIRNLEKEKLHAVNAEENSVLQERWLSDECINAVMSFLSRKAKL

>XP_003763843_ACBP_Sarcophilus_harrisii

MSQAEFERAAEEVKNLKAKPNDEEMLFIYSHYKQATVGDINTERPGMMDFRGKAKWDSWNSLKGKSKEEAMKAYIAKVEELKKKYGI

>XP_003771952_ACBD7_Sarcophilus_harrisii

MSLQNDFHNAAEDVRKLKTRPNDEELKDLYGLYKQSIVGDIDIECPGMLDLKGKAKWEAWNLQKGLSKEDAMSAYISKAKELIEKYGI

>XP_003770143_ACBP-L_Sarcophilus_harrisii

MAQVEFELACAAVKQLTGPVTDEEKLVVYSYYKQATIGDVNIPCPEVTDFKAKAKWEAWNCRKGMSKLDAMRVYVSKVEELKSRQC

>XP_003760261_ECI2_Sarcophilus_harrisii

MPAATLRACSRPPLPGSLWKRQLPAVGFPALQLHMTNMALRASQEDFERAKGQIKLLKEDPGNEVKLKLYALFKQATEGPCTSPKPGMLDFVNKAKWDAWNALGSLSKDTARQNYVDLVASLVSSQSLSQETSIDKKSEYETLVVTREDNITKIMLNRPAKKNAINNKMYNEIMLALEAADKDDSSLTVLTGNGDYYSSGNDLSEAAKVPPDDIEKKIKESFVLLRTFVDHFIDFSKPLVAVVNGPAVGISVTLLGLCDIVYATDRATFHTPFIQLGQSPEACSSYTFPKIMGPIKAAEMLIFGRKLTAQEAYAQGLITEVFPDSTFQREVWTRLKAYAQLPPKAMMFSKQLIRSFEKETLHKVNFEECTLLCERWVSDEFMNAVVNFINRKSKL

>XP_001367923_ACBP_Monodelphis_domestica

MSQAEFDKAAAEVKVLKSKPNDDEMLYIYSHFKQATVGDVNTARPGITDFKGKAKWDAWNSLKGKSKEDAIKAYIEKVEELKKKYGI

>XP_001370361_ACBP-L5_Monodelphis_domestica

MAQVEFELACATVKQLTGPVSDEEKLLVYSYYKQATVGDINIPCPEVTDFKAKAKWEAWNCRKGMSKLDAMRIYVSKVEELKKKQSS

>XP_001368082_ECI2_Monodelphis_domestica

MISAISQLARWRPPIPGSLWKRQLPAVSFPALQLHTTSMAMRVSQEDFEKAKEQVSLLKKDPGNEVKLKLYALFKQATEGPCTTPKPGMLDFVKKAKWDAWNALGSLPKDAARQNYVDLVSSLVSSESLSQKKTSFNDTQSEYKTLIVTKEDNITKIMLNRPSQKNAINIQMYKDITLALEAAEKDDSSLTVITGNGEYYSSGNDLSEPLKIPPDEIQKKLEENMQILRTFIDHFIDFSKPLVALVNGPAIGISVTLLGLFDIVYATDRATFHTPFIHLGLCPEACSSFTFPKIMGSVKAAEILIFGRKLTAQEAYARGLVTEVFPESTFQKEVWTRLKAYSQLPPVSMKKSKQLIRSFEKETLHRVNLEECNLLQTRFTSDECLNAIVNFLNRKSKL

>XP_003222139_ACBD7_Anolis_carolinensis

MERCKWKEARPGCKCSIKIVASLILLIIFLSHQADFDSVAEKVKKLKTRPTDDELKELYGLYKQVTVGDINIESPGALDLKGKAKWESWNQKKGMSKEDAMKAYISKANGLIQKYGL

>XP_003227602_ACBP_Anolis_carolinensis

MTSMPAFRVQLFQPPLGTATTQAEFDKAAEEVKKLKTQPADEEMLFIYSHFKQATVGDVNTERPGMLDFKGKAKWDAWNGLKEASERYQVSAVLSQVLRQGQE

>XP_003223610_ECI2_Anolis_carolinensis

MQFSQEDFEKAKDQLKLLEDDPGNEVKLKLYALYKQATEGPCKTPKPGMLDFVKKAKWDAWSSLGSLPQDSARQKYIELVSSLVSADSSMASGSKSDYQTLQVTTKDNITKIVLNRPKKKNAISTKMYNEIIEALEEAAKDDSVITVVTGNGDFYSSGNDMNNYINISPDDVEKKAKESAKMLKSFVGSFIDFPKPLIAVVNGPAVGIAVTLLGLFDIVYATDRATFHTPFSSLGLSPEGCSSYTFPKIMGLAKATEMLIFNRKLTAAEACSQGLVTEVFPDNTFQKEVWARLKAYASLPRNSLKLSKQLIRRTDKEKLNEANSKECACLQEIWASDECMNAVMNFFQKKSKL

>XP_007424768_ACBD7_Python_bivittatus

MTLQADFETVAENVKKLKSKPTDDELKELYGLYKQATVGDINTECPGVLDLKGRAKWEAWNLKKGMSKEDAMKAYISKANEMIQKYGM

>XP_007425599_ACBP_Python_bivittatus

MTQAEFDKAAEEVKKLKTQPTDAEMLDIYSHYKQATVGDVNTERPGMLDFKGKAKWDAWSALKGMSKEDAMKAYIAKVNELKDKYGMQ

>XP_015744757_ECI2_Python bivittatus

MFLAAVLKFLRPNPLRSCSPLCGISKVYGVHFPVLQLHTTNSTMQFSQEDFNKAKEQVKVLQDEPSDEVKLKLYALFKQATLGQCNTPKPSMLDFVNKAKWDAWHSLGSMTQDNARQSYIELVSSLVSAESSPVNEIPPGSKSNYETLQVTTKDNITKITLNRPKRKNAINVKMYNEIMEALDEAANDDSALTVLTGNGDYYSSGNDLNNFANISAGGMEESAKNGAVLLKNFVQCFIDFPKPLIAVVNGPAVGISVTVLGLFDIVYASDRATFHTPFSNLGQTPEGCSSYTFPKIMGLTKATEMLLFNKKLTAAEACSRGLVTEVFPDSTFQKEVWTRLKAYANLPKKVSVSKIAYIISCITWCYYILLRWNGIAISNVFSTVKFSNLNNKLDAKLNETVVDVKQLNDKLVILENNMDNKGMEEERDLDTLLDFKEKEPYFGLPVTSEWPNDDLILDLQKDEF

>NP_988874_ACBP_Xenopus_tropicalis

MSQEAFDKAAEEVKQLKSTPTDEEMLETYALYKQATVGDVDTARPGMLDFKGKAKWDSWKKKEGTSKEDARAQYVDWVEKLKAKYGS

>NP_001165067_ACBD7_Xenopus_tropicalis

MSPQADFDKAAEDVKKLKTRPTDEELKELYGLYKQSTVGDINIDCPGMLDLKAKAKWDAWNLKKGLSKEEAMHAYISKTNELVEKYGL

>NP_001135696_ECI2_Xenopus_tropicalis

MQARQEDFEKAQSNLKLLKNDPGNEVKLKLYALFKQATQGPCNVPKPGMLDFVNKVKWDAWKSLGSLPKDDARQSYVELVSSLVSSESSTKSNADPGIGHKKYETIHVSCEDNIIKIFLNRPEKKNAITLTMYKEIGEALEEAGKDESVFAVLSGFGDYFCSGNDLNNFTNIPPEGKEKMAKDSADLLETFVSKFIDFPKPLIAVVNGPATGISVTILGLFDLVYATDRATFHTPFSQLGQSPEGCSSYTFPRIMGLGKATEMLLFNKKLTAQEACNLGLVAEVFPDSSFQKEVWERIKDYSTLPKNSLAFSKQLIRVNEKEKLHAVNIQECERLKERWLSEECMNAIISFFQKRAKL

>XP_018431086_ACBP_Nanorana_parkeri

MDEARRCKRIGGRVRVTPPVAERSEIPPPCNPEWKQEIYRIHQSGDGRRLLCVLLSQAEFEKAAEEAKKLTKKPADDEMLKLYALYKQATVGDVNTARPGMLDFTGKAKWDAWESKKGISQEDARAQYIALVEELKGKYSS

>XP_018422372_ACBD7_Nanorana_parkeri

MAPQADFDKAAEDVKKLTKRPTDDELKELYSLYKQSTVGDINIACPGILDLKAKAKWDSWNLKKGLSKEDAMSAYVSKAHELIEKYSH

>XP_018425955_ECI2_Nanorana_parkeri

MAASLLYVTNCLRLSPFRAVSKLRSVSVLGVHTTARHMQASQEDFEKAQNDLKTLKKDPGNEVKLKLYAFFKQATQGACNVPKPGMLDFVNKAKWDAWNSLKDLPKEKARQSYIELVSSLISAELPVKDPTSTSANKKYETLEVFSQDNITKIFLNRPEKKNAITLQMYEEIGLALDEAAKDDSVITVLTGHGDYYCSGNDLNNFTNIPPEGKEKMASDSALILESFVGKFIDFPKPLIAVVNGPAVGISVTILGLFDVVYATDRATFHTPFSQLGQSPEGCSSYTFPRIMGLSKASEVLLFNKKLTAHEACQLGLVTEVFPDSTFQKEVWERLNSYSSLPKNSLAFSKQLTRAPEKEKLHAVNREECERLKERWLSEECMNAIISFFQKRVKL

>NP_955902_ACBP_Danio_rerio

MSEAEFQKAAEEVKQLKAKPTDAEMLEIYSLYKQATVGDVNTARPGMLDFTGKAKWDAWDAKKGTSKEDAVKAYIAKVEELKGKYGI

>NP_001122240_ACBD7_Danio_rerio

MTLKAEFDQYAEDVKKVKTRPTDQELLDLYGLYKQAVVGDINIDKPGMIDLKGKAKWDAWDSRKGMSTEDAMKAYITLAKQAIEKYGK

>NP_001002645_ECI2_Danio_rerio

MASLIKHVSPWRFARFVRSSKTAFIPCVQLHSTAVMGASVEDFNKAKDKLNTLKKDPGNEVKLKIYALFKQATVGPCNTPKPGMLDFVNKVKWDAWKGLGSISQEEARQQYVDLISSLVGAEAPAVAAQPTGGGKTFQTLLVSTEDNITTIRLNRPDKKNAITVEMYNELIEALELAGKDDSVITVMTGNGDYYCSGNDLNNFTKIPEGGVEKMAKDAGELLRRYVKAYIDFPKPLIGVINGPAVGVSVTLLGLFDVVYATEKATFHTPFSQLGQSPEGCSSYLFPKMMSAAKASEVLLFNKKLSATQACELGLVSEVFPESSFQSEVWSRLKAYAKLPKNSLALSKQLIRGLEEEKLHAVNDAEVERLTERWLSDECMQAIMSFFQGKSKL

>XP_018587841_ACBP_Scleropages_formosus

MSQAEFDKAAEEVKHLKVKPTDAEMLEIYSLYKQTTVGDVNTARPGMLDFAGKAKWDAWEKKKGMSKEDAMKAYIAKVEELKEKYGI

>XP_018618608_ACBD7_Scleropages_formosus

MSLKAEFEQIAEDVKKVKTRPEDQELLDLYGLYKQVIVGDVNTEKPGVLDLKGKAKWEAWNSRAGMSKDDAMTAYIALAKEVINKYGM

>XP_018620717_ECI2_Scleropages_formosus

MAVQLLLYPARLVQVLRTPVLRLHVTARTMGATVEDFNRAKDQLGTLKKDPGNEVKLKIYALFKQATQGPCNTPKPSMLDFVNKAKWDAWKSLGSVSQEEARQKYVELIRLLVGDEESIQAAPTSAGSSAIFKTLLFNTEDNITTICLNRPEKKNAITVPMYSEIVKALEMASKDDSVITVITGSGEYYSSGNDLNNFTSVPEGDIEKKAKDAAELLKGFVKAFIDFPKPLIAVVNGPAIGISVTLLGLFDVVYATERATFHTPFSQLGQSPEGCSSYTFPKMMGAAKASEVLLFNKKLTATEACERGLVTEVFPDATFQIEVWKWLKAYSKLPRNSLALSKQLMRAVEKEHLYAVNAQEVQRLAECWQSDECLNAIVNSFFQAKAKL

>XP_003456254_ACBP-L_Oreochromis_niloticus

MAELQTKFDEAAAEVKQLKAKPTDEEMLQIYSLFKQATVGDVNTSRPGMFDFTGKAKWDAWEKQKGKSKENAMNEYISLVEELKQKYGI

>XP_003450922_ACBD7_Oreochromis_niloticus

MTLQAEFDKAADDVKKVKAKPTDEELLFLYALYKQAVVGDINTERPGMLDLKGKAKWDAWESRKGMSKEDAMSAYIAKAKEVISKYGV

>XP_003440905_ACBP-like_Oreochromis_niloticus

MTEAFHKAVEEVKVLKQKPNQQEMGDLYGLYKQATVGDINTERPGFLDFTGKAKWDAWNARKGLSKEEAMVKYVTLVEELKEKYGI

>XP_003457227_ECI2_Oreochromis_niloticus

MAVSTECRGVCGYLTANDICQTQVSHRDGLRLTGALLALKAEKYQLLYKQLAAASHDLCSTFRHDLKMRLSFILCSFSERVTVRSNIPSLKFHTTASPMMGVTVEQFEQAKSKLSTLKNDPGNEVKLKIYALFKQATQGPCNTPKPGMLDFVNKAKWDAWKSLGSISQDEARQKYCDLIGSLVEAESGSSAQVSAQPAGSGATYETLLVTKEDDITTIKLNRPAKKNAITTEMYNEIIAALEQAATDDSVITVVTGAGDFYCSGNDLTNFTKIPENGVEEMARHGADLLRKYVNAYIDFPKPLVAVVNGPAVGVSVTVLGLFDLVYATERATFHTPFSQLGQSAEGCSSYIFPKLMGAAKASEMLLFNKKLTAVQACELGLVTEVFPDSSFQSEVWTRLKAYAKLPRNSLALSKQLIRSVEKERLHAVNDAEVERLMERWTSDECFNAVMSFFQAKAKL

>SINCAMP00000019993_ACBP_Callorhinchus_milii

MTEVLGQWRCSFTGKVILLCYKRIAFQKAAEEVKHLTTVPTDEEMLAIYSLYKQATVGDVNTERPGMLDFKGKAKWDAWSKLKGTSKEEAMKLYIAKVEEMKVIYGMAE

>SINCAMP00000011604_ECI2_Callorhinchus_milii

SVWCFLPILILHLHCRLIQSFPAVKLHLTGAVMRATEVEFDKAKEHLKTLKNDPGNDTKLRIYALFKQATQGSCSSPKPGMLDFVNKAKWEAWNSLGNISKEDARQKYVDLVGTLISSEAPIQKEATTAGDPKSSYQTLQVTTENNITTILLNRPEKKNAISRKMYEEIMQALEQAGKDDSVLTVMTGSGDYYCSGNDLSNFTQVGAEGVEKLARDSGELLTRYVSHFIDFPKPLIAVVNGPAVGIAVTGLGLFDVVYATDRATFHAPFSQLGQSPEGCSSYTFPKMMGTAKANEILLFNKKLTAAQACDLGLVTEVFPDNTFQQEVWKKLRAYAKLPKDSLRYSKQLIRGMEKEKLHAVNAQECVRLQERWLSEECMNAIMSFFQNKSKL

>XP_005991125_ACBP_Latimeria_chalumnae

MSQAEFEKAAEEVKNLKSKPNDQDMLDIYSLYKQATVGDINTARPGMLDFAGKAKWDAWEARQGMSKEAAMKAYIQKVEELKGKYGI

>XP_005998554_ACBD7_Latimeria_chalumnae

MQGVAASAKPSVELHLSTEIPAAHCSSAMTLQAEFENMAEDVKKLKTRPTDDELRDLYGLYKQAIIGDINIECPGMLDLKGKAKWEAWNARKGTSKEDAMTAYISKAKELIEKYGI

>XP_006010031_ECI2_Latimeria_chalumnae

MATALLGISRWQLLRKARSEFSPPANVLQLEAGFHSGTWRPSKRFVQALYPPRLQAHTTGAAMGATQEQFEKAKEQLGLLKNDPGNEVKLQIYALFKQATQGPCNVPKPGMLDFVGKVKWEAWNSLGNLSKEGAREKYVGLVSSLVSSESSPQAEAVSVAGKQLYKTLQVTTEENITTIRLNRPEKKNAISIVMYNEIMQALEEAAKDESTITVITGTGDYYCSGNDINNFTMIPPEGIEKMAKDSAQLLKNFVLHFIDFPKPLIAVVNGPAVGVSVTLLGLFDAVYATDRATFHTPFSQLGQSPEGCSSYIFPKLMGSAKATEVLSFNKKLTAREACDLGLVTEIFPDASFQKEIWLRLRGCAKLPKMSLVYAKQLSRGPEKEKLYAVNIQECERLEERWLSEDCRNAIAKFFTRSKI

>XP_002602095_ACBP-L_Branchiostoma_floridae

MSEADFEKAAEEVKNLKSSPTDEEKLEIYSLFKQAKIGDVNTARPGMLDFTGKAKWDAWESKKGMSQEDARAKYIAKVEELKGKYGV

>XP_002594078_ECI2_Branchiostoma_floridae

MGRTAFGAHDAAFEAAKERLNTLKEEPDNNVKLQIYALFKQATKGPCNTPKPGAFDFVGRAKWQAWSGLGDISQDEAQKQYIDIINGLAGEESPAETEQAGEEASSYKEIKVTKENKVCSILLNRPAKKNAITWLMYNEIVQALDDASKDDSVTVAVITGAGDYYCSGNDLGNFMNIDPKDMPKMARDGKELLRRFVTAFIDFPKPLIGAVNGPAVGVSVTVLGLFDAVYATDKATFHTPFTELGQSAEGCSSYVFPKLMGNTKANEMLLFNKKLTAHEACERGLVTEVLPHDSFQKEVQTRVEYVAQLPPQSVREGKKLVRDQEREDLHKANEKECEVLEGRWLSEECVRAIMSFFTKKARL

>XP_003250509_ACBP-like_Apis_mellifera

MTLDEKFKKAAEEVKELSAPASDADLLELYSLYKQATIGDCNTSKPGMLDFKGKAKWDAWDKRKGMSQDAAKEQYIHKVEELISIIGKNTTTTGSTETCECIERSKVS

>NP_609187_CG8498_Drosophila_melanogaster

MSELQEFNQAAEDVKNLNTTPGDNDLLELYSLYKQATVGDCNTDKPGFLDFKGKAKWEAWNNRKGMSNTDAQAAYITKVKALIAAVGLKS

>NP_648081|CG8629_Drosophila_melanogaster

MVSFEEAAELAKNFSKKPTDSEFLEFYGLFKQATVGDVNIDKPGILDLKKKAMYEAWNAHKGLSKDAAKEAYVKVYEKYAPKYA

>NP_648083|CG8628_Drosophila_melanogaster]

MVSFEEATELANKFTKKPTDAEFLEFYGLFKQATVGDVNIEKPGALALKDKAKYEAWSSNKGLSKEAAKEAYVKVYEKYAPKYA

>NP_648255_CG5804_Drosophila_melanogaster

MADFNAILEKTKAFSKKPPTEVYLEFYGLYKQFQEGDINIEKPADAEGAAKYDAWLSRKGLSVDDAKAAYVALYEKYNPIYG

>NP_648082_CG15829_Drosophila_melanogaster

MPTFEEIVEKAKNFKNLPSKEEFLEFYGYYKQATVGDCNIEEPEDEEKKARYNAWKSKAGLTADDAKAYYIEVYKKYAPQYE

>NP_729218_ACBP_Drosophila_melanogaster

MVSEQFNAAAEKVKSLTKRPSDDEFLQLYALFKQASVGDNDTAKPGLLDLKGKAKWEAWNKQKGKSSEAAQQEYITFVEGLVAKYA

>XP_974824_ACBP-like_Tribolium_castaneum

MSLDERFKKAADDVQKLKSKPSNDDLLEIYALFKQGSVGDCNTDRPGMLDLKGKAKWDAWNGKKGMSQDKAKEEYIAKVESLIQSIGLQ

>XP_974813_ACBP-like_Tribolium_castaneum

MSLDEKFKSACDQIRQFTKRPSDSDMLEVYSLYKQATVGDINTPKPSEAKAKAKWEAWSGKKGLNANVAKEQYVAKIKALAPTYA

>KDR20996_ACBP-L_Zootermopsis_nevadensis

QKFNEAAESVKNFKKRPSDDELLQLYALFKQATDGDNHKEKPGMLDLKGKAKWEAWSDKKGQSKEVAMEAYVALANKLEARYA

>KZS11387_ACBP-L1_Daphnia_magna

MSLDEKFNKAAESIRSMTTSPSDDEMKEIYALYKQSTVGDVNTARPGMLDLKGKAKWDSWESKKGMSADEAKEAYVTKTEELVAKYSASSA

>KZS07345_ACBP-L3_Daphnia magna

MSLDEKFNKAAEDIKALTARPTDDELKEIYALFKQATIGDVNVARPGMLDFKGKAKWDSWESKKGMSSDAAKEAYVAKTEELLAKYSA

>JAN37855_ACBP-L4_Daphnia_magna

MTTSPSDDEMKEIYALYKQSTVGDVNTARPGMLDLKGKAKWDSWESKKGMSADKLRKLTSPRRKSWWPSIVHQVHXSIA

>JAN53111_ECI2_Daphnia_magna

MIIPTAANSFRSQCLAAMLLKRSFVNSPNQRTFLAPANGIKHMSTSISQQFDEAQKRLQTLKTSPGNEAKLKLYGLFKQATAGAVNTKRPGMTDFVGKAKWDAWNSLGSMSQEEAKNKYIEFVDSLVGPANSTESLNVVESSSPGFDVTIDGKLRIITLNKPTTKNAFTLGMYVGFAKLLKEAAEDPNTTLVAVTGAGNIFSSGNDLTSFTSFTGTMREAAEEGKRCLSIFVGSLIDFPKPIIGVVNGPAVGIACTILGLMDVVYATDRGWFQTPFSALGQSPEACSSHIFPKLMGSLKANEMLFFNKTITATEACKLGLVTSVLPDANFQSEVWPKLKEWSELPQKVSDRRKHFIDSAIKKLTSLFQSLVHSKELSRQFDRELLHKVNAAECDRLLERWQSSDCMEAVMKFFSKNAK

>XP_018023004_ACBP-L_Hyalella_azteca

MSTETLFQEAAEKVKKLAKQPTDDELKEIYGLYKQATIGDINTERPGMLDFKGKAKWDSWESKKGMTKEAAMTAYVAKVEELVAAYGLQ

>XP_018025342_ECI2-L_Hyalella_azteca

MVAKSFLAASIPFRRCFFNSTANHVVNVQACRNLGPSFIGGAPRYMSSSIAQQFEEVKHRLASLKEDPGNEVKLKMYALYKQGMEGKATGKRPGVMDFVARAKWDAWNSLADMSKEEALKAYLAIVDELSAAQGATSTEEPSILVTVEDGLRIIKLNRPKKKNALNPEMYFRWTELMHEAAKDDKTVLTAITGAGDFFCSGNDLGNFMNIPPGGEAELANRTKEFLYQFIDAFIEFPKPLIGVVNGPAVGVSVTTLGLYDAVYSSDQAWFQTPFSQLGQTAEGCSSYVFPRLMGPGIASEMLMFNKKLSAHEAQRYRLVTEVFPHDRLQQEVWPRLQALAKLPARCLIYSKELTRAADKEILKKTNRAECERLCERWQSEDCKNAIMNFFSRQRK

>XP_015907443_ACBP-L1_Parasteatoda_tepidariorum

MSLEEKFTKAAEDVTKLKTKPTDEELLEIYALYKQASIGDVNTTRPGLFDLKGKAKWDAWNSKKGMDQTEAKEAYVTKVNHLIEIYGLA

>XP_015904839_ACBP-L2_Parasteatoda_tepidariorum

MSLDEKFNTAATSVKDMKSRPSDSELLELYSLYKQATCGDCSADKPGALDLKGKAKWEAWTGRKGMAQDAAKEAYVTLANTLITKY

>XP_015922765_ECI2_Parasteatoda_tepidariorum

MSIIFSSIRKLSSSLRPSIFLPYSKSSKSIYLLKSFSTSVRMNSSLTFQQACDKAKTLEKDPGNDVKLKMYALFKQATEGPCSQTKPSVFDMVGKAKWEAWNTLGSLSQDEAKSQYVSLINGLFDQKQQSEQSAEIQELKYEGIKLSVSGDITELRFNRPEKKNAITTKMYKDIALALKEASENDTAITILTGEGDYYSSGNDLSNFASVDQDISAAAKKAGQLLNEFVGAFIDFPKILVAAVNGPAVGIPVTLLGLCDVVYASDKATFQTPFSFLGQSPEACATYTFPKIMGYSKANEVLLFNKKFDAVEAKSMGLVSEIFPNDTFHEDVKRKLEFLSQASKQSMILSKELIRKFEKDTLHTVNDEECKLLVERWQSQDCMEALMRFFTRKSKL

>sp|Q20507.2|ACBP3_Caenorhabditis_elegans

MSLQEKFDAAVEIIQKLPKTGPVATSNDQKLTFYSLFKQASIGDVNTDRPGIFSIIERKKWDSWKELEGVSQDEAKERYIKALNDMFDKIAEELDVAAWLEQIDPVIKTNLALIGK

>sp|O01805.1|ACBP1_Caenorhabditis_elegans

MTLSFDDAAATVKTLKTSPSNDELLKLYALFKQGTVGDNTTDKPGMFDLKGKAKWSAWDEKKGLAKDDAQKAYVALVEELIAKYGA

>NP_496552_ACBP6_Caenorhabditis_elegans

MPISELEKKWTGLTFEIAAEEMRRLKSEPTDRERLKLYALYKQALHGDIPNEDVYPVPAGDEVGRKKYAAWKSQKGANSEKCRADYVAIAEEMIKKYGRNIVRCKWNSEVWSVDY

>XP_784299_ACBP-L_Strongylocentrotus_purpuratus

MSDAFNTAAAEVKDLATSPSNEDLLKIYSLFKQVTVGDCNTDRPGMLDYKGKAKWDAWNGLKGKSTADAEKEYIELVGQLKTSCGMK

>NP_496330_ECH-4_Caenorhabditis_elegans

MLRNLTLAARSVLYNPQVSVRSFSAQADFEKAQKNLKTLKEEPDNDVKLQLYGLFKQATAGDVQGKRPGMMDFVGRAKYDAWNTLKGQTQDEARANYAKLVGGLISEEASAAPEPTGPSIEGLENVDGLSVTREGKVFKIALNRPKKFNALTLEMYQGIQKALEVSNNDKSTSITVITANGSYYCAGNDLTNFKAAAGGTKEQIADMANTAKVIMKDYVNAYINHEKPLIALINGPAVGIAVTVLGMFDYVIATDKASFHTPFAPLGQSPEGVSSYTFPLIMGSLRASEMLLVCKKISAQTAKDYGLVNEVVPDAEFQSHAQKTVEAFSQLPPETLRINKKLLRSLHKEKLLEVNNIEADQICERWQSKECHQAIAAFMTKGAKK

>XP_780031_ECI2_Strongylocentrotus purpuratus]

MAALFSLKCGTRRLFNICNTCTTRLHAPVLKRQIQNTACMMSFSDAEFTAAKDRLNSLKEDPGNEVKLQIYALFKQATAGTCNTPKPGAFDFVGKAKWSSWNSLGNMSQDQAKEKYVGIVDDLVAQEGGEAETASTTQGSLSFTGLKYTVDNGVATITLNRPNKKNAVTTEMYSEWTAALKMAGEDDRVVLAVITGAGDYYCSGNDLNNFMKIDPSVLHEESVKGSDLLEIFVNGFIDFPKPLICAVNGPAVGISVTTLGLMDVIYASDKATFHTPFAALGQSPEGCSSYTFPKIMGTAQANECLLFGKKLTAQEAFDRGLVTEVIPDAQFRETVDKKVKEYAQLPRNALRLAKNLIRETEKERLHKVNRAECDLLVDRWTSDECTQAIMNYFSKSKL

>XP_015749156_ACBP-L_Acropora_digitifera

MSEAFLKAAEEVKTFKTEPSDSDKLELYAFYKQATVGDCNTDRPGMMDFKGKAKWDAWNGKKGTSKDAAETTYIAKVEELKKTCS

>XP_015747922_ECI2_Acropora_digitifera

MAFCLRSLLHAKKLSFRPTPFTFSILRQIQIGNIRQVEGDKDFQAAVAKVKTLKQDPGNDNKLKLYALFKQASAGKCNEPKPGAFDFVGKAKWTAWNDLGDLSKNDAQQKYIIYVNDLVDKFGSTDEESNVVSNSEEKTTNMGSKYKELEVTLENGVQKIRMNRPAKYNAITWEMYHEFMTALEEGGKNDACVVAMVTGTGDYYCSGNDLSNFTRIPPEGPQKMARDAREILKKFVAHFIDFPKPLIAAVNGPAVGISVTIMGLFDLVYASDQATFHTPFMELGQSPEGCSSFLFPRIMGPAKANEMLLAGRKLTAMEAHQCGLVTDVIPHNNFQGEIERRVQALGKLPPKSLRLSKELIRDSSRDLLHEANEKEADLLEERWLSEECMQAIMNFMARKSK

>XP_002736654_ACBP-L_Saccoglossus_kowalevskii

MPSDAFNKAAEDVKNLKTRPSDAEMLKLYALFKQTSVGDCNTDRPGMLDFKGKAKWDAWNEKKGTSQADAEAKYIELVEELKGKYGMA

>XP_002741676_ECI2_Saccoglossus_kowalevskii

MAFCRRIGVLAFDSVVMKTSRFSVIRPVSTSGKNFAAIDTEFSAAKERLTTLKEDPGNEIKLKLYALFKQATVGKCNAKKPSTFDFVGKAKWTAWNSLGVLSQEDAQKQYISTVNDLVAVEESASEEVASGTAGDYKQIKVTVEDGVCTILLNRPAKKNAINLEMYNEIGVALNEIGKDPKVVLAVVTGAGDYYCSGNDLENFMNIDPSKIHEFAKEGADVLRQFVASFINFPKPLIGAINGPVVGVAVSTLSLFDVVYATDRATFHTPFTALGQSPEGCSSVLFPRIMGQGKVCPGF

>jgi|Lotgi1|205113|Lottia_gigantea

MSAEFTTAAEEVKNLKEKPADAEMLEIYALYKQATVGDVNTTRPGMLDFTGKAKWDAWEAKKGTSKEDAEKAYIAKVAELKGKYGMK

>jgi|Lotgi1|178218|Lottia_gigantea

MPAHDAEFNKAKERLNTLSEAPGNDIKLKIYGLFKQATEGKCNKPKPGMMDLVGKAKWQAWNGLGDISQDDAQKKYIDLVNELIAADSPEPTSTTDSKFKTILVNKENKIYKITLNRPKKMNALNHLMYEEIMQALDEAGKDNSVLTVITGTGNYYCSGNDLENFTSTGGADIPAMAKQAREILYRFVGSFIRFPKPLIALINGPAVGISVTTLGLFDVVYCTDRATFHTPFSSLGQSPEGCSSYIFPQLMGSAKASEVLLFNKKLTAQEALDRNLVTEIFPDASFEKETMARVTKYAQLPPQSLQKSKNLMRQMQKDKLDKVNSEECDLLVERWQSDECMNAIMNFFKRKESKL

>XP_003386304_ACBP-like_Amphimedon_queenslandica

MSNSEAFLKAAEDIKTLTTSPTNEEFLDLYKFYKQATVGDCNTDRPGMFDLKGKYKWDAWNSLKGTGKEEAEKKYVDLVTELLTKYAN

>XP_003384577_ECI2-like_Amphimedon_queenslandica

MSLVFARRVLQGRAPLLLSSSSSAVPSIHMLWRRSFSLSNEDKEKLEAAKGKVSQLTTDPGNDKKLELYALYKQAVEGPCDTPKPGVMDFVGRAKWNAWNSLGQLSQSEAAVKYSELVDSLVGSSTSNETASPSNPVKLSSDDLLVTEEGGVQTITLNRPSKKNAITVKMYEDITSLLNSSASNPAIKATVVTGNGDYYCSGNDLSNFMNIPPEGPEKLAADSAILLRNFVSSLIRYPKPLVACVNGPAVGISVTTLLLFDLVYAADNATFHTPFMQLGQSPEACSSFLYPRIMGPAKSNELLILGRKISAEEAFERNMITRVFPKDELQERVKEIVRELSELPHQSVVKSKALIRSSFTDLLEDANAKECELLRERWLSEECMQAIMKFLEKRK

>XP_002110753_Trichoplax_adhaerens

MEEEFKSAVDKINNLASKPSNEDLLEIYGLYKQATVGDCNTDRPGFFDQKNRAKWDSWNSKKGMSTEEAKQAYIKKANSL

>XP_646321_Dictyostelium_discoideum

MTTFEEAAQKVKEFTKKPSNDELLSLYGLYKQGTDGDCNISEPWAVQVEAKAKYNAWNALKGTSKEDAKAKYVALYEQLATKYA

>XP_001744739_Monosiga_brevicollis

MTEAQFNKAVWLIRNGPAVGDSSNETKLSFYKYYKQATVGDNNESQPWAVQLEASAKWKAWNSVRGMSKEDAMKAYVDLLAKDDPNWEQHPALKDYKA

>XP_001011628_Tetrahymena_thermophila

MISKRLNNQDNASKKFRYALLILNLAIPFKKRGKKRYQLINIINQFVKLQTIKFQKETKVVKNQQKNKQMTSLEERYNKALEFIKNPPADYPPIDMDNTQRLTFYAIFRQIKDGPCKGPQPSRLKVVERAKYDAWKALGKMSKEDAMKKYITEITKLAPGWEKPTPKL

>XP_001012898_Tetrahymena_thermophila

MPECSLKTAGKFQNQQYLSKQIIKVKINKQSKKTKNQEMAENFELAAEEIANFFKNKGDTSDDNKLELYALYKQGTTGDNTTAKPGMLDFKGKAKWEAWNKKKGVSQNDAKAQYVVLANKVLATVGKKV

>NP_011551_ACB1_Saccharomyces cerevisiae

MVSQLFEEKAKAVNELPTKPSTDELLELYALYKQATVGDNDKEKPGIFNMKDRYKWEAWENLKGKSQEDAEKEYIALVDQLIAKYSS

>XP_001383919_ACB1_Scheffersomyces_stipitis

MPSQEFTDKANAVQNLSKRPNDDELLKLYGLYKQATVGDNTTDKPGIFDLKGKYKWQAWKDLEGTSQEEAEQSYIELATSLIEKYDS

>EEQ46883_ACB1_Candida_albicans

MVSAEFEEKANQVSNLSKRPSDDELLKLYGLYKQATVGDNNTDKPGTFDFKGKYKWQAWKDLEGKSQEDAEKEYIDLATELISKYN

>XP_002143080_ACB1_Yarrowia_lipolytica

MPSAEFTAAADSVQKLPKTPSDDELLELYGLYKQATVGDNNTDRPGAFNFKAKYKWDAWDKLKGKSQEEAEQEYIALVQTLSDKYN

>NP_596820_ACB1_Schizosaccharomyces_pombe

MSSTFEQAAADVKELKETPNSDELLKLYALFKQATVGDNNTEKPGLLDLKGKFKWNAWEELKGKSKEDAASEYISFVDELKTKYGMK

>jgi|Spore1|5942|Sporesorium_reilianum

MSVEAKFNKAVSIVGSLPKDGPVQPSQDDQLKFYGLYKQATIGDVNTKKPGMFDLAGKYKWEAWNKNQGLSKEEAQQAYVDALLAILKKHEDEGDSAEHIKAIESA

>jgi|Elmca1|251068|Aporpium_caryae

MSEAKFNKAVAIVQSLPKDGPVKPSQDEQLQFYAQYKQATVGDVNTSRPGLLDFVGKAKWDAWKAVEGTSKEAAQAKYVELLLAVLKAQDSEEAKKYLKELEETN

>jgi|Clapy1|1795290|Clavicorona_pyxidata

MSASKFEKAVSIVQGLPKGGPIQPSQDEQLYFYSYYKQATVGDVNVPRPGMLDFVGKAKWDAWKGVEGTSKEEAYSKYVQKLIEVLTAADTAEAKGYIAEIEAA

>XP_001874747_Laccaria_bicolor

MSSHELIDAQFDRAVEIVQSLPKTGPIQTDYDEKLTMYSLYKQATVGNVRAPRPGIFDMLGRAKWDAWAKHKDLDPFEAKWLYVDALLKV

>XP_001880619_Laccaria_bicolor

MSEAKFDKAVAIIGALPKEGAIKPTQEDQLYFYKYYKQAKIGDNTTVRPSGFLDFAGKAKWDAWSEVKGTSKETAWKLYVDKLLEILKKVDDEESKKYIAELEAA

>XP_001836410_Coprinopsis_cinerea

MSEAKFNKAVEIVRSLPKDGPIQPSQDDQLFFYKYFKQGTIGDNTTTRPGMFDFTGKAKWDAWESVKGTSKEDSWKAYVEKLLEILNKVGDEDSKKYVAEIEAAA

>jgi|Croqu1|132120|Cronartium_quercum

MTADKKQVEFEKAVEIVQGLPKDGDEKPSQTEQLAFYALYKQATIGNVNIPRPGILDFTGKAKWDAWKEKEGLSKEDAQAEYVKLLREYLEKFSDKEQATKLLAQLDSA

>jgi|Pucgr2|11284|Puccinia_graminis

MSEIDKKFDRATELVKSLPSDGDDKPSQEEQLEFYALFKQATVGDVNSSRPGMMDFTGKYKWDAWKAKEGMKSEEAKTKYVELLKSKLEKSSNQEQAKKILEQLEAA

>jgi|PleosPC15_2|1090291|Pleurotus_ostreatus

MSAKFEKAVSIVQSLPKDGPIKPTQDDQLNFYKLYKQATVGDVNIPRPGMLDFTGKAKWDAWDSVKGLSKEDAMAQYVEKLIEILKKADTEEANKWIAEIEAA

>jgi|Amamu1|262412|Amanita_muscaria

MSEVTKAKFDKAVQIIESLKNVPNDGFKLETDDLLFFYRYYKQATIGDVNTKKPEGMFASLTANLVDFRETAKRNAWESVKGTPVEVCYKKYVEKLIELLKKAGDSKSLELVQELEAA

>jgi|Saico1|60794|Saitoella_complicata

MPSAEFTKAAEEVKNLSKKPTDDELLCLYGLFKQATIGDNNTDKPGMFDLKGKYKWEAWNKLKGTSQEDAEKQYIALVEELKAKNA

>jgi|Boled1|964172|Boletus_edulis

MTLTLQEKFDKAVKYVQSLPESGPLKPDQDTKLKYYAYYKQATIGDVNTGRPGIMDFVGKAKWDAWNAVKDTSKEDAQKAYVELLVMHLEKITDKSEEVKELIQELKE

>jgi|Morco1|607810|Morchella_conica

MTSAEFDAAVTAVNSFKKMPSQDDALLFYAYFKTAKFGKPTDGRPGLFDMKGRYKYDAWAKAADELSPEEAQKKYIELYESKKADYEN

>jgi|Gyresc1|520122|Gyromitra_esculenta

MSELPKVPKSPEFERATAAVRSFVQMPNEKEALKFYGLYKFGCHGKNSEGPPLFPTFYAGKKWAAWNTIAESGITPDDAQKQYVAFYEANKEKYEAGHV*

>jgi|Gyresc1|536723|Gyromitra_esculenta

MTTLPEHPQSAEFQEAAAGLHGLAKRPSNHELLEFYGLYKVGRHGKNITPQPWTKLSMEGQKWESWTKYSKKGITPEEAQAEYVEKYNAIKEAQNNASK

>jgi|Kalpfe1|694171|Kalaharituber_pfeilii

MGAEFEKAAEDVKNLESKPSDDELLKLYGLFKQATTGDNTTDKPGLFDFIGGYKWKAWKALEGTTKEDAEKQYIAFVNELKVKHGFQMYEG

>jgi|Artol1|1519|Arthrobotrys_oligospora

MPSPEFDAAAAAANAFTKKPSDDELLKLYGLFKQATVGDVNTDRPGMMDFKGKYKWDAWKSEEGKSQADAEAEYIAYVEGLKAGFA

>jgi|Monha1|10055|Monacrosporium_haptotylum

MVSAEFQAAADKVNNLTSKPTDEQLLKLYGLFKQANVGDVNTSRPGMMDFKGKAKWDAWKSEEGKSQEAAEAEYIAYAEEVVASVA

>jgi|Phybl2|124466|Phycomyces_blakesleeanus

MPSANFEAAAAAAQAFTSKPSNDELLALYGLFKQATVGDNETTRPGAFDFKGKAKWDAWTAKKGLSSEDAEAQYIALVEELKAKQ

>jgi|Phybl2|104584|Phycomyces_blakesleeanus

MSAIPSHVSERYINSRYNKALNIVQHLSASSSVQPTKEEKLELYALYKQVSHGNVNTPRPGMFDLVGKAKWDAWKNQESMAAIEAKYRYVDLLLRVASEVSVIKGFFD

>jgi|Phybl2|110945|Phycomyces_blakesleeanus

MIPPHYTDRYVDQRYNKALMIVQNLPASSSFQPTKEQKLELYALYKQVSHGSIDTQRPGIFDVVGRAKWDAWKKLEGLNDLEAKHRYVDTLLRSATEVY

>jgi|Absrep1|411144|Absidia_repens

MPSPAFEQAAKDAKNFTKLPEDEELLQLYGLYKQATLGDNNTDKPLLDFKGRYKWDAWNDNNGIPQVEAEVQYIALVESLKTKYQ

>jgi|Absrep1|411342|Absidia_repens

MPSAEFNTAAEEVKTLAQTPSNDVLLELYALFKQATVGDNTTDRPGVFDMKGKAKWDAWTKIKGTSQEDAEKQYIALVQKLKN

>jgi|Mucci2|146146|Mucor_circinelloides

MSFIPTHYSNRYINQRYNKALYIVQHLPSSSNVQPTKDQKLELYAFYKQVSHGDIDTQRPGIFDVVGRAKWDAWKRLEGISEMEAKHRYVETLLQAAKEVQRWQRALVECPC

>jgi|Mucci2|145636|Mucor_circinelloides

MASIPPHYTDRFILQRYNKALHFVQHLPATSNFQPTKSQKLELYALYKQVSEGDINTQRPGLFDVVGRAKWDAWKKLEGISTLEARHIYVEALLRV

>jgi|Mucci2|113048|Mucor_circinelloides

MPSAEFNTAAEEVKNLSTKPSDNQLLELYGLFKQATVGDNTTSKPTFDIKGRYKWDAWTKLKGTSQEDAEKQYIALVESLKAAQ

>jgi|Mucci2|110863|Mucor_circinelloides

MPSPAFEQAAKEVHDLTSKPSNDDLLKLYALYKQATIGDNETTKPTFDIKGRYKWQAWEDLKGILPVEAETQYIALVQELKTKHQ

>jgi|Blatri1|382870|Blakeslea_trispora

MPSEKFNAAAEEVQKLSTKPSNDELLKLYALFKQATVGDNETSKPTFDIKGRYKWDAWTEVKGLSKEEAEEKYIALVEELKAKQ

>jgi|Spifus1|1857028|Spinellus_fusiger

MPSAAFESAAVEIKQLTTSPSNDNLLKLYGLFKQATVGDNTTSKPTFDIKGRYKWDAWTELKGLSSQEAEDKYIALVKELTSQ

>jgi|Rhior3|9713|Rhizopus_oryzae

MPSQQFTTAAEEVQKLSTKPSNDELLELYGLFKQATVGDNETSKPTFDIKGRYKWDAWTKLKGMSQEEAEQKYIELVEKLKASQ

>jgi|Conco1|56255|Conidiobolus_coronatus

MSGLNNPEFAKVAEDVKTLTTKPSNDVLLALYAHFKQATVGDNNTSAPGMFDLTGKAKWNAWNDIKGLSQEDATAKYIEIANKAIANQ

>jgi|Conco1|20929|Conidiobolus_coronatus

MEIDNIEESTEENSNDSTEADTEADTEDSSDSTNSENLEQKIEEIKSLYSQLPQLKQNPGYEMLLDLYSHYQQATLGDNTTEKPNILDFEGKSKWYSWYQLKGMTKGEAVEKFMQIYTLIMAKTLKKV

>jgi|Maseb1|107623|Massarina_eburnea

MPSAKFDEVYKKTRDIKSGPSNDDLLNLYAYAKVAQGEDIEKAAKPGMMDFTGKAKRKRWQEVVDAGTSKSDAEKKYIELGEGLISKHS

>jgi|Verda1|497|Verticillium_dahliae

MSVPQSEAFKTAVVDSQKLTSKPSNDDLLQLYALFKIGNGEDIRQSPSPGMFDLKAKAKRSAWQKETDAGTTAEQAQAKYVELVNSLKESHGYDADKVPESVGA

>jgi|Xylhe1|258819|Xylona_heveae

MAQSPEFELAVKQVKQLTKPLGPEDMKRLYGLYKQATQPQTLDEFYANVKKPEGMFNFKEKGKYAGWEQAVKDAPTGEEAQKLYIEFVESLKEKYAFDPNKEPESVKS

>jgi|Trigu1|1083200|Trinosporium_guianense

MAQSPEFEKAVKEVKQLTKPLGPEDMKRLYGLYKQATQPQSLDEFYANVKKPEGMFNFKEKGKYAGWEQAVKDAPTGEEAQKLYIEFVESLKDKYAFDPSKEPESVKS

>jgi|Chalo1|564282|Chalara_longipes

MSAFDTAVADSKKLTSKPSNDELLKLYGLFKVATGEDITKAENPGTFDLKGKAKKRAWQAVVDEGLTSDEAKEKYVTLVEGLKAKYGYDATKEPEAVGGGS*

>XP_568458_Cryptococcus_neoformans

MVNTKAQFDKAVAIVKGLPEDGPVKPTQDDKLAFYAHFKQANEGDVSGPAPGMFDFVGKAKYNAWKKIAGMSKEDAMAKYVELLTEMLKKSDDEASKQYLAELEAAGASA

>jgi|Aspnid1|4403|Aspergillus_nidulans

MSVPTFTAALTASQNKEKYSAKVQELAAAINADALSSAIEAILSGGDDATVSDAEQSKALTAGFEYATELVKELKSSPGNDDKLKLYAFFKRSKNEEPAAPGAFSFEAKYKYNAWKEIKDISQQRAQALYIQKVNALLESIGTN

>jgi|Morel2|14613|Mortierella_elongata

MACVSVCLGSLQPFNLELQTPEPSAEFDAAAKKIKELTVSPSNDNLLKLYALFKQATVGDNDTTRPGAFDFKGKAKWDAWTEKKGLSQEDAEKQYIELVKDLTA

>um02959_Ustilago_maydis

MSAEAKFNKAVSIVGSLPKDGPVQPTQDDQLTFYGLYKQATVGDVTSKRPGMFDLAGKYKWDAWNKNQGMSKEDAQQAYVDALLEILKKHEDEGDSAQYIEQIQNA

>jgi|Basme2finSC|346410|Basidiobolus_meristosporus

MSDAASTGNTAFANASEEVKTLATKPSNTQLLELYALFKQGIFGDNTAERPGMFDIQGKAKWDAWTGKKGMSKEEAQAAYIALVESLKN

>jgi|Picpa1|36433|ACBP-L_Pichia_pastoris

MVSQEFNDKAEAVKNLKTKPNNDELLKLYGLFKQATVGDNTTEKPGVFDFKGKAKWEAWDKLKGTSQEEAEQEYIAYVGDLEDKYN

>jgi|Ascru1|77692|Ascoidea_rubescens

MVSQLFTEKAEAVKTLPKTPDNDELLELYGLYKQATIGDNKTDKPNAFNFKAKYKWEAWKDLEGTSQEEAEELYIKLVDKLIAKYTS

>jgi|Zygro1|529|Zygosaccharomyces_rouxii

MVSQLFEQKAKQANELPSKPDTDELLKLYALYKQATVGDNNKEKPGIFNMKDRYKWDSWEELKGKSKEDAEKEYIEFVEELQQKYSQ*

>jgi|Gloin1|343716|Rhizophagus_irregularis

MTDINRSAEFDAAAKEFEEVVKNHNPSDEDKLEGYALFKQGSFGDNTKPEPGFFARTDKAKWNAYNTKKGITPEDAQKQYVDFVAKMKEKYGS

>jgi|Gloin1|349696|Rhizophagus_irregularis

MTDTQSSLIFNNAAQDFATHAKANKFTAEEVLEGYGLYKQAIAGDIPSEKNNSDAVQDMAWSMKKGIARDDAASQYIAFVDKLKSKYSL

>jgi|Synplu1|692118|Syncephalis_plumigaleata

MAEATGNAAFIQAAKDVVTFKSADNNESKLKLYGLFKQATVGDNETARPGLFDPAGKYKWDAWTENKGISQAEAQQKYIDYVEELRTKESS

>jgi|Tapde1_1|1756|Taphryna_deformans

MSKADFDKAAAEVKNLPSKPSDDDLLNLYGLFKQATVGDNTEAKPGMFDLKGKYKWEAWNKRRGLSKEDAQKEYIDLVEKLKAK

>jgi|Coere1|78996|Coemansia_reversa

MSNVNVELSAEEKARLENLNKEFLEAKKNSEVLPKKPSNDEKLKLYSLYKQGTVGDNDTEKPSAFSFERKYMWDAWTKLKGMKSEEAKQKYIDFVKELEEKFKKELE

>jgi|Linpe1|263816|Linderina_pennispora

MSEAASTGNAAFVTASQEVTKLSTAPSNDIKLQLYANFKQGINGDNTTPAPGFMDFTGKAKWNAWTALKGTSKEDAQAKYIALVEELKKTHA

>jgi|Blabri1|377594|Blastocladiella_britannica

MAETTGNTAFIKAADAAKTLTYQPTNDELLELYAFFKQGTVGDNDTPRPGMFDIKGKAKHDAWSAKKGLTKEDAQAKYVELVEALKLK

>jgi|Blabri1|345767|Blastocladiella_britannica

MTSPPAALAALFSRAVQAIQNLPPAGPTDAGLAPTNTEKLEFYALYKQATTGPLDAATTKRPGFFDPVGRAKYDTWLGKVRLTRAEAMRAYTDLFMAFLRR

>jgi|Blabri1|377593|Blastocladiella_britannica

MPSDAFNTAANEVKSLKTTPSDAQMLEVYALFKQATVGDVQGERPGMFDLKGKAKYDAWAAKKGTSTDAAEKEYIALVESLKASHGI

>jgi|Allma1|3154|Allomyces_macrogynus

MPSDAFTQAAEEVKSLAYQPSNDELLELYALYKQATVGDNTTARPGMFDLKGKAKWDAWAAKKGLASEEAEAKYIALVEEFKKK

>jgi|Rozal1_1|5921|O9G_001025_Rosella_allomycis

MSDPKSTNNPEFIAAADAAKSLKTTPSNDDLIKLYSLFKQAIEGDNTTPQPGMLDLKGKAKWNGWNSQKGKSKETAQKEYIQFVKELQSK

>jgi|Rhihy1|780965|Rhizoclosmatium_globosum

MPSADFEAAAAAAKSLSYNPSNDELLALYGLYKQATVGDNTTERPGLFDLQGKAKWDAWEKNKGTSQADAEAKYIELVKSLQAK

>jgi|Neosp1|386106|Neocallimastix_californiae

MSGNEKFDTAAVEVKQLTYRPSDSELLELYSYYKQGTIGDNETDKPGIFDLKASAKWKAWNKVKGMSKEEAQSKYIELVEILKAKN

>jgi|Pirfi3|348160|Piromyces_finnis

MSGNDKFDTAAVEVKQLTYRPSDSELLELYAYYKQGTVGDNETEKPGIFDLKGSAKWKSWNKLKGMSKEEAQAKYIELVETLKARN

>jgi|Malsy1|2953|Malassezia_sympodialis

MSDTQFQKAVEIIRNLPKNSPAKVTQAQQLRIYGLYKQATEGDVSTSRPGMLDFTGRAKWYVCFGIDSFYRDAWNSEKGKSSEQAKKDYVDLFIEVHDAEFNKYLEEVKNA

>jgi|Psean1|70307|Pseudozyma_antarctica

MSVEAKFNKAVSIVGSLPKDGPVQPTQDDQLKFYGFYKQATIGDVNTKKPGMFDLTGKYKWEAWNKNQGMSKEDAQQAYVDALLQILKKHEDEGDSAQHIKDIESA

>jgi|Batde5|86602|Batrachochytrium_dentrobatidis

MSDPASTGNSDFIKAAADVKNLATKPNNEELLQLYALFKQSISGDNTSACPGMFDLQGKAKWSAWEKVKGTSKEDAQAQYIALVKQLQGKN

>NP_001056611_Oryza_sativa

MGLQEEFEEFAEKAKTLPDTISNEDKLLLYGLYKQATVGPVTTGRPGIFNLKDRYKWDAWKAVEGKSKEEAMADYITKVKQLLEEASASTS

>NP_001061062_Oryza_sativa

MGLQEDFEQYAEKAKTLPESTSNENKLILYGLYKQATVGDVNTARPGIFAQRDRAKWDAWKAVEGKSKEEAMSDYITKVKQLLEEAAAAAS

>NP_001050536_Oryza_sativa

MGLQEDFEEYAEKVKTLPESTSNEDKLILYGLYKQATVGDVNTSRPGIFAQRDRAKWDAWKAVEGKSKEEAMSDYITKVKQLQEEAAALKAVLSVEISRKFDRNILQTCKFSGHGCPEKLPGQKSSNSSEFLVAGAH

>Glyma04g14650|Glycine_max

MGLKEDFEQYAEKAKTLPPTQSNEDLLILYGLYKQATVGPVNTSRPGMFNMRDRAKWDAWKAVEGKSKDEAMSDYITKVKQLLEAAGMPA

>AT1G31812|Arabidopsis_thaliana

MGLKEEFEEHAEKVNTLTELPSNEDLLILYGLYKQAKFGPVDTSRPGMFSMKERAKWDAWKAVEGKSSEEAMNDYITKVKQLLEVAASKAST

>PGSC0003DMG400030867|Solanum_tuberosum

MALKEEFEAHAEKAKTLPESTTNENKLILYGLYKQATVGNVNTSRPGIFNMRDRAKWDAWKAVEGKSTDEAMNDYITKVKQLLEEGAASA

>270651|Selaginella_moellendorffii_ACBP

MATTEEQFKKAAEDALKLPPTTTDADKLILYGLYKQATVGNNETSRPGMLDFKGKAKWDAWKKCEGKSADDAMKDYIAKVEQLMEATA

>Pp1s7_333V6|Physcomitrella_patens

MGLDEDFQAAAAAAKELKTKPSDDDLLILYALYKVATVGKVDTSCPGMFDFKGKAKWNAWKKAEDKSPEDAKRDYILKVQQLQEA

>Pp1s36_294V6|Physcomitrella_patens

MGLDEDFEQAAKDAKALTAMPSNDDLLILYGLFKVATVGKNNTVRPGMLDLKGKAKWDAWKKVEDKSPEDAKRDYILKVQQLQEA

>Vocar20003619m.g|Volvox_carteri

TSRRNPCPDTVKQETMGLKEDFEAAAAEATNSLPDTLSNDEKLELYALFKQAKEGDCNTSQPGIFDPKGRAKWNAWNGKKGTSQEDAMKQYIEYVAALKAKHGTK

>XP_016557647_ACBP-L1_Capsicum_annuum

MALKEEFEEHAEKAKTLPESTTNENKLILYGLYKQATVGNVNTSRPGIFNMRDRAKWDAWKAVEGKSTDEAMNDYISKVKQLLEEAAASA

>XP_016572376_ACBP-L3_Capsicum_annuum

MPLKEEFEEHADKAKTLPESTTNENKLILYGLYKQATVGNVNTSRPGIFNMRDRAKWDAWKAVEGKPTDEAMNDYITKVKQLLEEAVASA

>Zosma123g00500_Zostera_marina

MGLQEEFEEYAVKAKTLPENTTNESKLILYGLYKQATVGPVDTSRPGIFNQRDRAKWDAWKAVEEKSKDEAMGDYIIKVKQLLEESA

>Zosma74g01040_Zostera_marina

MELQEDFEEHAKKIMTLTEEPSNDDKLILYGLFKQASFGPVTTDRPGIFKLKERAKWDAWKAVEGKSKEDAMNDYIIKVKQMMEC

>Sphfalx0016s0055_Sphagnum_fallax

MGLKEDFDQAAKDALTLPESTTNEDKLILYGLFKVATVGKPETSRPGIFDPKGRAKWDAWKKVEDKSKDEAMQEYIVKVTQLKEA

>Sphfalx0295s0007_Sphagnum_fallax

MGLKKDFDQAAKDALTLPPSTTNDDKLVLYGLFKSATVGKPETSRPGIFDPKGRVKWDAWKMVEEKSKDEAMQDYILKVTQLKEA

>MBO95836_Euryarchaeota_archaeon

MIEQRFTEACAEVERVYTRLDNATVRKVYAYYKQATEGDVKGKRPSVLKLRDRIKYDSWSSISGMAMHDAMVAYVELVDNLTLQSEAISCDAREALLEK

>MAQ04628_Euryarchaeota_archaeon

MNLRKKFERAAKRVWKLKETPDDSTLLQLYALYKQATEGDARSPRPISXGMAGMVKWQAWRKLRGISVDEAMERYCNLVDKLMSRGANPSSS

>DAC36007_Marine_Group_II_euryarchaeote

MSIEKKFEKATKRVWRLKEKPDDMTLLELYALYKQATEGDATGPRPLSGGLAGMAKWRAWRKLRGTSNEEAMESYCAIVDRLMSGD

>AIF18224_Marine_Group_II/III_euryarchaeote

MSDGKASRSLRMRFERAAKKAWELPTRPSNEKLLEMYALYKQATEGDCEGKPRGGLRDRAKHKAWKGVAGTTEADAMQGYCEIVDSLMG

>MAZ23250_Euryarchaeota_archaeon

MTGKASQTLRLRFKRASKKAWELPEKPTNEQLLDLYALFKQATEGDCEGRPRGGLKERAKWKAWNSISGTSESDAMERYCEMVDSLMGTN

>TFG05618_Lokiarchaeota_archaeon

MSVDNGKTLKSEFEEAIARSDKLPKQPVDTQLELYGLYKQALFGDVTGERPGRLKVKDRAKFDNWESRKGMLKEDAMKAYITLIEKLEQEKK

>WP_014434299_Caldilinea_aerophila_Chloroflexi

MSDLKTQFEQAARESTELPSRPDNQTLLQLYALYKQATVGDVNTKRPGMTDFVGRAKWDAWEKLKGMTTEAAMKAYIDLVNQLKAKG

>WP_138771349_Alcanivorax_gelatiniphagus_gamma

MADTARFEQAQKDVKTLNKKPSNDDLLFLYSHYKQGSEGDVSGKRPGMLDMVGRAKYDAWAKLKGMSKDDAMGKYIDKVESLLKTHK

>WP_110265910_Sinimarinibacterium_flocculans_gamma

MADTKAAFEQAQKDVKTLTKRPGNEDMLFLYAHFKQAAEGDVKGSRPGMLDMVGRAKYDAWAKLKGTKADDAMKKYVDKVATLLKSHK

>WP_028007895_Solimonas_flava_gamma

MADLKADFTKAQAEVKTLTKRPGNDDMLFLYAHYKQAAEGDVSGSRPGMLDMVGRAKYDAWAKLKGTKADEAMKKYIDKVAALLKTHK

>WP_037336982_Salinisphaera_hydrothermalis_gamma

MADTQAEFEQAQKDVETLSRRPSNDDLLALYAHYKQATAGDVSGKRPGMTDFKGRAKYDAWASLKGMDAEKAMQGYITKVGALFDAERDR

>WP_091532065_Fontimonas_thermophila_gamma

MADLKAQFAQAQQDVKTLTRRPDNDTMLFLYAHYKQATEGDVHGSRPGMLDMVGRAKYDAWAKLKGMKTDEAMKKYIDKVAALLKTHK

>WP_047885692_Photobacterium_ganghwense_gamma

MADLHSQFEQAQANIKTLTQRPTNDELLALYALFKQATEGDVSGKRPGMFDFKGAAKYDAWEQLKGTNAENAMQQYIDKVAELATQYA

>WP_090197211_Pseudomonas_pohangensis_gamma

MSTFEKAQADIKTLKSRPGNDDMLFLYAHYKQGSAGDVSGKRPGMLDMVGRAKYDAWAKLEGLDSAEAQARYVSKVKELLKADG

>WP_029921280_Nevskia_soli_gamma_gamma

MADLKAQFEKAQADVKTLTKRPGNEDMLTLYALFKQGSQGDVSGSRPGMLDMVGRAKFDAWTKLKGTGKDAALQKYIDKVAELLKTHK

>WP_043768677_Algiphilus_aromaticivorans_gamma

MSDLKSAFEQAQADVKTLTERPSNDDLLALYAHFKQATDGDVSGKRPGMLDMVGRAKYDAWAGLKGVSKDDAMQKYIDKVNELLGR

>WP_018161031_Smaragdicoccus_niigatensis_Actinobacteria

MSELADEFTQAQEDVKQLSKRPDNDSLLFLYAHFKQASAGNASGKRPGMFDVVGRAKYDAWAKLSGTSSEDAMTAYIGKVRELQAADQ

>WP_040732400_Nocardia_tenerifensis_Actinobacteria

MATGLHNPEFTQLAEQVKSLSSKPSNDTLLRLYAHFKQATVGDNDTSAPGMFDLTGKAKWNAWNDLKGMSQDEATSNYIQIARDTIAQDS

>WP_023544486_Streptomyces_roseochromogenus_Actinobacteria

MSDATSTGNEEFVTLGEKIKQLTSKPDNDTLLALYAHYKQATKGDNDTDAPGMFDLTGKAKWNAWNELRGMSKEQAQERYIQIAKATISSHS

>WP_030200352_Corynebacterium_variabile_Actinobacteria

MAGAEFEQAVEAVKGLTTDPGNEVKLRLYGLFKQATAGDATGSRPGFTNPVGRAKYDAWAKLSGTSPEAAEEQYVAIVRDLTAG

>WP_068423517_Rhodococcus_kyotonensis_Actinobacteria

MSDLDASFQQAQIDVKTLTTKPSNDDLLNLYSLYKQGSNGDVTGKRPGRLDMVNRAKYDAWAKLEGTDQESAKQSYVDLVDRLLGR

>WP_048547342_Tetrasphaera_jenkinsii_Actinobacteria

MPSPAFEPSPAFDEAVAAVKTLTADPGNDVKLTLYALYKQVTEGDVSGKRPGMFDLVGRAKYDAWQKLAGTSAADAEQAYIAQVDELLA

>WP_034716401_Intrasporangium_chromatireducens_Actinobacteria

MASDFESAVAAVGRLEKDPGNDVKLRLYALYKQATEGDVTGSRPGMMDFVGRAKYDAWAKLKGTPTEDAKLEYARIVDGLLPP

>KHL18146_Mumia_flava_Actinobacteria

MSELTAQFDQAQIDVKQLTERPGNLTLLRLYALFKQATDGDAHGDKPGFTDIVGKYKYDAWDALKGTSQDAAKQQYIELVESLKNGTAS

>WP_120235081_Collimonas_fungivorans_beta

MTIQAQFEQAQADSKTLSERPDNMTMLKLYALFKQGSSGDVTGERPGMTDFVGRAKWDAWNELKGTSQDDAQQQYVDLVEDLKD

>WP_104358340_Schlegelella_thermodepolymerans_beta

MSDLKARFEQAVAESKTLPEKPDNMTLLKIYALYKQATAGDVEGERPGMTDFVARAKWDAWNALKGKSAEEAMQEYIDLIESLK

>WP_058089394_Aquabacterium_parvum_beta

MSDLQAQFEKALADSKLLPAKPDNNTLLKIYSLFKQGSVGDVQGDRPGMMDFVGRAKYDAWAELKGKSQDAAKQAYIDLIESLKK

>WP_014427063_Rubrivivax_gelatinosus_beta

MPQLKAAFEKAVAESKSLPAKPDNATLLKIYSLYKQATVGDVEGDRPGFTDFVGRAKYDAWAGLKGTSANEAMQQYIDLIEDLK

>WP_085753311_Rhizobacter_gummiphilus_beta

MSDLATQFEAARADSKNLPEKPDNMTLLKLYAFYKQGSEGDVNTERPGMTQFVDRAKWDAWNELKGSSKESAQQGYIDLIESLK

>WP_104302599_Zhizhongheella_caldifontis_beta

MADLQAAFEKAVAESKTLPEKPDNMTLLKIYALYKQATVGDVEGSRPGFTDMVGRAKWDAWNALKGQSADEAKQSYIDLIESLK

>WP_047193708_Polyangium_brachysporum_beta

MSDLKARFDQAVAESKSLPEKPDNMTLLKIYALFKQASSGDVEGDRPGMTDFVGRAKWDAWNALKGKSNEQAMQDYIDLIESLK

>WP_076592091_Herminiimonas_arsenitoxidans_beta

MSLQEQFDQALADSKTLPERPDNLTLLKIYGLYKQASGGDATGEQPAMTDFVARAKWDAWNNLKGISKEDAMQQYIDLVEDLKA

>WP_090258327_Fabibacter_pacificus_Bacteriodetes

MSITAQFEEAVKNANSLTQRPSNEDLLKIYGLYKQATEGDNNTERPGGFDFKAAAKYNAWANLKGTSNEEAMQQYIDLINRLKSA

>WP_121811061_Mucilaginibacter_kameinonensis_Bacteriodetes

MTNLKDAFQSAVKESRELPSKPDNETLLRLYSLYKQATEGDINTGNPPGMFDFVAKAKYDAWLKLQGTSADDAMQQYINLVAQLSNKTN

>WP_028787056_Terrimonas_ferruginea_Bacteriodetes

MDLKVLFEKAVTESKQLTSRPSNEMLLRLYGLYKQATEGDVNTDPPGAFDFVNKAKYEAWSSLKGKSQDEAMKEYADLVEKLKD

>WP_101445955_Pontibacter_ramchanderi_Bacteriodetes

MATQEEFEQAVAQSKELTERPSNNILLQLYGLFKQATEGDVNTERPGGFDFKNIAKWDAWKSLQGTSQEDARAQYVQLVNSLRNA

>WP_091367495_Mucilaginibacter_mallensis_Bacteriodetes

MELKEQFDKAVADSKQLPSRPDNDTLLRIYSLYKQATEGDINAEPPGMFDFVAKAKYDAWTKLKGVSADDAMGQYIEVVEGLK

>WP_011529541_Deinococcus_geothermalis

MSFELQNAFEQAQQEVQGLSEKPRNDVLLKLYALYKQGTVGDVTGERPGGFDFVGSAKYDAWAKLRGLSREEAQREYVNLVETLKARR

>WP_124736557_Rhodobacteraceae_bacterium_alpha

MSDLQTLFAQAQQDVTSLAERPDNAAMLQLYSLFKQATSGDATGERPGMMDFVARAKFDAWEKFKGLSADEAMQKYIDLVAELKA

>MBT28230_Thalassobius_sp._alpha

MKEKFELAREEVQKLKSKPNNTELLKLYGLYKQATEGDVKGDRPGTFDIKGQFKYDFWKRYLGKGEEESMGEYIALVDTLKEKYGMEEA

>WP_127848065_Mesorhizobium_sp._alpha

DQSFADAQVDVKKLTSKPSNDDLLSLYSLYKQGSIGDATGKRPGRLDMVNRAKFDAWAKLEGTSQDSAKQQYVNLVTKLLA

>WP_047856308_Archangium_gephyra_delta

MALEDDFRSAQERVKTLKTRPSNDTLLELYSLFKQATEGDVQGKRPGMLDLKGRAKYDAWAGRKGVGREAAMQQYVALVERLLRG

>AKV03253_Labilithrix_luteola_delta

MTHCAATKKIRPIDDVRSLARLGSNSLPSMDLTARFDDAQTRVKGLSKAPSNDDLLELYALYKQATVGDVSGSRPGMLDLKGRAKFDAWTKKKGVAKDAAMTSYVALVDRLLAS

>WP_095976253_Melittangium_boletus_delta

MSDLNAQFTKAQADVKTLSARPSNDTLLELYSLYKQATEGDVTGKRPGMLAVKDRAKYDAWEKVRGTGKTEAMQKYVDLVSRLLKK

>WP_080802002_Desulfamplus_magnetovallimortis_delta

MTDLNEQFETASKDVQTLTKKPGNDILLKLYSLFKQGSVGDVTGKRPGMTDFKGRAKYDAWSKCQGTPQDKAKQEYIDLVASLKG

>CAD19080_Stigmatella_aurantiaca_delta

MALEDDFRSAQERVKALKTRPSNDRLLELYSLFKQATEGDVQGKRPGILDVKERAKYDAWVGRKGLGRESAMQQYVALVERLLRG

>WP_071898123_Cystobacter_ferrugineus_delta

MSDLNARFQEAQVQVKTLTKRPDNDTLLQLYSLFKQATEGEVKGSRPGMMDFTGRAKYDAWAKLKGTPPDMAKQRYVELVNRLLGG

>RLA63917_Epsilonproteobacteria_bacterium

MEMDLKEEFEKASETVMNLSERPSNEELLKLYSFYKQGTEGDVSGKRPGMINLKGRAKYDAWAKLEGMHAQEAQKNYVELVANLLGK

>MBQ49925_Zetaproteobacteria_bacterium

MSTKEEFQQAAVDIKSLSSKPSNEDLLALYALFKQGNQGQVTGSRPGMMNLVGRAKYDAWKALGTMPEADAQLNYIAKVKELITADN

>sp|P07107|ACBP_Bos_tausus

MSQAEFDKAAEEVKHLKTKPADEEMLFIYSHYKQATVGDINTERPGMLDFKGKAKWDAWNELKGTSKEDAMKAYIDKVEELKKKYGI
